# Organocatalytic atroposelective heterocycloaddition to access axially chiral 2-arylquinolines

**DOI:** 10.1038/s42004-021-00580-5

**Published:** 2021-10-13

**Authors:** Gongming Yang, Shaofa Sun, Zhipeng Li, Yuhan Liu, Jian Wang

**Affiliations:** 1 School of Pharmaceutical Sciences, Key Laboratory of Bioorganic Phosphorous Chemistry and Chemical Biology (Ministry of Education), Tsinghua University, Beijing, 100084 China; 2 College of Chemistry and Biological Sciences, Hubei University of Science and Technology, Xianning, Hubei 437100 China

**Keywords:** Asymmetric catalysis, Synthetic chemistry methodology, Organocatalysis

## Abstract

Axially chiral heterobiaryls play a vital role in asymmetric synthesis and drug discovery. However, there are few reports on the synthesis of atropisomeric heterobiaryls compared with axially chiral biaryls. Thus, the rapid enantioselective construction of optically active heterobiaryls and their analogues remains an attractive challenge. Here, we report a concise chiral amine-catalyzed atroposelective heterocycloaddition reaction of alkynes with *ortho*-aminoarylaldehydes, and obtain a new class of axially chiral 2-arylquinoline skeletons with high yields and excellent enantioselectivities. In addition, the axially chiral 2-arylquinoline framework with different substituents is expected to be widely used in enantioselective synthesis.

## Abstract (web summary)

2-Arylquinoline derivatives are key components of many enantioselective catalysts, but existing synthetic routes are few in number. Here atroposelective aminocatalytic aza-Michael addition of amino-benzaldehydes to iminium intermediates yields single-enantiomer 2- arylquinoline derivatives with high selectivity

## Introduction

**Fig. 1:**
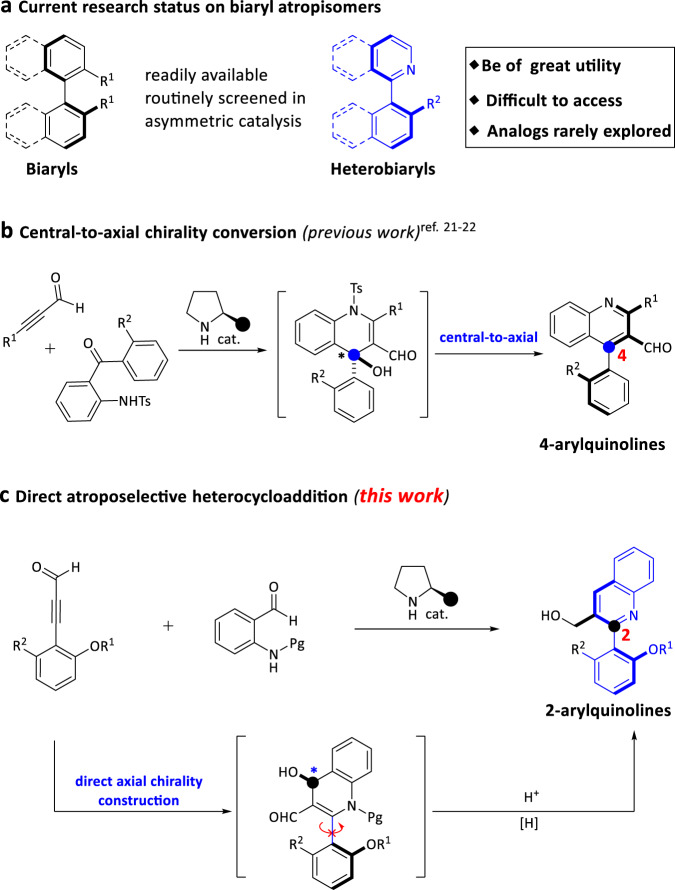
Current research on axial chirality and selected molecules. **a** Current research status on axially chiral biaryls. **b** Organocatalytic synthesis of 4-arylquinolines via central-to-axial chirality conversion (previous work). **c** Direct to 2-arylquinolines via atroposelective cycloaddition (this work).

Axially chiral biaryl scaffold is one of the most important structural units, which is widely found in many natural products^1,2^, bioactive molecules^3–5^, and functional materials^6,7^. Therefore, the study of axially chiral compounds has attracted extensive attention, and plays an important role in the development of chiral ligands^8,9^ and organic catalysts^10–12^. In particular, axially chiral 1,1′-bi-2-naphthol (BINOL) (Fig. 1a, *R*^1^ = OH) and its derivatives, as the most successful catalysts and ligands in enantioselective synthesis, have achieved great progress^13–15^. Despite advance has been made in the study of thesis axially chiral biaryls, there are still some limitations in the preparation of axially chiral heterobiaryls^16–22^. Atropisomeric isoquinoline derivatives (1-(isoquinolin-1-yl)naphthalen-2-ol, Fig. 1a, *R*^2^ = OH) has emerged as a unique backbone of several famous catalysts and ligands (e.g., N,O-ligand, QUINOX) in asymmetric catalysis^23,24^. Especially, Noyori’s BINAP catalyst^25^ is widely used in industrial and pharmaceutical production. In sharp contrast to axially chiral BINOLs, the atroposelective synthesis of axially chiral isoquinoline derivatives has not been greatly explored. To date, there are few methods for assembling axially chiral isoquinoline derivatives, most of the thesis reports are focus on transition-metal-catalyzed direct cross-coupling reaction^26,27^, [2 + 2 + 2] cycloaddition^28,29^ and recently reported (dynamic) kinetic resolution/transformation strategy^30–34^. However, building upon the increasing demand for this type of catalysts and ligands, a versatile, practical, and scalable method for the synthesis of optically pure axial chiral isoquinoline and corresponding analogues is undoubtedly an urgent need in this field. In addition, from the perspective of structural diversity, nonclassical isoquinoline-type analogues will offer more possibilities for catalyst/ligand development and drug discovery.

Although quinoline skeleton widely exists in bioactive compounds^35^, in sharp contrast to atropisomeric 2-arylisoquinoline, the research on axial chiral quinoline skeleton is very limited^34^. Meanwhile, most of the reports focused on the atroposelective synthesis of aryl-C4-^36,37^, C5-^38^, or C8-quinoline^39^ skeletons, in which the distant from the chiral axis of the decisive nitrogen atom for coordination with metal center leading to the difficulty in stereo-induction, which sometimes restrict their further applications in the area of asymmetric synthesis greatly. To face this issue, Tan and coworkers^40^ uncovered a chiral phosphoric acid-catalyzed atroposelective [4 + 2] cycloaddition to synthesize IAN analogs *via* the intermediate of vinylidene *ortho*-quinone methide. This is the only report on the atroposelective synthesis of 2-arylquinoline skeletons. Overall, the catalytic atroposelective synthesis of axially chiral 2-arylquinolines in a highly atroposelective manner remains an attractive challenge.

In the past two decades, chiral amine catalyst has attracted considerable attention in asymmetric catalysis due to its advantages of operational simplicity, low toxicity, and minimal impact on the environment^41–46^. However, most of the reports solely focus on the assembly of central chirality, and the amine-catalyzed enantioselective construction of axial chirality is still in its infancy^47–50^. The Sparr group constructed a series of axially chiral biaryl skeletons via chiral amine-catalyzed asymmetric aldol condensation^47–49^. Recently, Cheng^20^ and Wang^21^ reported the synthesis of axially chiral 4-arylquinoline skeletons via amine-catalyzed asymmetric heterocycloaddition of ynals with 2-(tosylamino)aryl ketones, followed by aromatization and central-to-axial conversion (Fig. 1b). As part of our group ongoing efforts on organocatalytic synthesis of axially chiral molecules^51–53^, we have successfully reported carbene-catalyzed atroposelective desymmetrization of biphenols, the [3 + 3] annulation of cyclic 1,3-diones with ynals and the kinetic resolution of anilides, resulting in valuable axially chiral biaryl amino alcohols (NOBIN analogues), α-pyrone-aryls, and isoindolinones, respectively. Despite the aforementioned achievements, unsolved challenges and the continuing demands for atropoisomers continue to drive us to develop more revolutionary protocols. We herein firstly report a chiral amine-catalyzed atroposelective heterocycloaddition to offer a class of axially chiral 2-arylquinolines (nonclassical isoquinoline-type analogues) with high yields and excellent enantioselectivities.

## Results

### Optimization of the reaction conditions

We commenced our study with the model reaction of 3-(2-methoxynaphthalen-1-yl)propiolaldehyde **1a** and N-(2-formylphenyl)-4-methylbenzenesulfonamide **2a**. The key results of reaction optimization are summarized in Table 1. First, the chiral secondary amine catalysts **A–C** derived from L-proline with different steric size on the silyl groups were tested. As a result, these catalysts provided the target product **3a** in high yields but with very low enantioselectivities (Table 1, entries 1–3). Inspired by the elegant work reported by the Wang group^54,55^, the catalyst **D** was selected for initial test under the model reaction. Pleasingly, the desired axially chiral 2-arylquinoline **3a** was separated with high yield (89%) and moderate enantiomeric ratio (er) (75:25) (Table 1, entry 4). To further improve the enantiocontrol, we investigated the effect of different O/N-protecting groups on substrates (**1b**, **1c**, and **2b**). It is surprising that the enantiomeric ratio of the reaction was arised to 96:4 and the yield was still kept high (91%) (Table 1, entries 5-7). In the case of the best catalyst (**D**) and suitable substrate (**2b**), the influences of solvents and catalyst loading were then examined (Table 1, entries 8–10). Finally, the optimal condition and procedure were obtained as follows: adding **1c** (1.2 equiv.) and **2b** (1.0 equiv.) to the mixture of catalyst **D** (10 mol%) and CHCl_3_ (0.1 M) at room temperture and giving corresponding reaction time, the axially chiral **3c** was obtained with 91% yield and 96:4 er (Table 1, entry 7).

**Table 1:** Optimization of the reaction conditions^a^.


entry	cat.	solvent	yield (%)^*b*^	er^*c*^
1	**A**	CHCl_3_	91	48:52
2	**B**	CHCl_3_	89	49:51
3	**C**	CHCl_3_	87	49:51
4	**D**	CHCl_3_	89	75:25
5^d^	**D**	CHCl_3_	91	88:12
6^e^	**D**	CHCl_3_	90	94:6
7^e,f^	**D**	CHCl_3_	91	96:4
8^e,f,g^	**D**	CHCl_3_	86	96:4
9^e,f^	**D**	CH_2_Cl_2_	90	95:5
10^e,f^	**D**	MTBE	80	95:5
11^e,f^	**D**	Toluene	86	95:5
12^e,f,h^	**D**	CHCl_3_	78	96:4

^a^Conditions: **1a** (0.11 mmol), **2a** (0.10 mmol), catalyst (10 mol %), solvent (2.0 mL), room temperature, 24 h. After the reaction was complete, the reaction was cooled to 0 °C, then MeOH (0.5 mL) and NaBH_4_ (0.2 mmol) were added to the mixture and stirred for another 0.5 h at room temperature. At last, HOAc (5.0 equiv) was added to the mixture and stirred for another 2 h. ^b^Isolated yield after flash column chromatography. ^c^Determined by HPLC analysis using a chiral stationary phase. ^d^**1b** was used. ^e^**1c** was used. ^f^**2b** was used. ^g^HOAc was replaced with KHSO_4_ (5.0 equiv) for 6 h.^h^**D** (5 mol %) was used, 48 h.

### Substrate scope

**Fig. 2:**
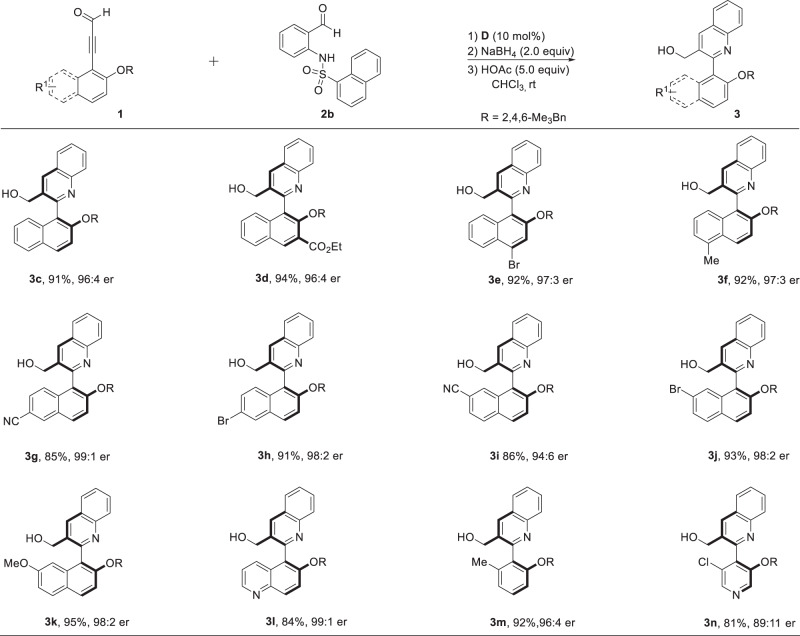
Scope of ynals. Reaction conditions: a mixture of **1c** (0.11 mmol), **2** (0.1 mmol) and catalyst **D** (10 mol%) in CHCl_3_ (1.0 mL) was stirred at room temperature for 24–36 h. After the reaction was complete, the reaction was cooled to 0 °C, then MeOH (0.5 mL) and NaBH_4_ (0.2 mmol) were added to the mixture and stirred for another 0.5 h at room temperature. At last, HOAc (5.0 equiv) was added to the mixture and stirred for another 2 h.

After obtaining the optimal conditions, we turned our attention to the substrate scope of ynals. An array of naphthalen-based propiolaldehydes bearing various *R*^1^ were tested (Fig. 2). The results show that the electronic and steric effects of the substituents at different positions on the aromatic rings have little impact on the reaction, and the corresponding products were produced in high yields and good to excellent enantioselectivities (**3c–3k**). Moreover, the quinoline-based ynal provided the coresponding product **3l** in 84% yield with 99:1 er. Notably, phenylpropiolaldehyde **1m** generated the corresponding biaryl (quinoline-phenyl) product **3m** with 92% yield and 96:4 er. Meanwile, a good level of er (89:11) was achieved for quinoline-pyridine-type biaryl product **3n** when pyridine-based propiolaldehyde **1n** was used.

**Fig. 3:**
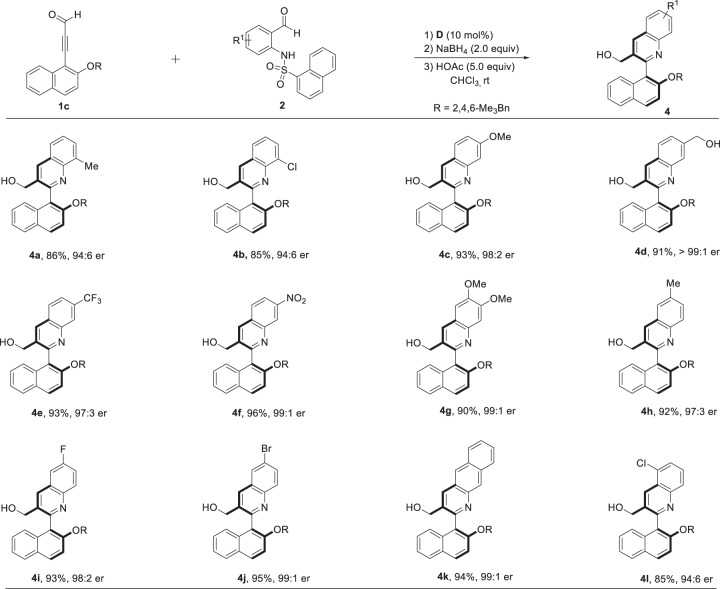
Scope of N-(2-formylphenyl)naphthalene-1-sulfonamides. Reaction conditions: a mixture of **1c** (0.11 mmol), **2** (0.1 mmol) and catalyst **D** (10 mol%) in CHCl_3_ (1.0 mL) was stirred at room temperature for 24–36 h. After the reaction was completed, the reaction was cooled to 0°C, then MeOH (0.5 mL) and NaBH_4_ (0.2 mmol) were added to the mixture and stirred for another 0.5 h at room temperature. At last, HOAc (5.0 equiv) was added to the mixture and stirred for another 2 h.

Encouraged by these results, we next examined the generality of *o*-aminoarylaldehyde **2**. As shown in Fig. 3, substrates bearing either electron-rich groups or electron-deficient groups reacted smoothly with **1c** affording the corresponding axially chiral products (**4c–4k**) in excellent yields (90–96%) and excellent er (96:4–>99:1). When steric hindrance was introduced at the *ortho* position of the substrate reaction site, the corresponding high yields (84–85%), and the high er (all 94:6) for product **4a**, **4b,** and **4l** were observed. The absolute configuration of **3k** was determined by X-ray crystallography (Fig. 2), and other products were assigned by analogy.

### Gram-scale synthesis and synthetic transformations

**Fig. 4:**
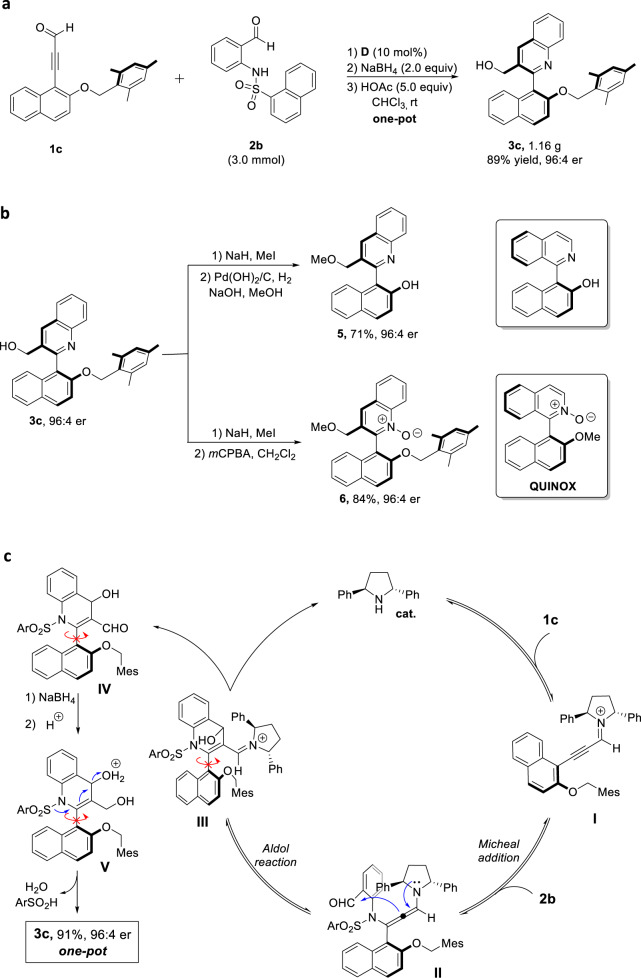
Gram-scale synthesis and synthetic transformations. **a** The gram-scale synthesis of **3c**. **b** The synthetic transformations of **3c**. **c** Plausible mechanism.

To evaluate the practicality of this protocol, a gram-scale reaction of **1c** with **2b** was carried out under standard condition (Fig. 4, 89% yield, 96:4 er), which indicated that the large-scale synthesis of enantioenriched 2-arylquinolines can be achieved. Synthetic transformations of **3c** were also illustrated in Fig. 4b. On the basis of methylation and hydrogenolysis, **3c** was easily converted into a versatile intermediatex axially chiral isoquinoline analogue **5** with a yield of 71%, and the er value is completely maintained. After methylation and oxidation, the axially chiral QUINOX analogue **6** (a potential Lewis base organocatalyst) was obtained in good yield without loss of enantiopurity.

A postulated reaction pathway is proposed in Fig. 4c. Initially, chiral secondary amine catalyst **D** added to alkynaldehyde **3c** and subsequently dehydrated to produce alkynylamine cationic intermediate **I**. Then **2b** reacted with **I** via aza-Michael addition to give axially chiral allenamine intermediate **II**, which underwent an intramolecular aldol reaction to give the chiral styrene intermediate **III**. Catalyst **D** was then released from intermediate **III** to produce chiral compound **IV**. Finally, compound **IV** was reduced by NaBH_4_ and then dehydrated in the present of acid in one pot to deliever the desired product **3c**.

## Discussion

In summary, we have successfully synthesized the axially chiral 2-arylquinoline analogues *via* catalytic heterocycloaddition of alkynaldehydes with *N*-protected *o*-aminoarylaldehyde *via* the formation of a critical intermediate (axially chiral styrene). In the presence of commercially available amine catalyst, this conversion can deliver a variety of axially chiral 1-aryl isoquinoline analogues with high yields and excellent er’s. The synthetic utility of this methodology is illustrated by further conversion to axially chiral QUINOX and isoquinoline analogues. Further studies on the application of the axially chiral 2-arylquinoline skeletons in asymmetric synthesis are currently underway in our laboratory.

## Methods

### Procedure for enantioselective syntheses of compound 3c

To a flame-dried Schlenk reaction tube equipped with a magnetic stir bar, was added the catalyst **D** (2.2 mg, 0.01 mmol), **1c** (39.4 mg, 0.12 mmol), and **2b** (0.10 mmol). The Schlenk tube was closed with a septum, CHCl_3_ (2.0 mL) was added. The mixture was then stirred at room temperature and monitored by TLC until **2b** was full consumed. Then the mixture was cooled to 0 °C and MeOH (1.0 mL) was added, subsequently, NaBH_4_ (11.3 mg, 0.3 mmol) was added slowly to the mixture, and stirred for 2.0 h at this temperature. After the completion of the reaction, as monitored by TLC, HOAc (5.0 equiv) was added to the mixture and then the mixture was warmed to room temperature and stirred for another 6 h. After the reaction was completed, as monitored by TLC, saturated NaHCO_3_ was added and stirred for another 0.5 h. Then the mixture was extracted with EtOAc. The combined organic layers were washed with water and brine, dried over anhydrous Na_2_SO_4_, filtered, and concentrated. The residue was purified by a silica gel flash chromatography (Hexane/EtOAc = 5:1) to afford the desired product **3c**.

### Procedure for enantioselective synthesis of compound rac-3c

To a flame-dried Schlenk reaction tube equipped with a magnetic stir bar, was added the catalyst ***rac-*A** (2.2 mg, 0.01 mmol), **1c** (39.4 mg, 0.12 mmol) and **2b** (0.10 mmol). The Schlenk tube was closed with a septum, CHCl_3_ (2.0 mL) was added. The mixture was then stirred at room temperature and monitored by TLC until **2b** was full consumed. Then the mixture was cooled to 0 °C and MeOH (1.0 mL) was added, subsequently, NaBH_4_ (11.3 mg, 0.3 mmol) was added slowly to the mixture and stirred for 2.0 h at this temperature. After the completion of the reaction, as monitored by TLC, HOAc (5.0 equiv) was added to the mixture and then the mixture was warmed to room temperature and stirred for another 6 h. After the reaction was completed, as monitored by TLC, saturated NaHCO_3_ was added and stirred for another 0.5 h. Then the mixture was extracted with EtOAc. The combined organic layers were washed with water and brine, dried over anhydrous Na_2_SO_4_, filtered, and concentrated. The residue was purified by a silica gel flash chromatography (Hexane/EtOAc = 5:1) to afford the desired product **3c**.

## Supplementary information

Description of Additional Supplementary Files

Supplementary Information

Supplementary Data 1

Supplementary Data 2

## Data Availability

The authors declare that the data supporting the findings of this study are available within the article and Supplementary information file, or from the corresponding author upon reasonable request. The supplementary crystallographic data for this paper could be obtained free of charge from The Cambridge Crystallographic Data Centre (**3k**: CCDC 2070558) via www.ccdc.cam.ac.uk/data_request/cif.
