# Supplementary material for: Organocatalytic atroposelective heterocycloaddition to access axially chiral 2-arylquinolines
Source: Commun Chem. 2021 Oct 13;4:144. doi: 10.1038/s42004-021-00580-5 (PMC9814953; doi:10.1038/s42004-021-00580-5)
Supplement: Supplementary file 1 — Description of Additional Supplementary Files [file 42004_2021_580_MOESM1_ESM.pdf]

Description of Additional Supplementary Files

File Name: Supplementary Data 1

Description: Crystallographic cif data of 3k

File Name: Supplementary Data 2

Description: Crystallographic fcf data of 3k
