# Supplementary material for: Organocatalytic atroposelective heterocycloaddition to access axially chiral 2-arylquinolines
Source: Commun Chem. 2021 Oct 13;4:144. doi: 10.1038/s42004-021-00580-5 (PMC9814953; doi:10.1038/s42004-021-00580-5)
Supplement: Supplementary file 2 — Supplementary Information [file 42004_2021_580_MOESM2_ESM.pdf]

Supporting Information

Organocatalytic Atroposelective Heterocycloaddition to Access Axially Chiral 2-Aryquinolines

Gongming Yang, Shaofa Sun*, Zhipeng Li, Yuhan Liu, Jian Wang*

Contents:

NMR Spectra 2

HPLC Spectra 59

General information 88

General procedure for the synthesis of **1** 88

Characterization data of 1 90

General procedure for the synthesis of **2** 95

Characterization data of **2** 96

Supplementary Table 1. Opitimization of the reaction conditions. 101

Experimental measurement of rotation barrier of **3c** 102

General procedure for preparation of compounds **3** and **4** 104

Characterization data of **3** and **4**. 105

Gram-scale synthesis and synthetic transformations 121

Proposed mechanism 124

Crystal structure of **3k** 126

Supplementary References 128

NMR Spectra

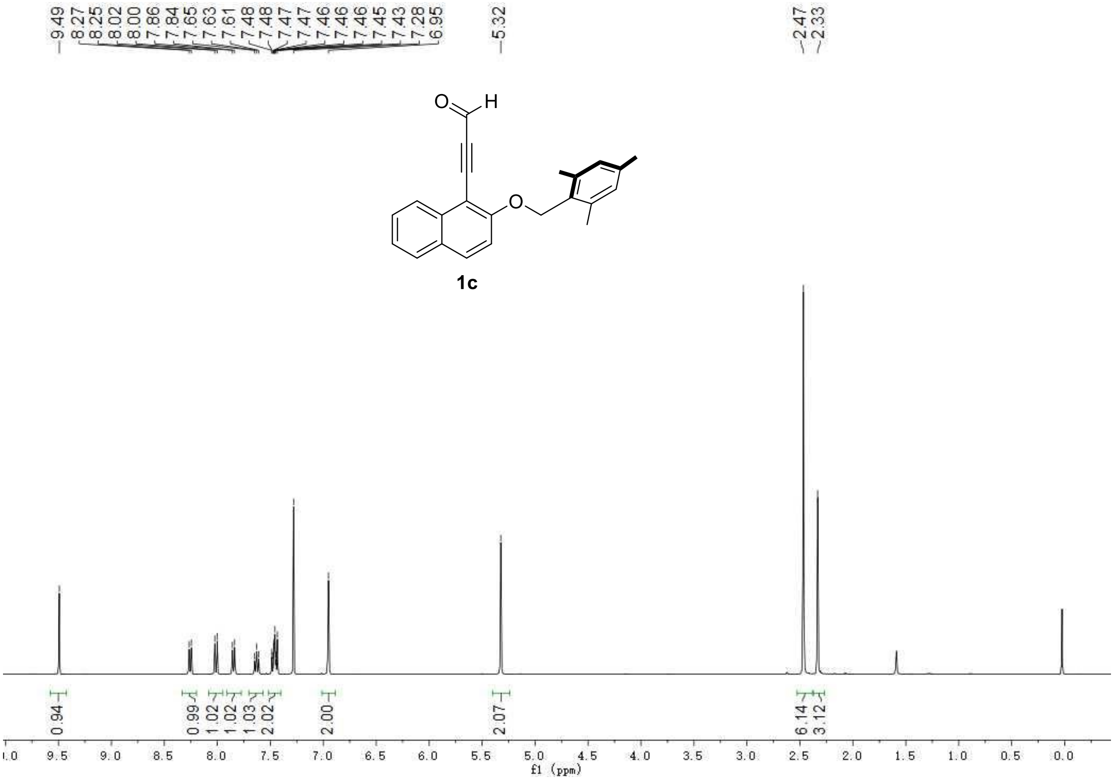

Supplementary Figure 1. ^{1}H NMR Spectra of **1c**

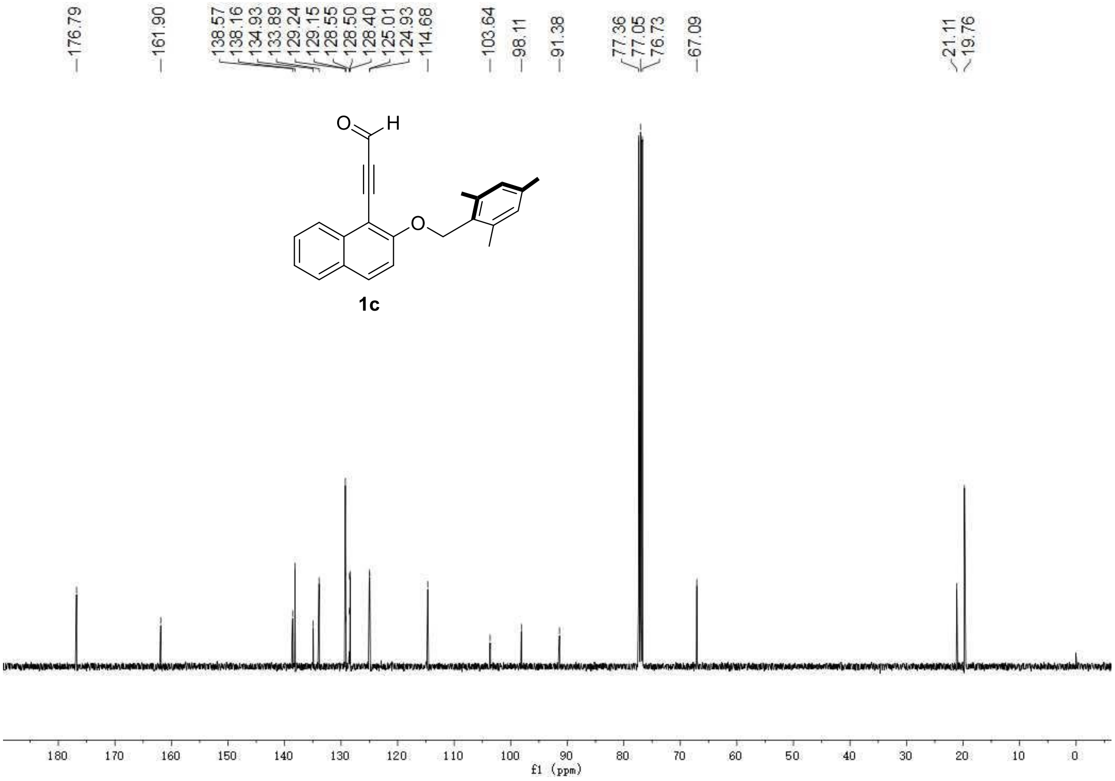

Supplementary Figure 2. ^{13}C NMR Spectra of **1c**

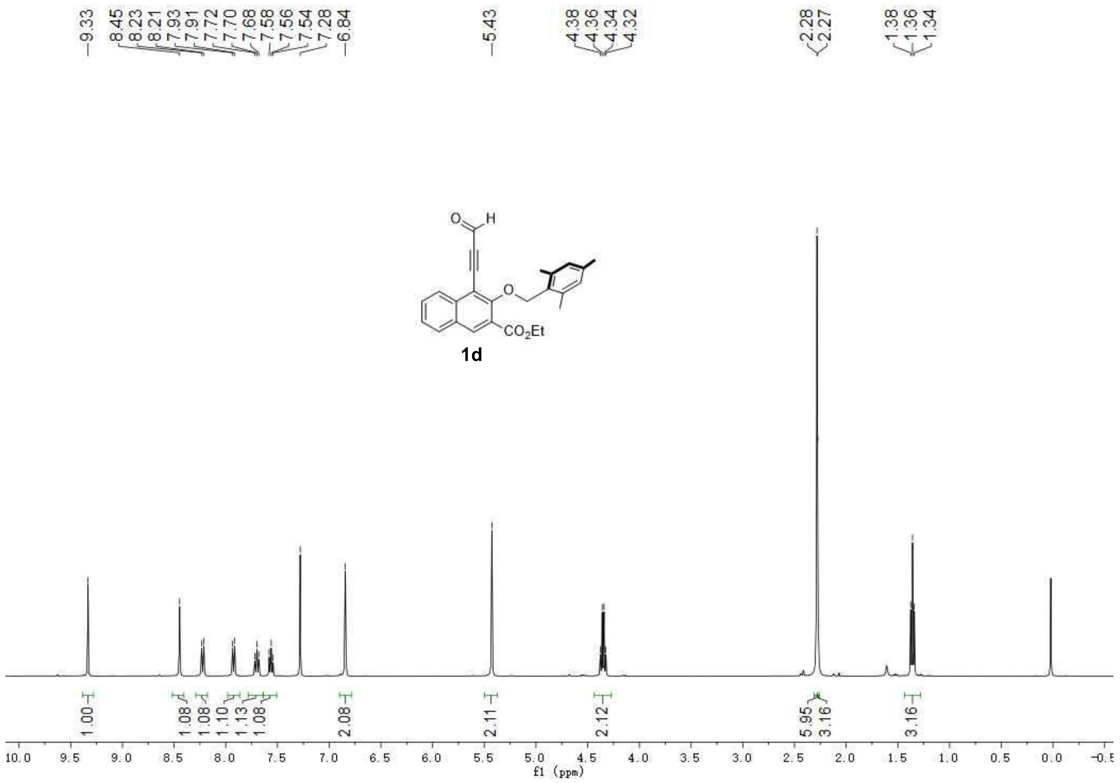

Supplementary Figure 3. ^{1}H NMR Spectra of **1d**

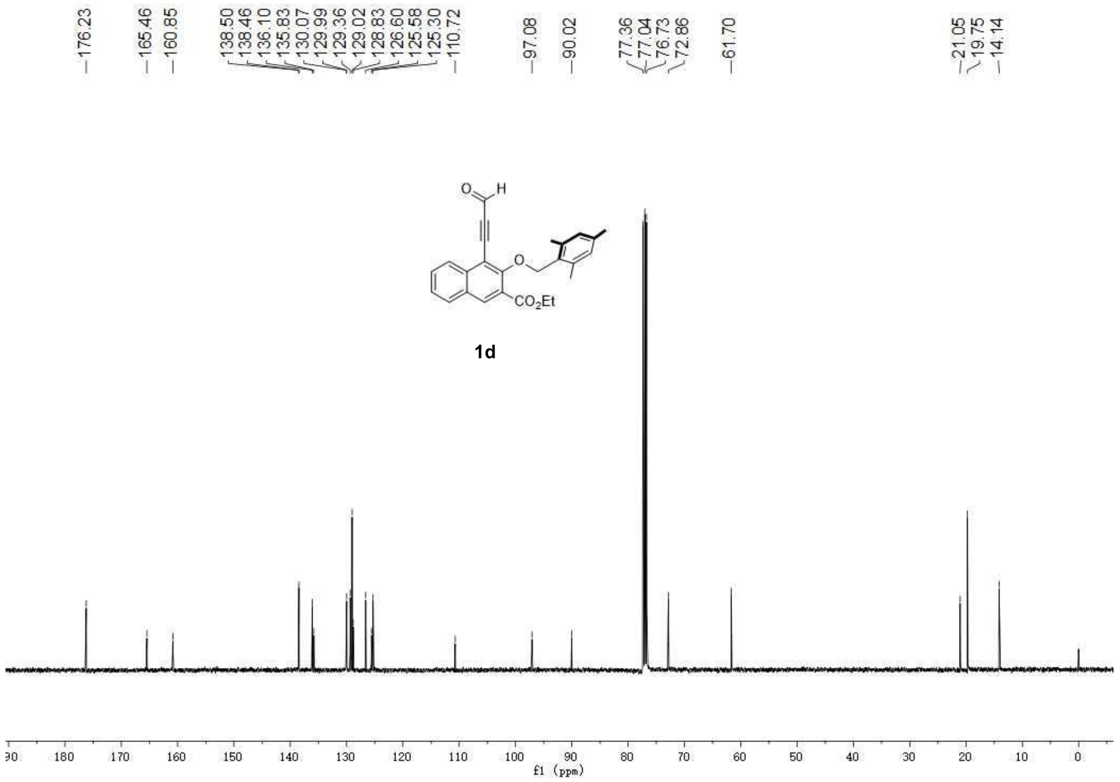

Supplementary Figure 4. ^{13}C NMR Spectra of **1d**

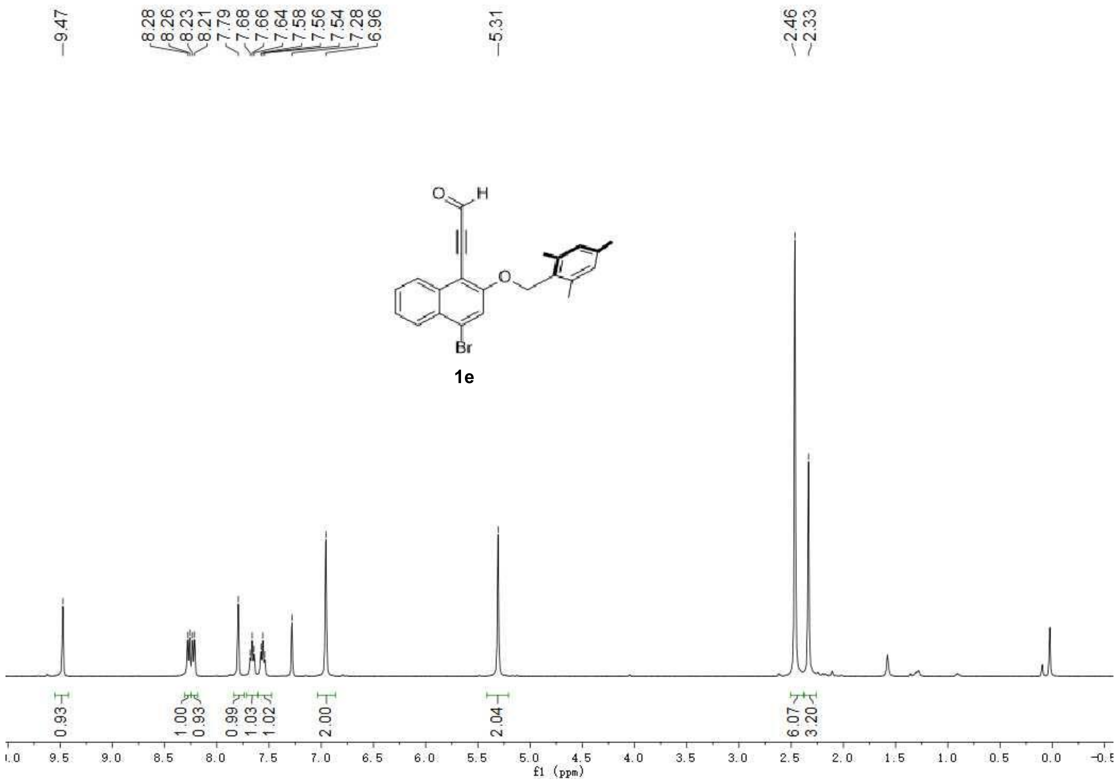

Supplementary Figure 5. ^{1}H NMR Spectra of **1e**

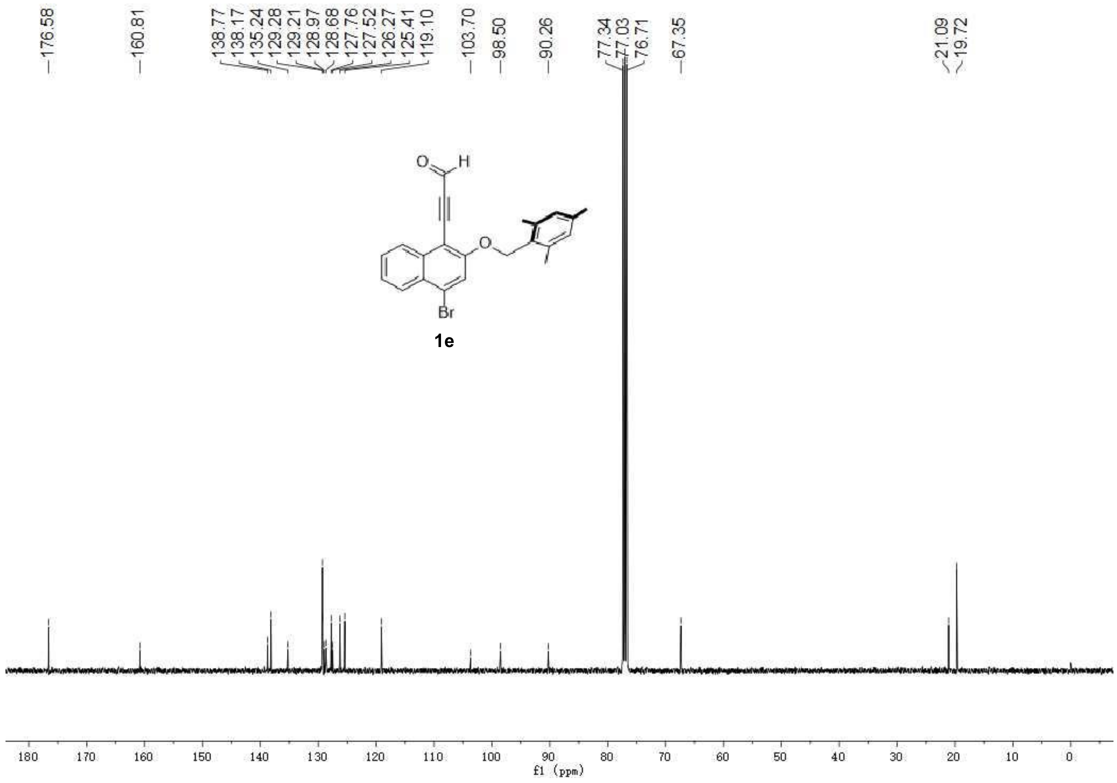

Supplementary Figure 6. ^{13}C NMR Spectra of **1e**

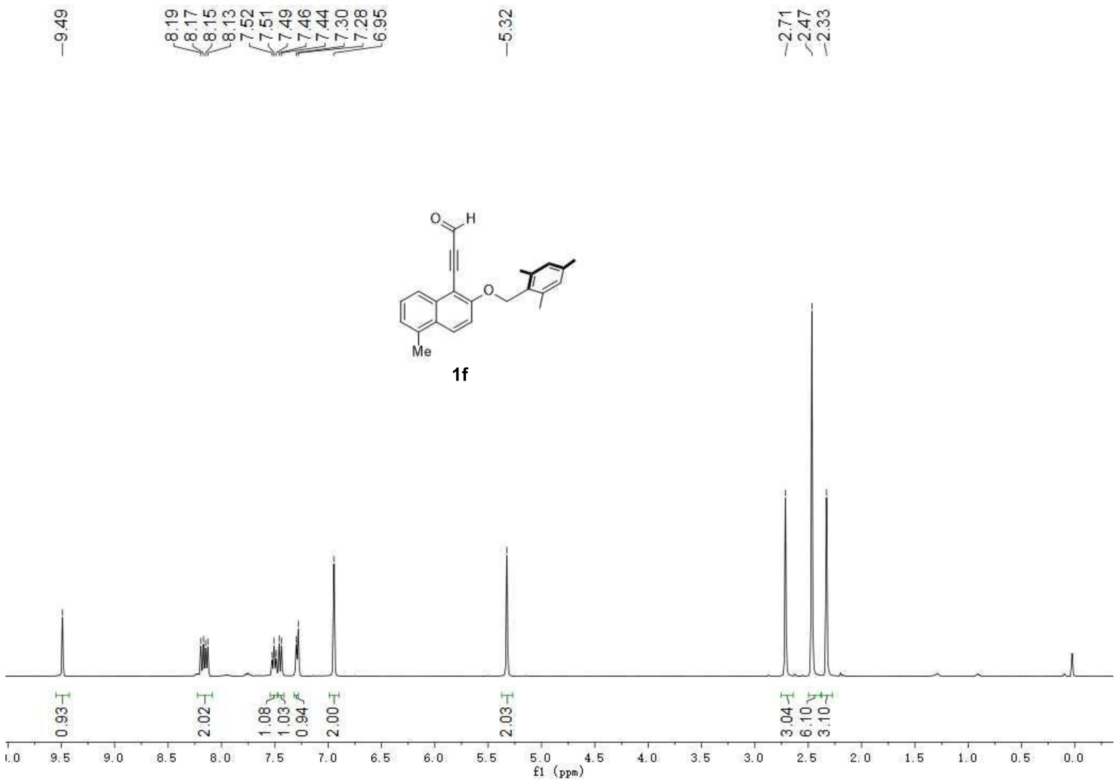

Supplementary Figure 7. ^{1}H NMR Spectra of **1f**

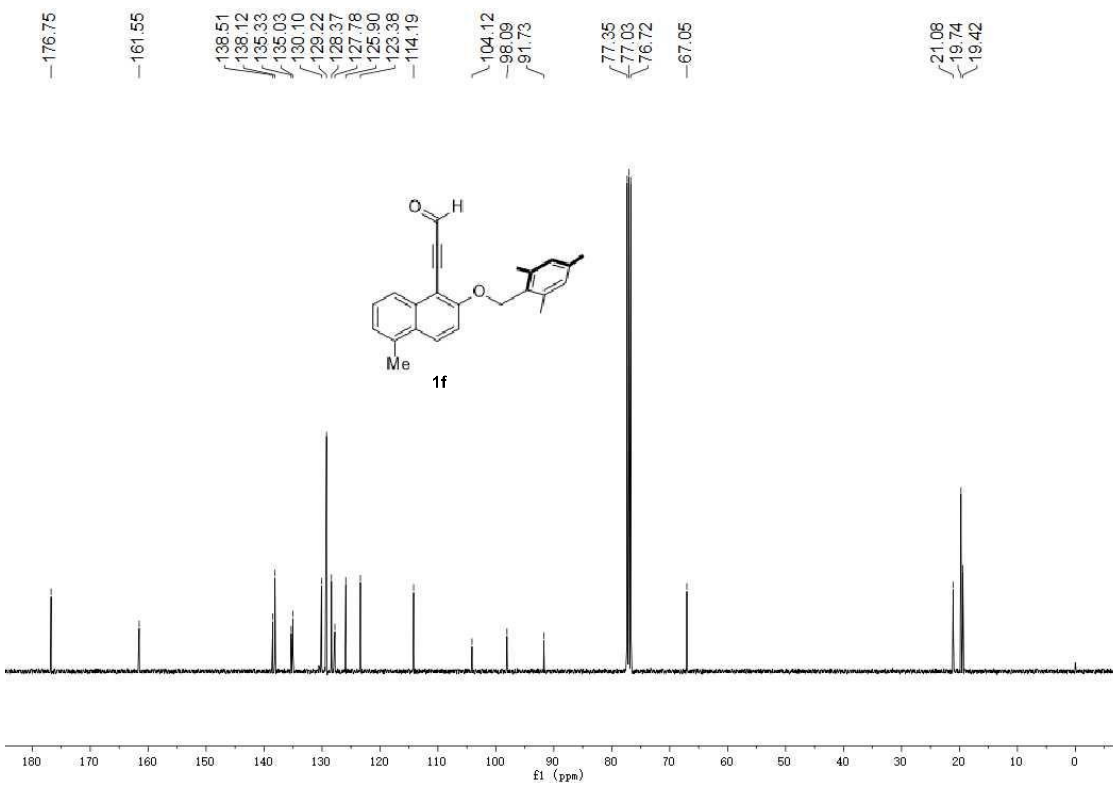

Supplementary Figure 8. ^{13}C NMR Spectra of **1f**

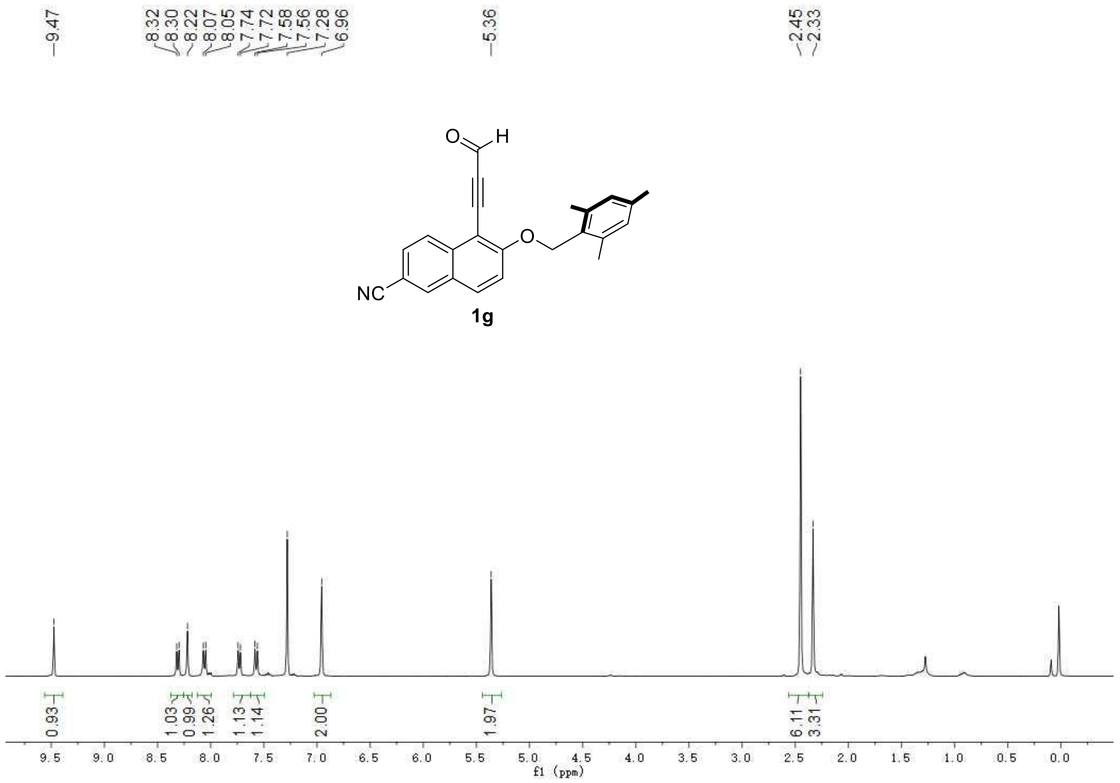

Supplementary Figure 9. ^{1}H NMR Spectra of **1g**

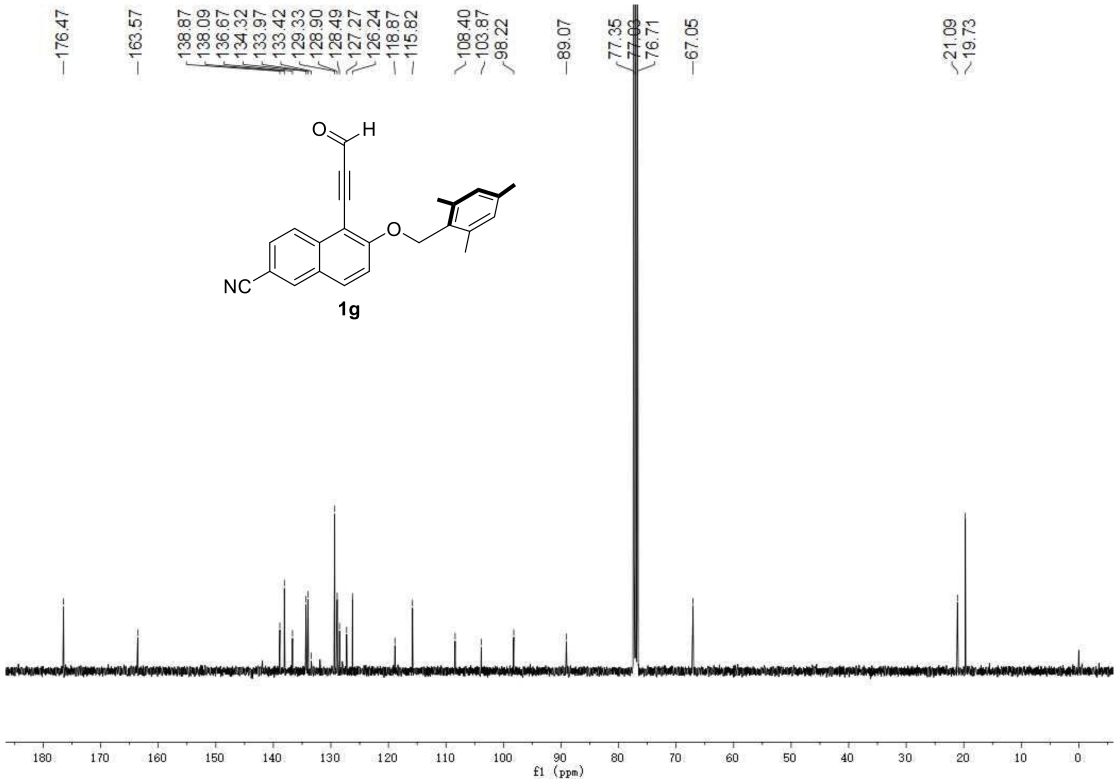

Supplementary Figure 10. ^{13}C NMR Spectra of **1g**

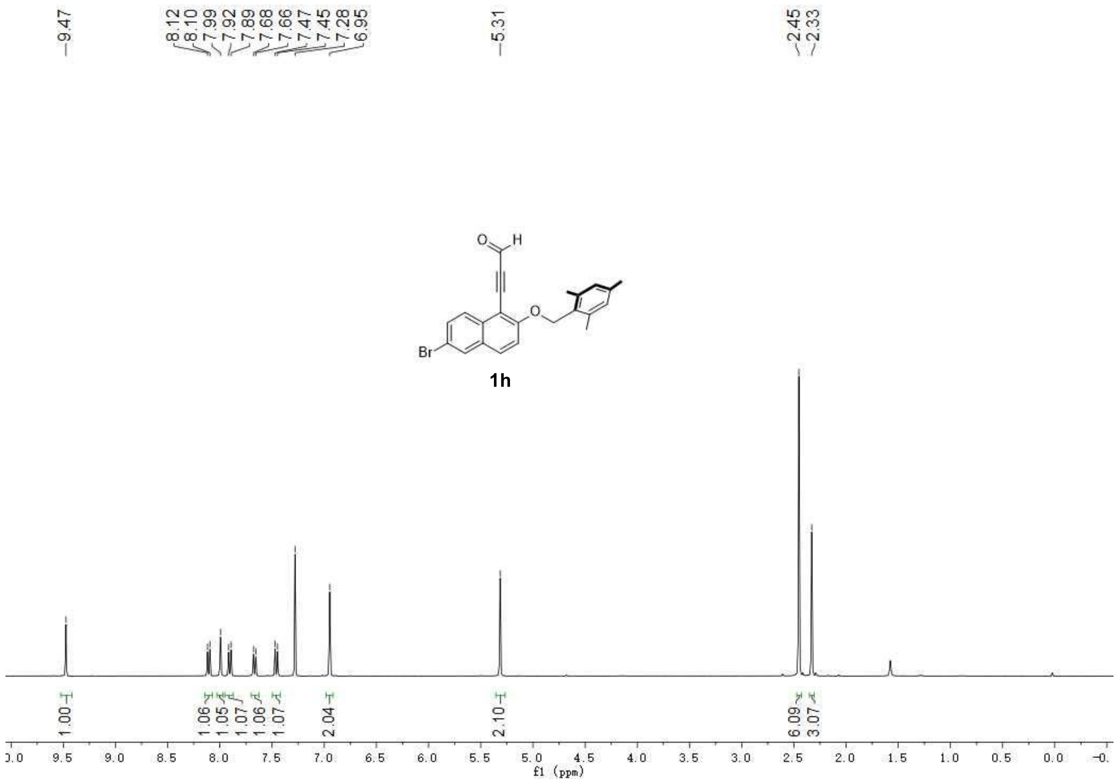

Supplementary Figure 11. ^{1}H NMR Spectra of **1h**

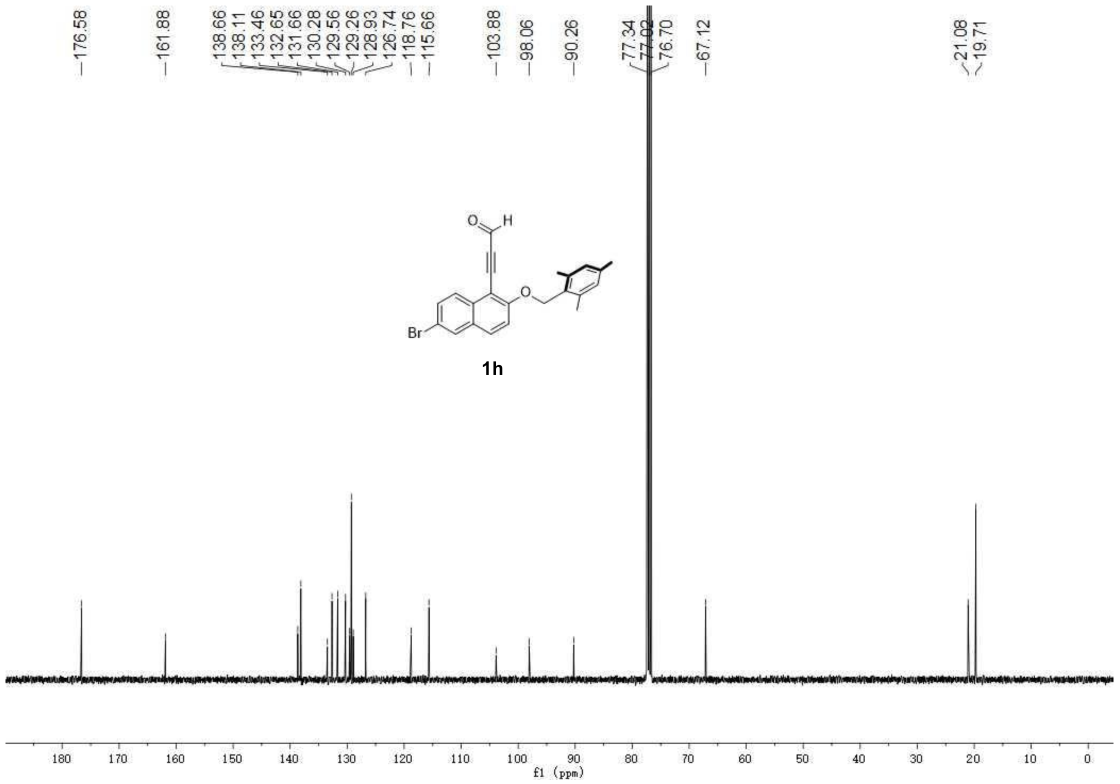

Supplementary Figure 12. ^{13}C NMR Spectra of **1h**

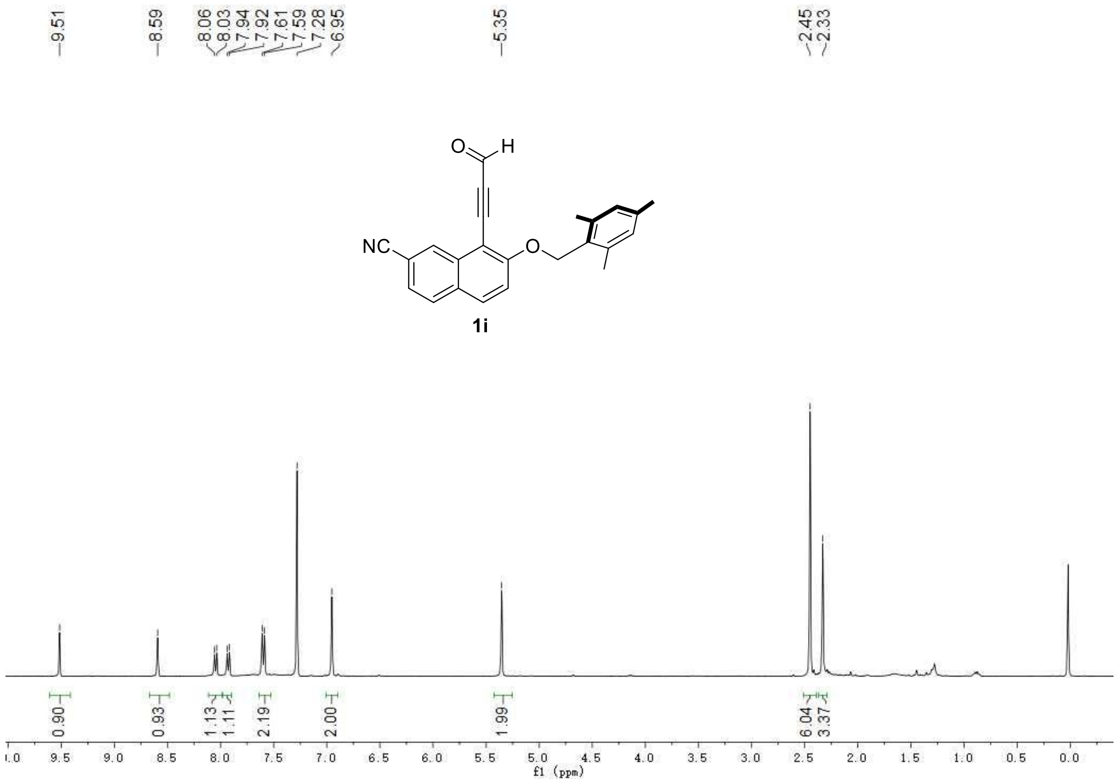

Supplementary Figure 13. ^{1}H NMR Spectra of **1i**

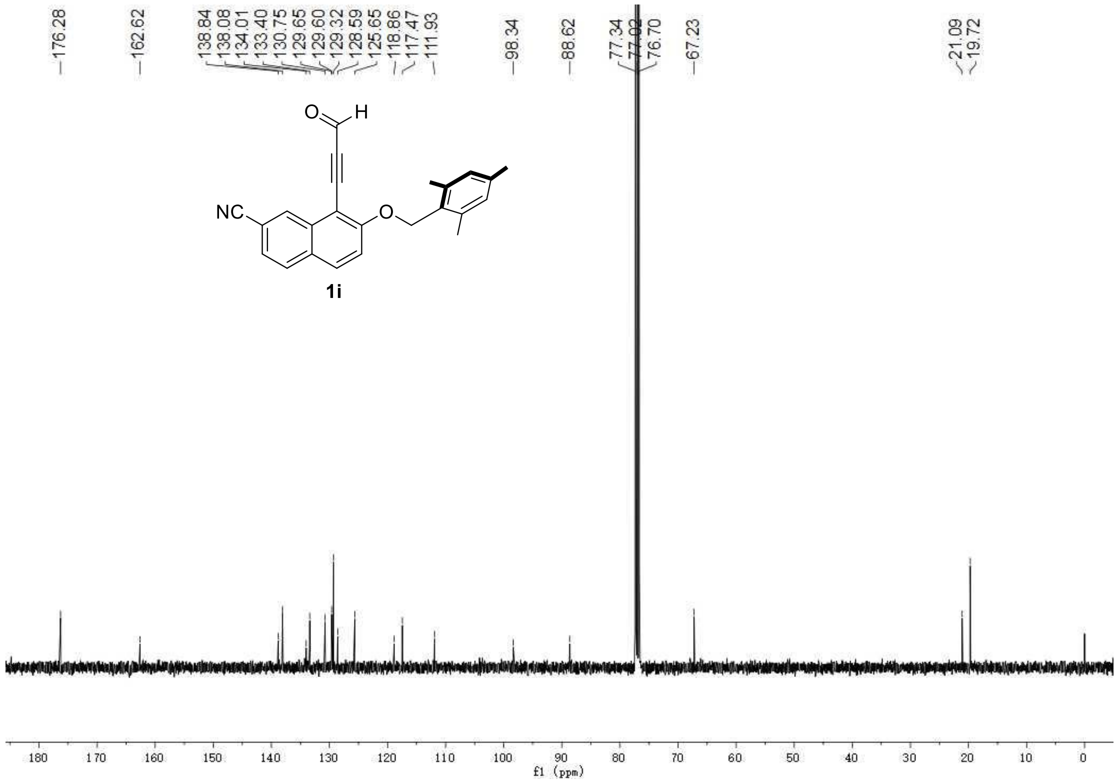

Supplementary Figure 14. ^{13}C NMR Spectra of **1i**

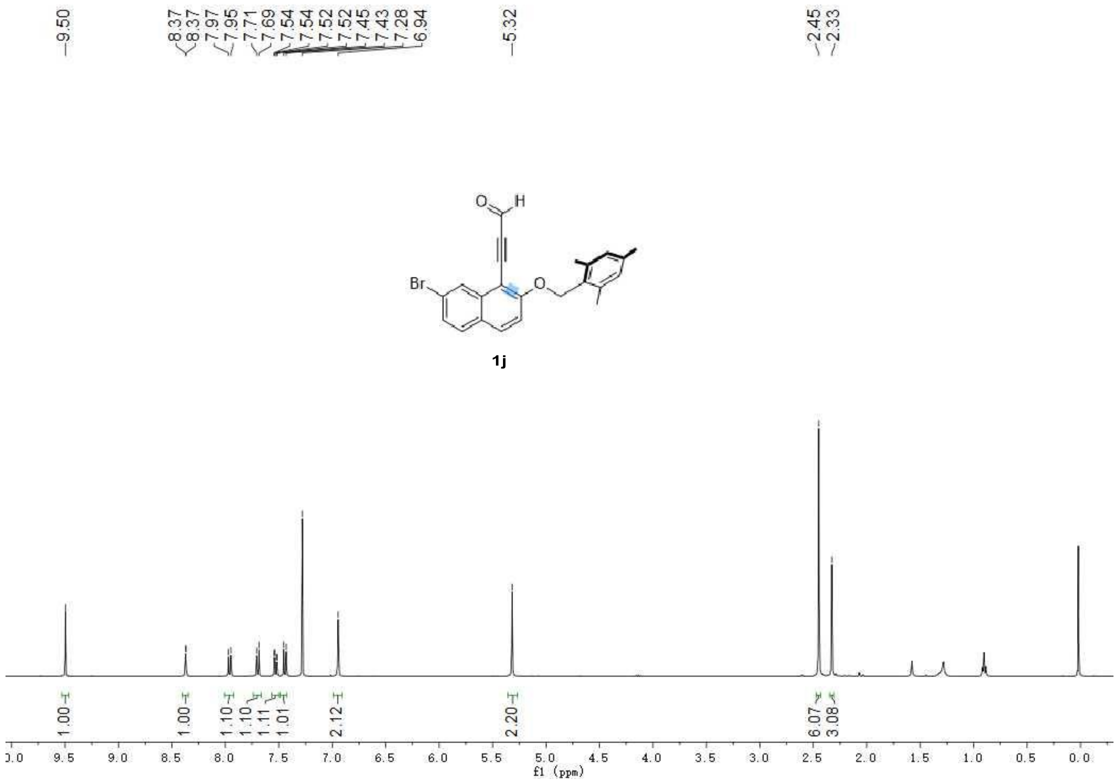

Supplementary Figure 15. ^{1}H NMR Spectra of **1j**

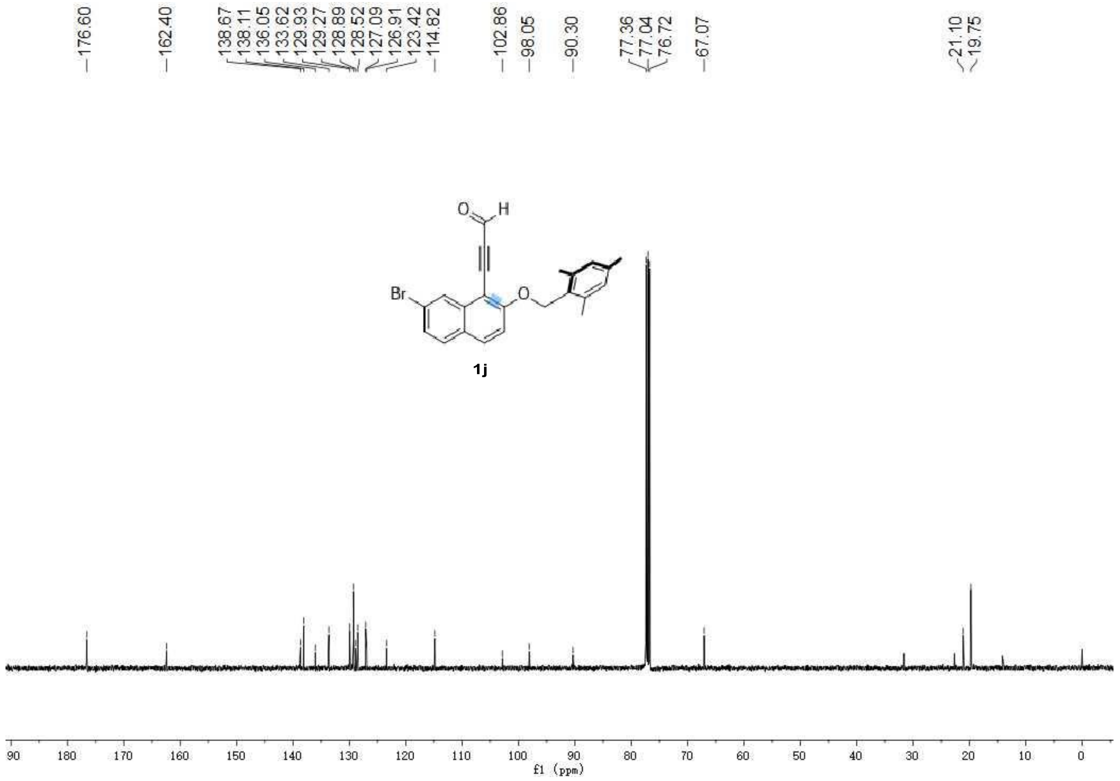

Supplementary Figure 16. ^{13}C NMR Spectra of **1j**

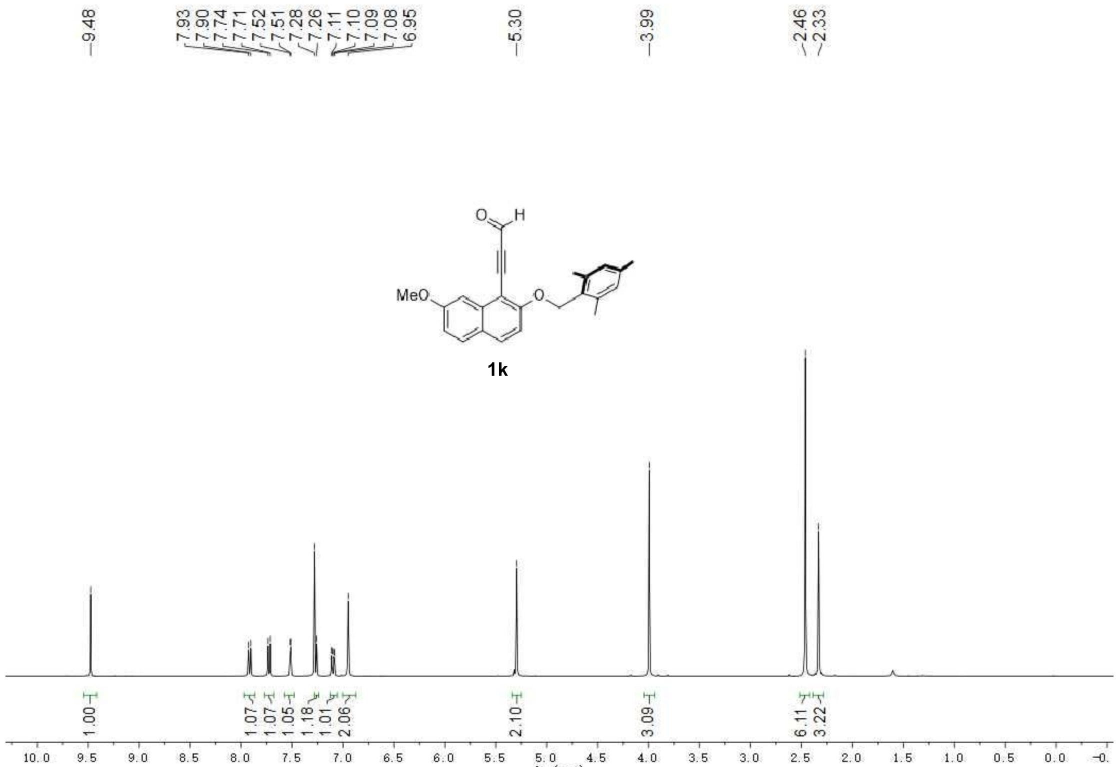

Supplementary Figure 17. ^{1}H NMR Spectra of **1k**

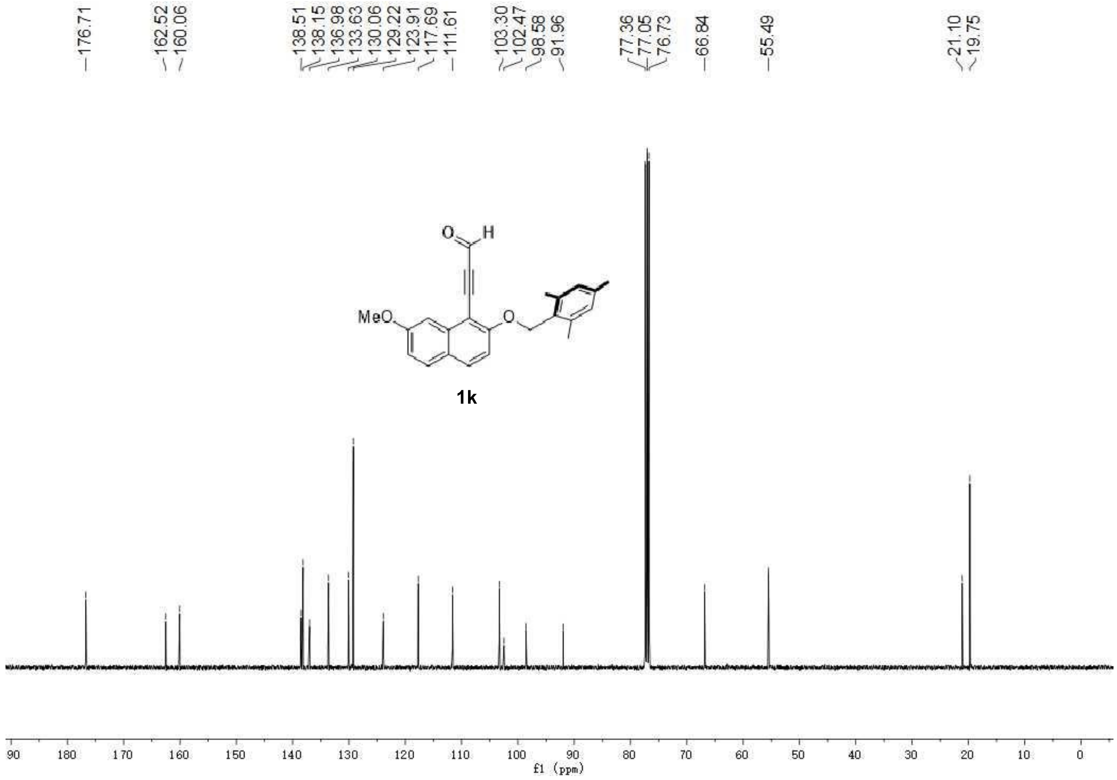

Supplementary Figure 18. ^{13}C NMR Spectra of **1k**

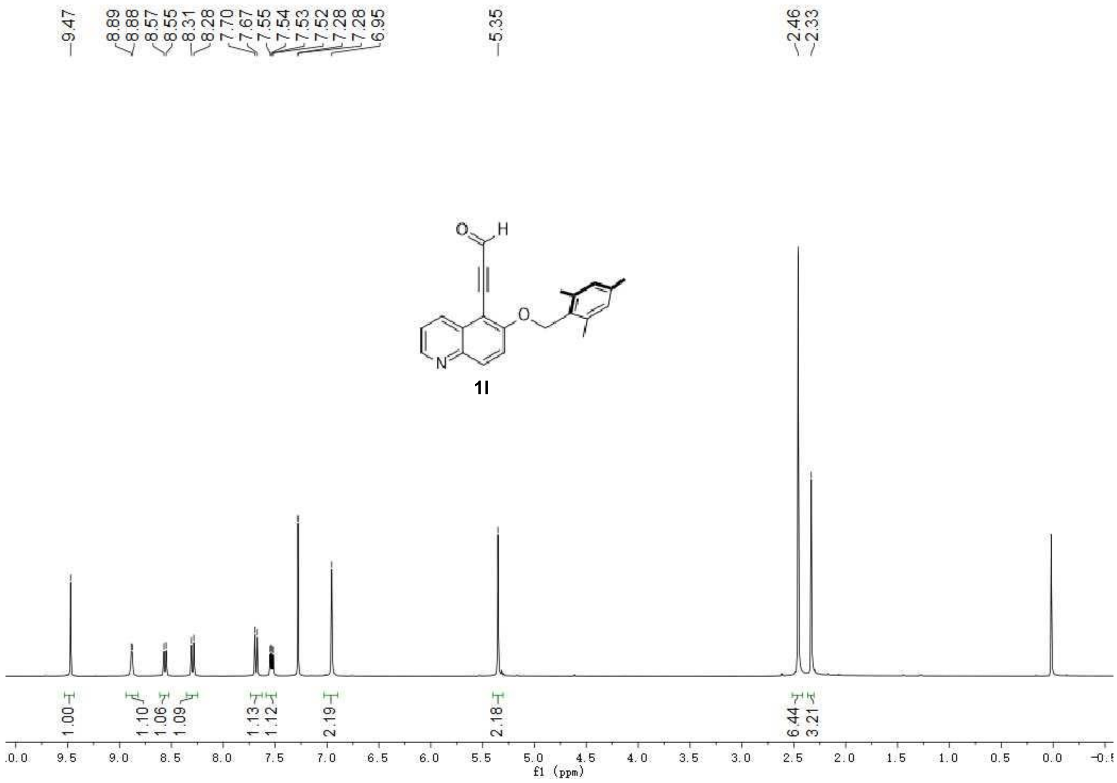

Supplementary Figure 19. ^{1}H NMR Spectra of **1l**

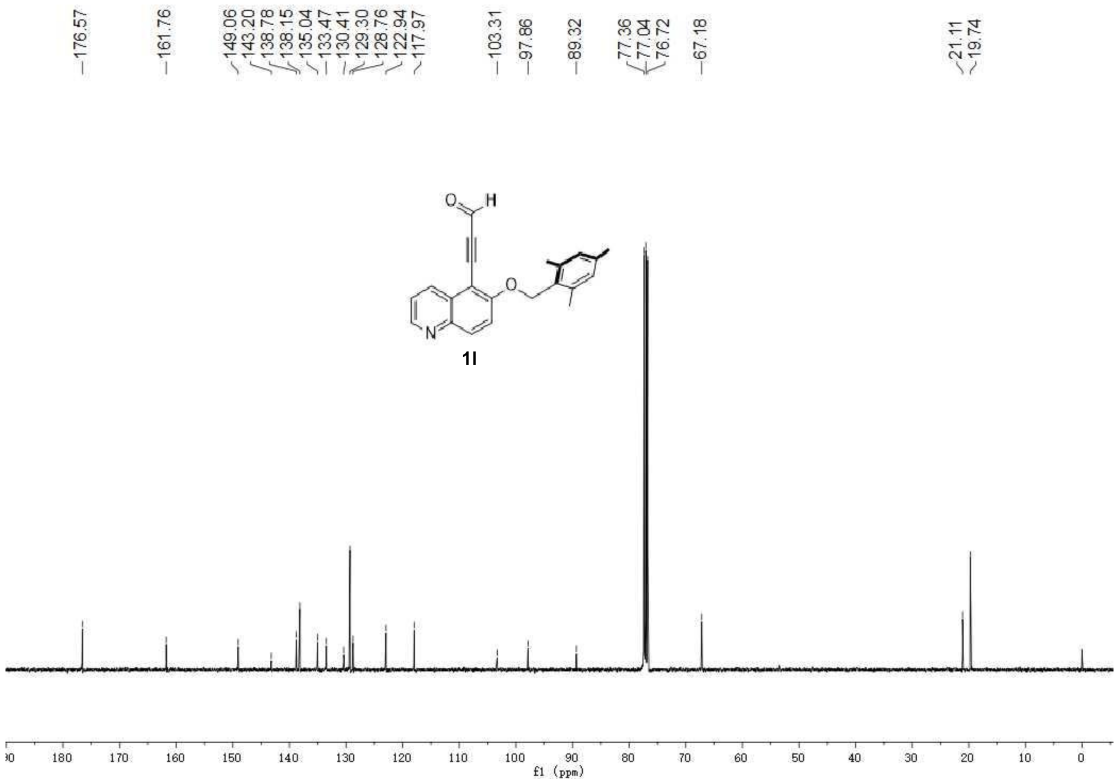

Supplementary Figure 20. ^{13}C NMR Spectra of **1l**

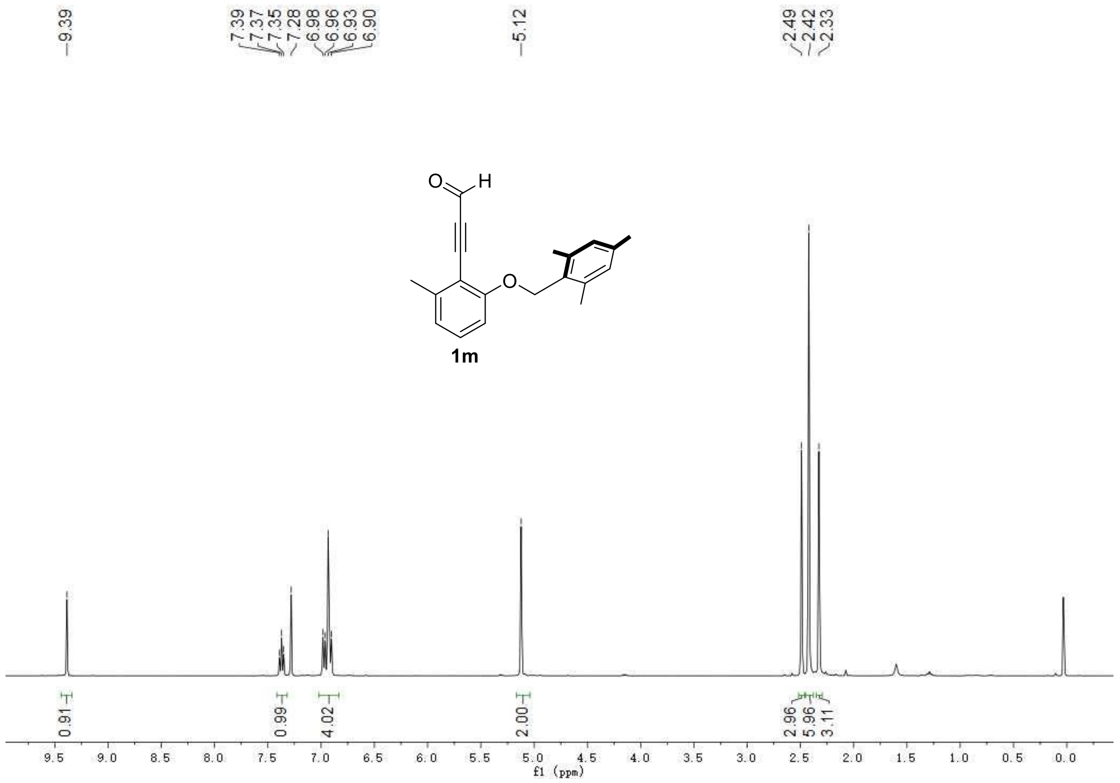

Supplementary Figure 21. ^{1}H NMR Spectra of **1m**

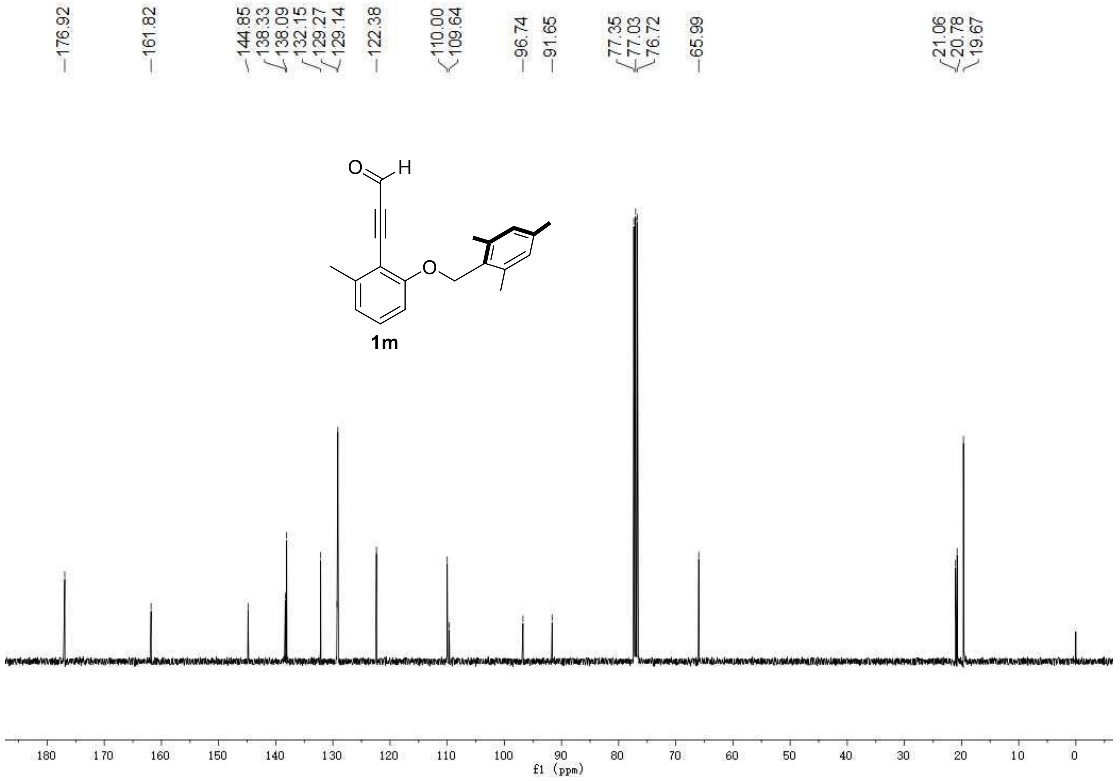

Supplementary Figure 22. ^{13}C NMR Spectra of **1m**

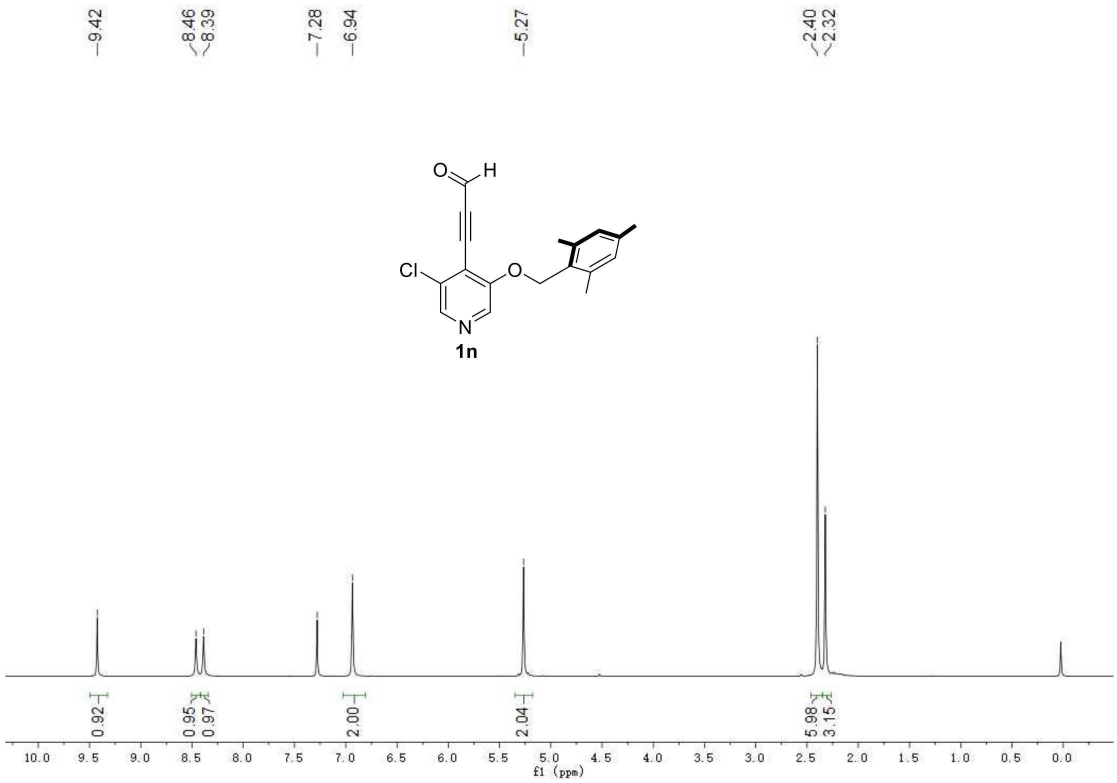

Supplementary Figure 23. ^{1}H NMR Spectra of **1n**

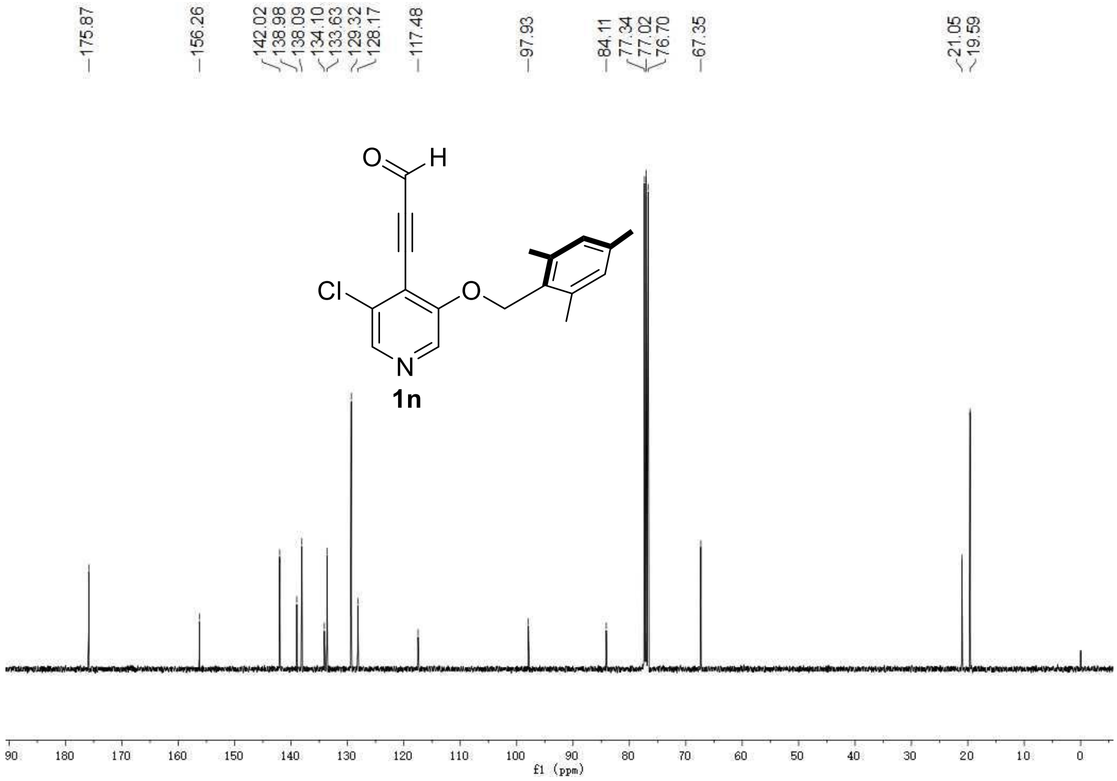

Supplementary Figure 24. ^{13}C NMR Spectra of **1n**

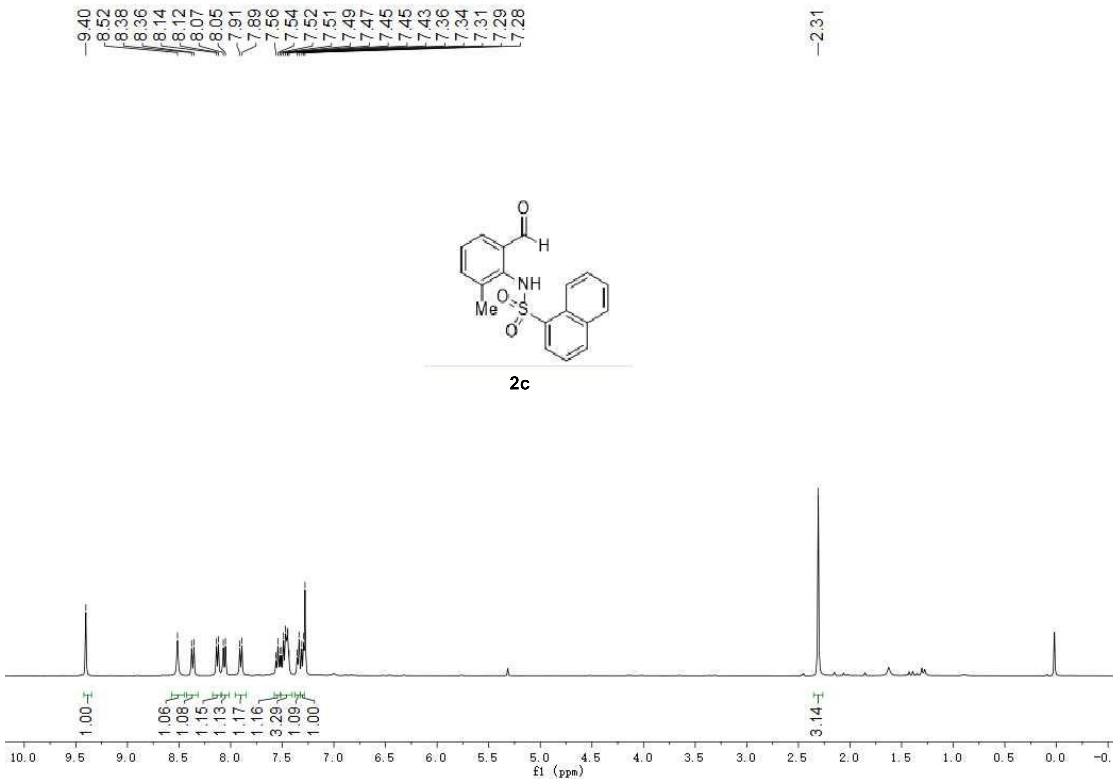

Supplementary Figure 25. ^{1}H NMR Spectra of **2c**

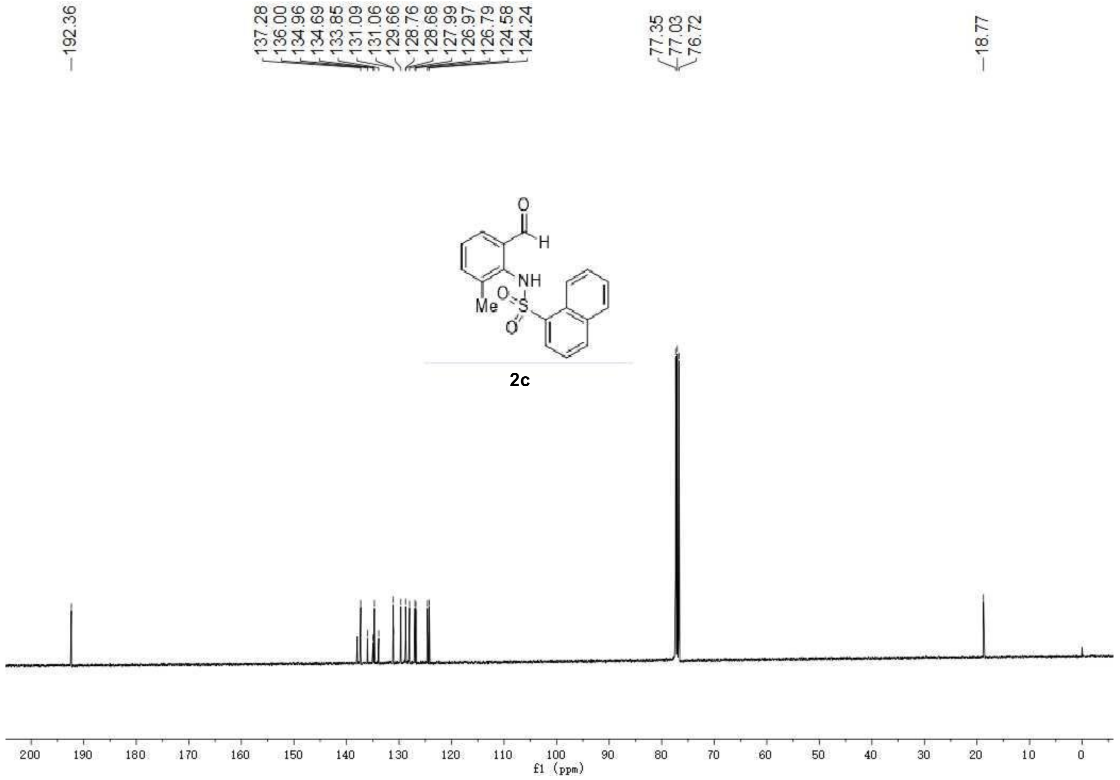

Supplementary Figure 26. ^{13}C NMR Spectra of **2c**

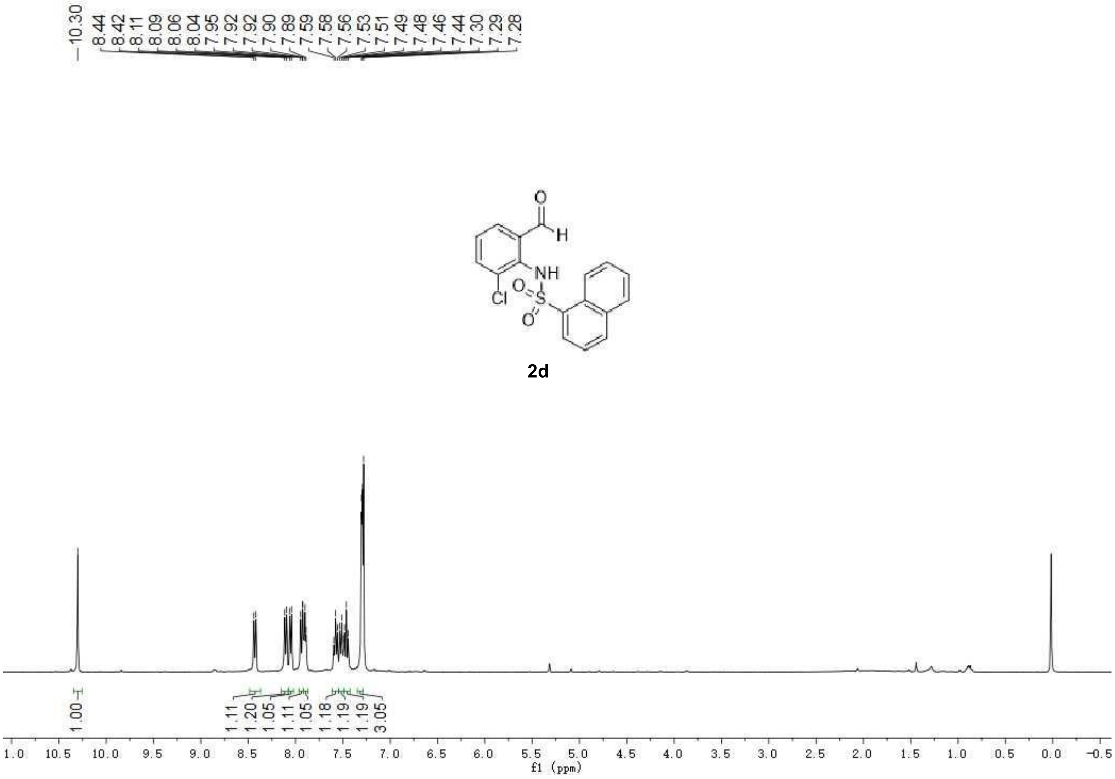

Supplementary Figure 27. ^{1}H NMR Spectra of **2d**

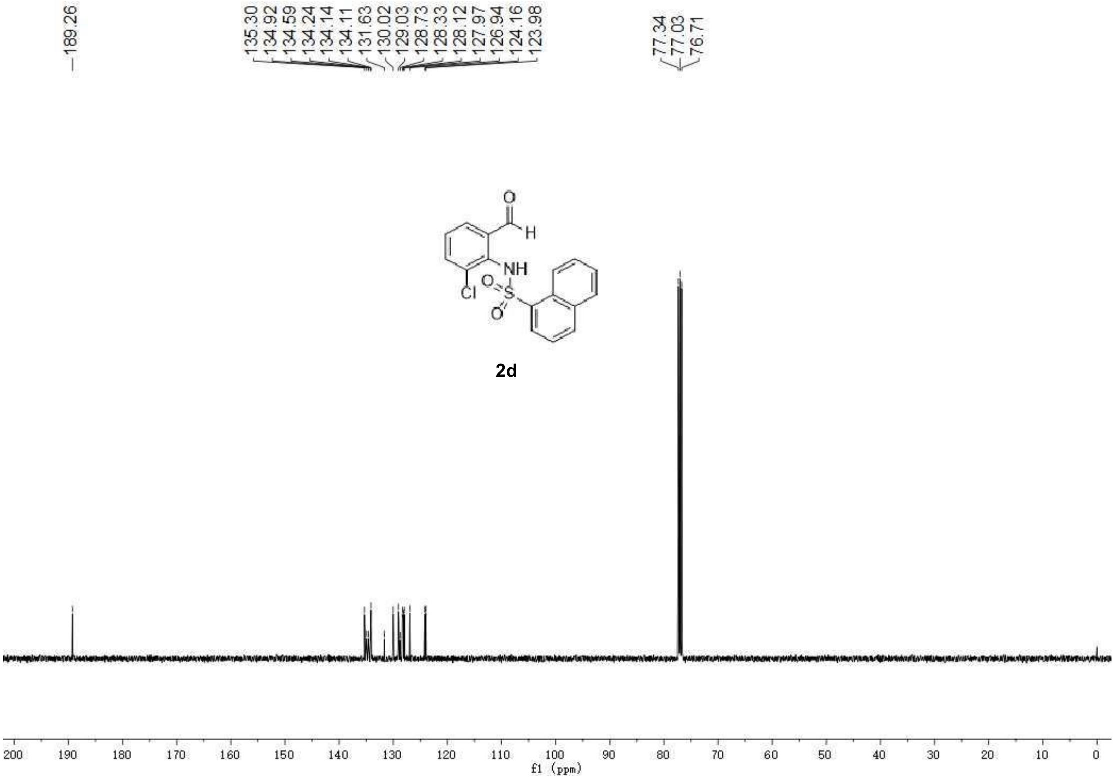

Supplementary Figure 28. ^{13}C NMR Spectra of **2d**

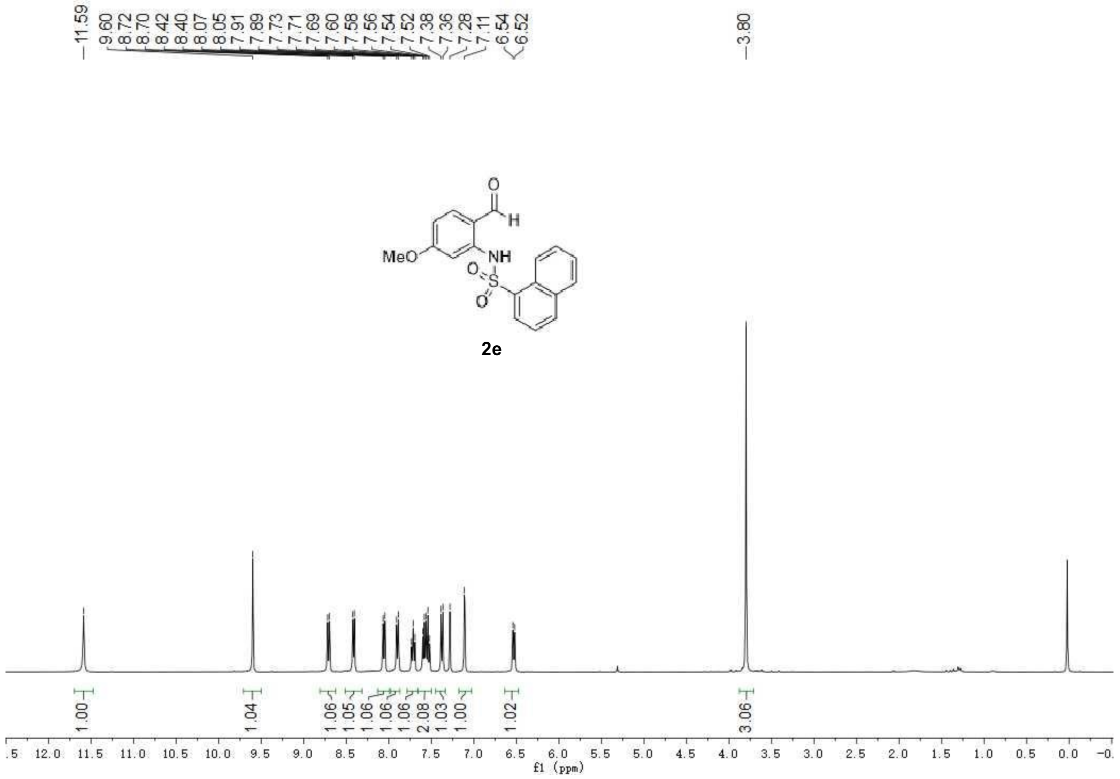

Supplementary Figure 29. ^{1}H NMR Spectra of **2e**

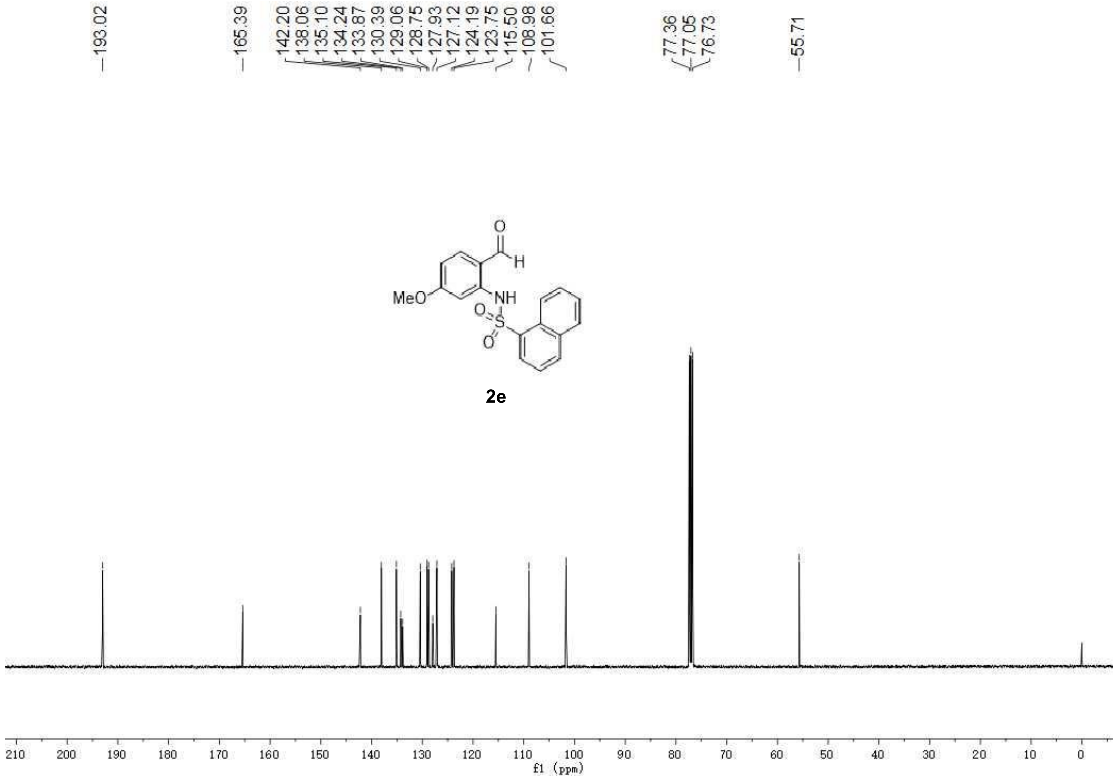

Supplementary Figure 30. ^{13}C NMR Spectra of **2e**

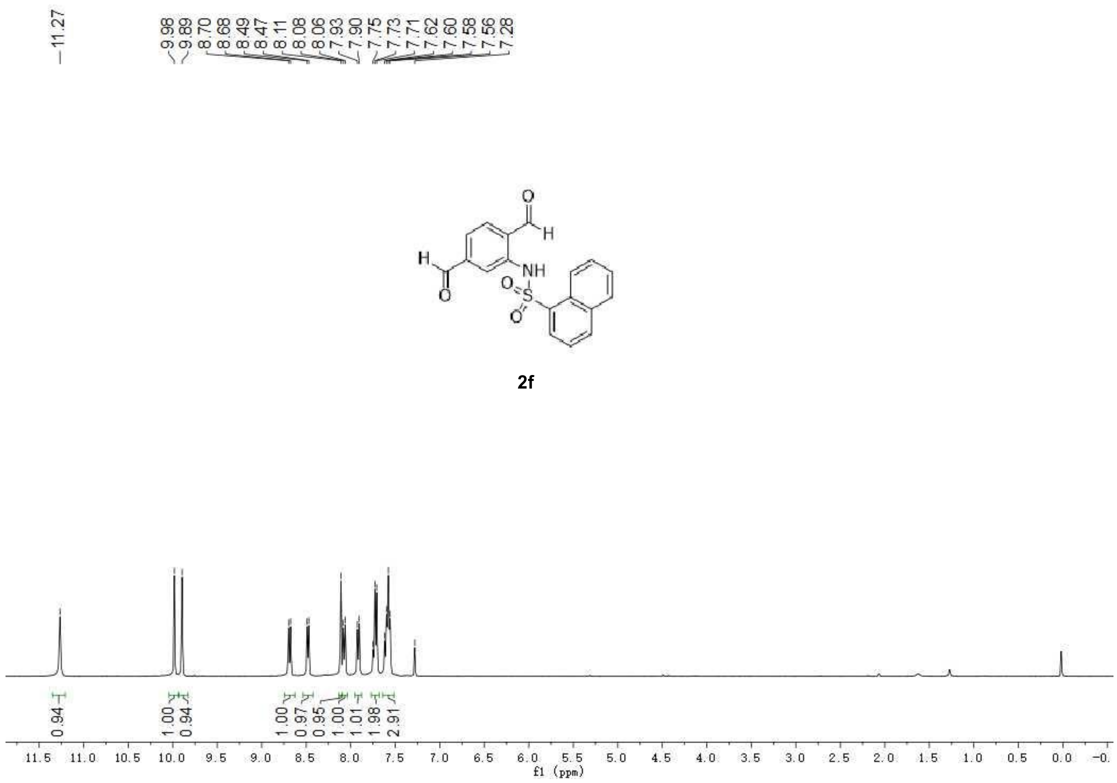

Supplementary Figure 31. ^{1}H NMR Spectra of **2f**

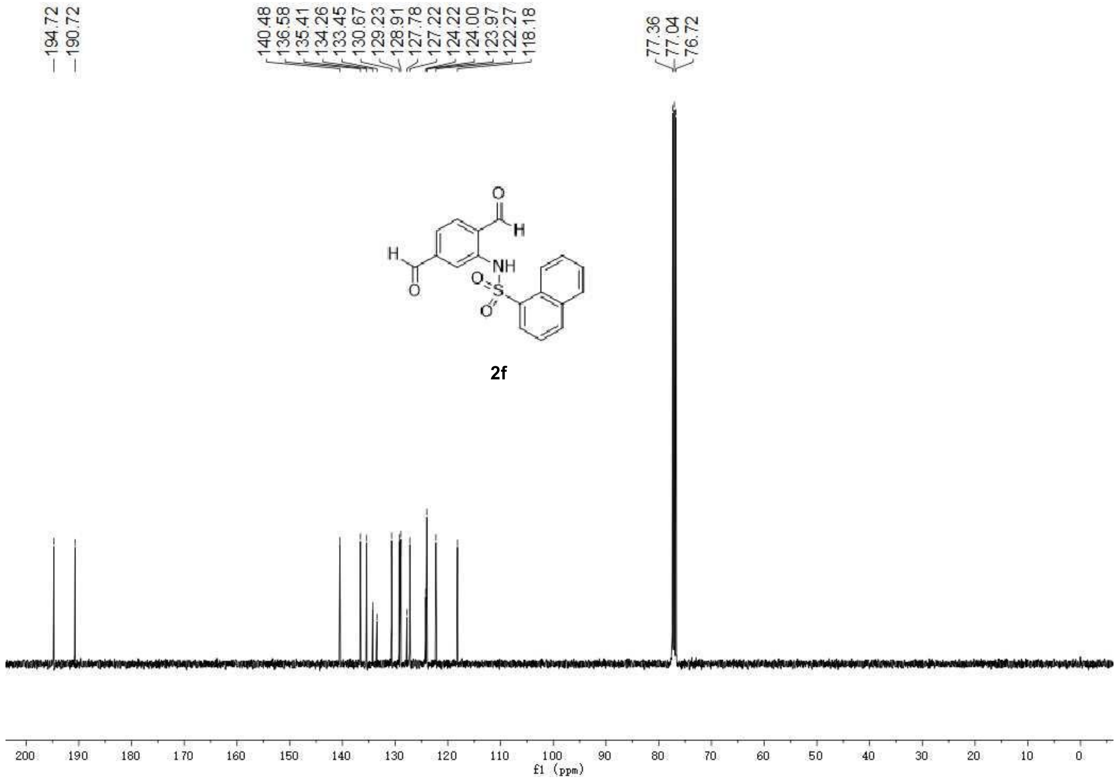

Supplementary Figure 32. ^{13}C NMR Spectra of **2f**

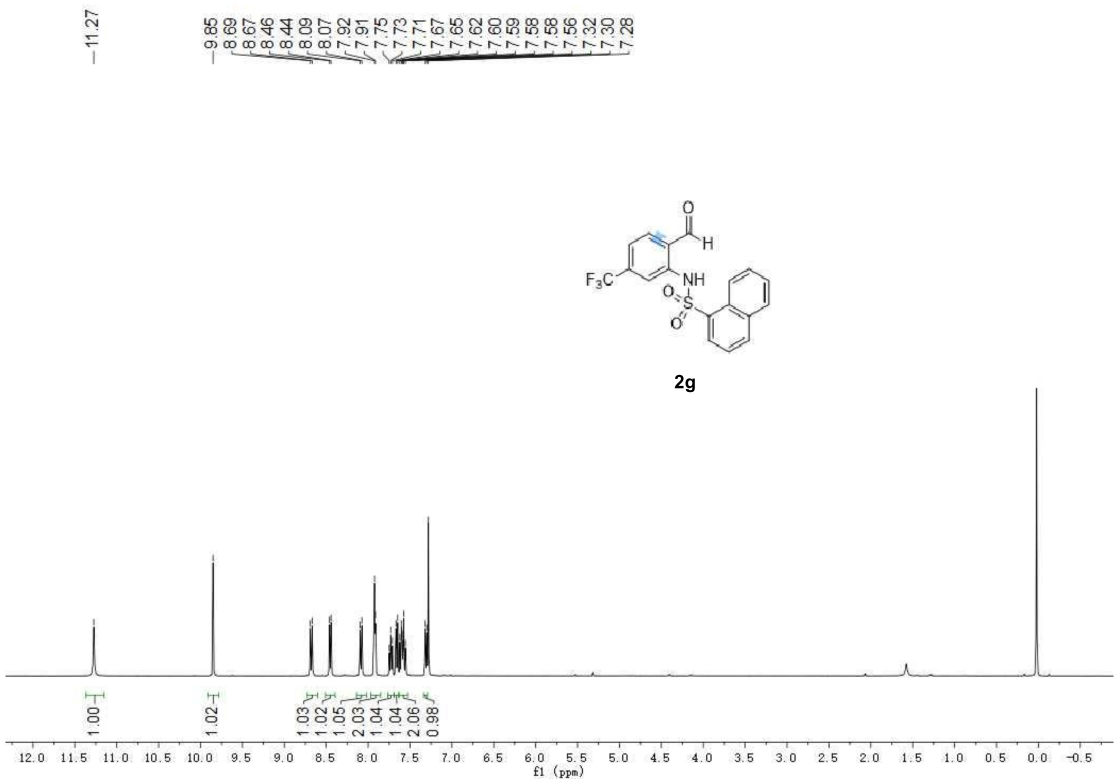

Supplementary Figure 33. ^{1}H NMR Spectra of **2g**

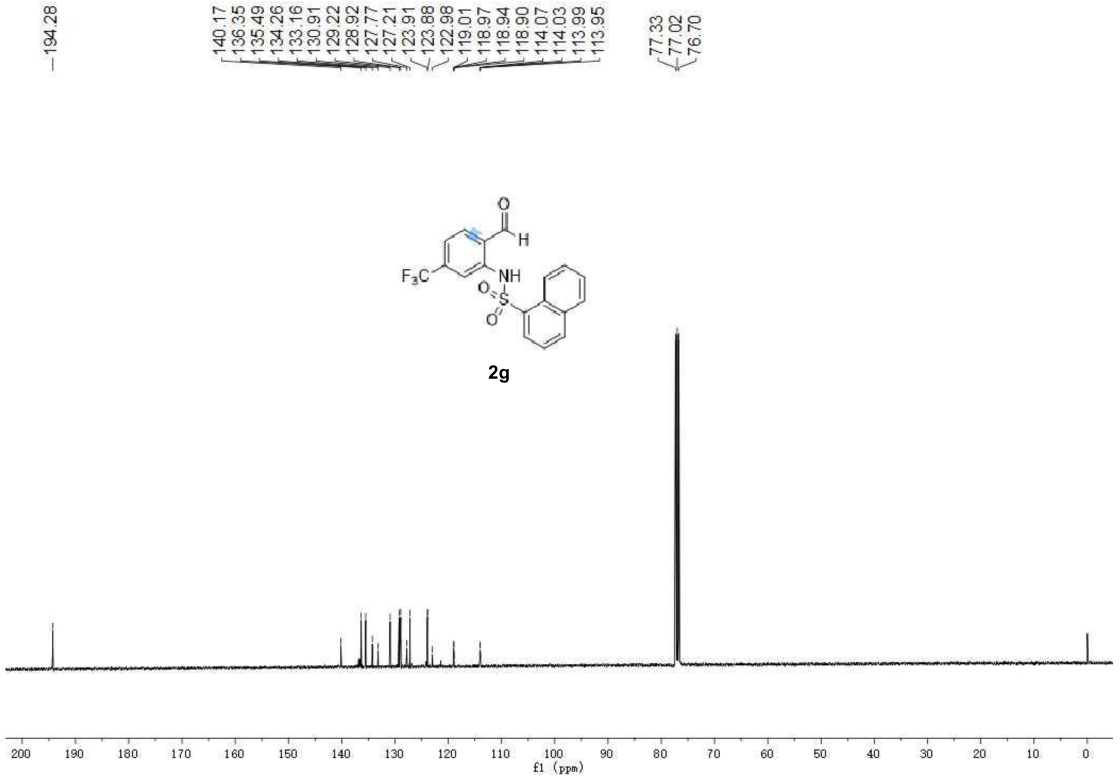

Supplementary Figure 34. ^{13}C NMR Spectra of **2g**

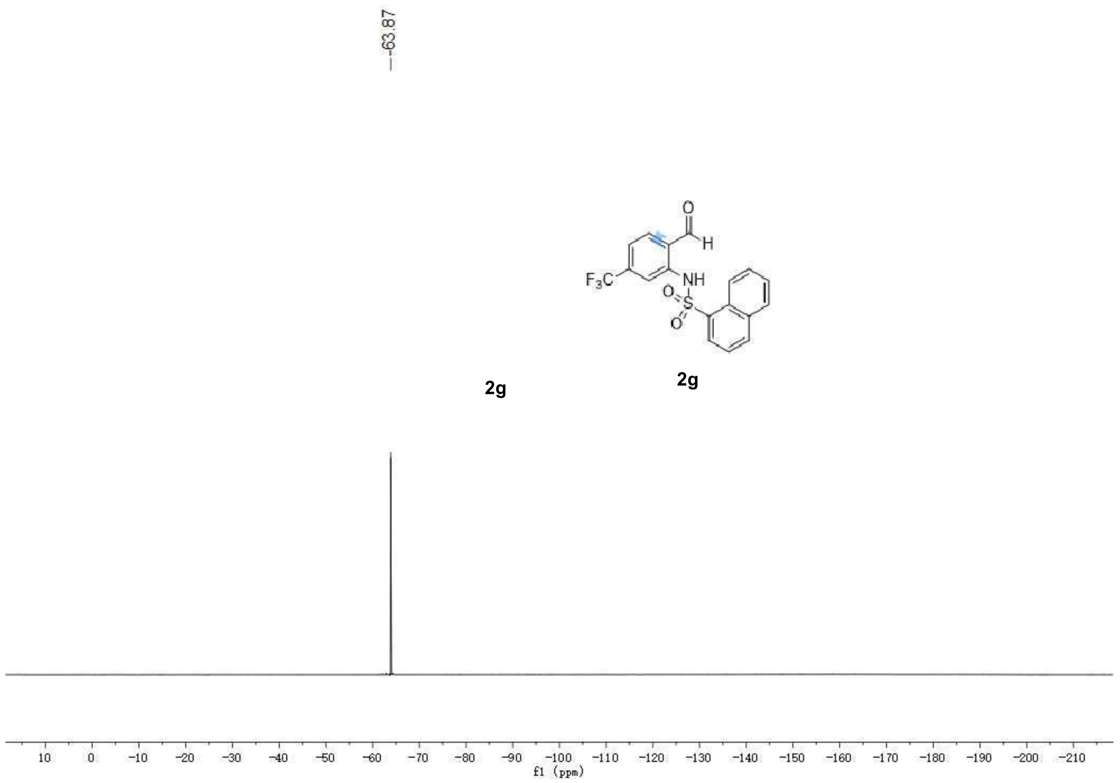

Supplementary Figure 35. ^{19}F NMR Spectra of **2g**

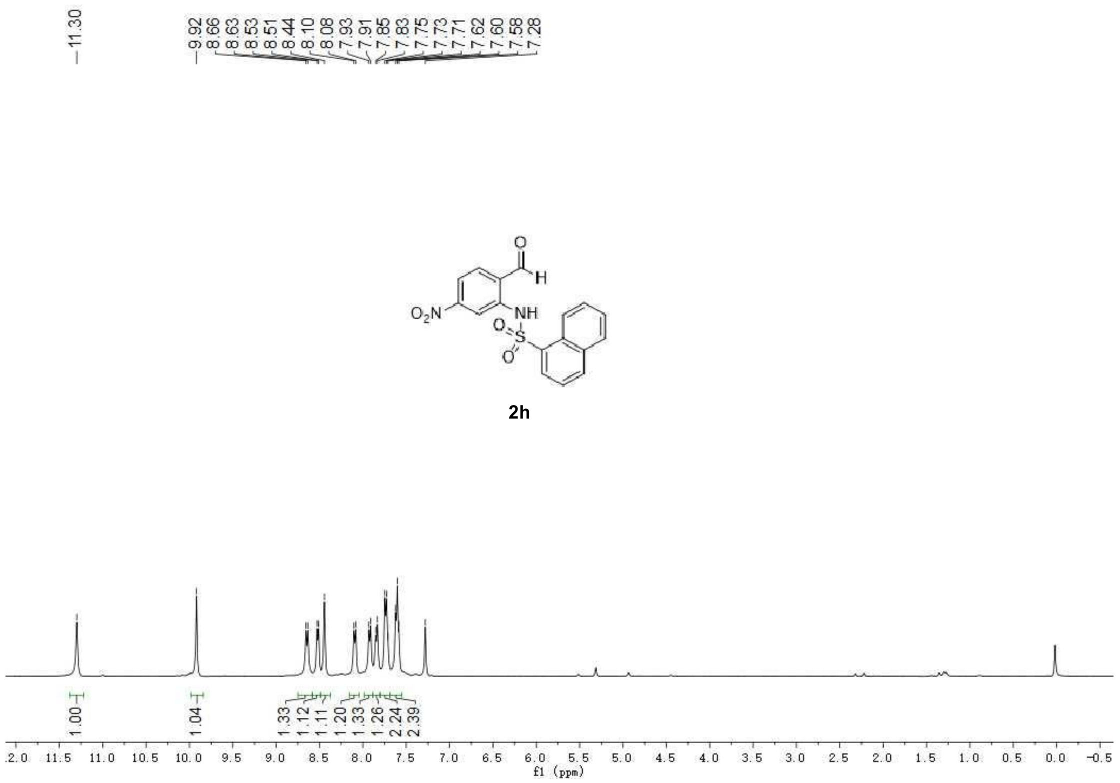

Supplementary Figure 36. ^{1}H NMR Spectra of **2h**

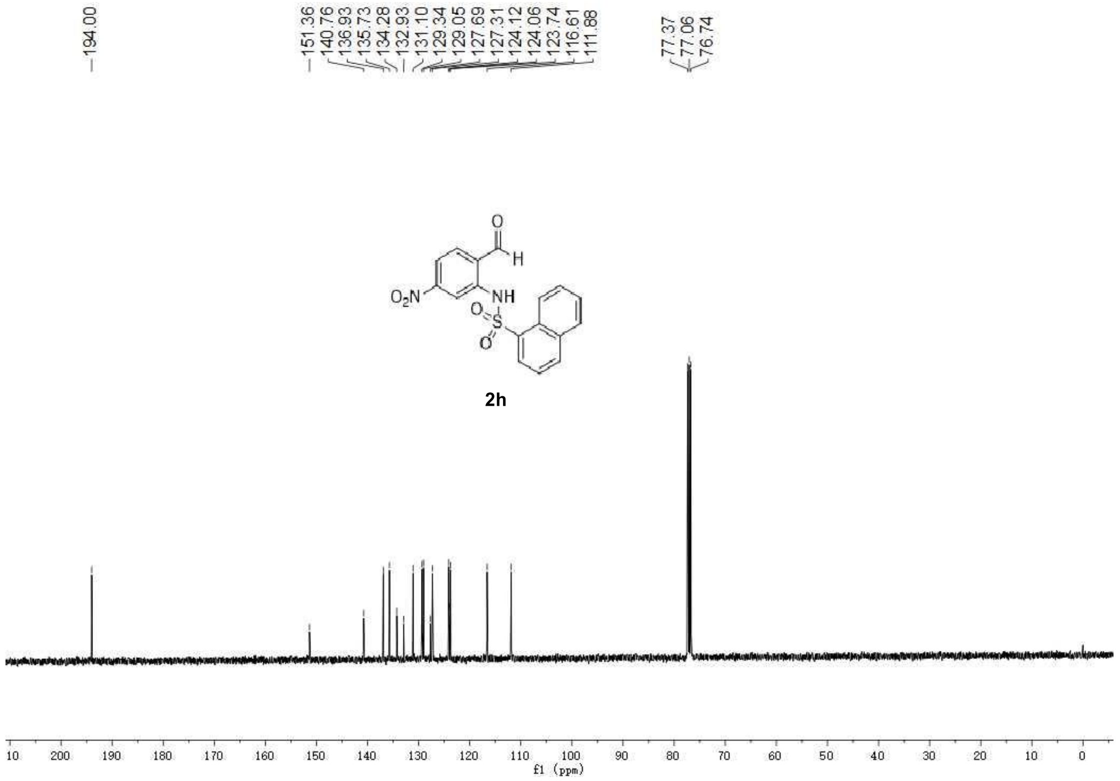

Supplementary Figure 37. ^{13}C NMR Spectra of **2h**

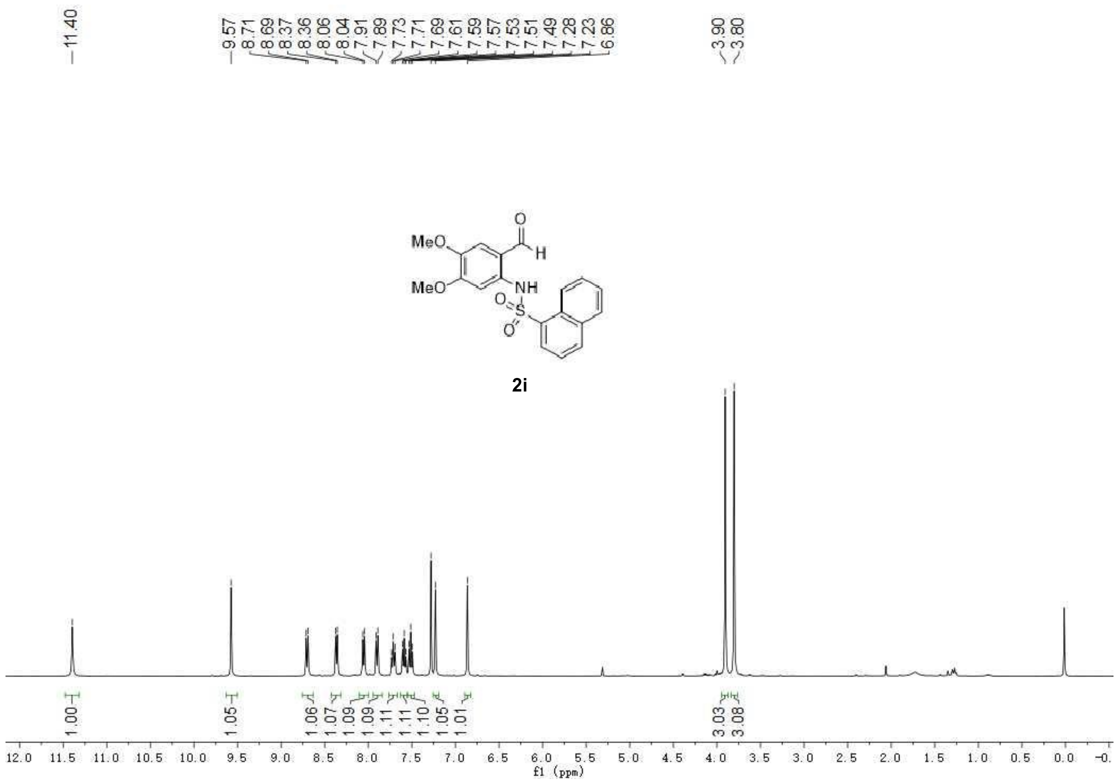

Supplementary Figure 38. ^{1}H NMR Spectra of **2i**

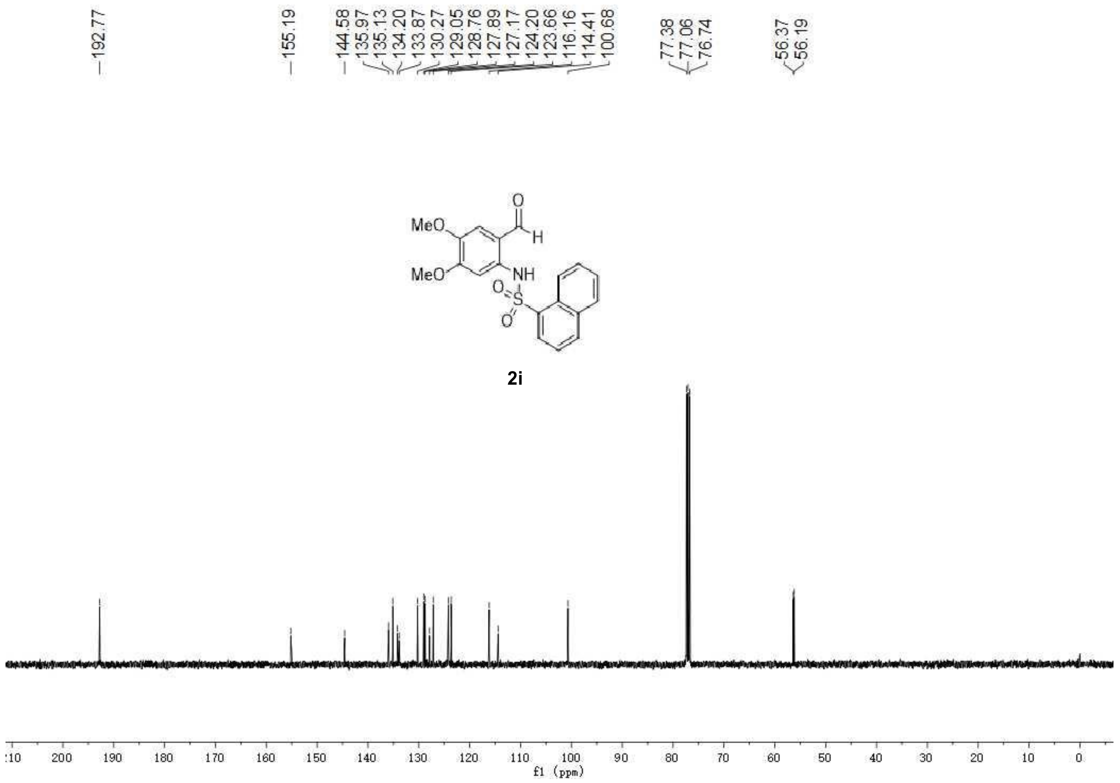

Supplementary Figure 39. ^{13}C NMR Spectra of **2i**

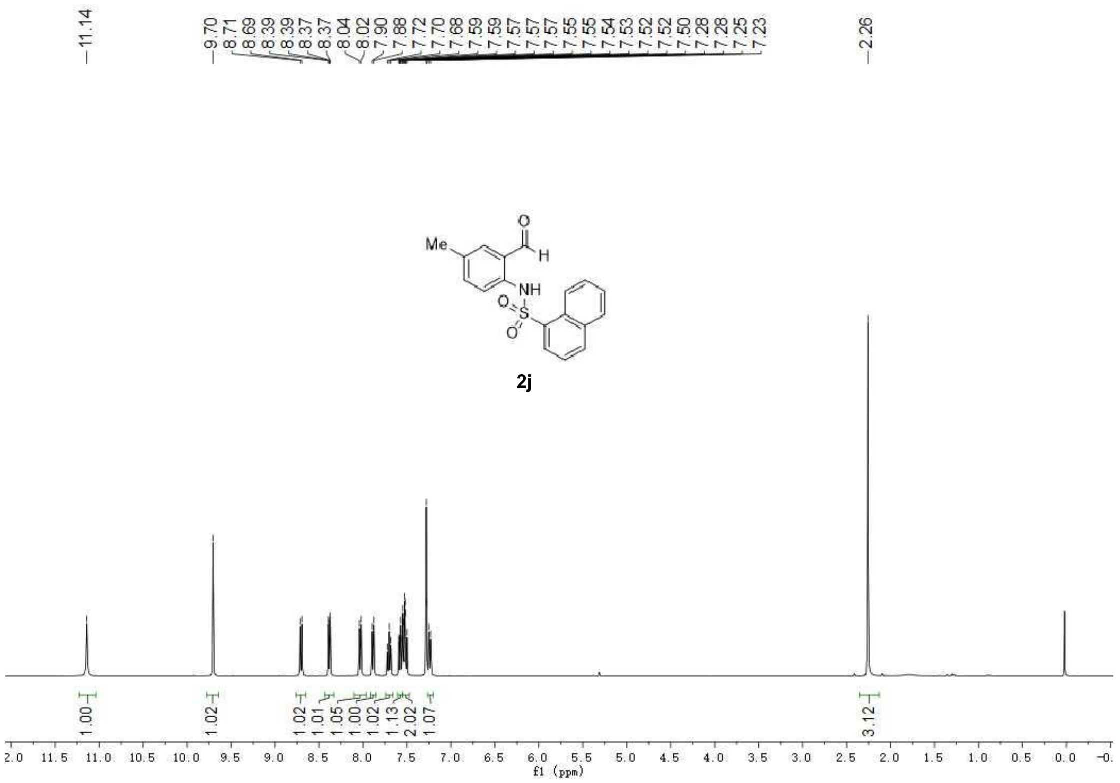

Supplementary Figure 40. ^{1}H NMR Spectra of **2j**

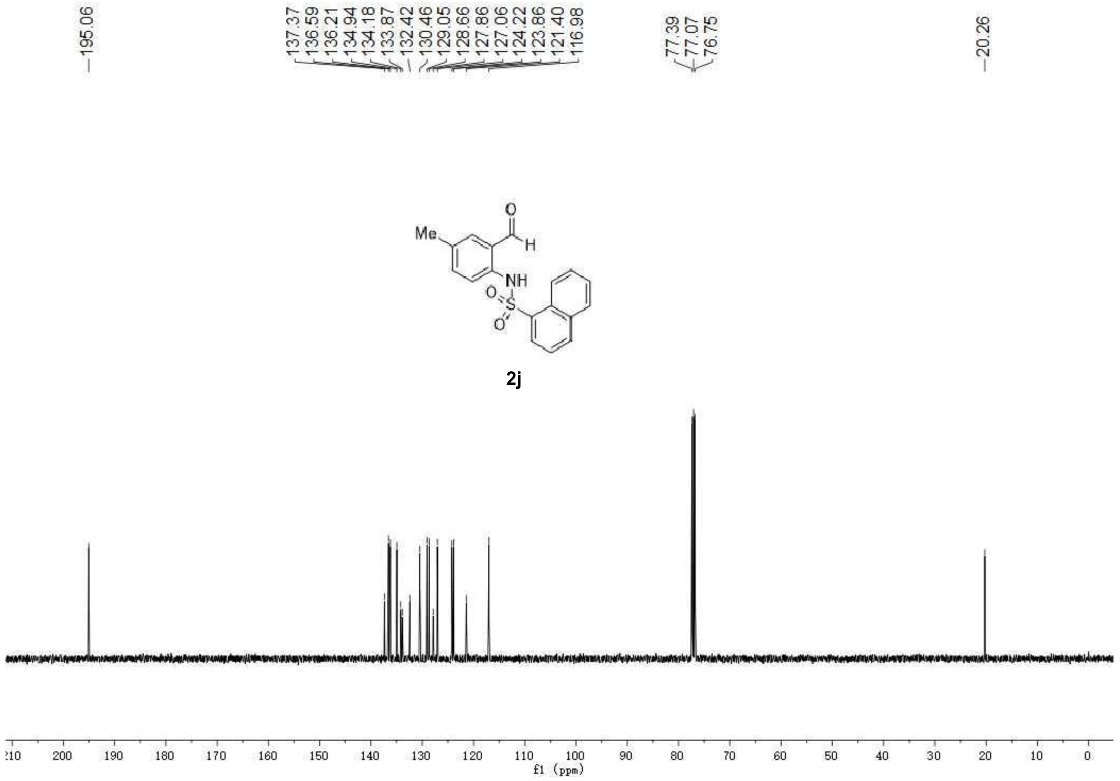

Supplementary Figure 41. ^{13}C NMR Spectra of **2j**

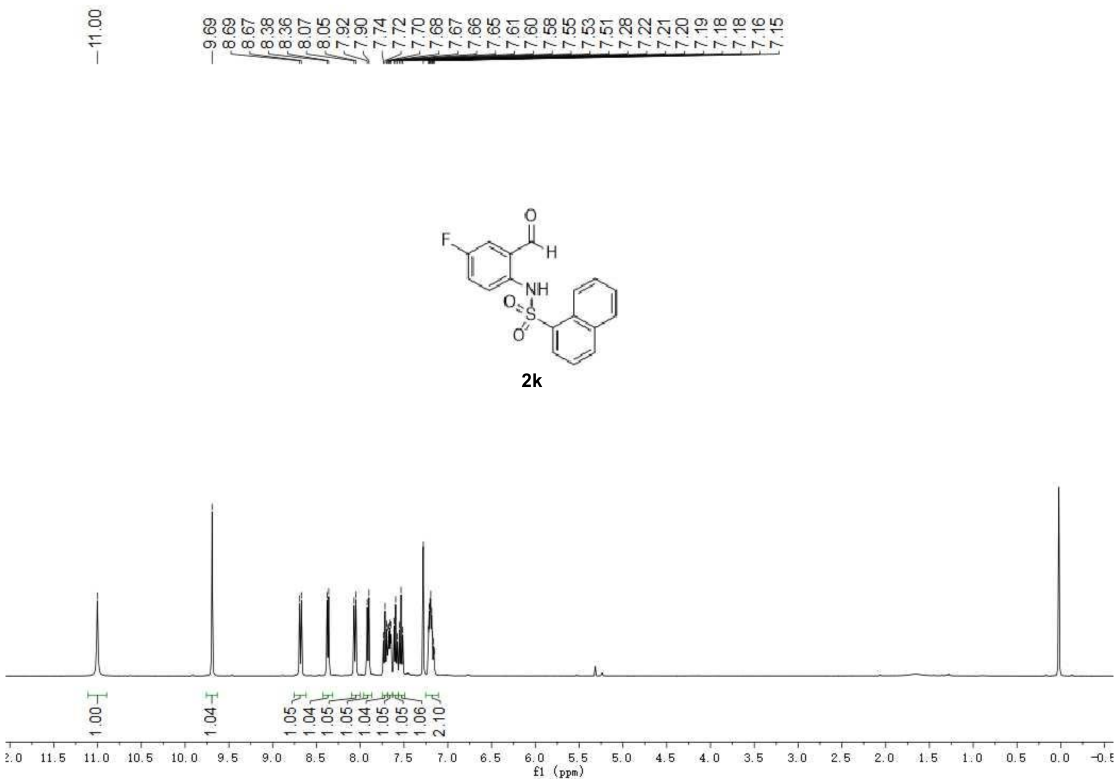

Supplementary Figure 42. ^{1}H NMR Spectra of **2k**

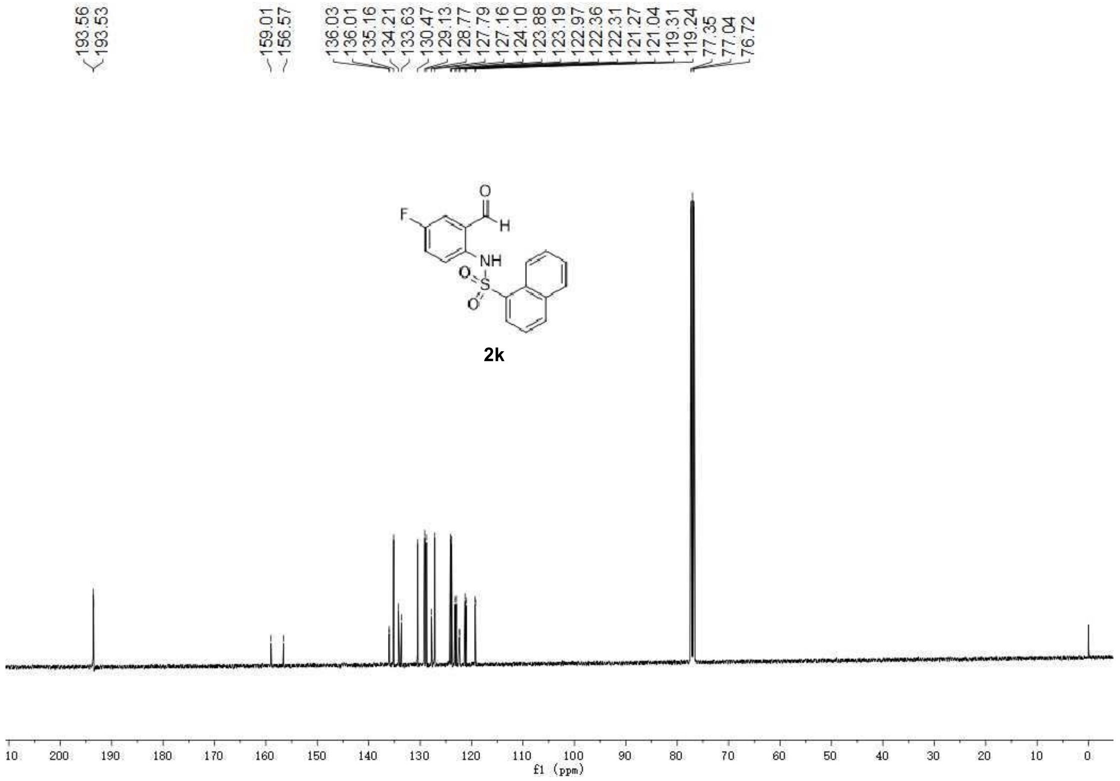

Supplementary Figure 43. ^{13}C NMR Spectra of **2k**

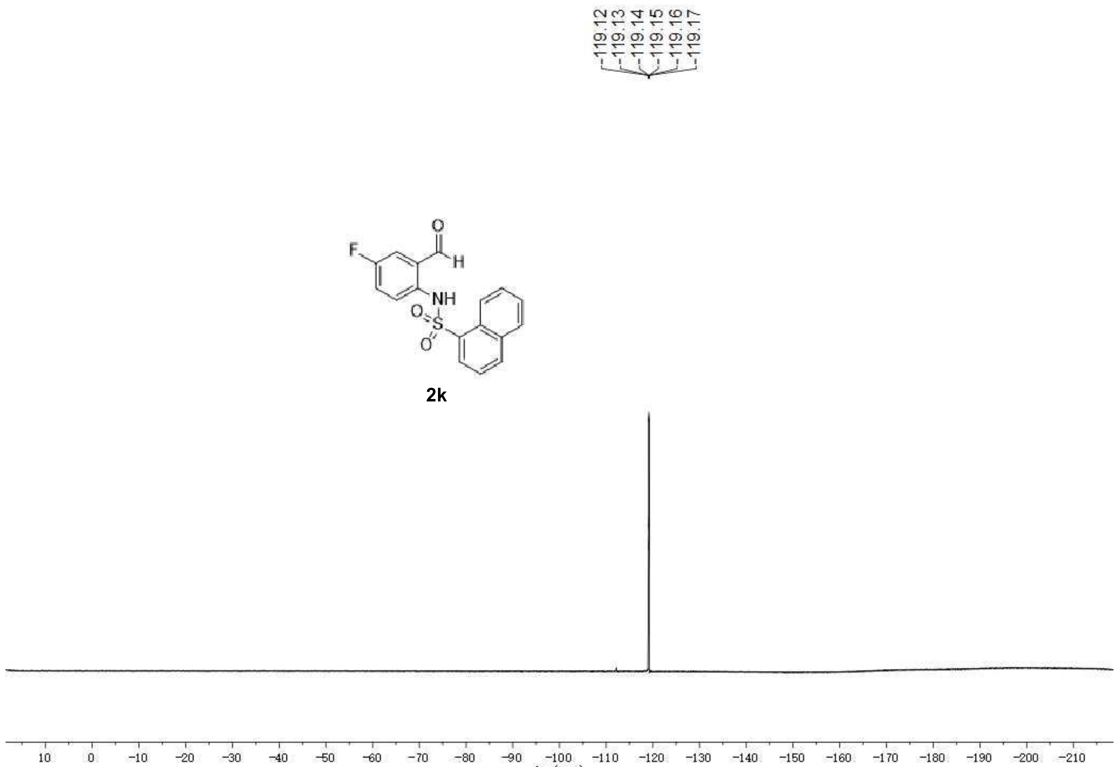

Supplementary Figure 44. ^{19}F NMR Spectra of **2k**

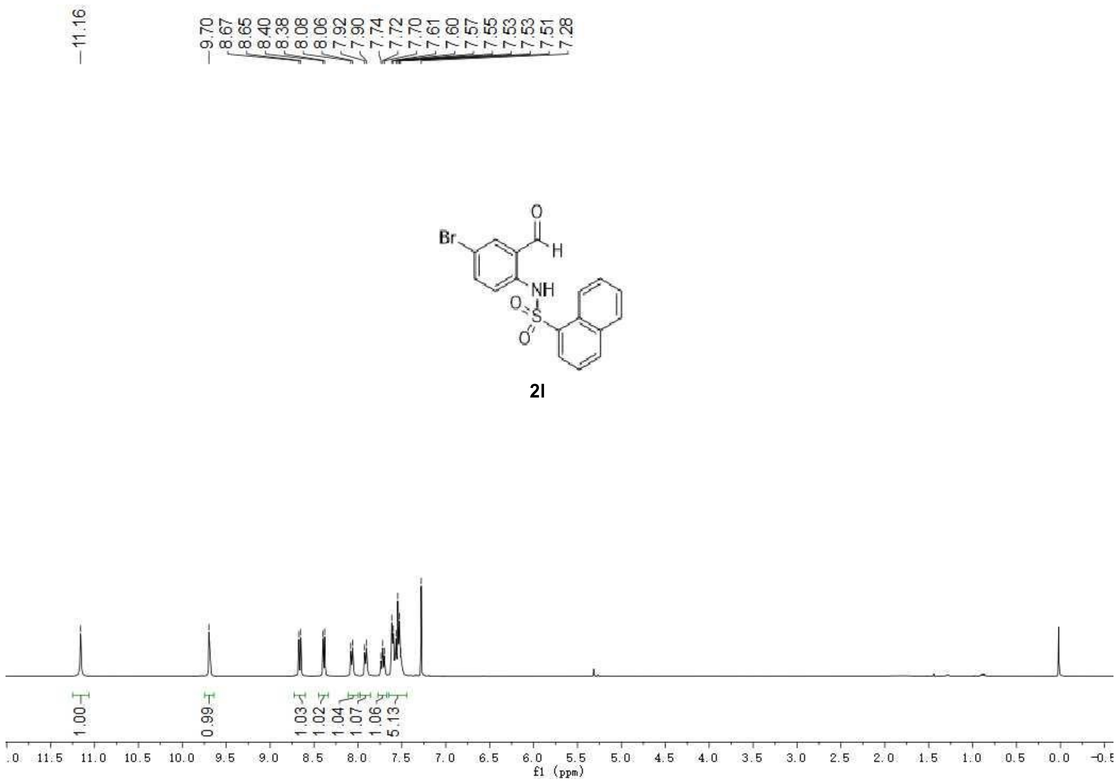

Supplementary Figure 45. ^{1}H NMR Spectra of **2l**

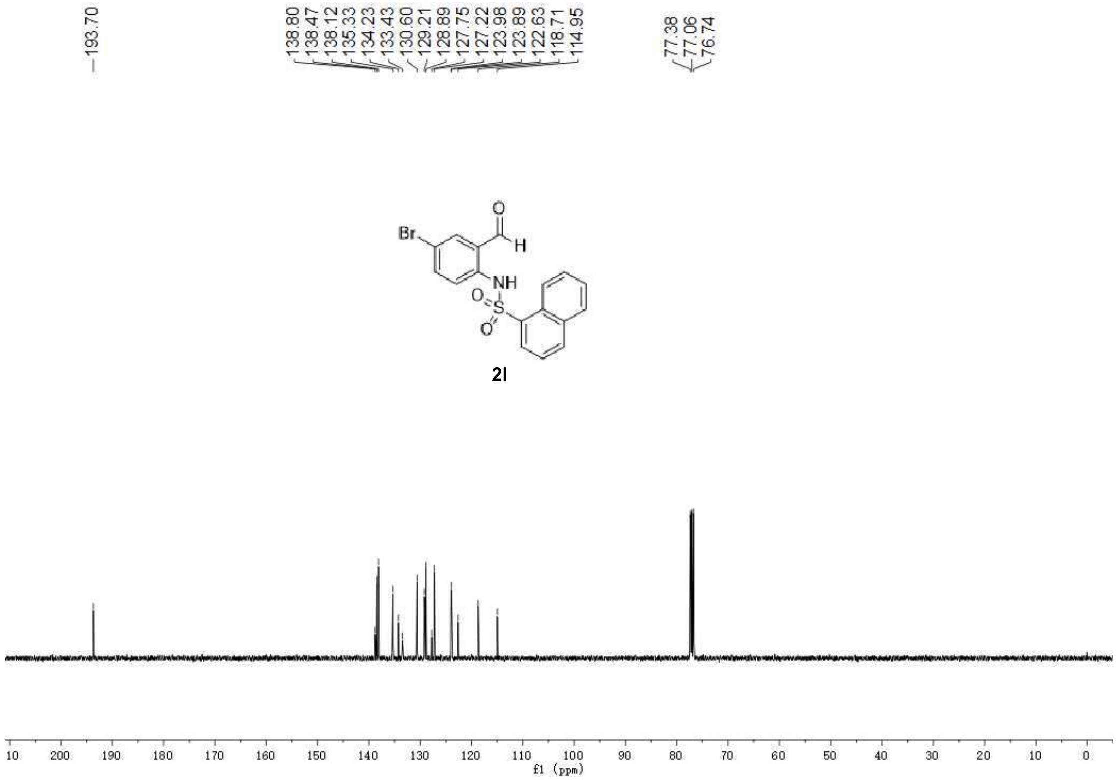

Supplementary Figure 46. ^{13}C NMR Spectra of **2l**

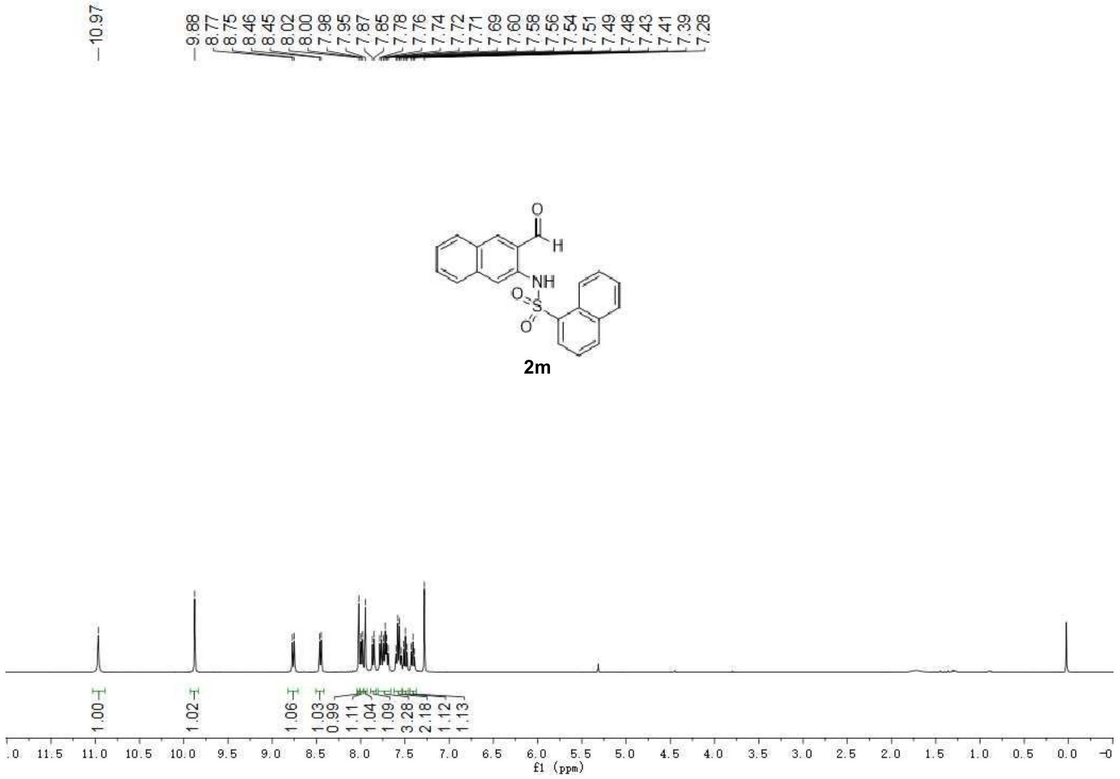

Supplementary Figure 47. ^{1}H NMR Spectra of **2m**

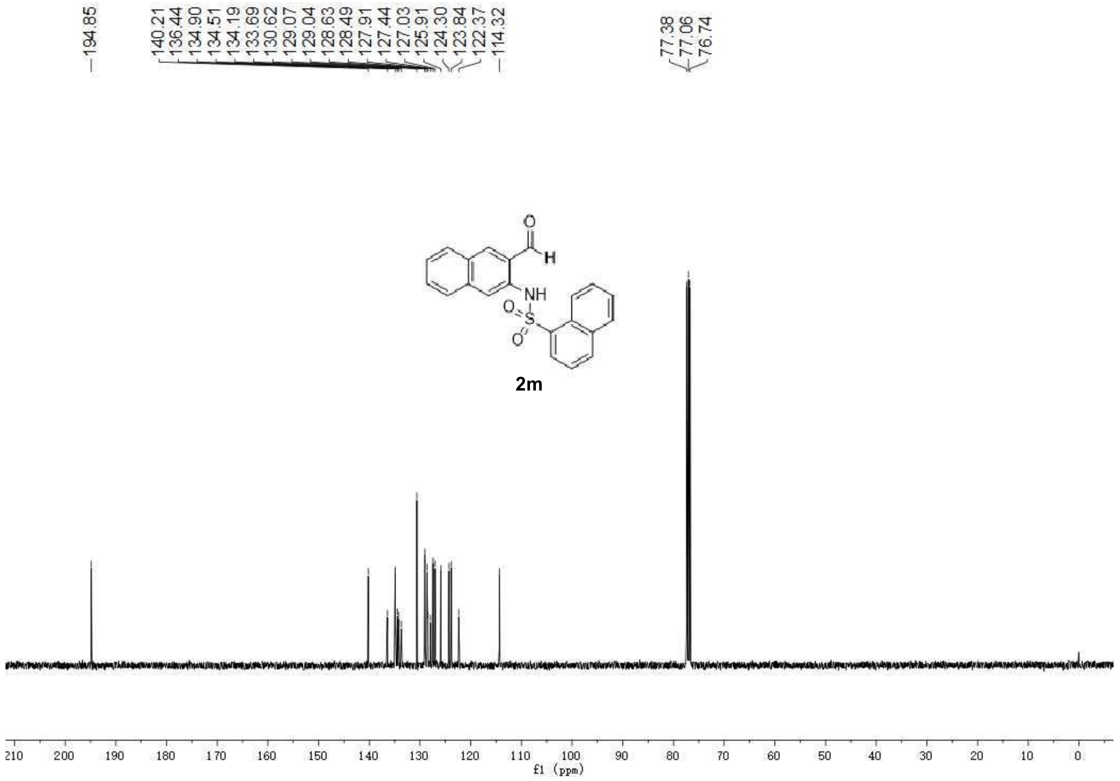

Supplementary Figure 48. ^{13}C NMR Spectra of **2m**

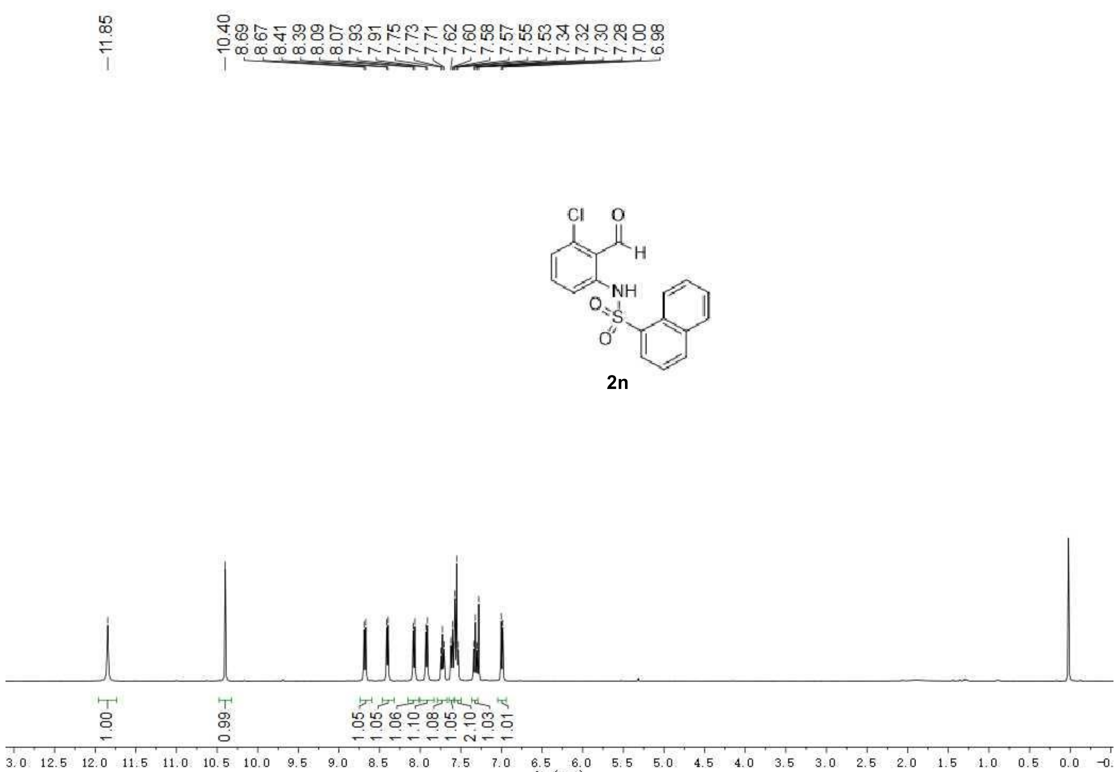

Supplementary Figure 49. ^{1}H NMR Spectra of **2n**

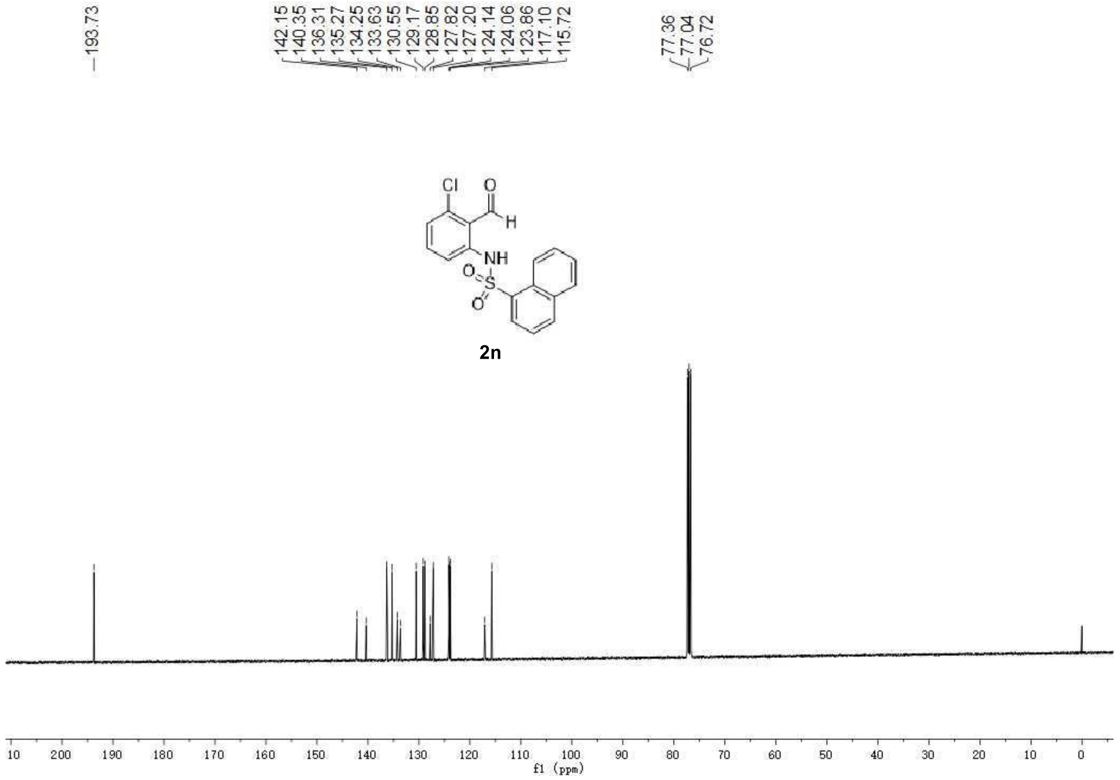

Supplementary Figure 50. ^{13}C NMR Spectra of **2n**

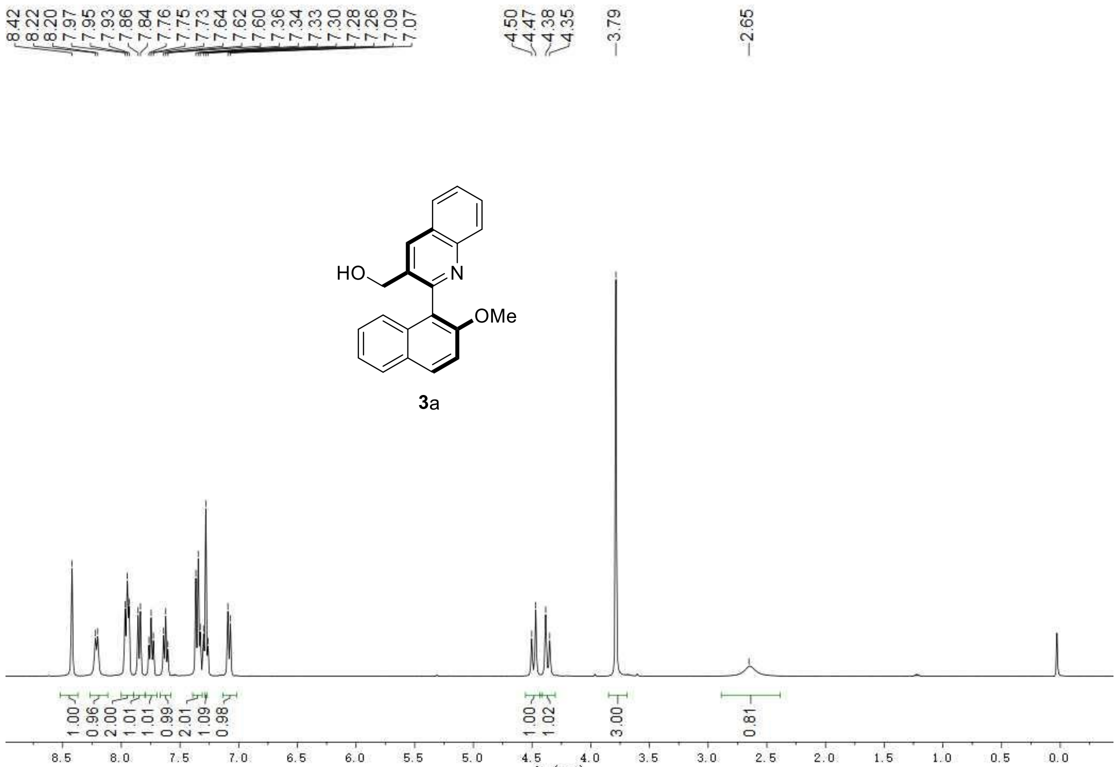

Supplementary Figure 51. ^{1}H NMR Spectra of **3a**

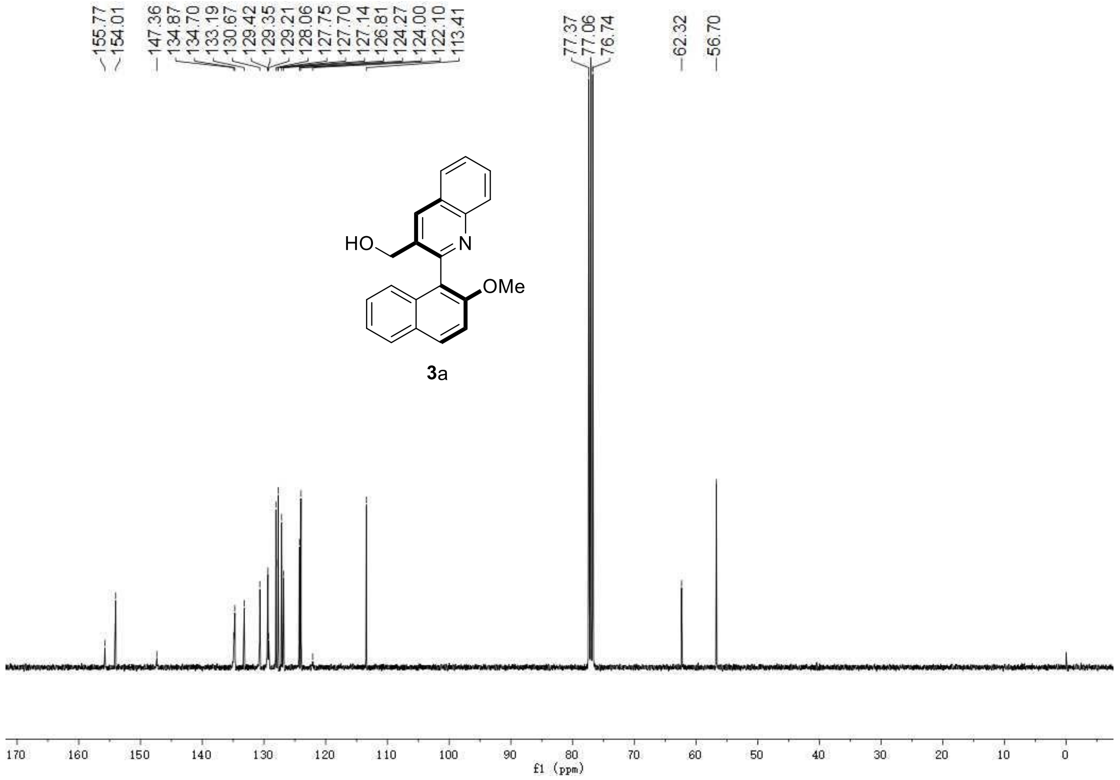

Supplementary Figure 52. ^{13}C NMR Spectra of **3a**

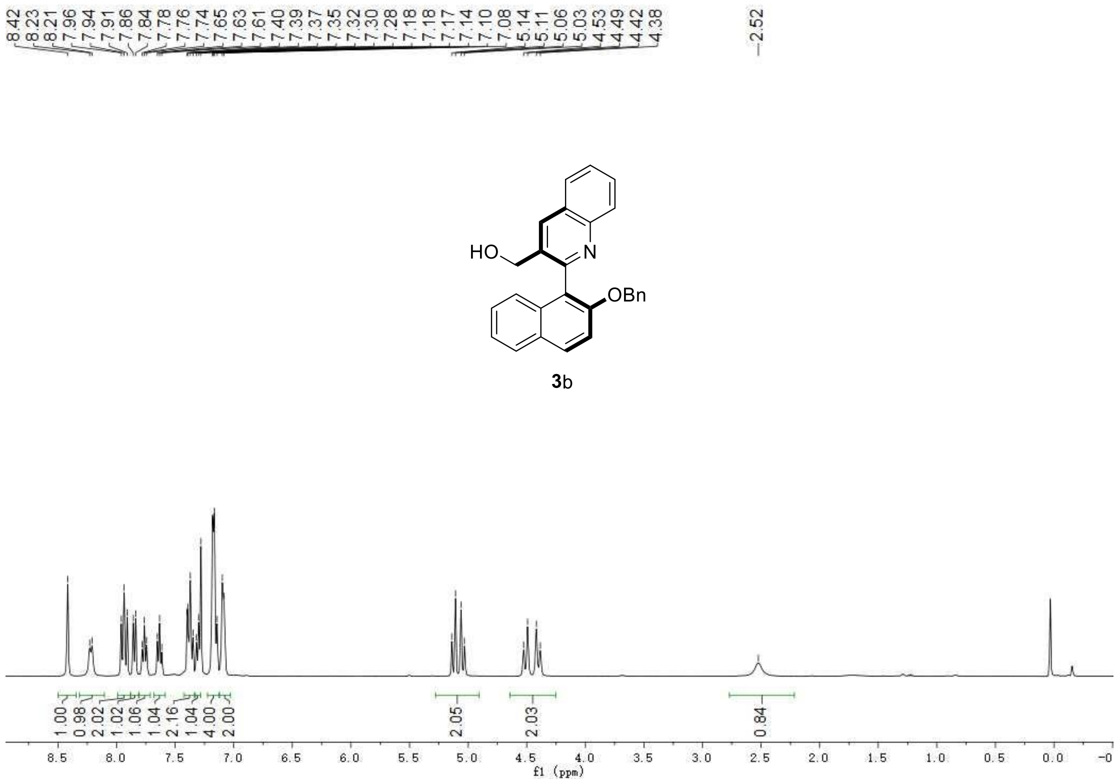

Supplementary Figure 53. ^{1}H NMR Spectra of **3b**

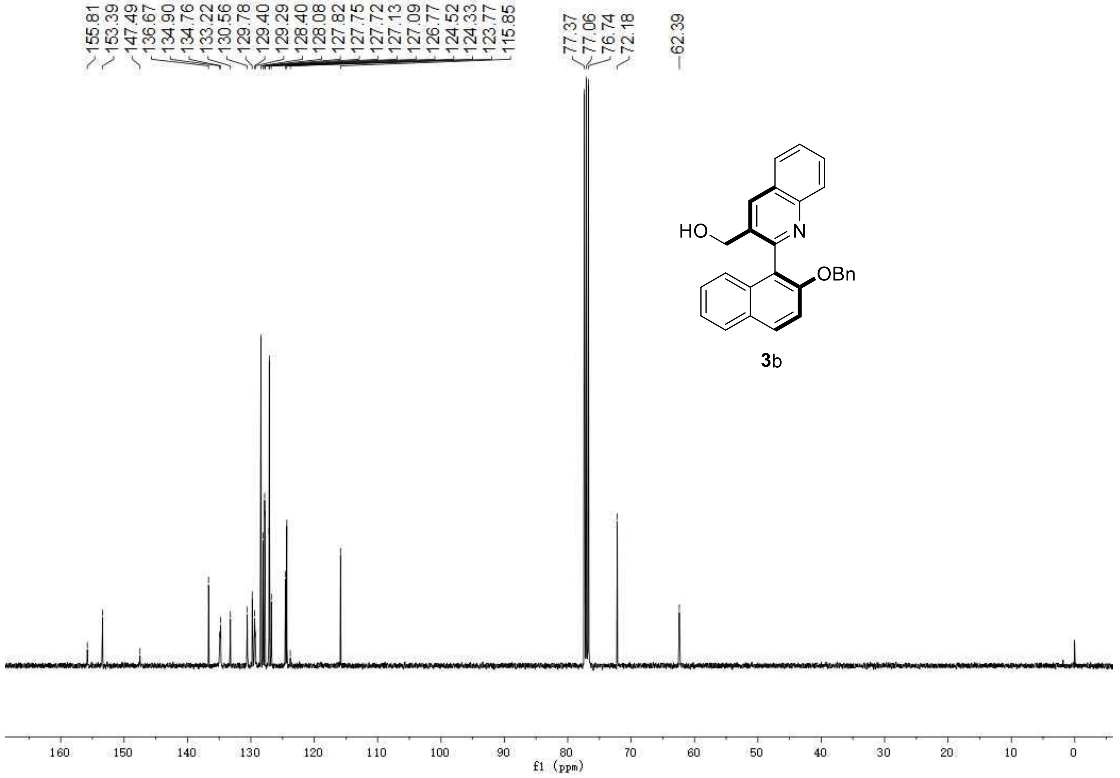

Supplementary Figure 54. ^{13}C NMR Spectra of **3b**

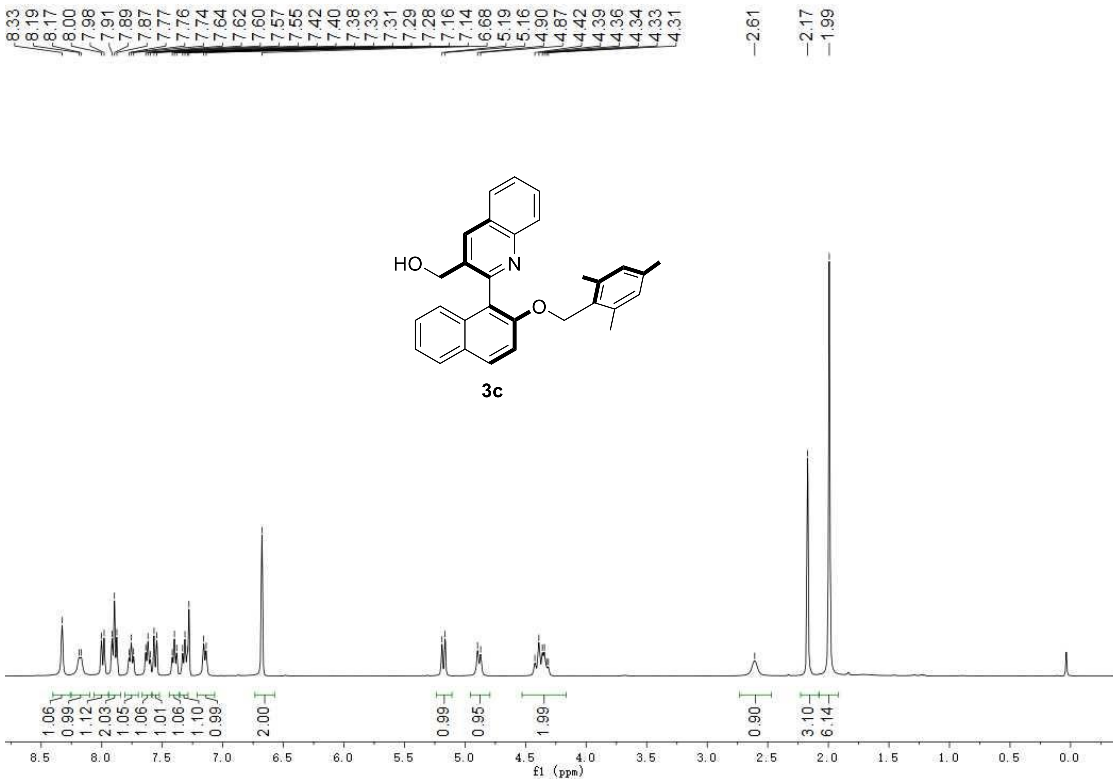

Supplementary Figure 55. ^{1}H NMR Spectra of **3c**

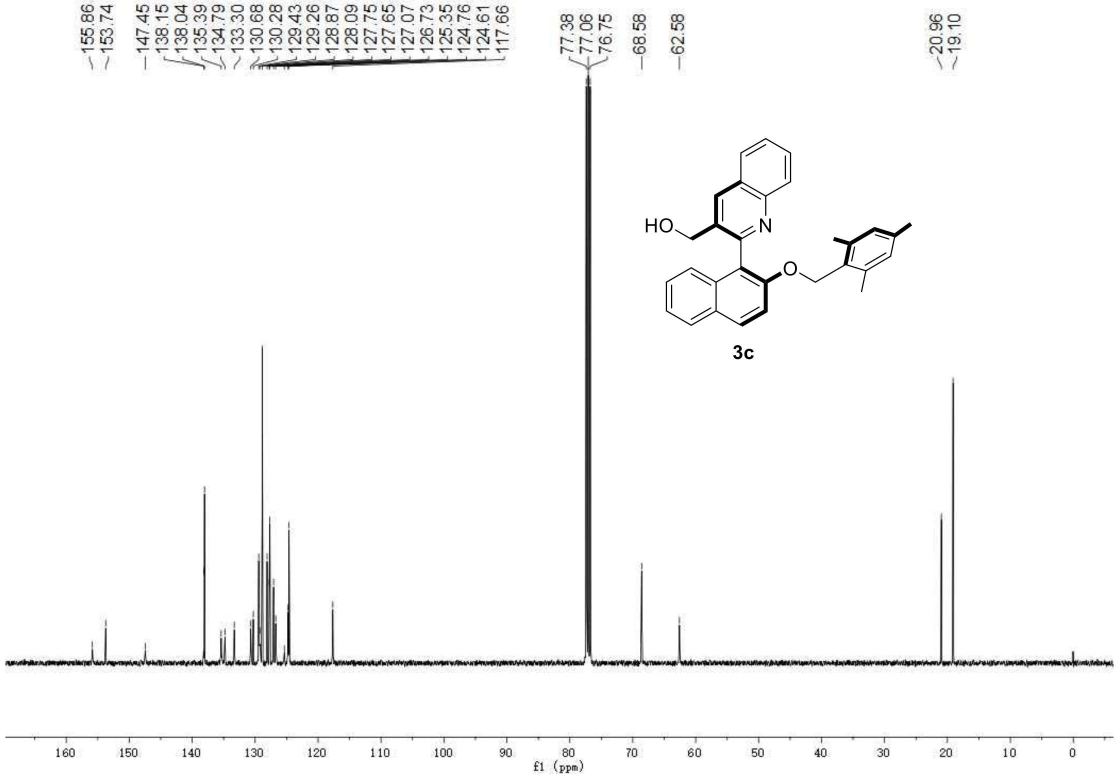

Supplementary Figure 56. ^{13}C NMR Spectra of **3c**

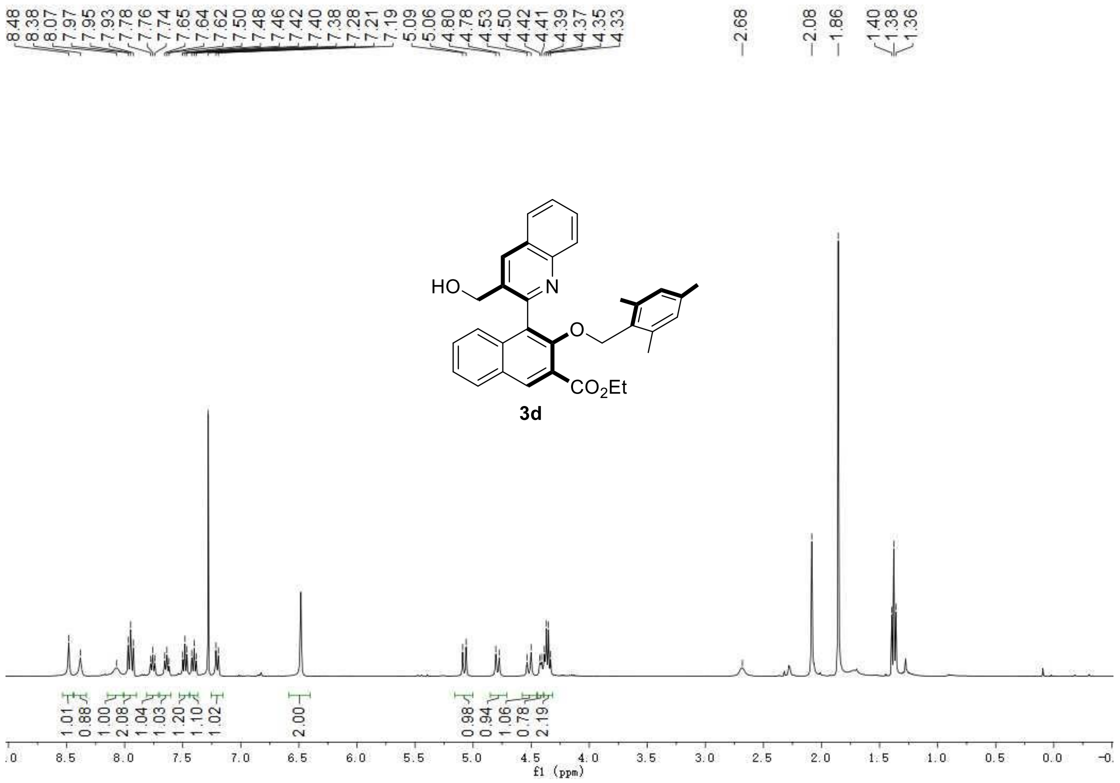

Supplementary Figure 57. ^{1}H NMR Spectra of **3d**

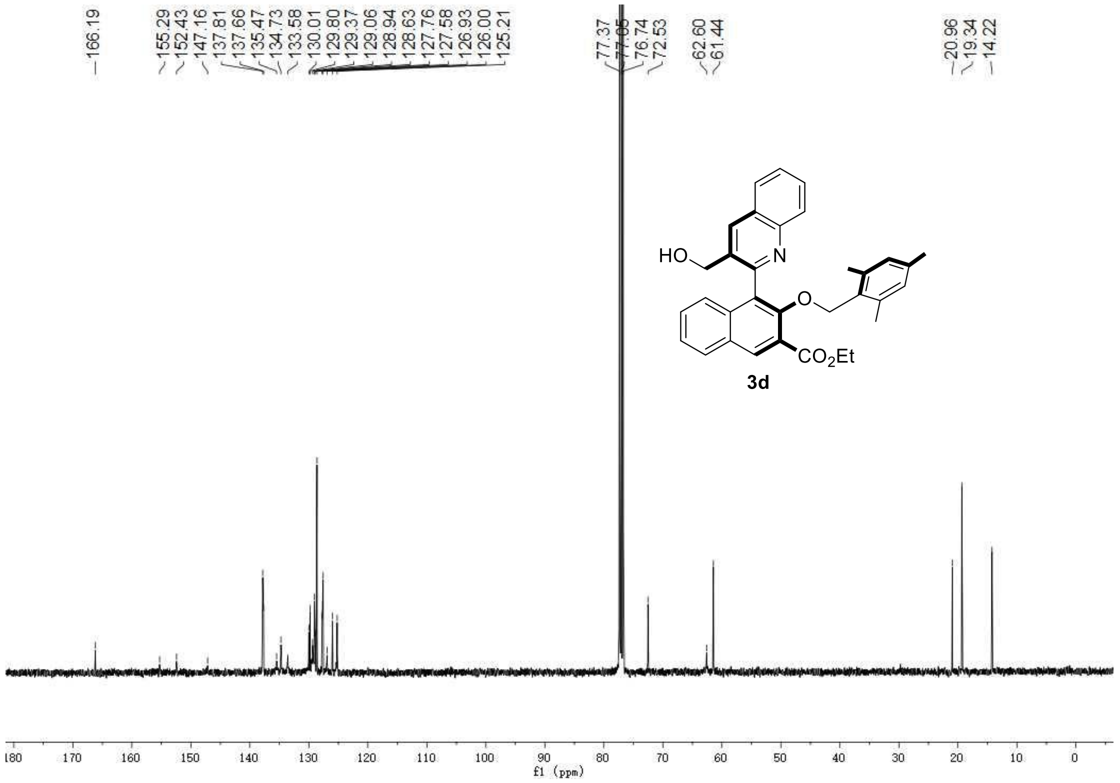

Supplementary Figure 58. ^{13}C NMR Spectra of **3d**

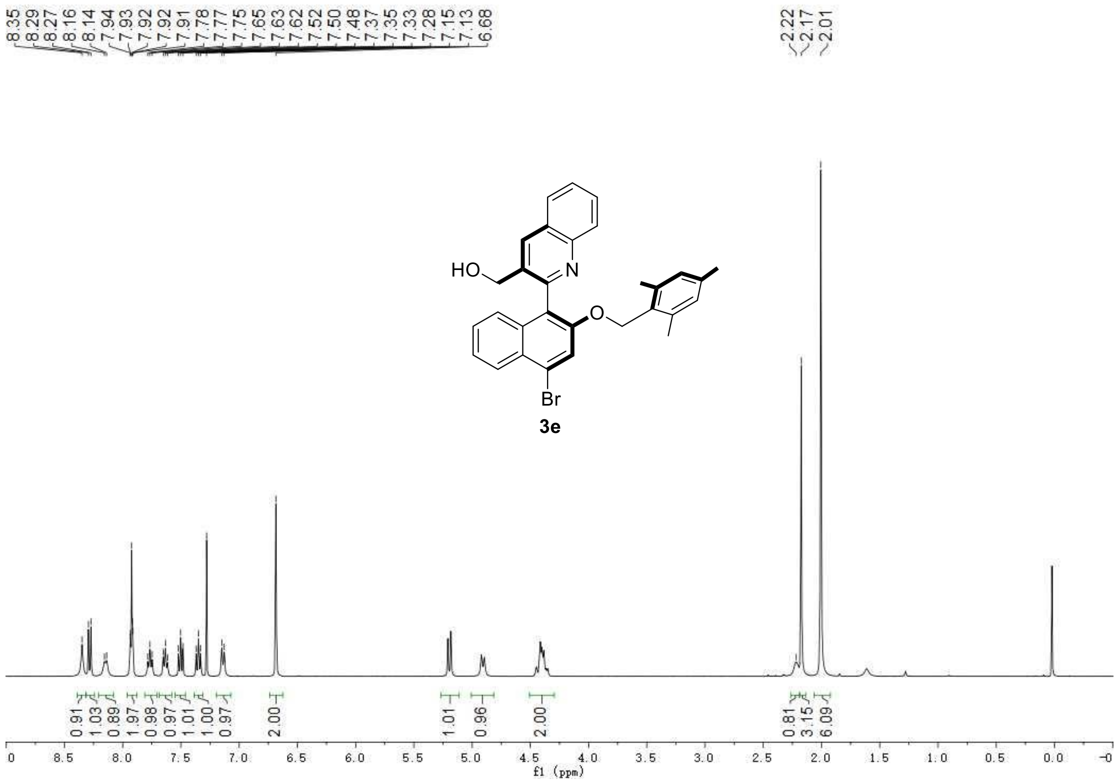

Supplementary Figure 59. ^{1}H NMR Spectra of **3e**

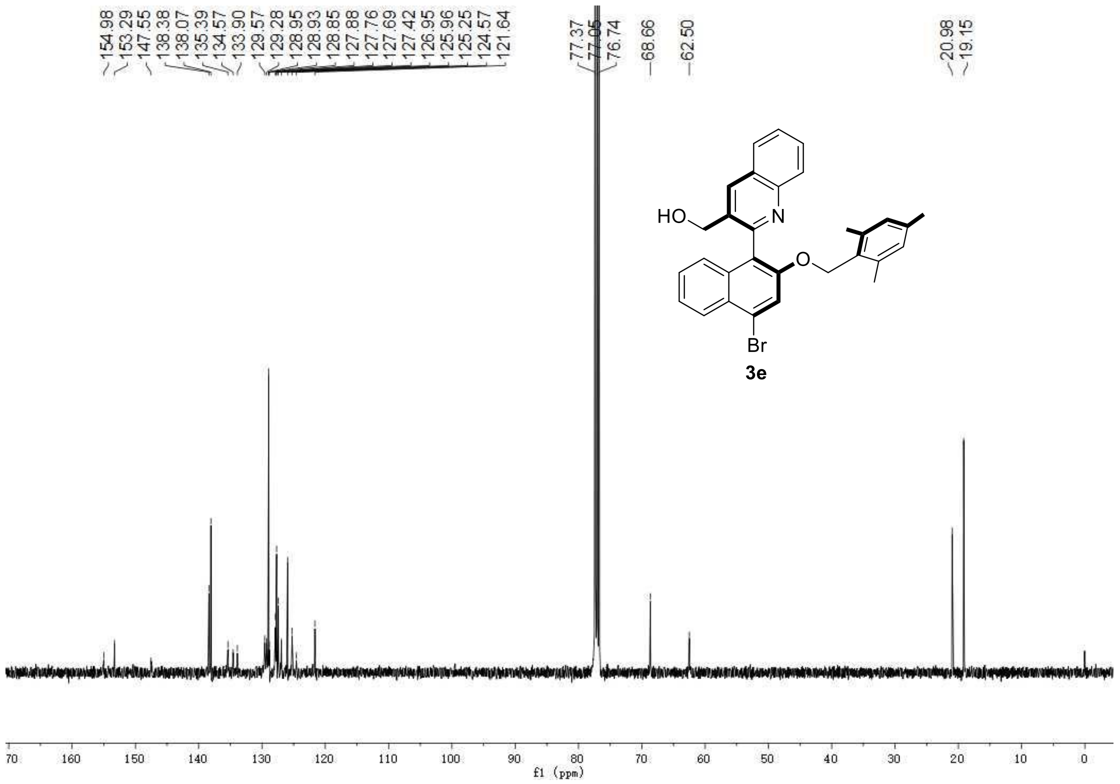

Supplementary Figure 60. ^{13}C NMR Spectra of **3e**

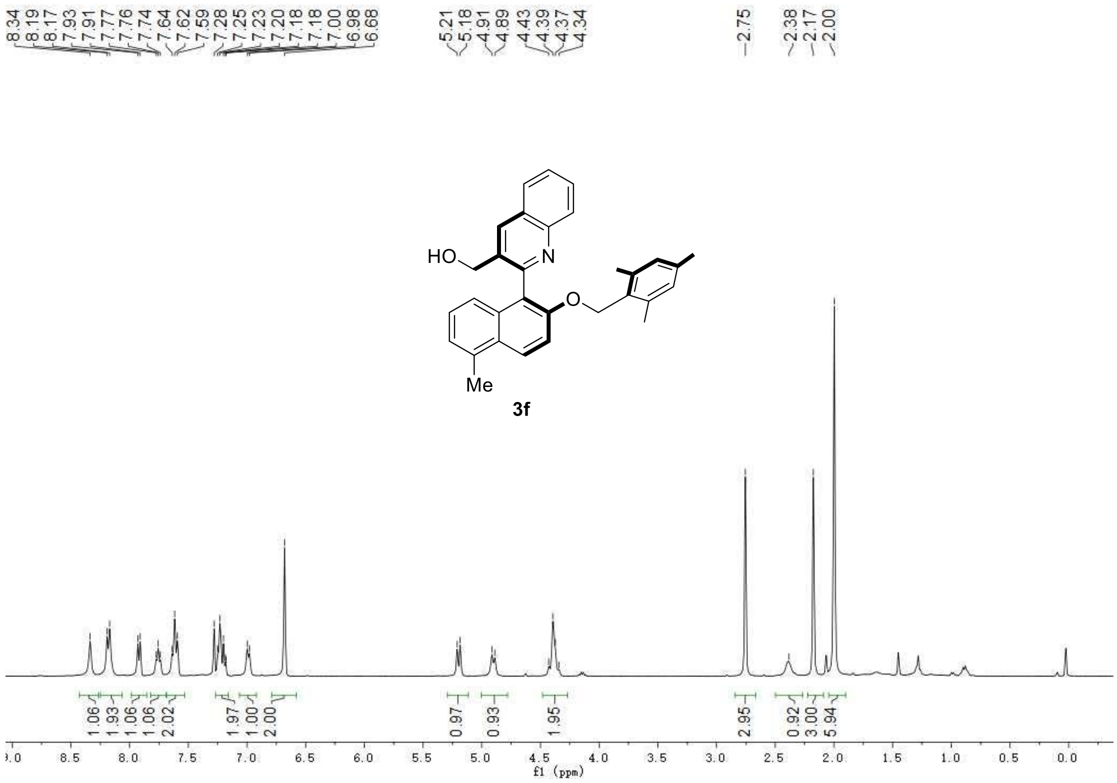

Supplementary Figure 61. ^{1}H NMR Spectra of **3f**

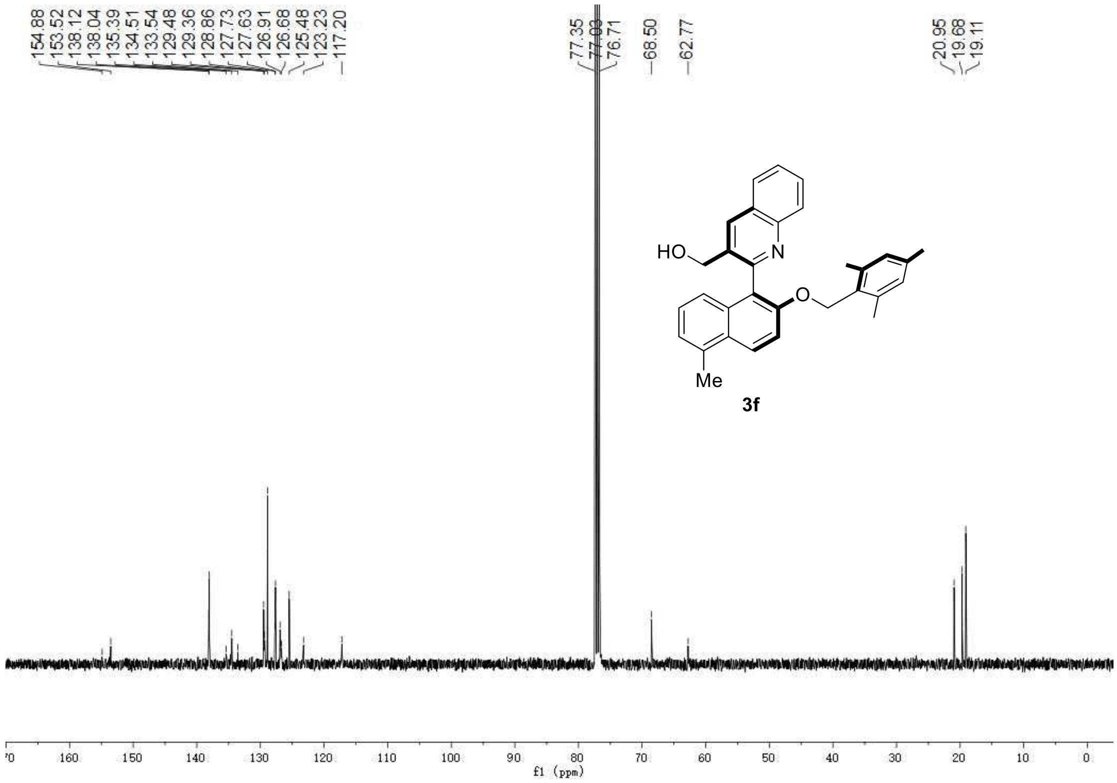

Supplementary Figure 62. ^{13}C NMR Spectra of **3f**

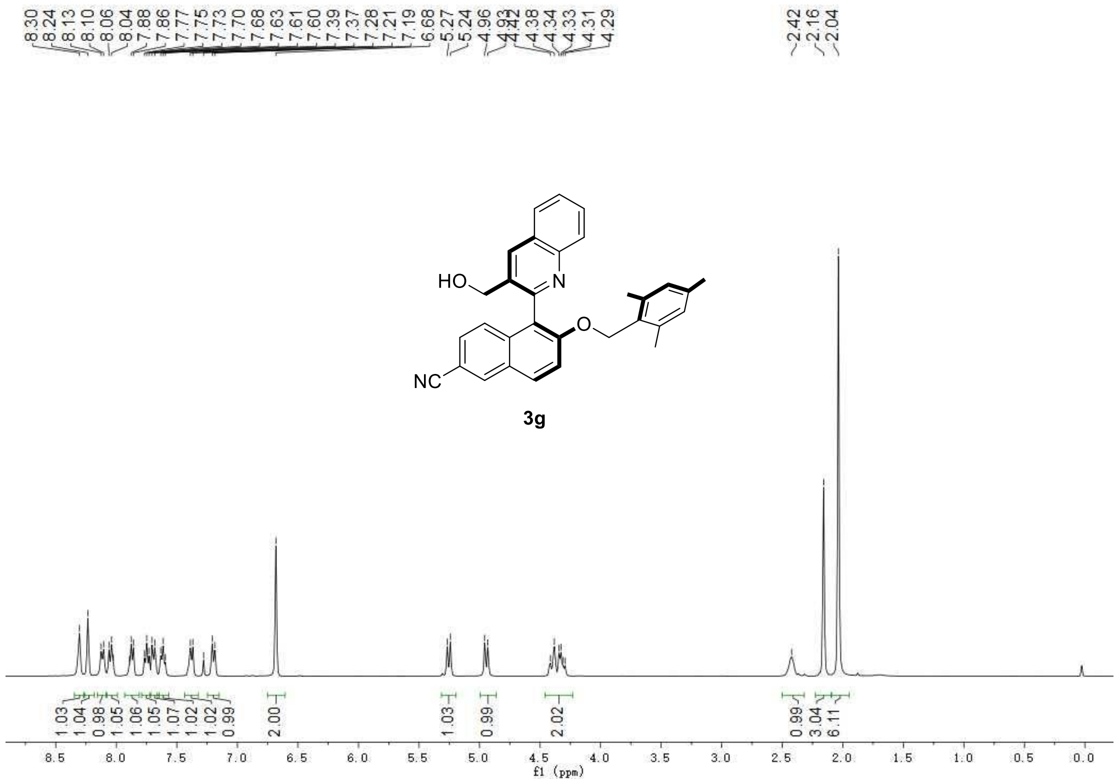

Supplementary Figure 63. ^{1}H NMR Spectra of **3g**

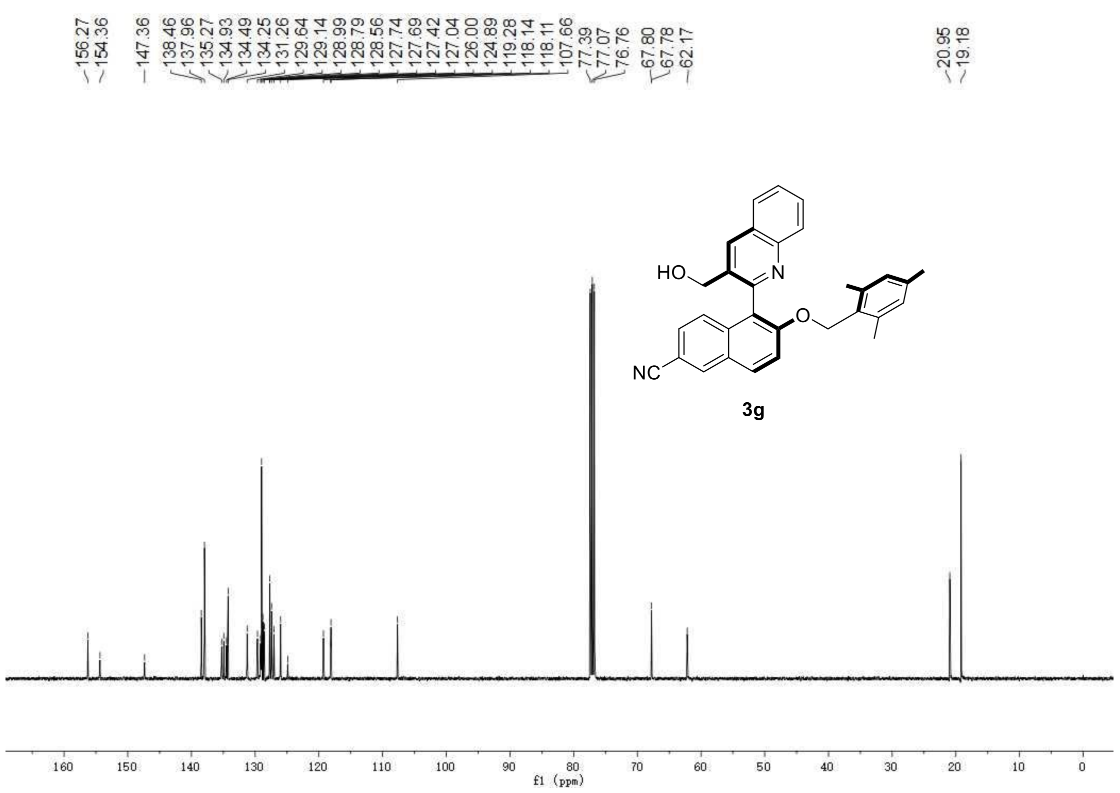

Supplementary Figure 64. ^{13}C NMR Spectra of **3g**

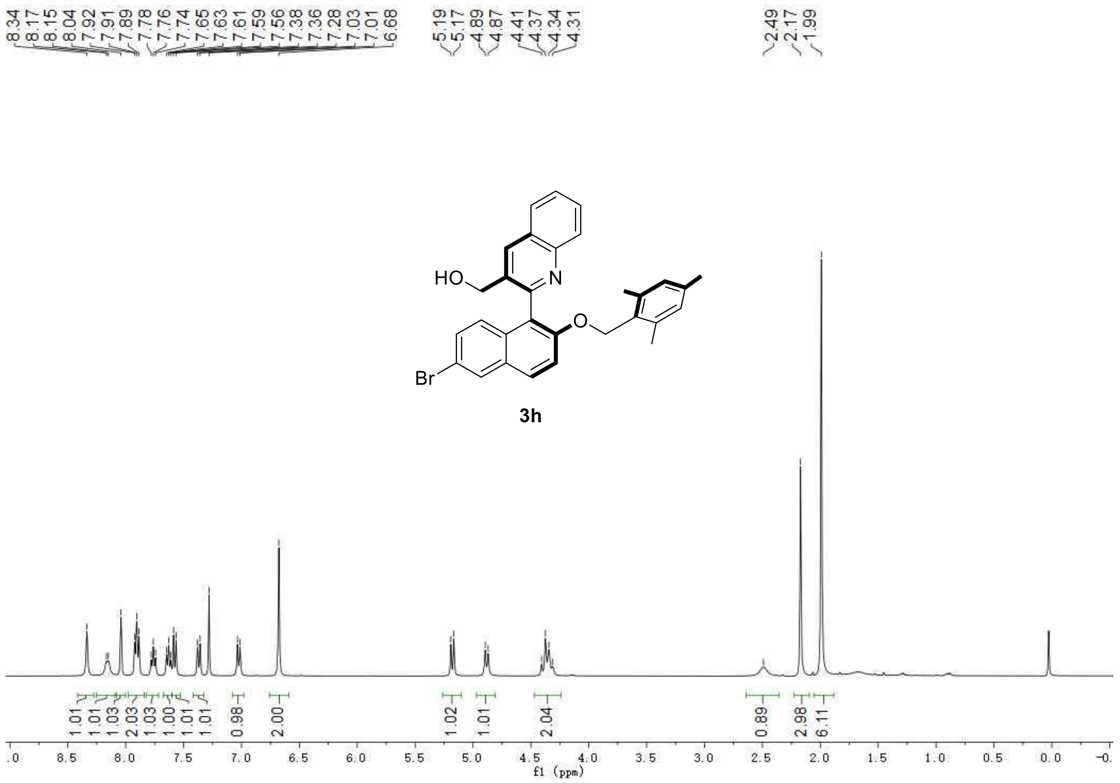

Supplementary Figure 65. ^{1}H NMR Spectra of **3h**

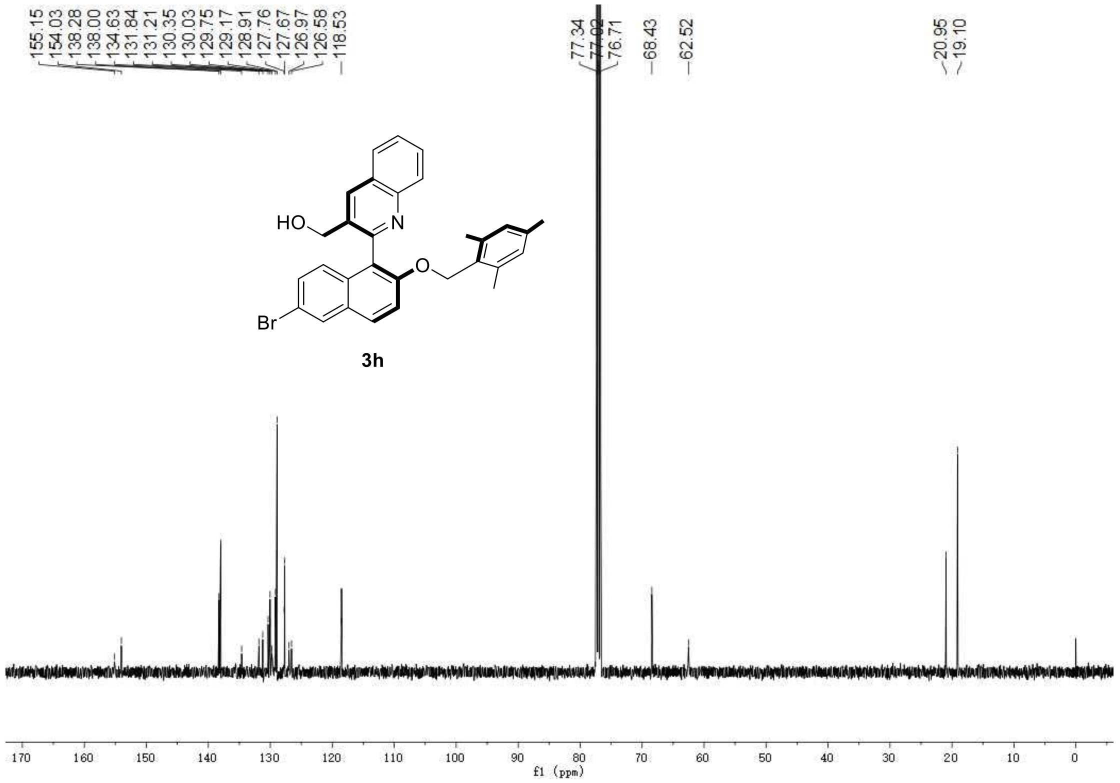

Supplementary Figure 66. ^{13}C NMR Spectra of **3h**

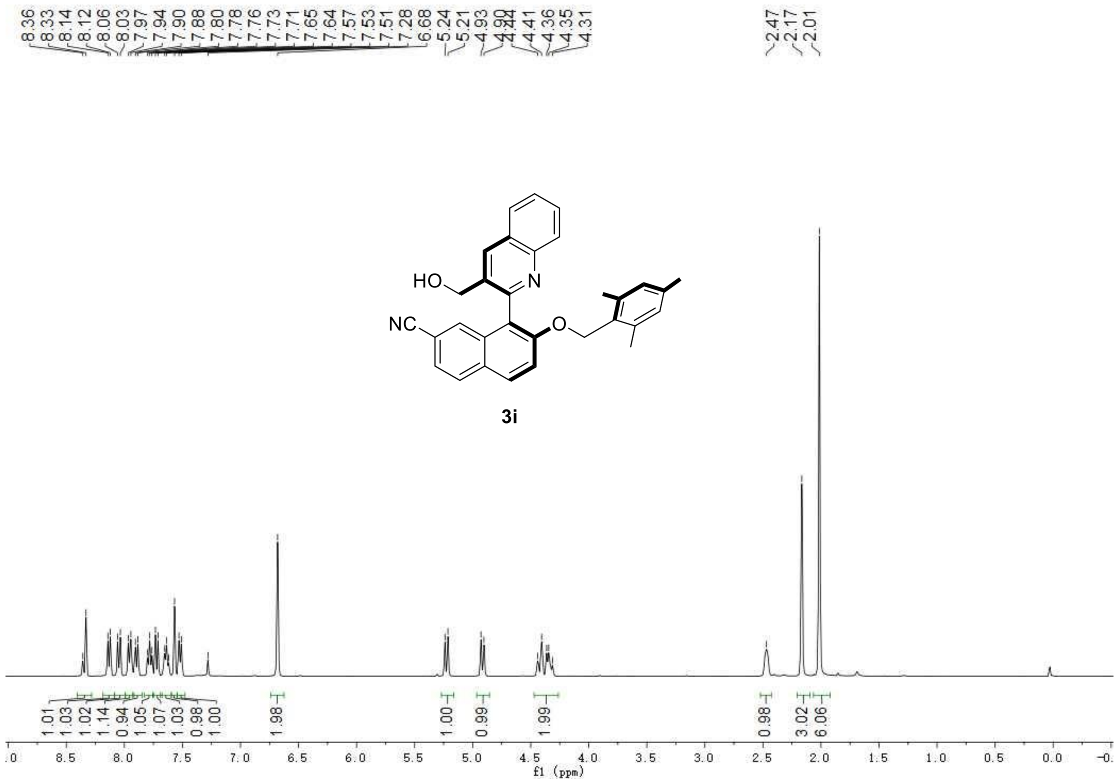

Supplementary Figure 67. ^{1}H NMR Spectra of **3i**

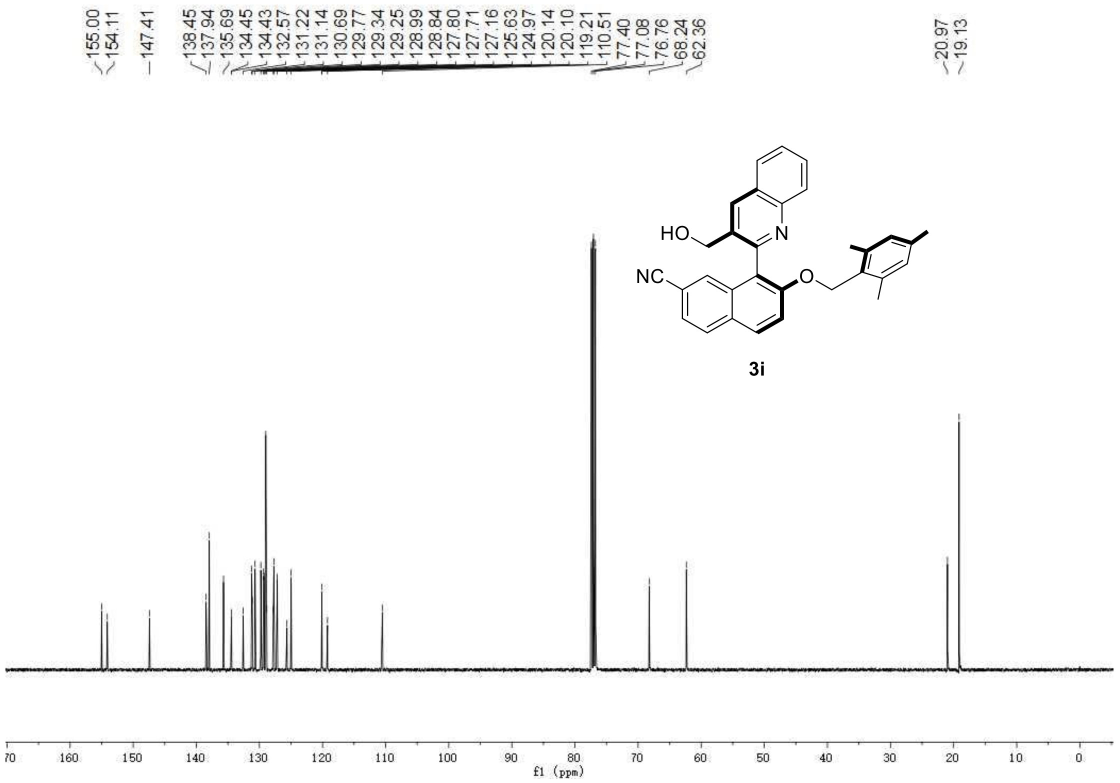

Supplementary Figure 68. ^{13}C NMR Spectra of **3i**

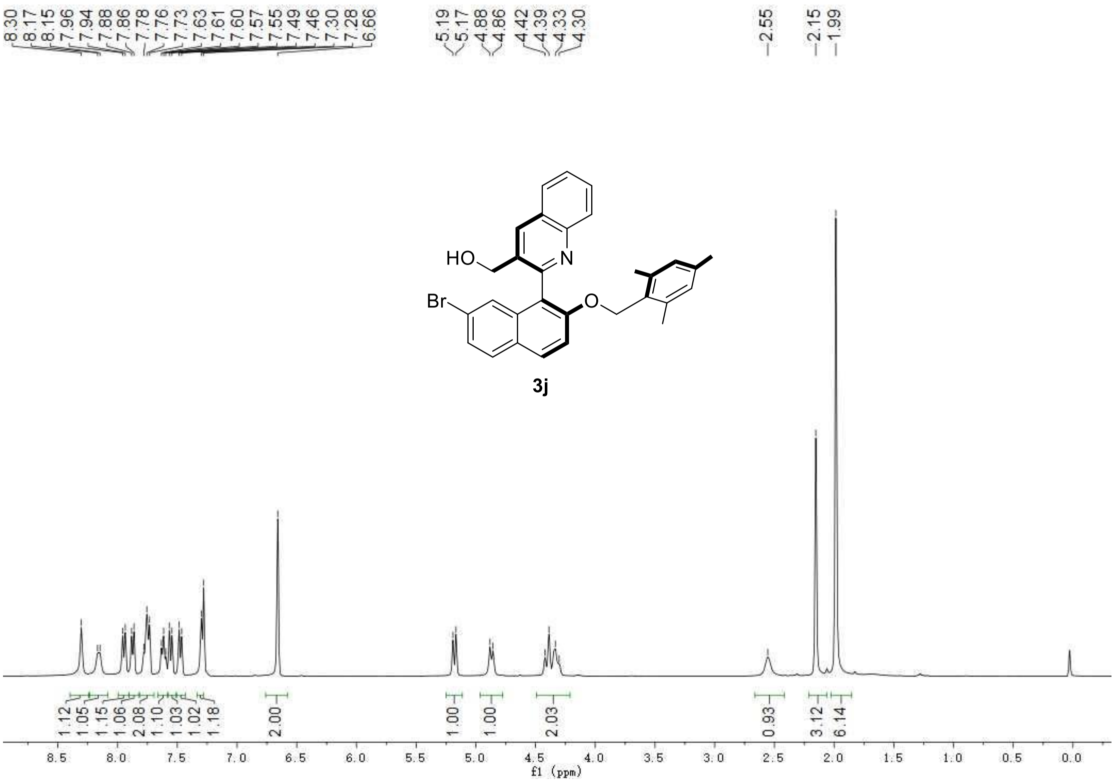

Supplementary Figure 69. ^{1}H NMR Spectra of **3j**

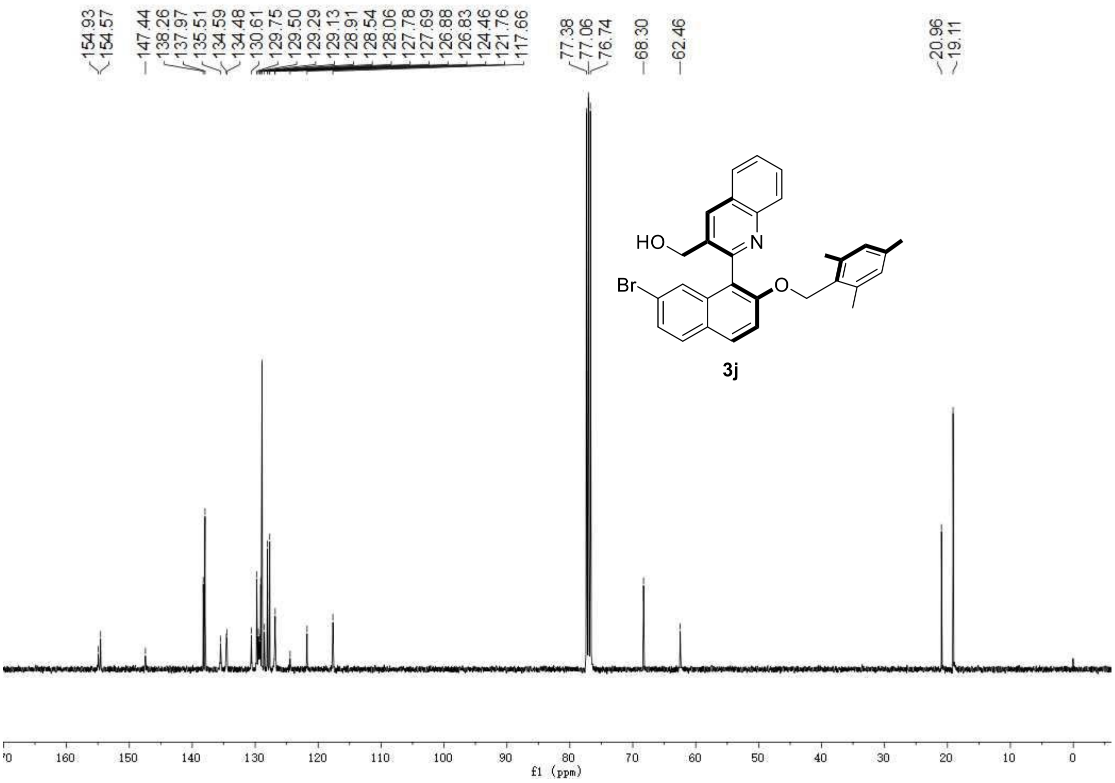

Supplementary Figure 70. ^{13}C NMR Spectra of **3j**

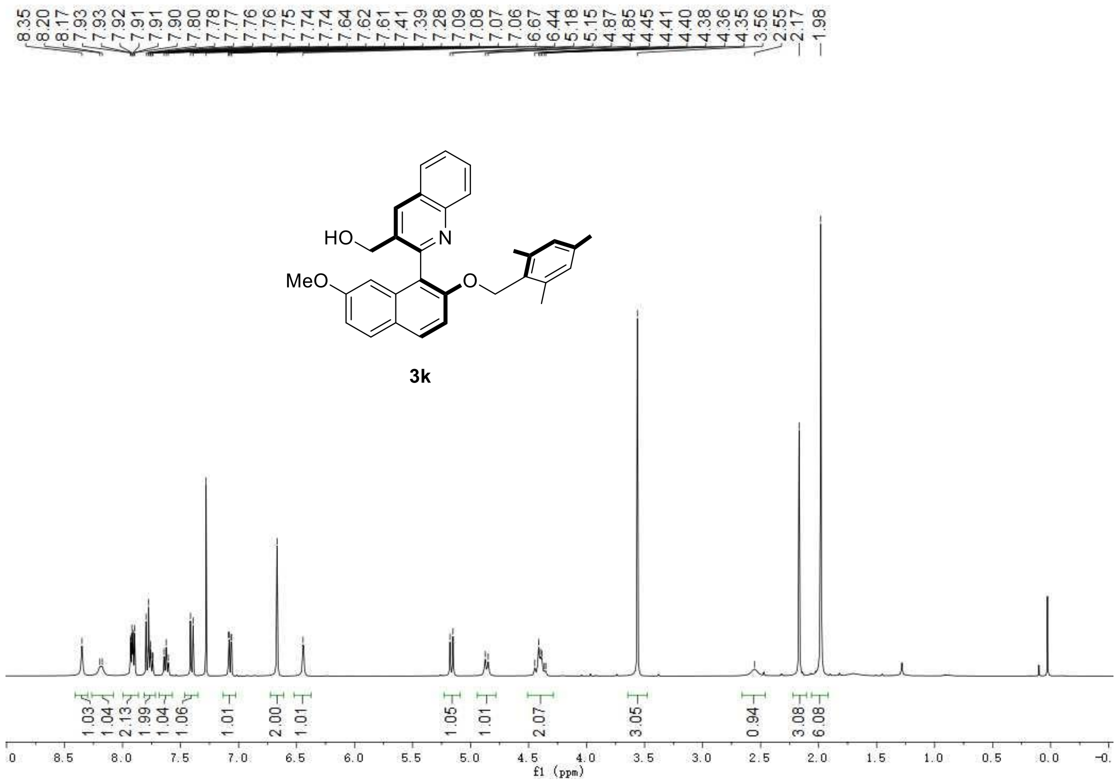

Supplementary Figure 71. ^{1}H NMR Spectra of **3k**

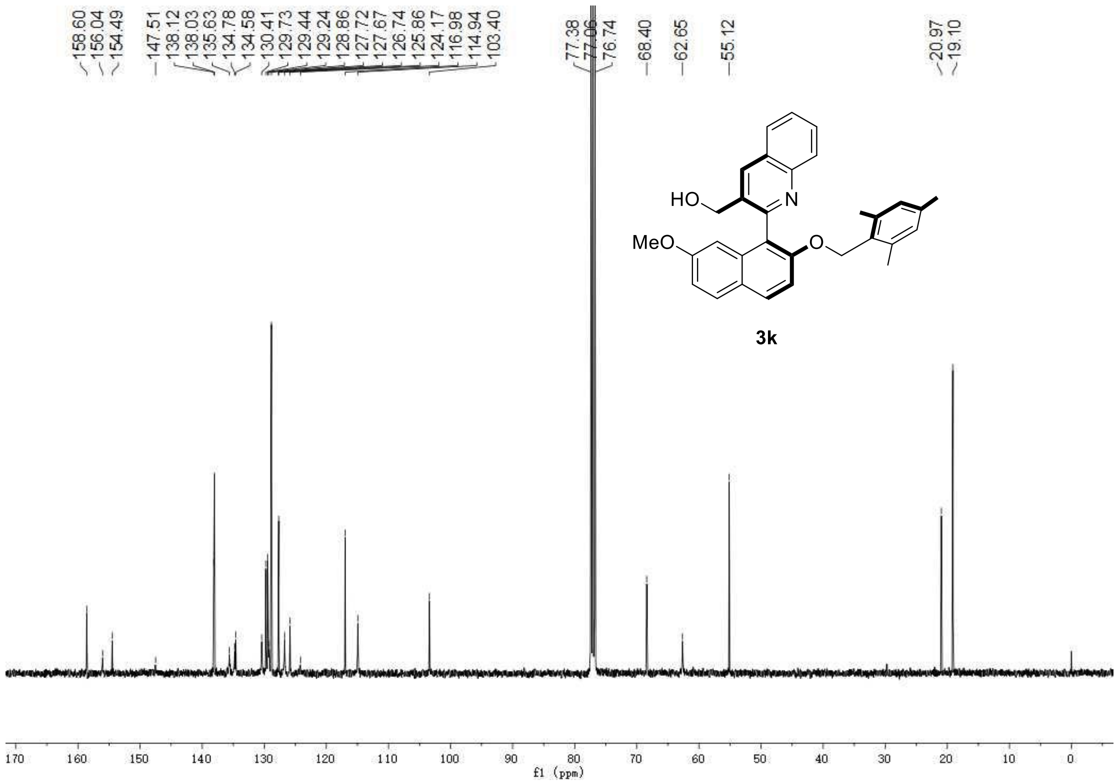

Supplementary Figure 72. ^{13}C NMR Spectra of **3k**

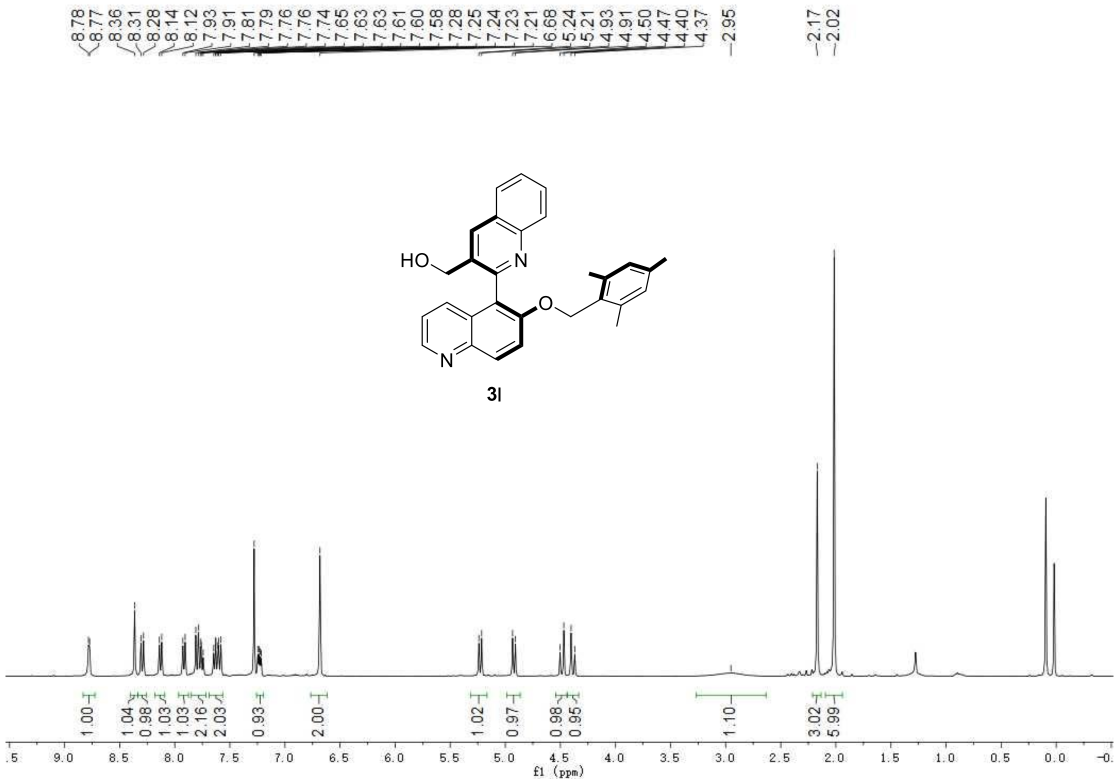

Supplementary Figure 73. ^{1}H NMR Spectra of **3l**

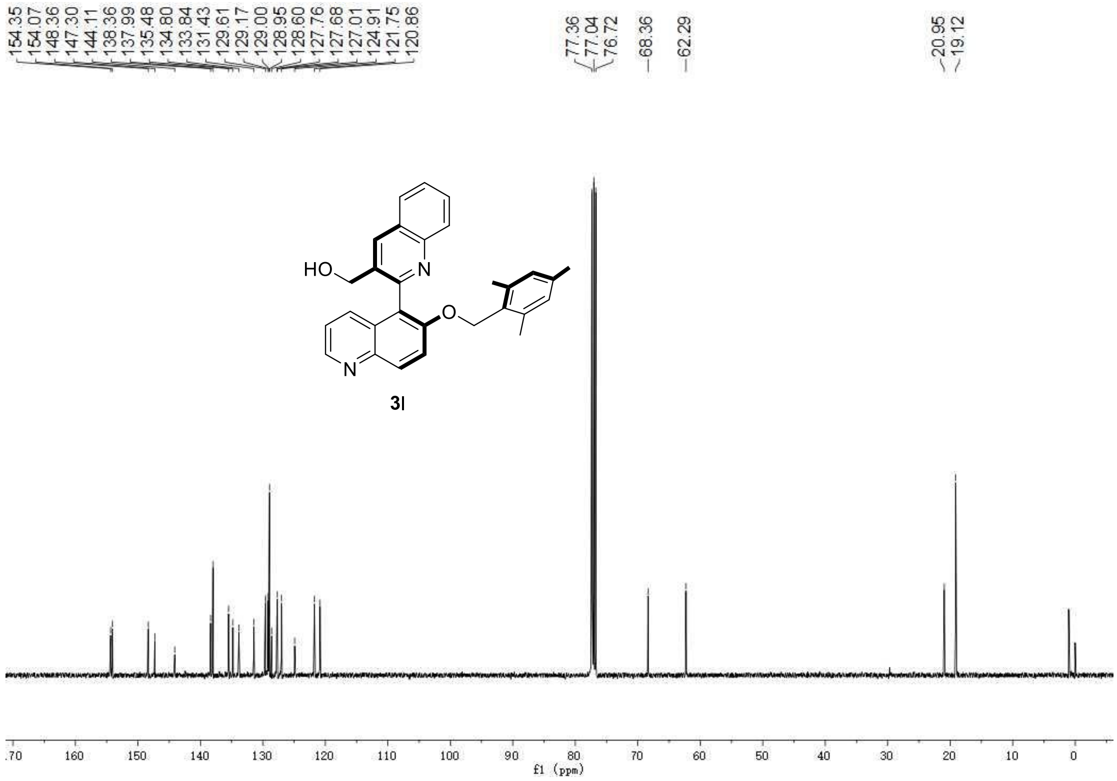

Supplementary Figure 74. ^{13}C NMR Spectra of **3l**

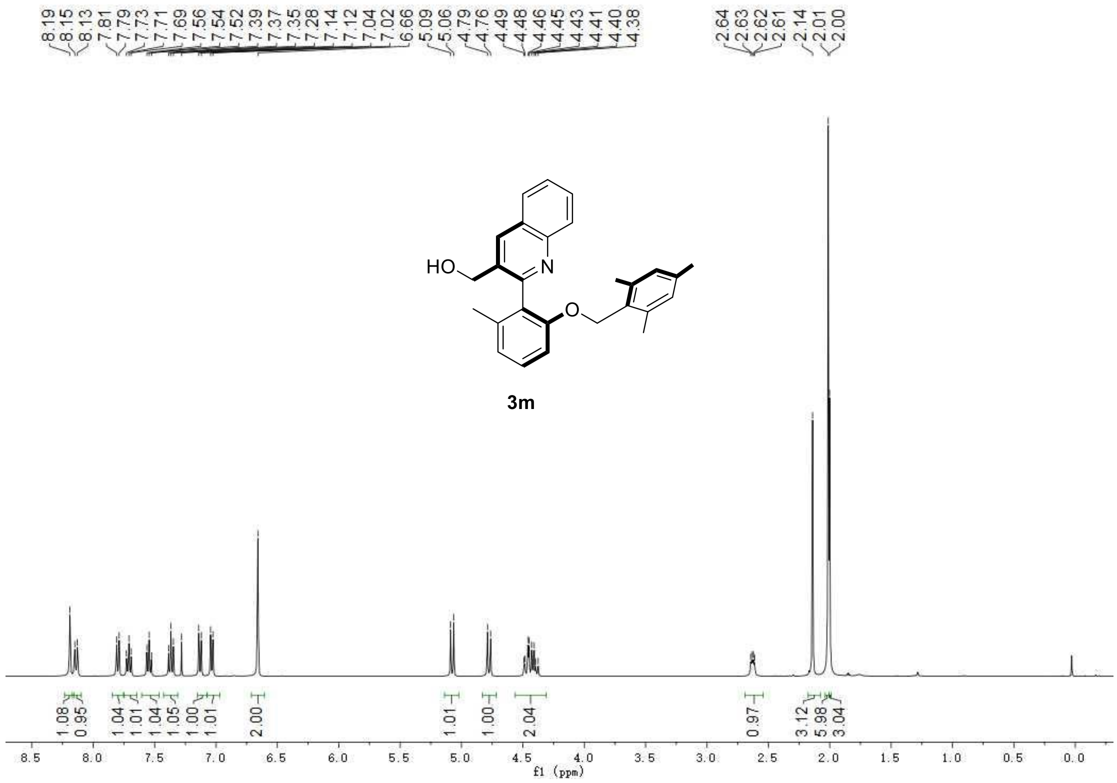

Supplementary Figure 75. ^{1}H NMR Spectra of **3m**

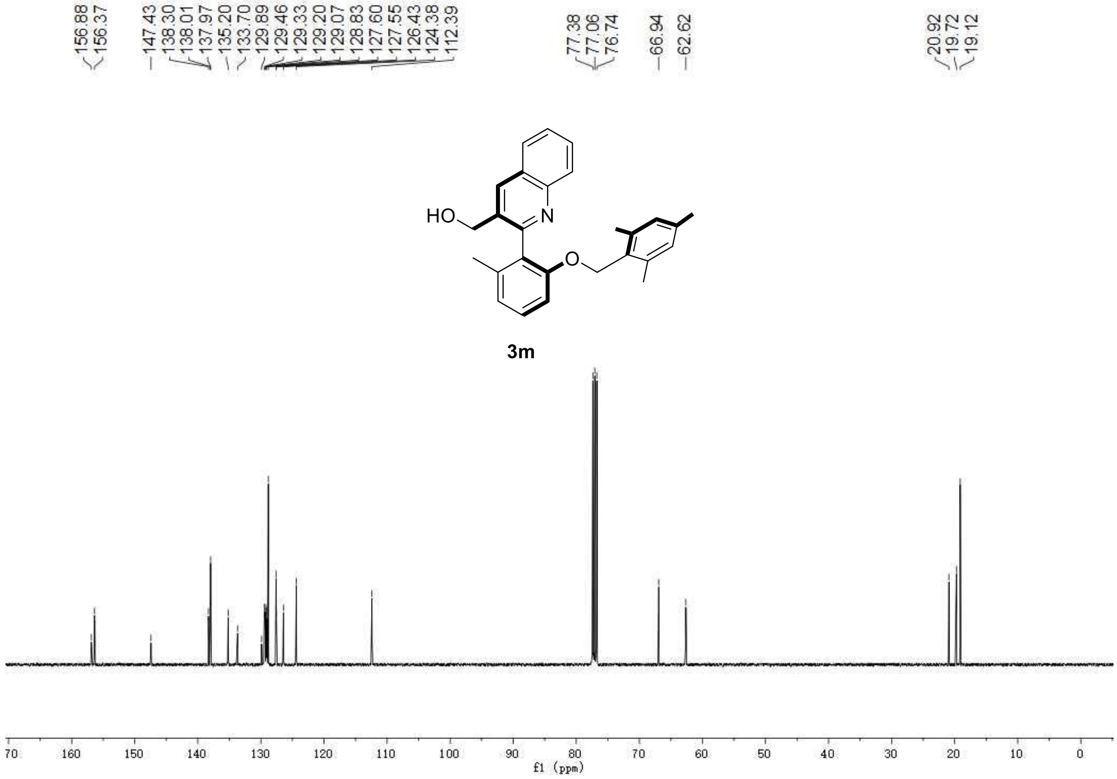

Supplementary Figure 76. ^{13}C NMR Spectra of **3m**

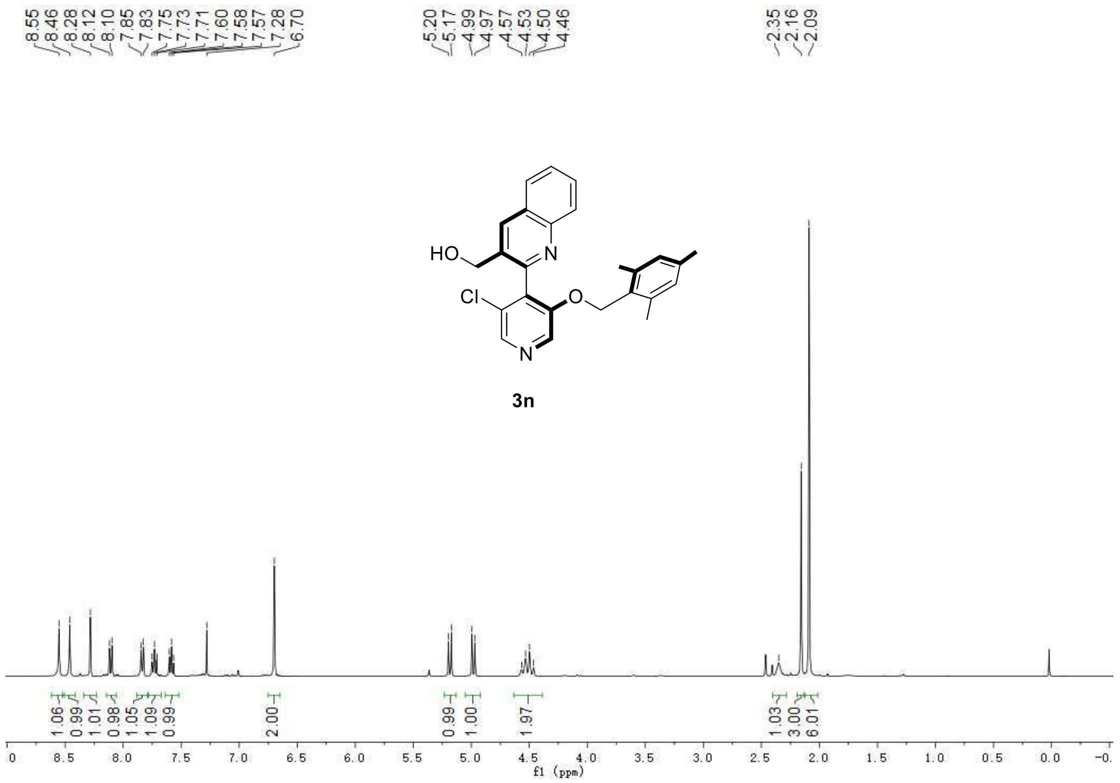

Supplementary Figure 77. ^{1}H NMR Spectra of **3n**

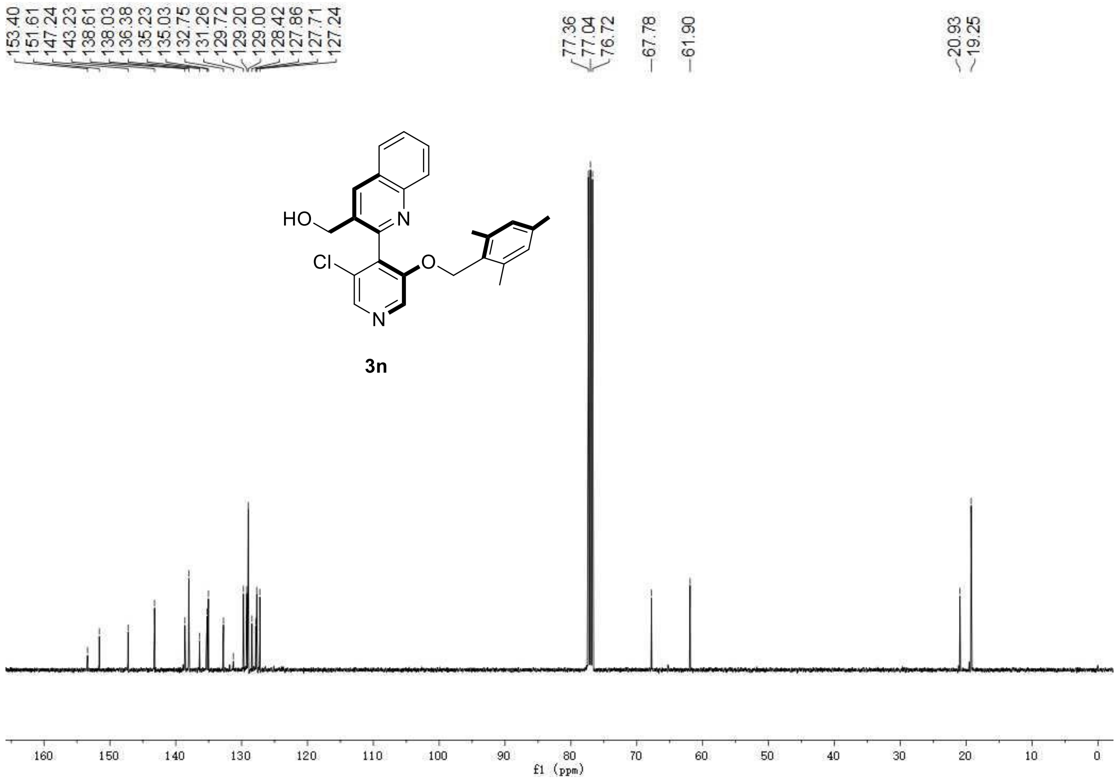

Supplementary Figure 78. ^{13}C NMR Spectra of **3n**

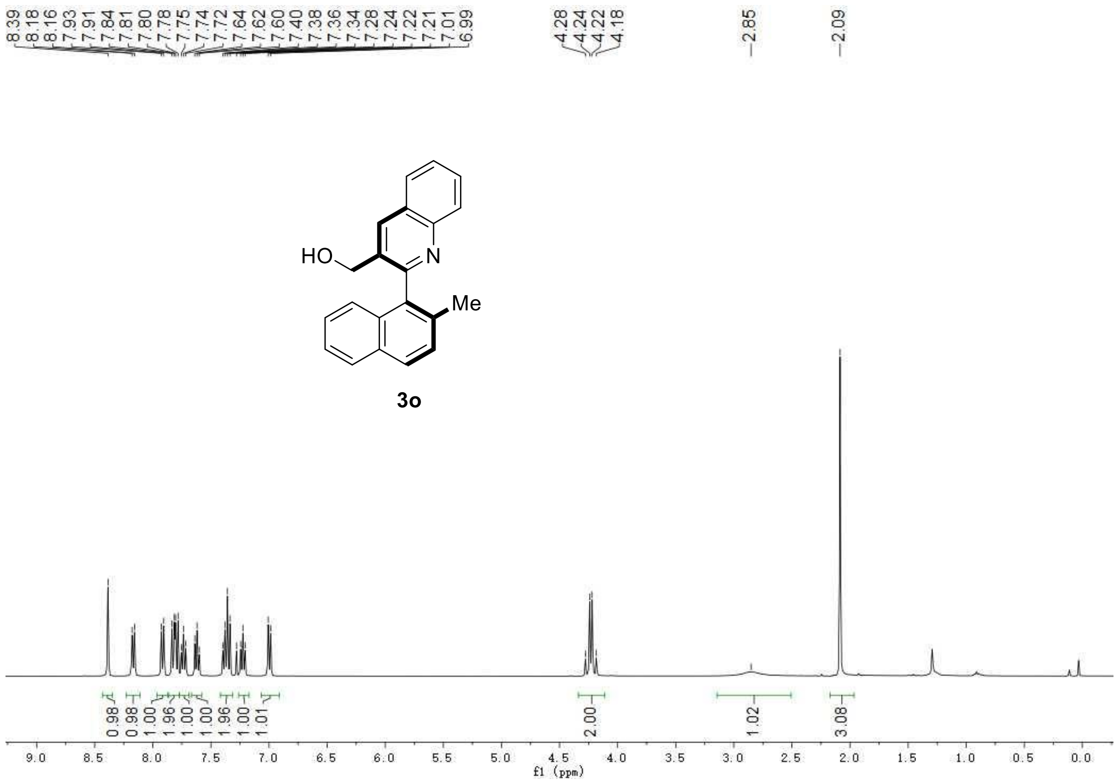

Supplementary Figure 79. ^{1}H NMR Spectra of **3o**

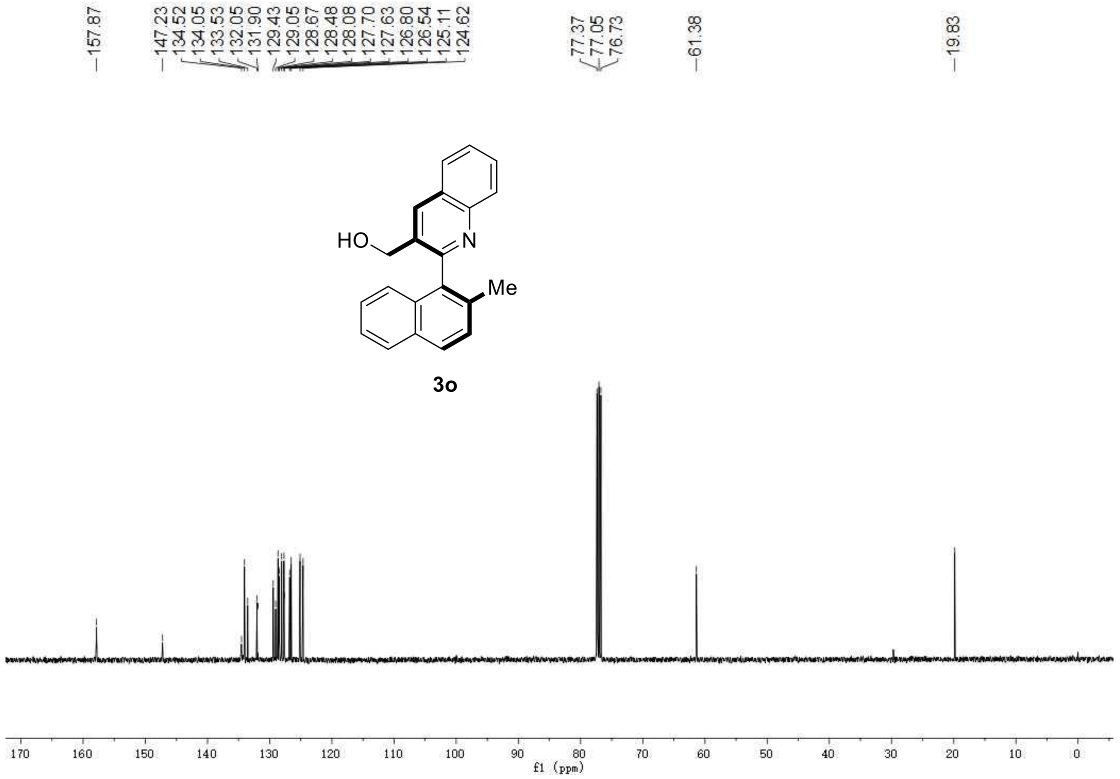

Supplementary Figure 80. ^{13}C NMR Spectra of **3o**

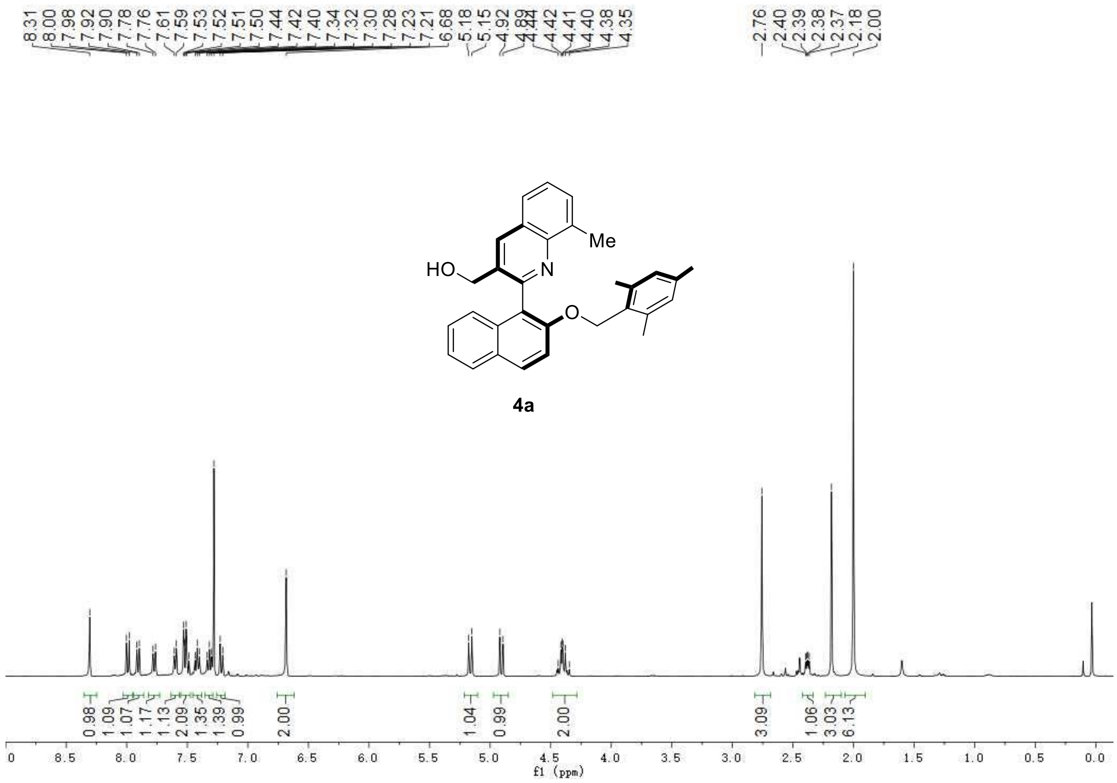

Supplementary Figure 81. ^{1}H NMR Spectra of **4a**

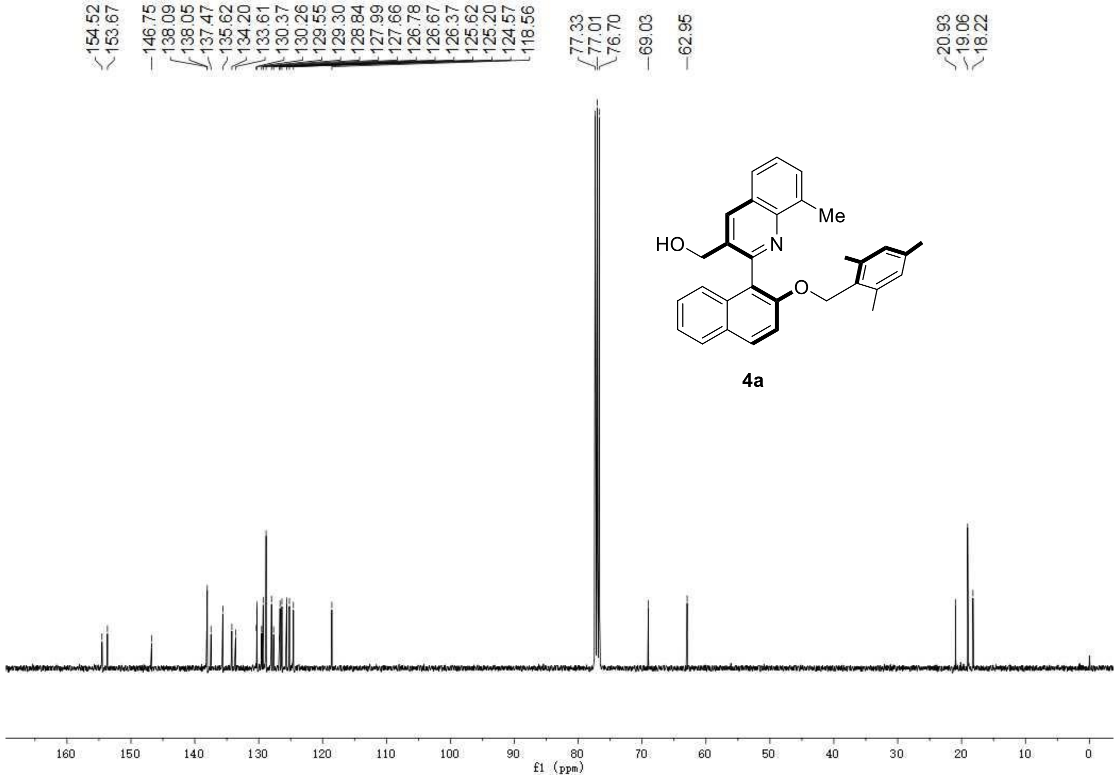

Supplementary Figure 82. ^{13}C NMR Spectra of **4a**

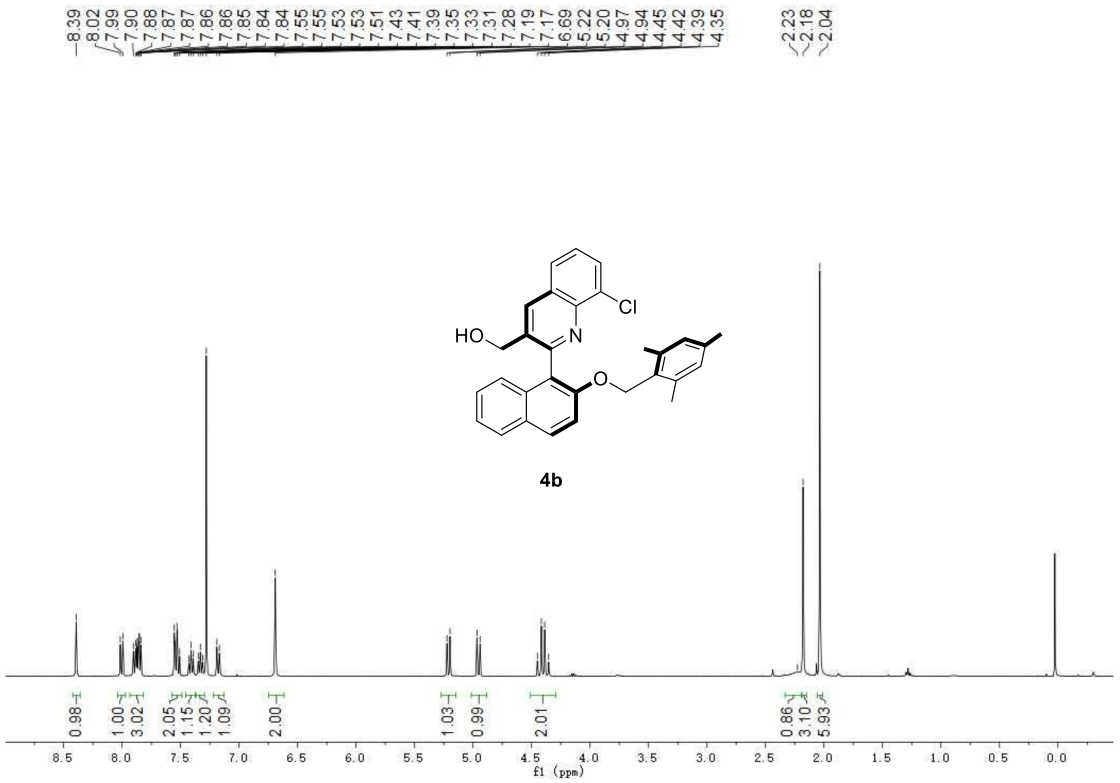

Supplementary Figure 83. ^{1}H NMR Spectra of **4b**

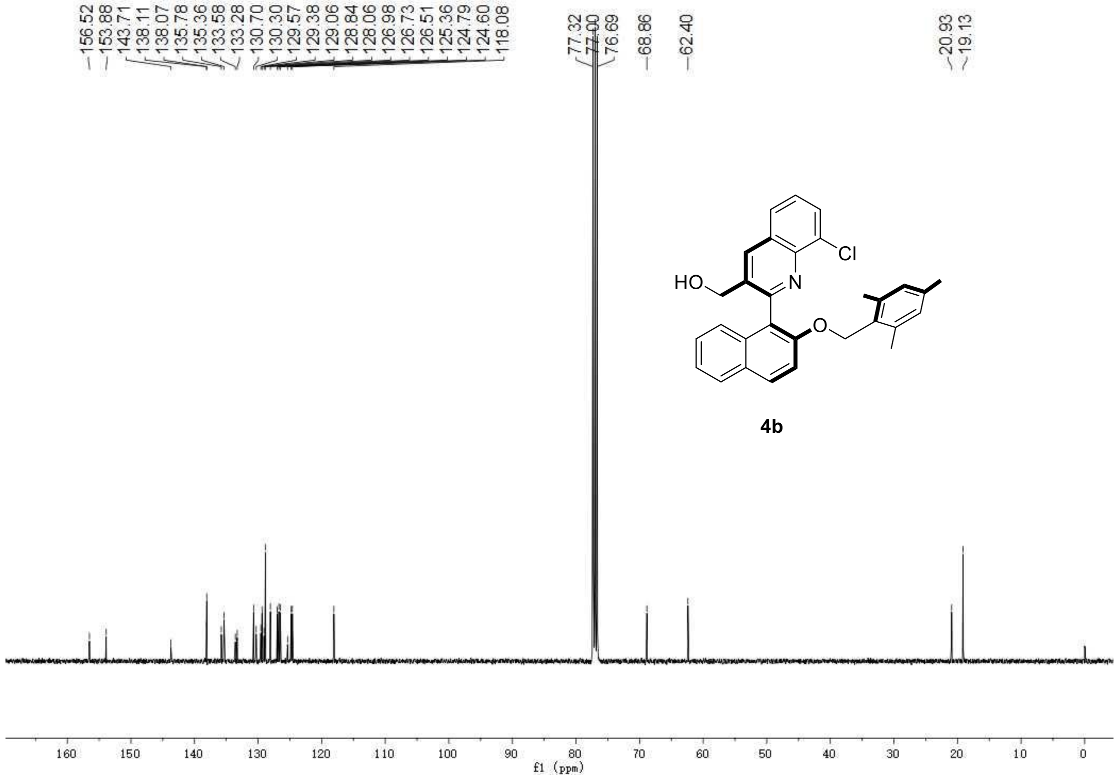

Supplementary Figure 84. ^{13}C NMR Spectra of **4b**

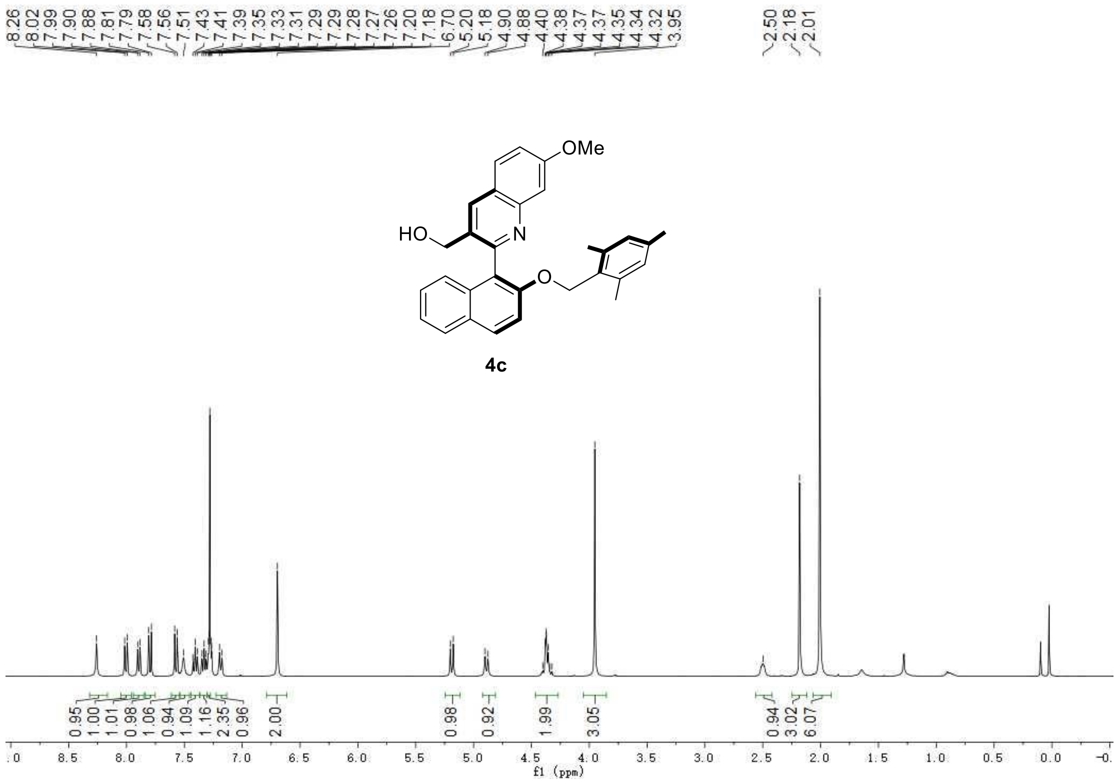

Supplementary Figure 85. ^{1}H NMR Spectra of **4c**

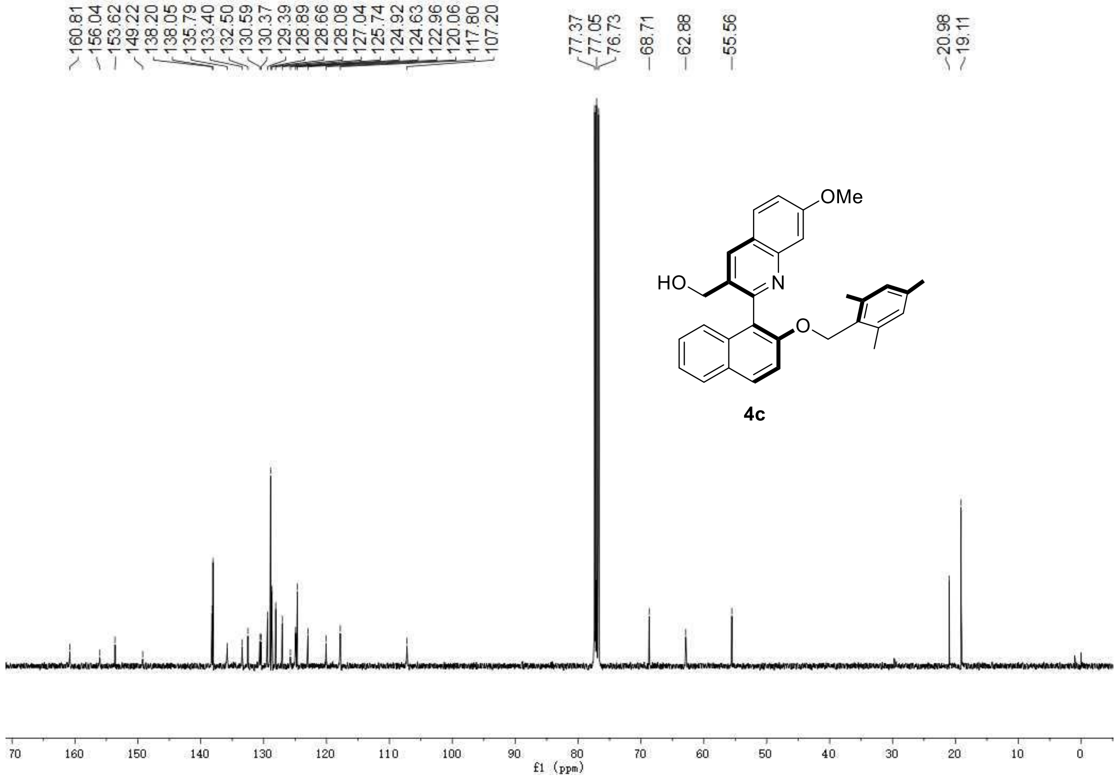

Supplementary Figure 86. ^{13}C NMR Spectra of **4c**

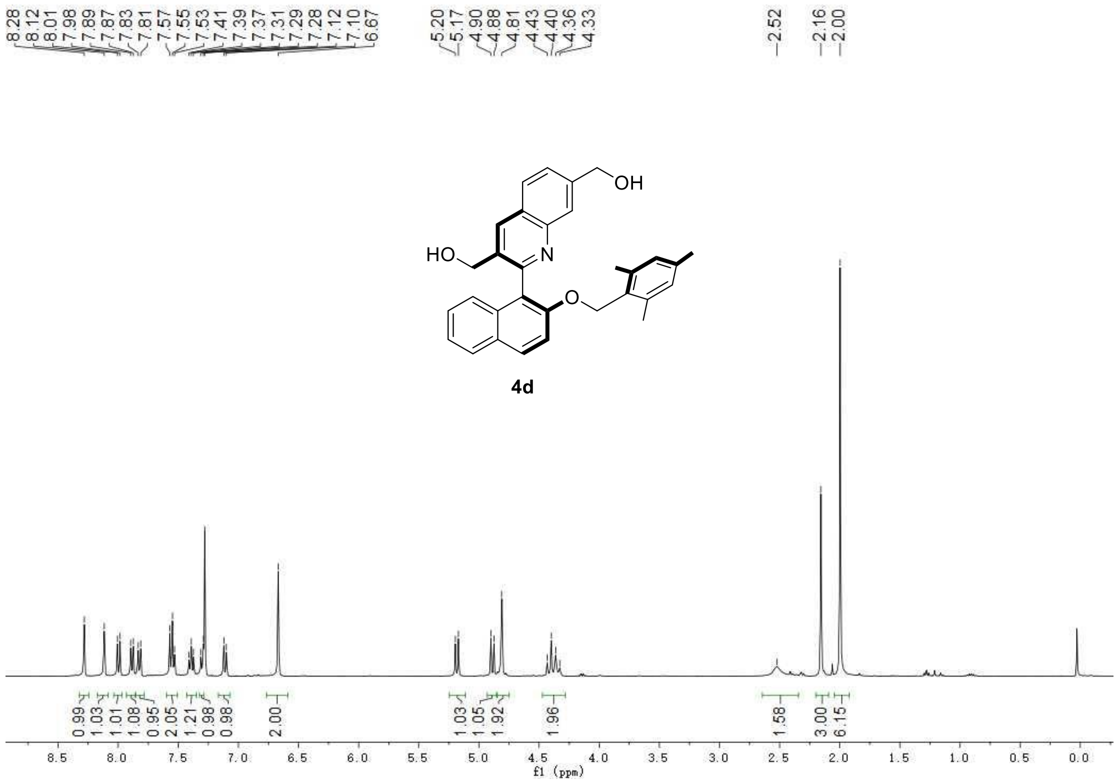

Supplementary Figure 87. ^{1}H NMR Spectra of **4d**

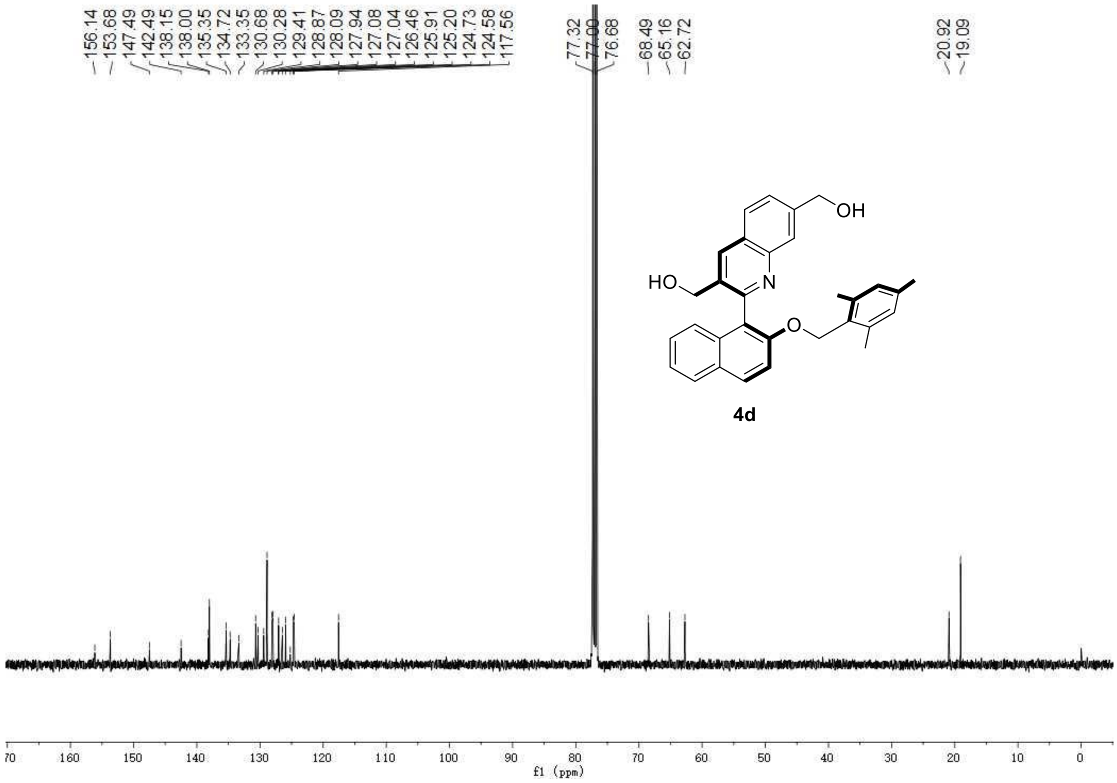

Supplementary Figure 88. ^{13}C NMR Spectra of **4d**

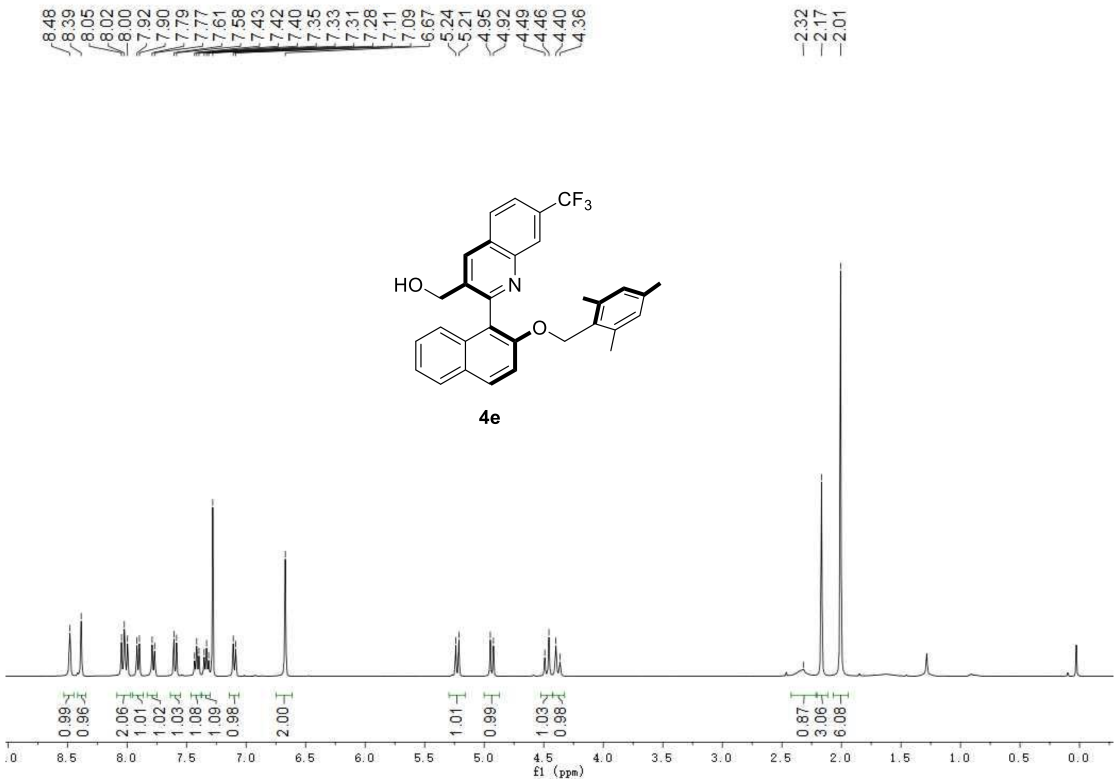

Supplementary Figure 89. ^{1}H NMR Spectra of **4e**

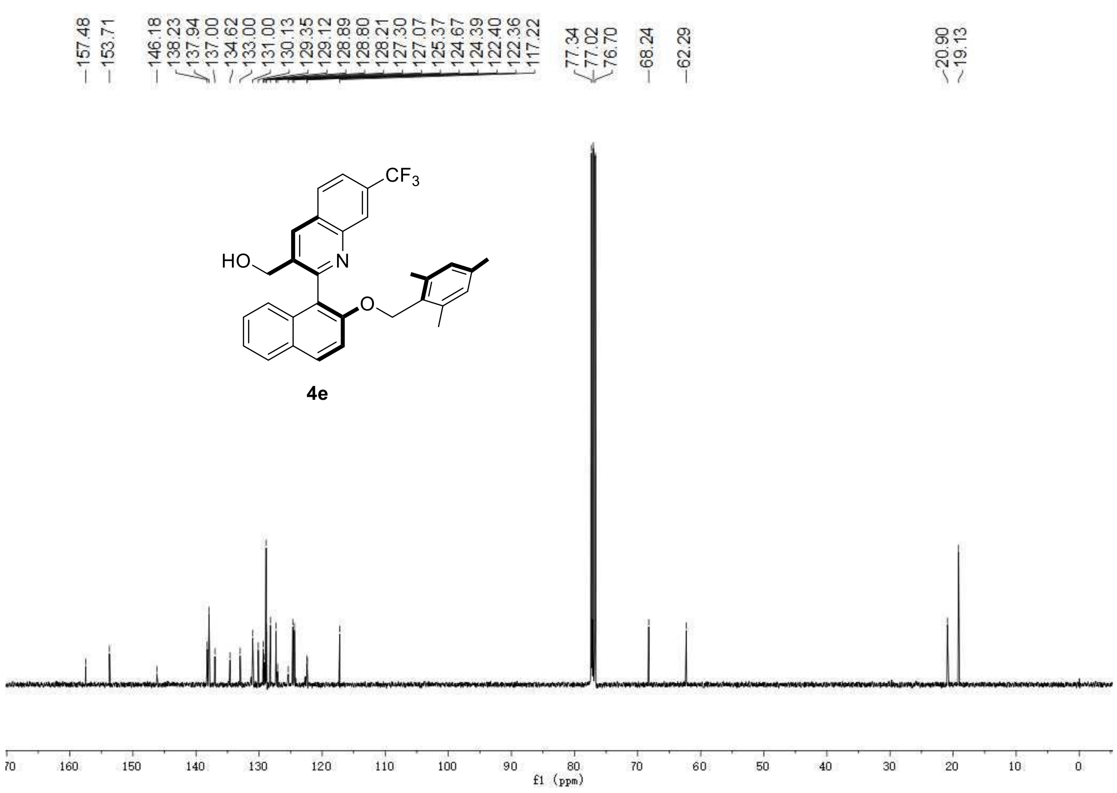

Supplementary Figure 90. ^{13}C NMR Spectra of **4e**

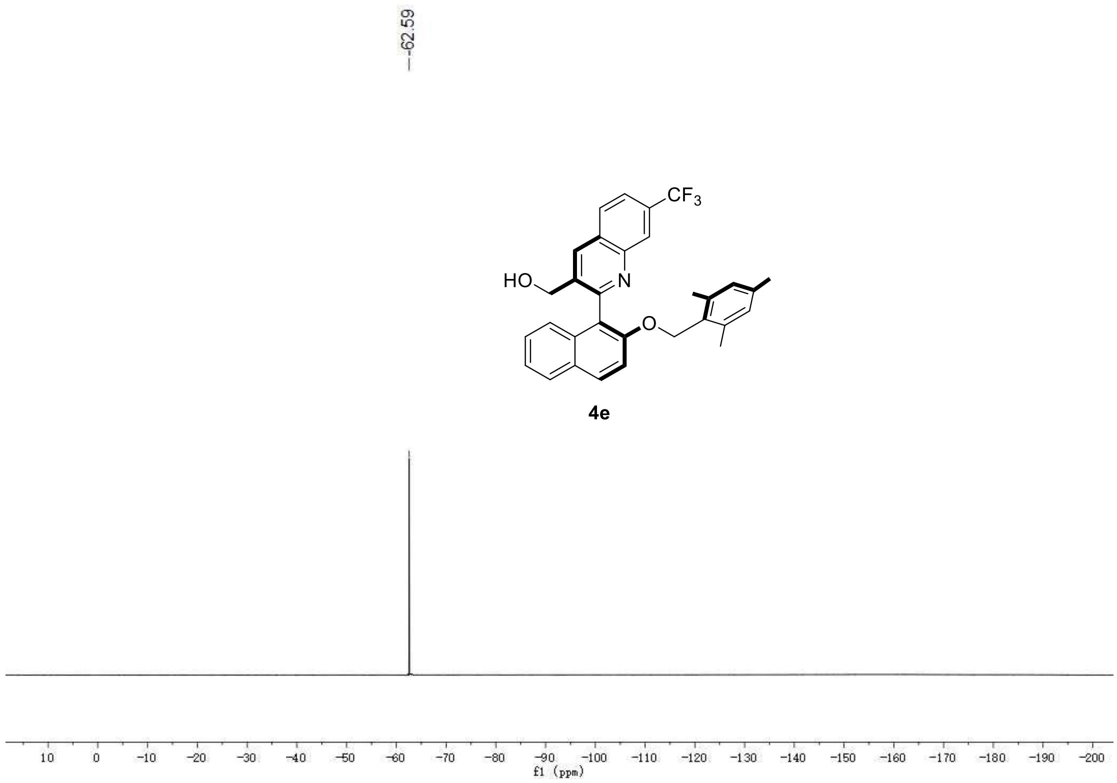

Supplementary Figure 91. ^{19}F NMR Spectra of **4e**

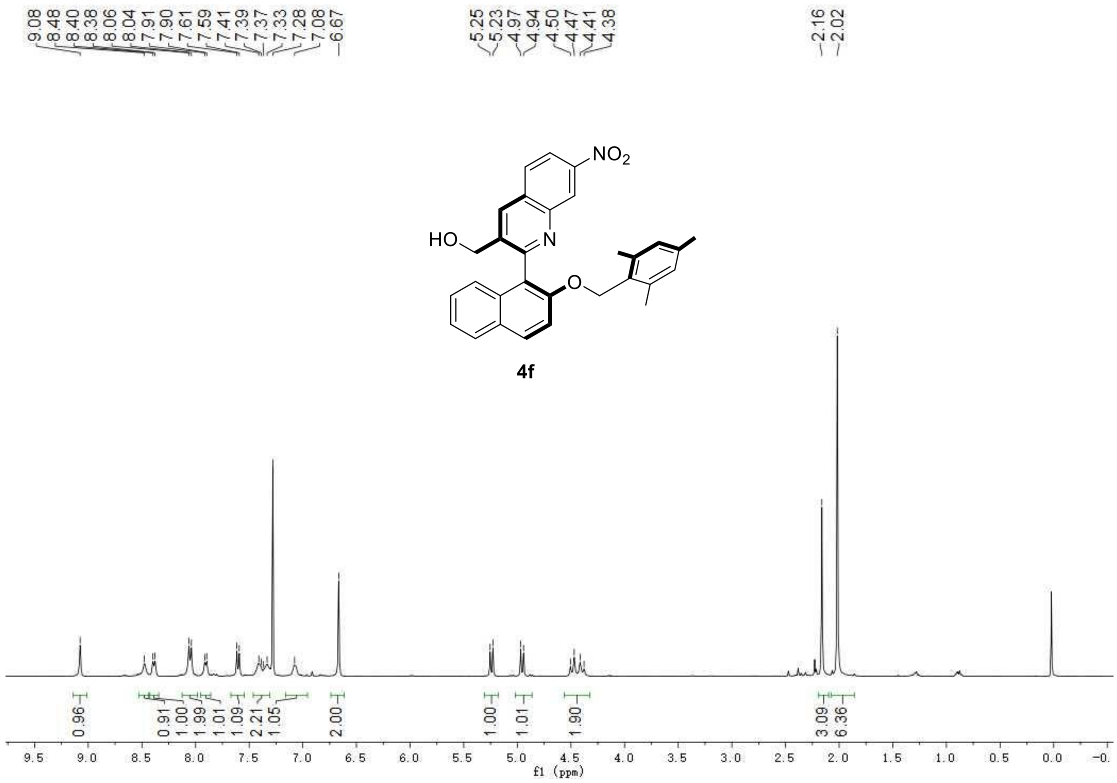

Supplementary Figure 92. ^{1}H NMR Spectra of **4f**

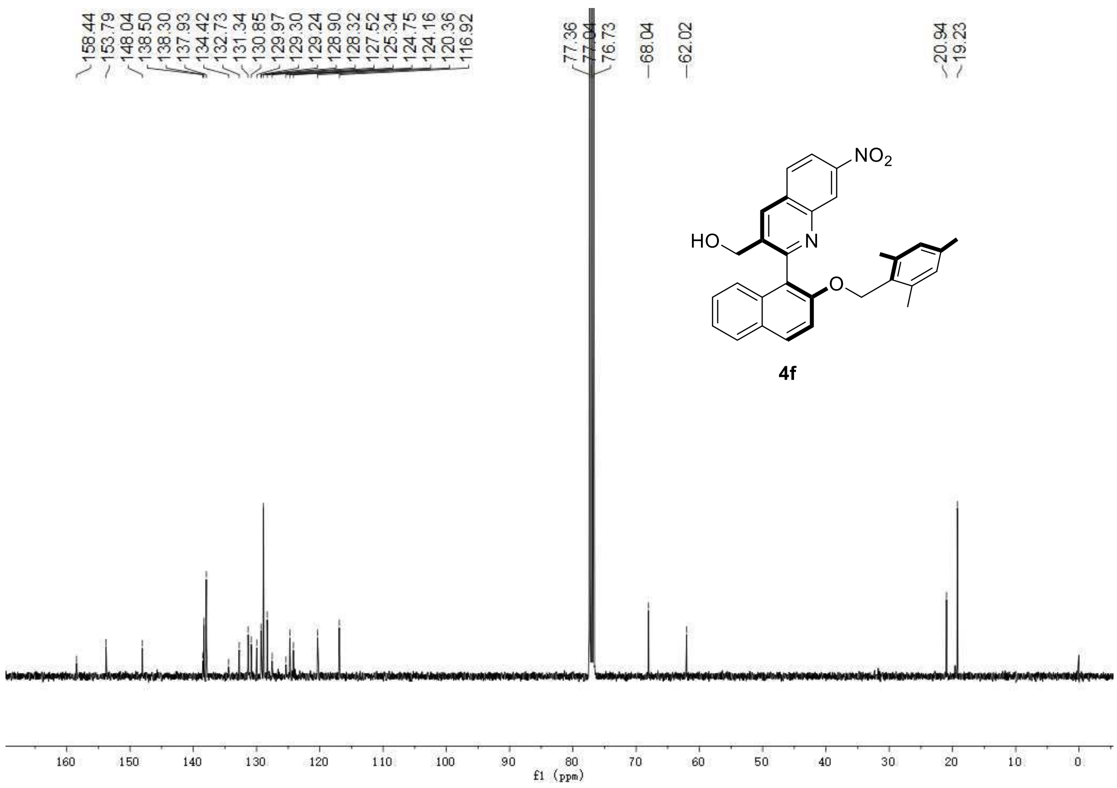

Supplementary Figure 93. ^{13}C NMR Spectra of **4f**

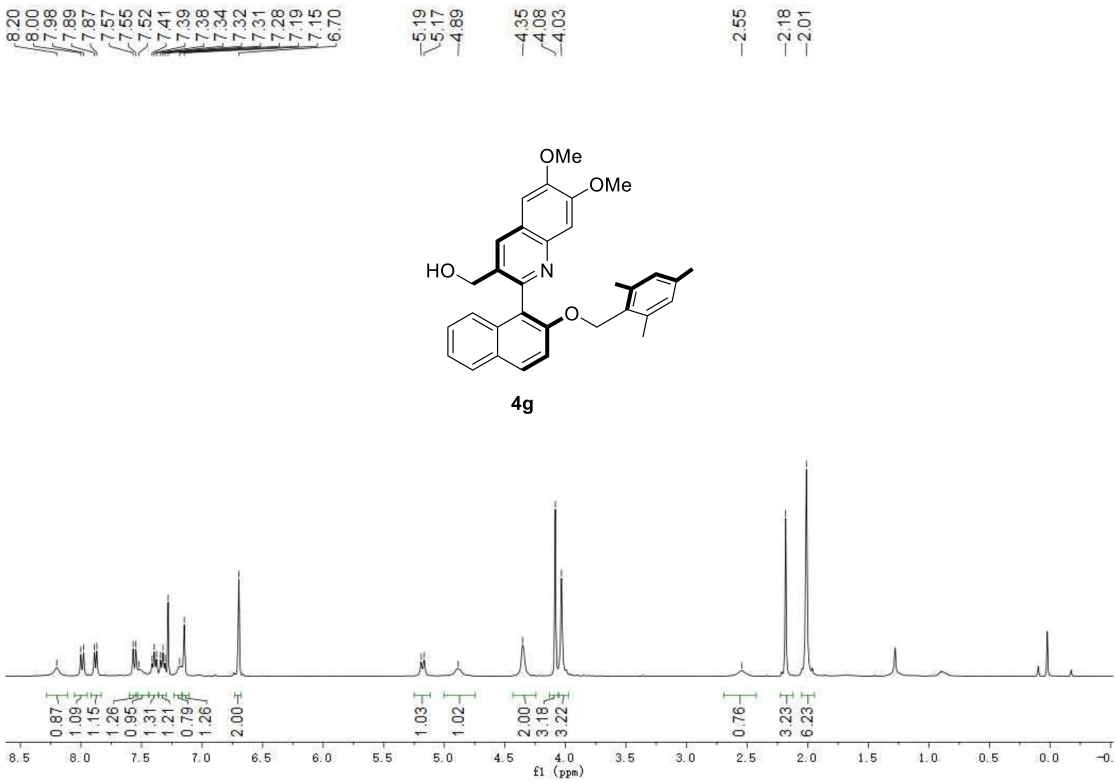

Supplementary Figure 94. ^{1}H NMR Spectra of **4g**

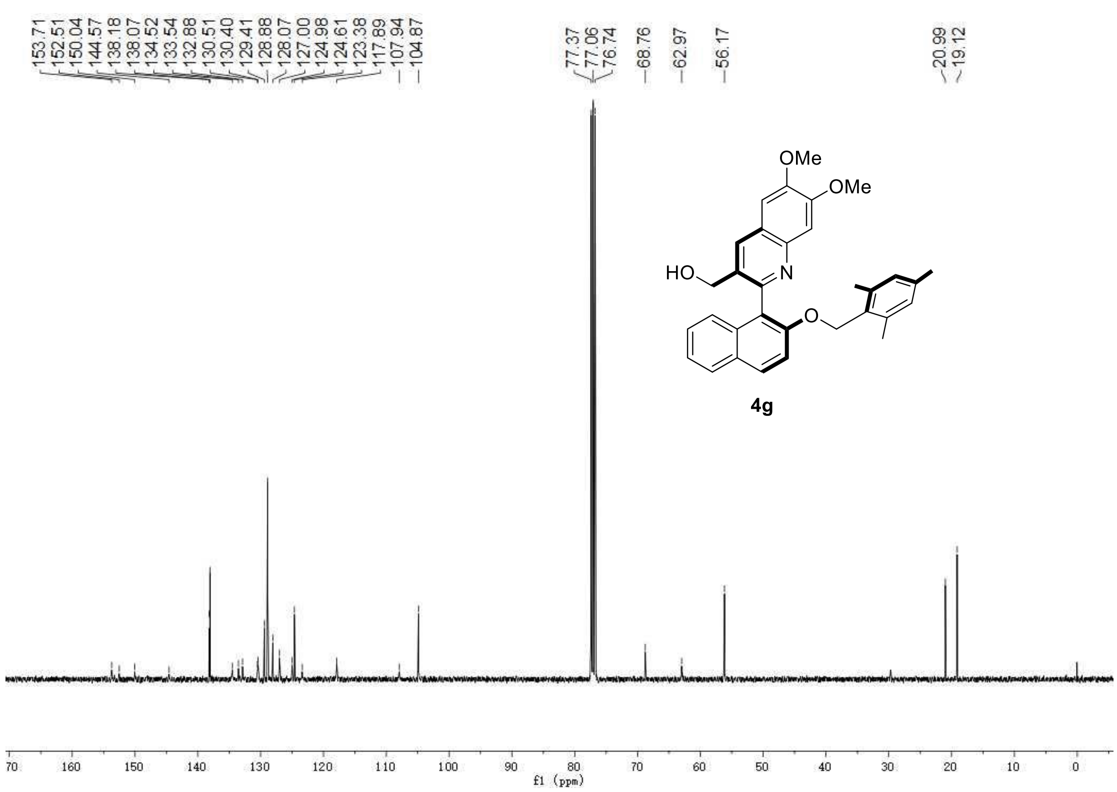

Supplementary Figure 95. ^{13}C NMR Spectra of **4g**

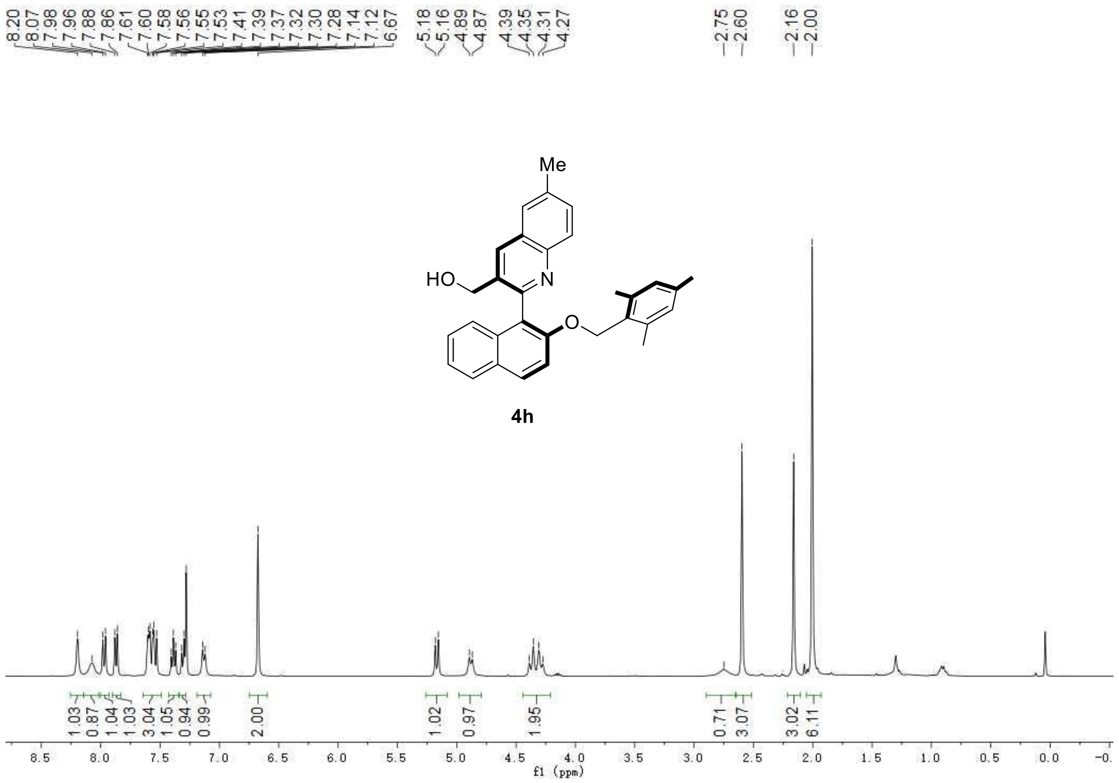

Supplementary Figure 96. ^{1}H NMR Spectra of **4h**

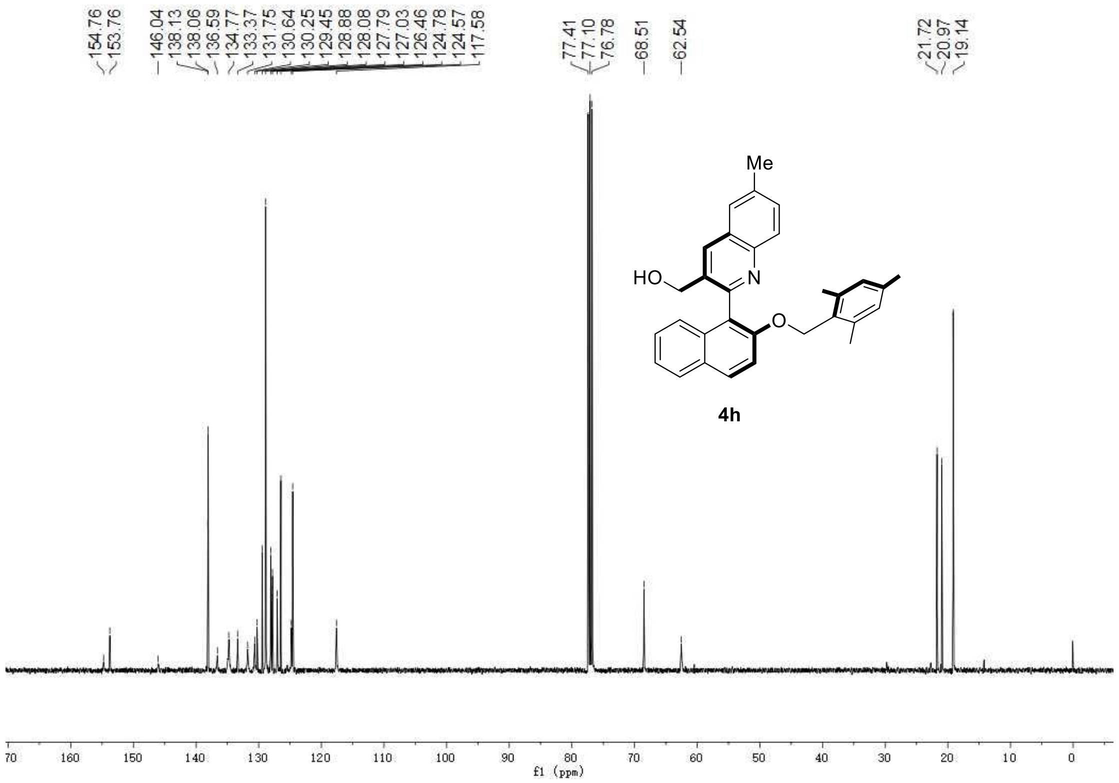

Supplementary Figure 97. ^{13}C NMR Spectra of **4h**

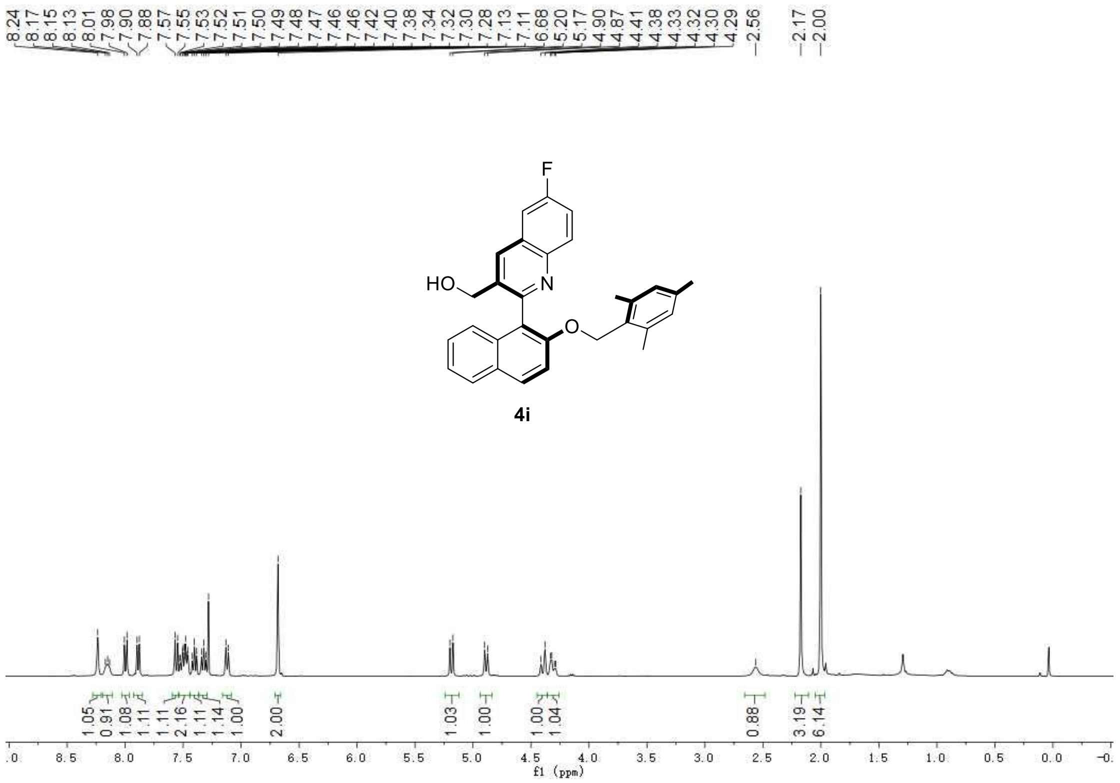

Supplementary Figure 98. ^{1}H NMR Spectra of **4i**

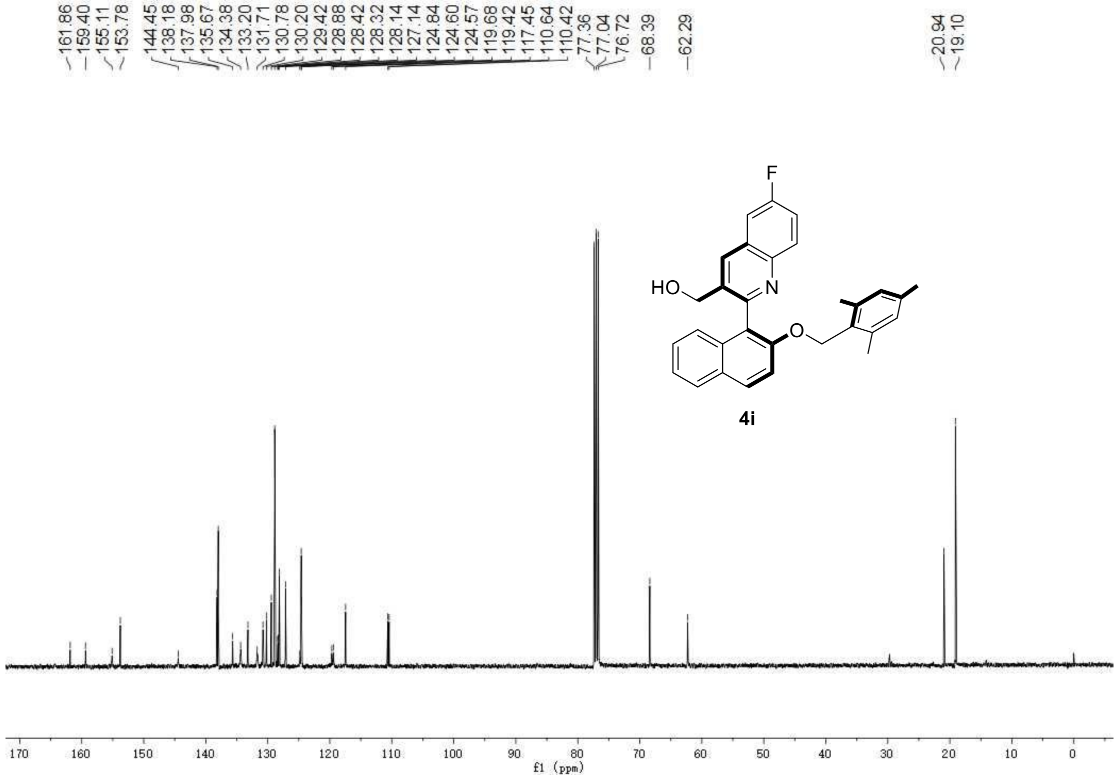

Supplementary Figure 99. ^{13}C NMR Spectra of **4i**

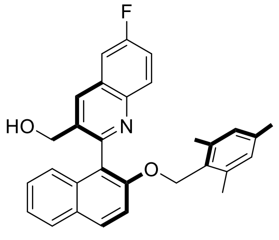

4i

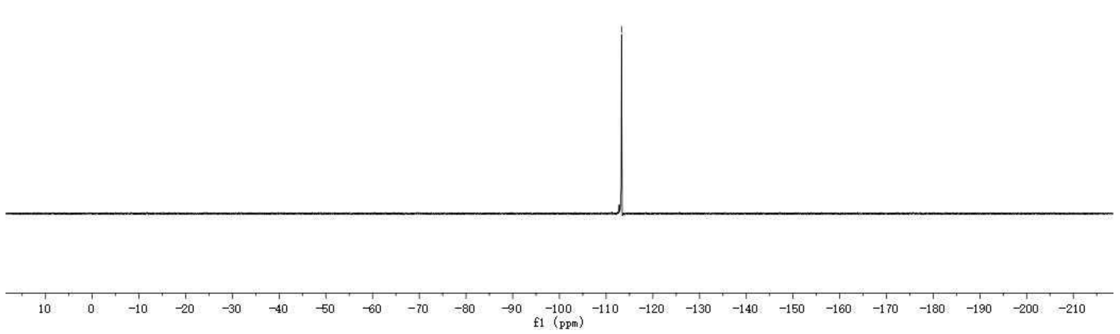

Supplementary Figure 100. ^{19}F NMR Spectra of **4i**

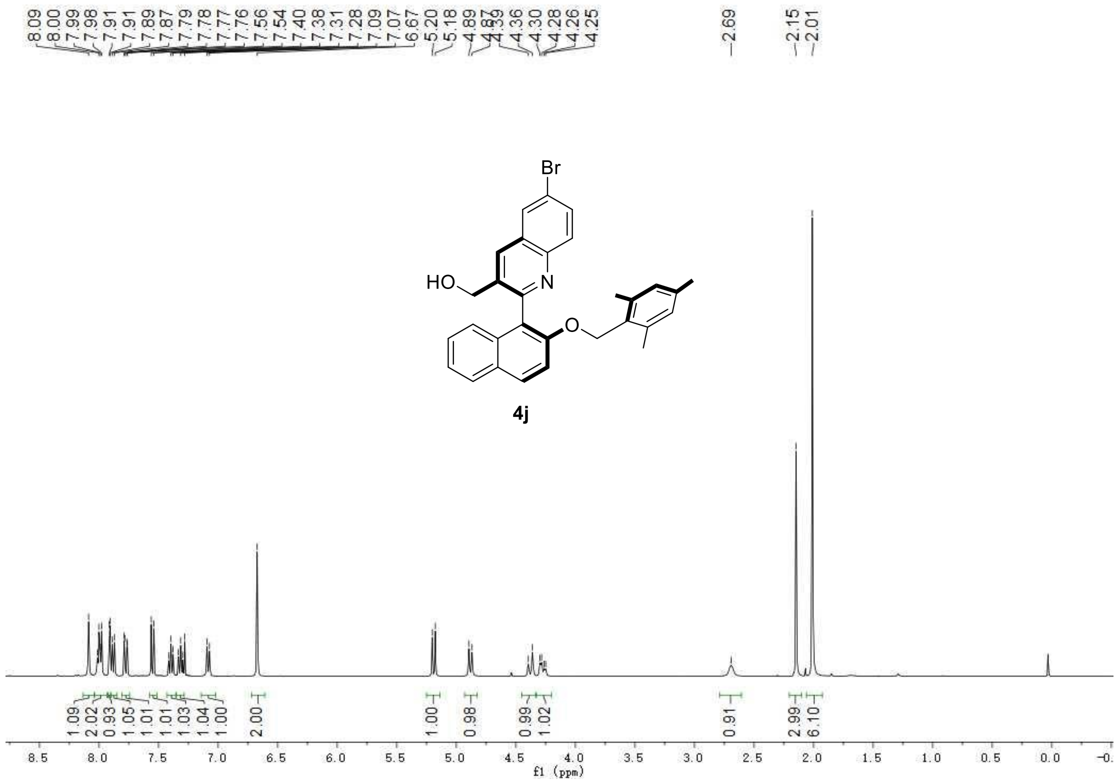

Supplementary Figure 101. ^{1}H NMR Spectra of **4j**

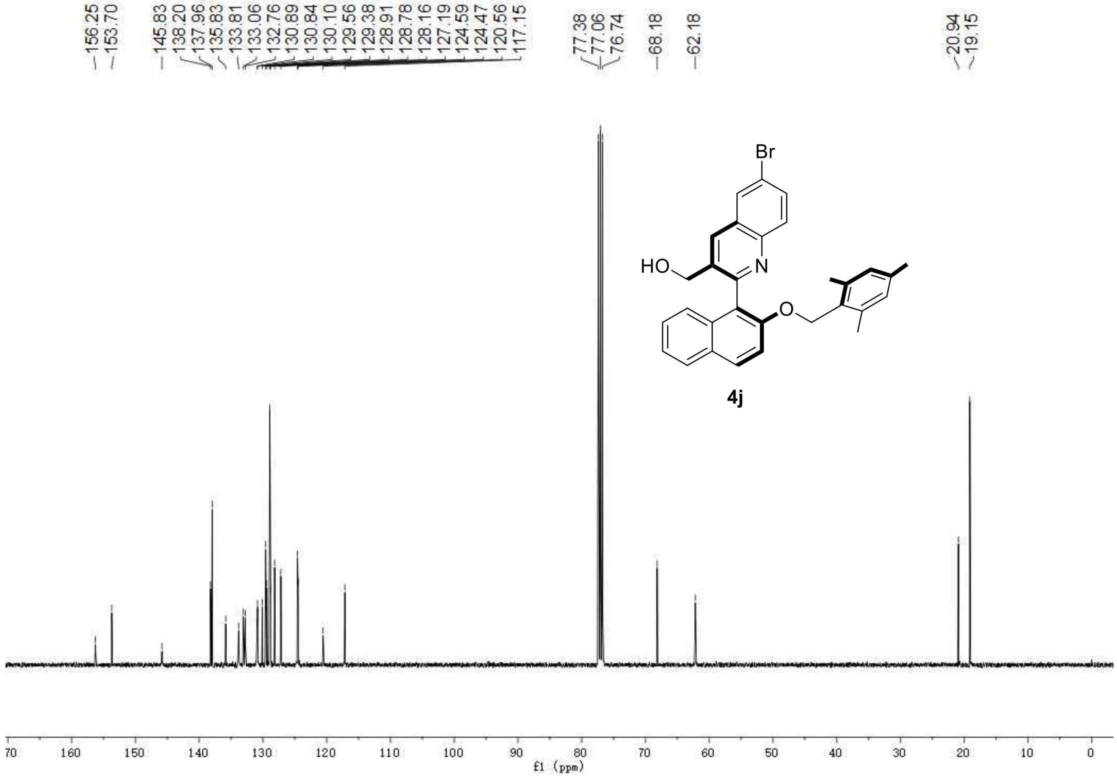

Supplementary Figure 102. ^{13}C NMR Spectra of **4j**

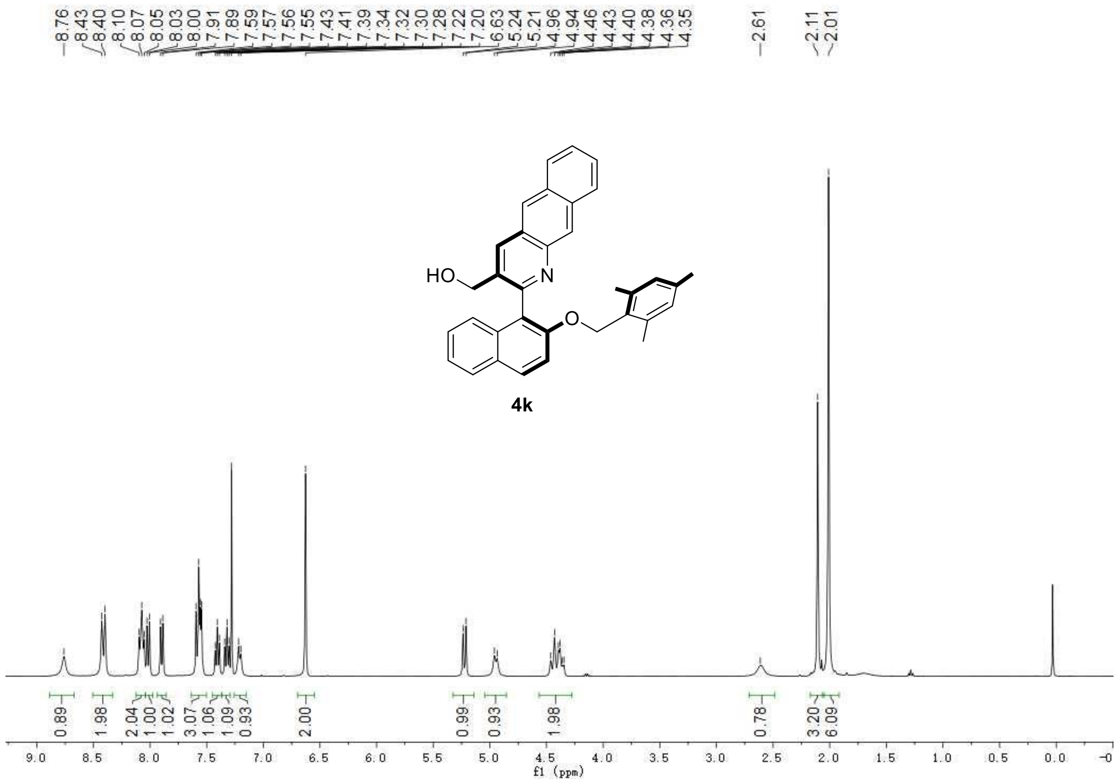

Supplementary Figure 103. ^{1}H NMR Spectra of **4k**

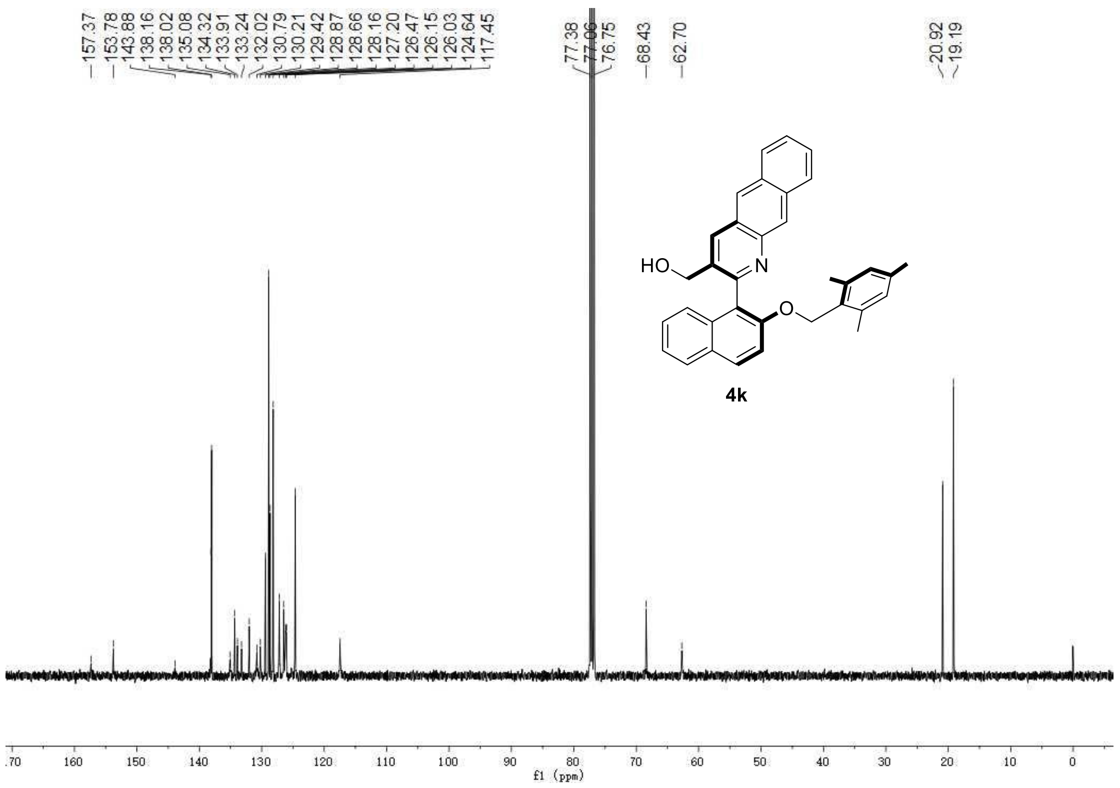

Supplementary Figure 104. ^{13}C NMR Spectra of **4k**

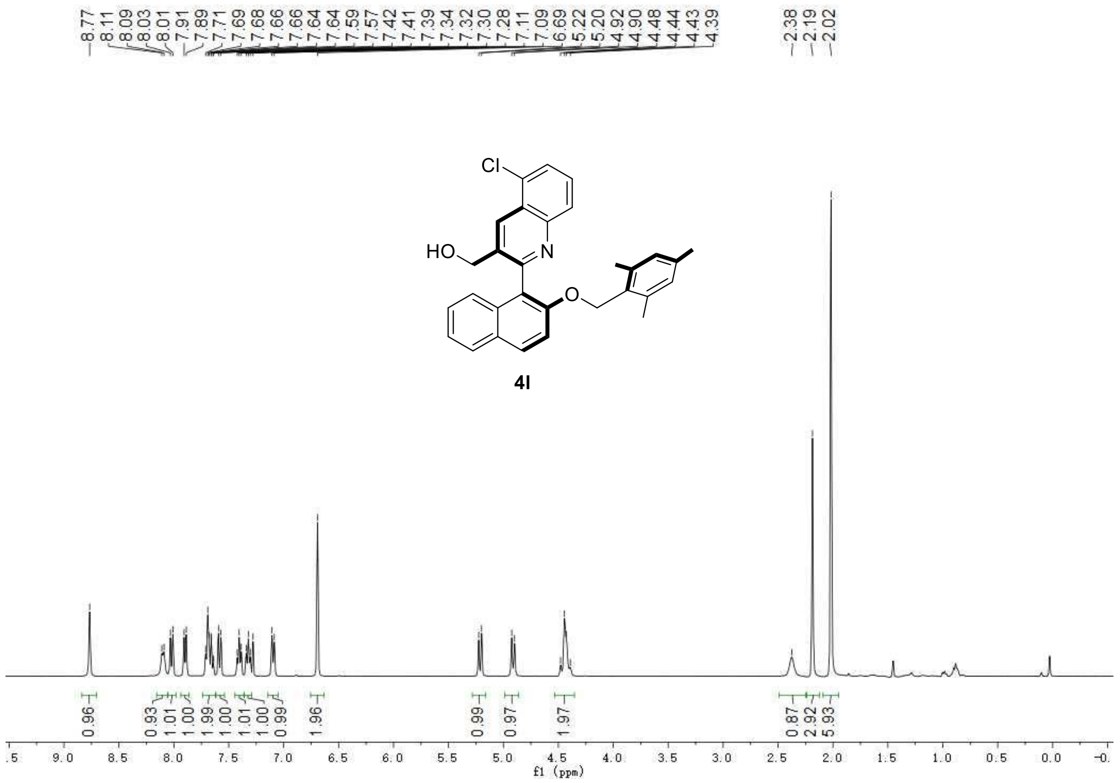

Supplementary Figure 105. ^{1}H NMR Spectra of **4l**

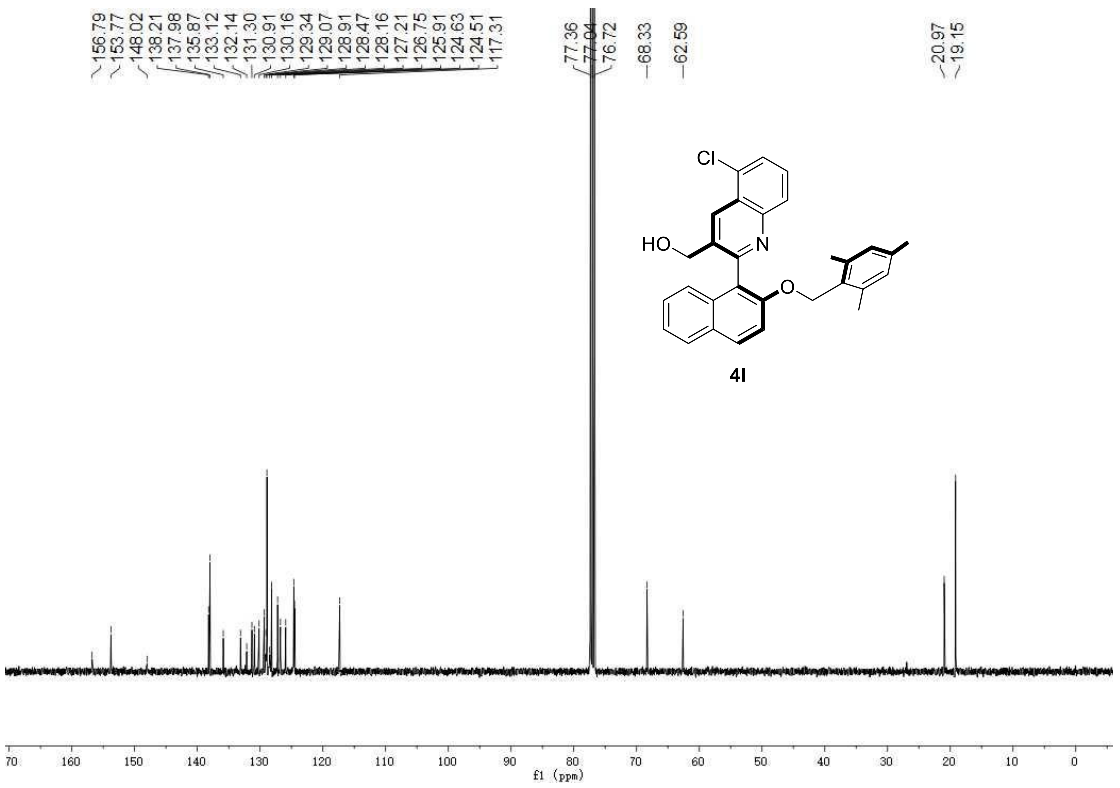

Supplementary Figure 106. ^{13}C NMR Spectra of **4l**

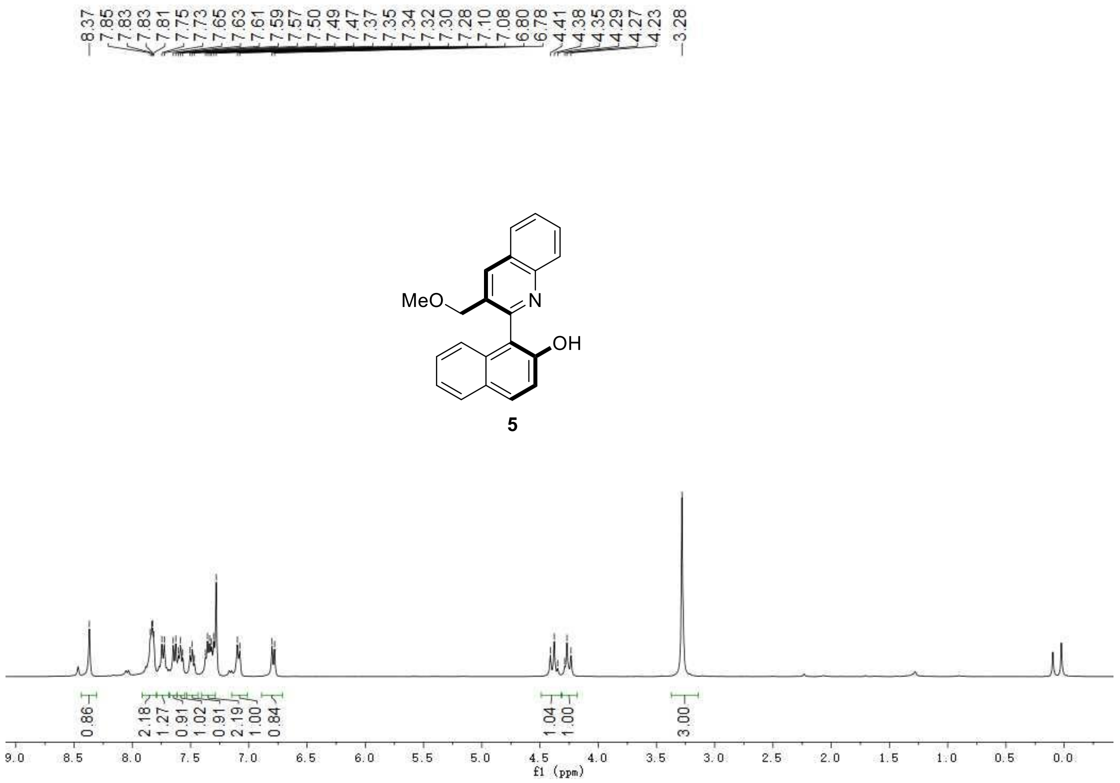

Supplementary Figure 107. ^{1}H NMR Spectra of **5**

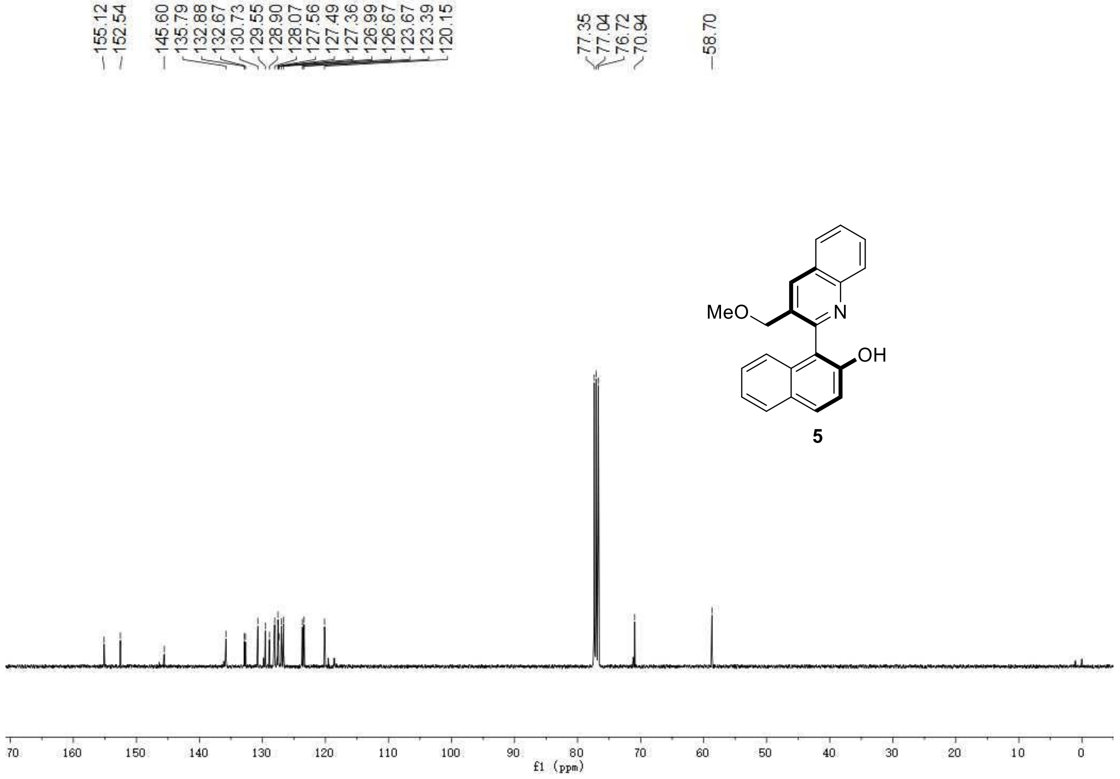

Supplementary Figure 108. ^{13}C NMR Spectra of **5**

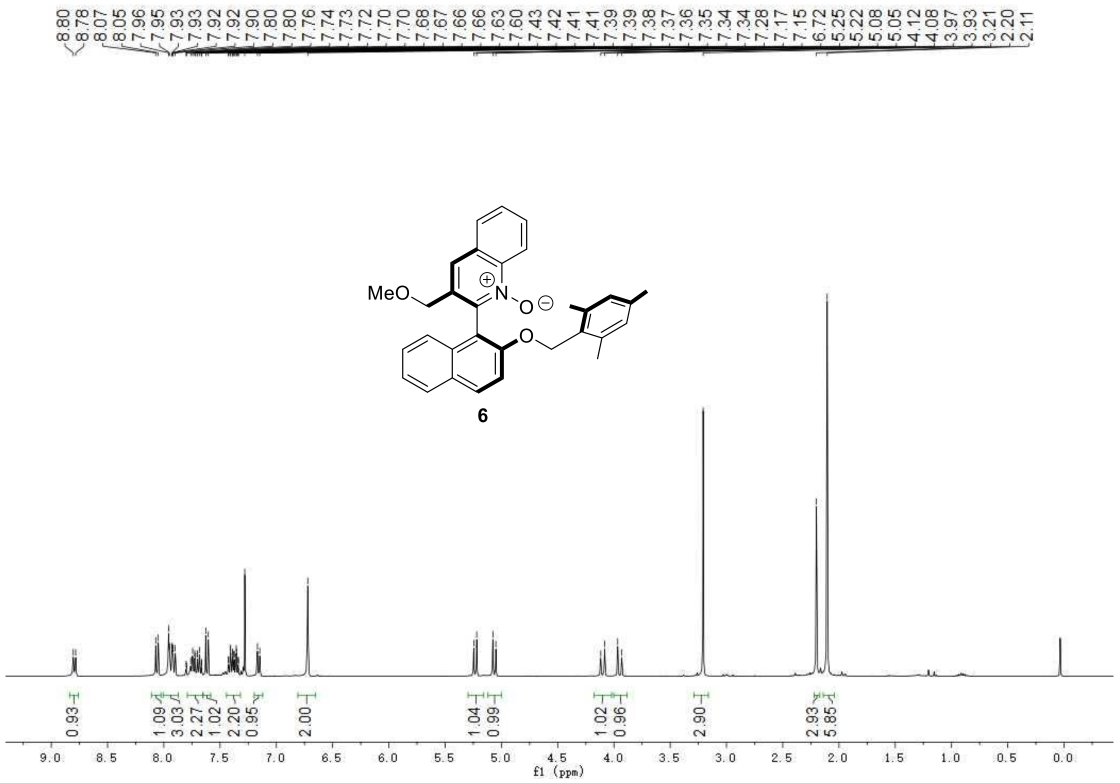

Supplementary Figure 109. ^{1}H NMR Spectra of **6**

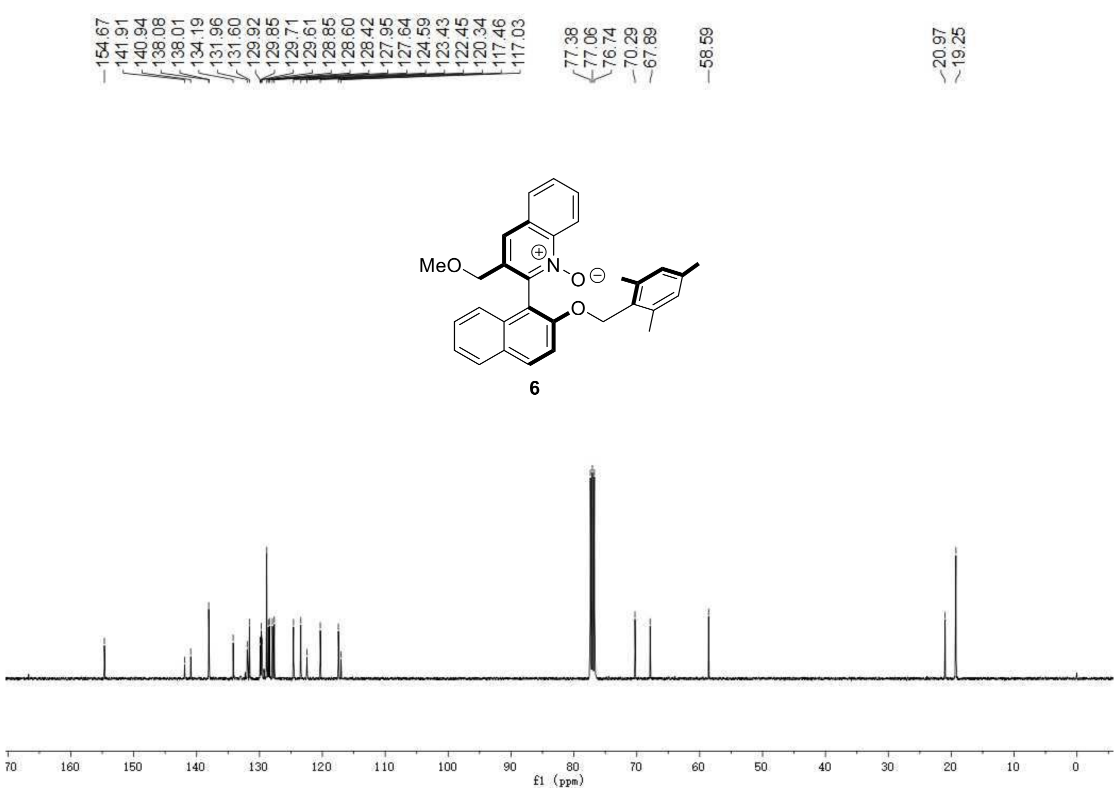

Supplementary Figure 110. ^{13}C NMR Spectra of **6**

HPLC Spectra

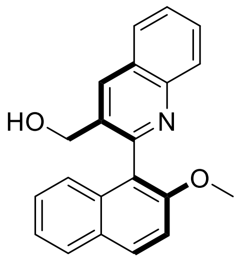

3a

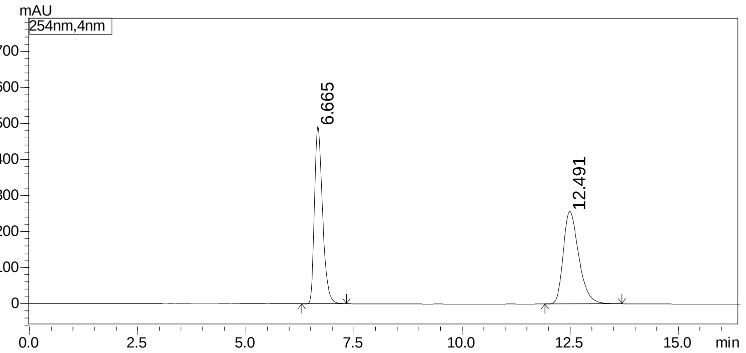

Peak	Ret. time	Area	Height	Area%	Height%
1	6.665	6394680	493226	49.941	65.716
2	12.491	6409810	257316	50.059	34.284
Total		12804490	750542	100.000	100.000

Supplementary Figure 111. HPLC spectrum of *racemic* **3a**

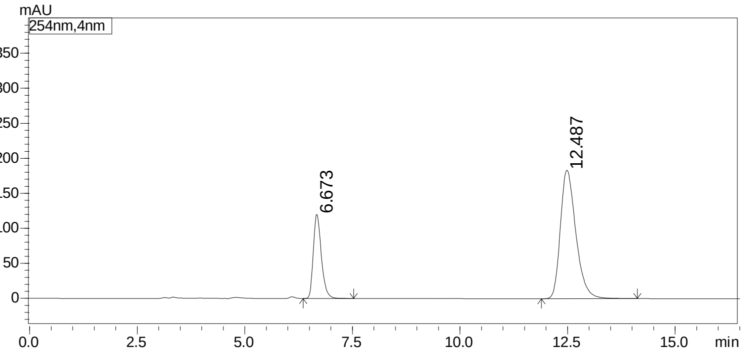

Peak	Ret. time	Area	Height	Area%	Height%
1	6.673	1565546	120083	25.443	39.571
2	12.487	4587680	183377	74.557	60.429
Total		6153226	303460	100.000	100.000

Supplementary Figure 112. HPLC spectrum of **3a**

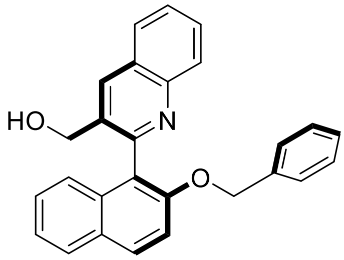

3b

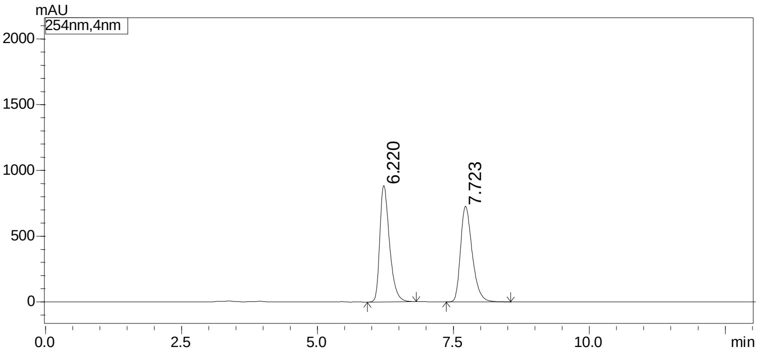

Peak	Ret. time	Area	Height	Area%	Height%
1	6.220	11000741	886164	50.279	54.917
2	7.723	10878572	727481	49.721	45.083
Total		21879312	1613645	100.000	100.000

Supplementary Figure 113. HPLC spectrum of *racemic* **3b**

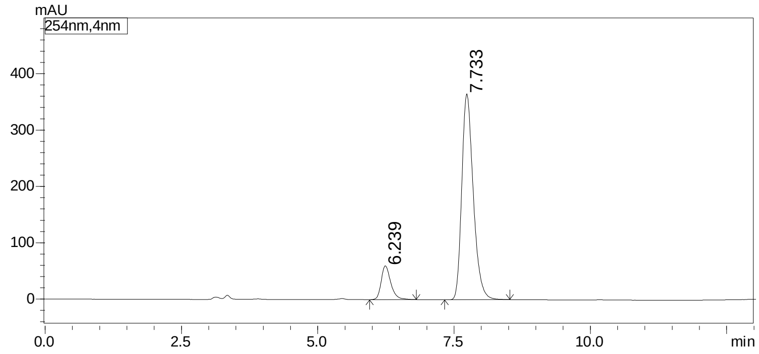

Peak	Ret. time	Area	Height	Area%	Height%
1	6.239	739808	60095	12.137	14.128
2	7.733	5355614	365261	87.863	85.872
Total		6095422	425356	100.000	100.000

Supplementary Figure 114. HPLC spectrum of **3b**

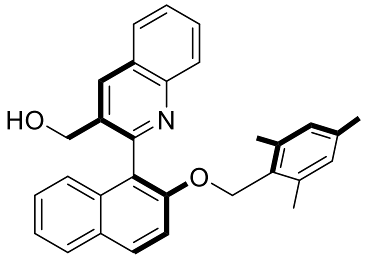

3c

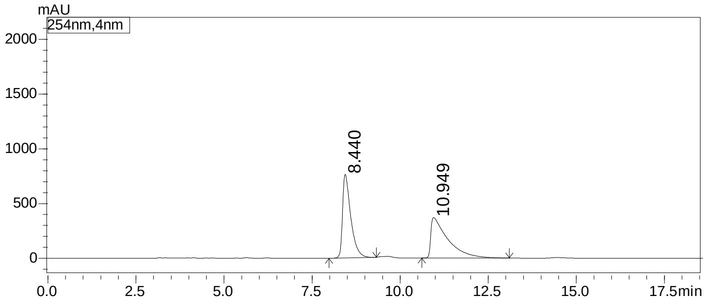

Peak	Ret. time	Area	Height	Area%	Height%
1	8.440	12368141	762787	49.840	67.266
2	10.949	12447789	371197	50.160	32.734
总计		24815930	1133985	100.000	100.000

Supplementary Figure 115. HPLC spectrum of *racemic* **3c**

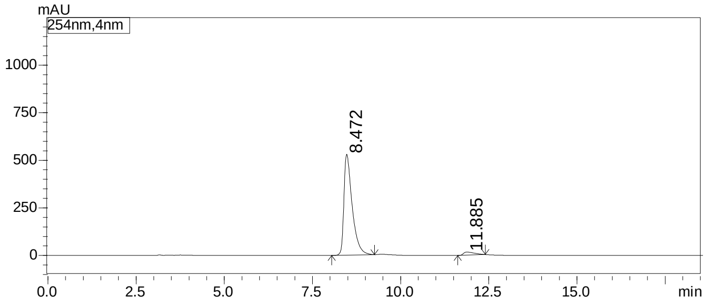

Peak	Ret. time	Area	Height	Area%	Height%
1	8.472	8617378	530514	96.075	97.046
2	11.885	352083	16150	3.925	2.954
总计		8969461	546665	100.000	100.000

Supplementary Figure 116. HPLC spectrum of **3c**

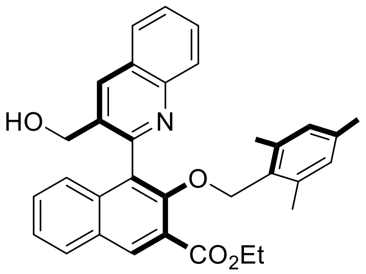

3d

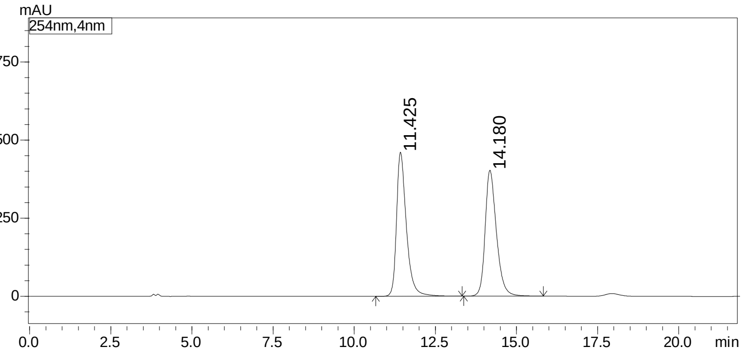

Peak	Ret. time	Area	Height	Area%	Height%
1	11.425	9740530	461364	50.004	53.375
2	14.180	9739164	403024	49.996	46.625
Total		19479694	864389	100.000	100.000

Supplementary Figure 117. HPLC spectrum of *racemic* **3d**

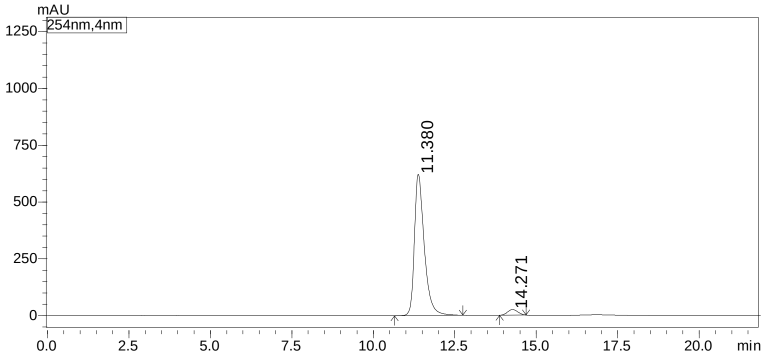

Peak	Ret. time	Area	Height	Area%	Height%
1	11.380	13090472	622234	95.973	96.136
2	14.271	549341	25008	4.027	3.864
Total		13639813	647242	100.000	100.000

Supplementary Figure 118. HPLC spectrum of **3d**

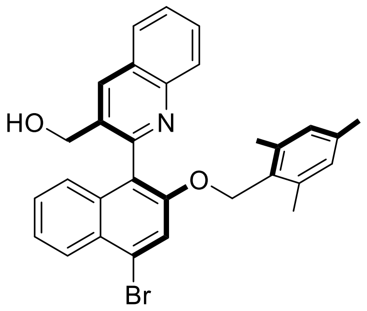

3e

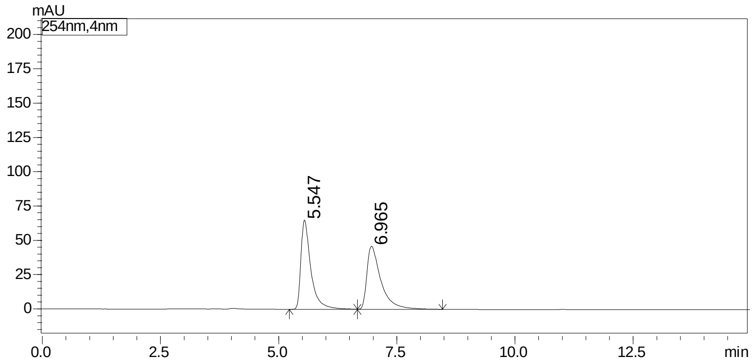

Peak	Ret. time	Area	Height	Area%	Height%
1	5.547	955448	65116	50.141	58.609
2	6.965	950084	45986	49.859	41.391
Total		1905532	111102	100.000	100.000

Supplementary Figure 119. HPLC spectrum of *racemic* **3e**

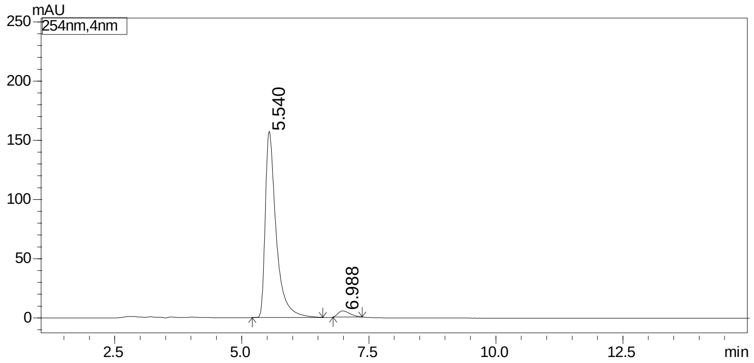

Peak	Ret. time	Area	Height	Area%	Height%
1	5.540	2342428	157181	96.512	96.828
2	6.988	84667	5150	3.488	3.172
Total		2427096	162330	100.000	100.000

Supplementary Figure 120. HPLC spectrum of **3e**

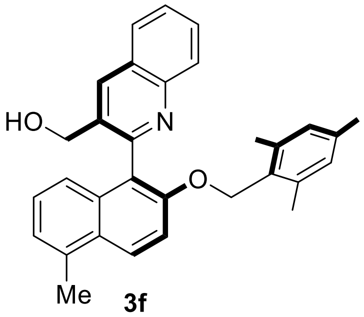

3f

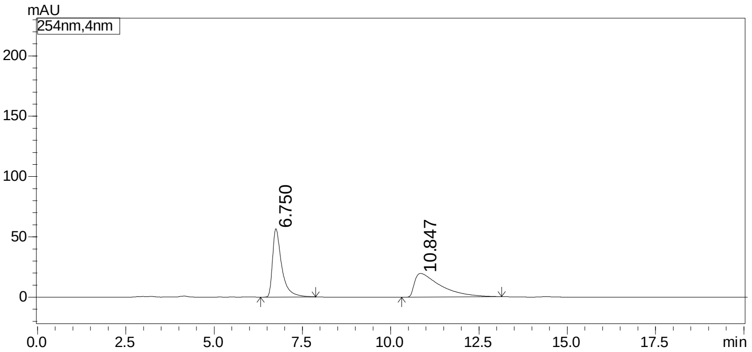

Peak	Ret. time	Area	Height	Area%	Height%
1	6.750	1025056	56579	50.452	74.259
2	10.847	1006696	19612	49.548	25.741
Total		2031752	76191	100.000	100.000

Supplementary Figure 121. HPLC spectrum of *racemic* **3f**

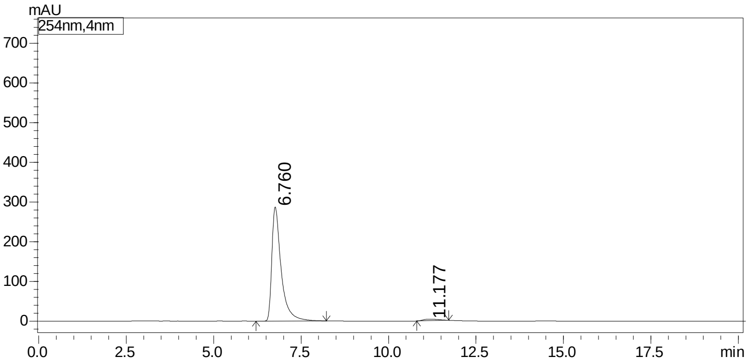

Peak	Ret. time	Area	Height	Area%	Height%
1	6.760	5338859	287748	97.781	98.644
2	11.177	121156	3955	2.219	1.356
Total		5460015	291702	100.000	100.000

Supplementary Figure 122. HPLC spectrum of **3f**

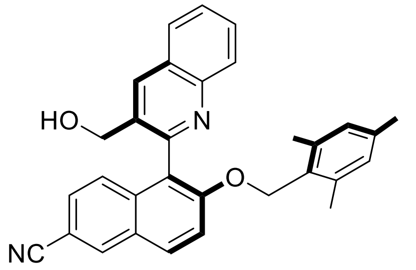

3g

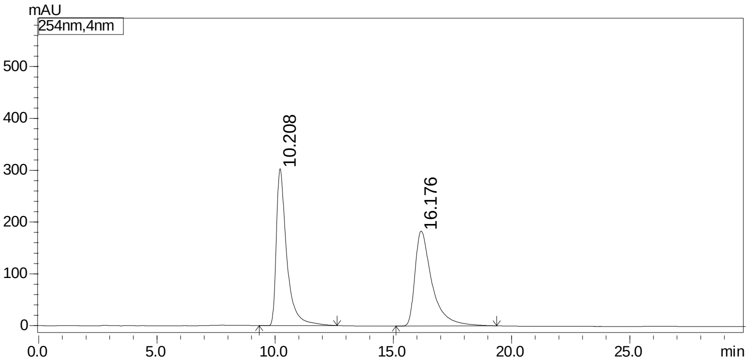

Peak	Ret. time	Area	Height	Area%	Height%
1	10.208	9274157	302587	50.228	62.268
2	16.176	9189840	183355	49.772	37.732
Total		18463997	485942	100.000	100.000

Supplementary Figure 123. HPLC spectrum of *racemic* **3g**

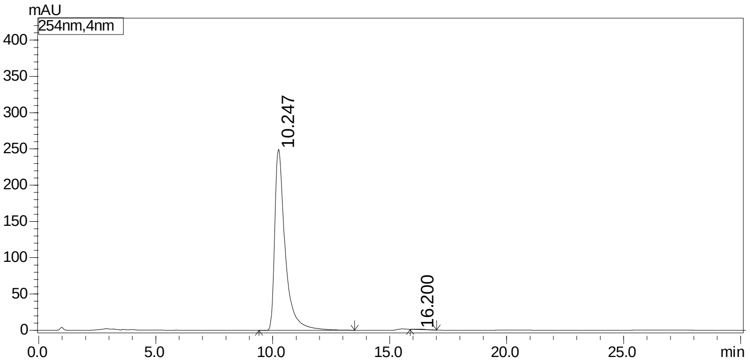

Peak	Ret. time	Area	Height	Area%	Height%
1	10.247	7763038	249672	99.721	99.730
2	16.200	21736	677	0.279	0.270
Total		7784774	250349	100.000	100.000

Supplementary Figure 124. HPLC spectrum of **3g**

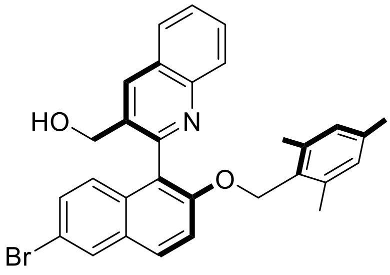

3h

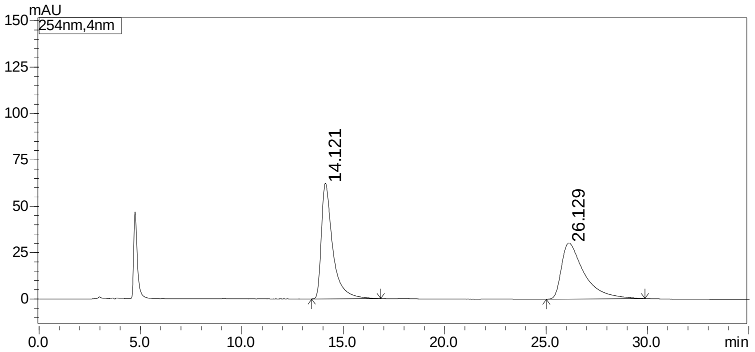

Peak	Ret. time	Area	Height	Area%	Height%
1	14.121	2425845	62356	50.766	67.362
2	26.129	2352673	30213	49.234	32.638
Total		4778517	92570	100.000	100.000

Supplementary Figure 125. HPLC spectrum of *racemic* **3h**

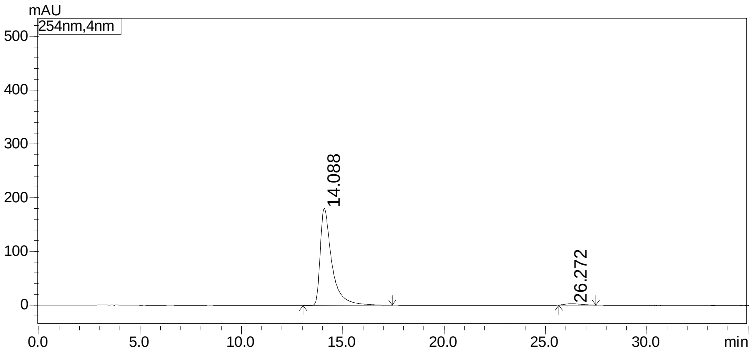

Peak	Ret. time	Area	Height	Area%	Height%
1	14.088	7179507	180509	98.007	98.583
2	26.272	146007	2595	1.993	1.417
Total		7325514	183104	100.000	100.000

Supplementary Figure 126. HPLC spectrum of **3h**

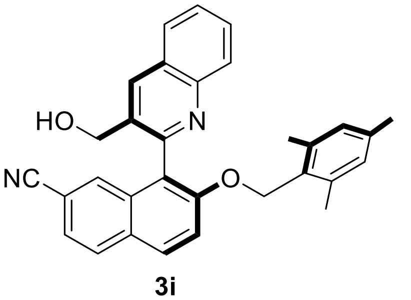

3i

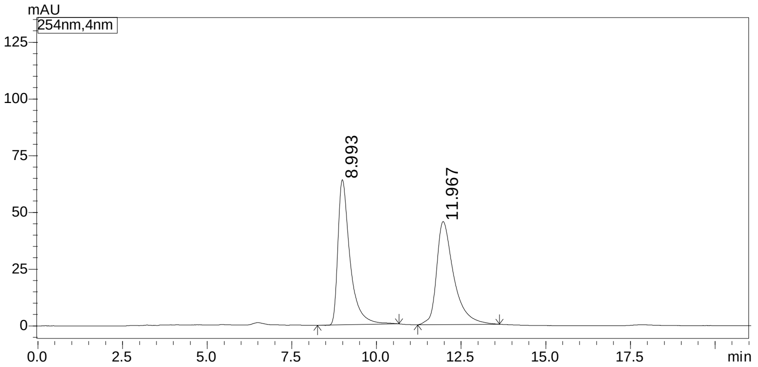

Peak	Ret. time	Area	Height	Area%	Height%
1	8.993	1585276	63994	49.708	58.473
2	11.967	1603871	45448	50.292	41.527
Total		3189147	109443	100.000	100.000

Supplementary Figure 127. HPLC spectrum of *racemic* **3i**

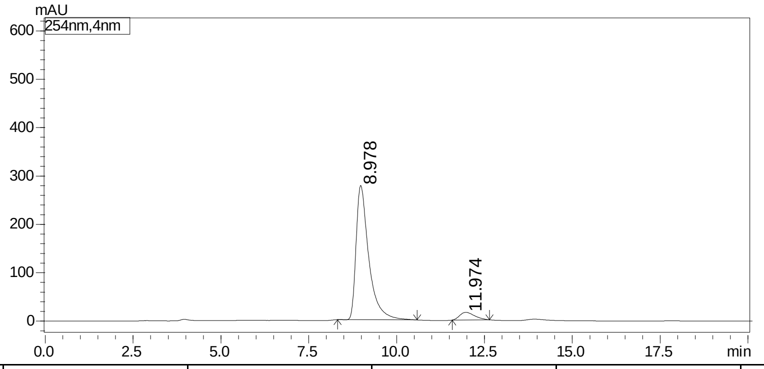

Peak	Ret. time	Area	Height	Area%	Height%
1	8.978	6981752	277596	93.793	94.619
2	11.974	462035	15787	6.207	5.381
Total		7443787	293383	100.000	100.000

Supplementary Figure 128. HPLC spectrum of **3i**

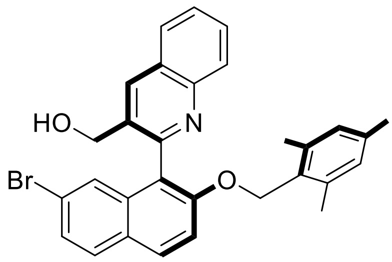

3j

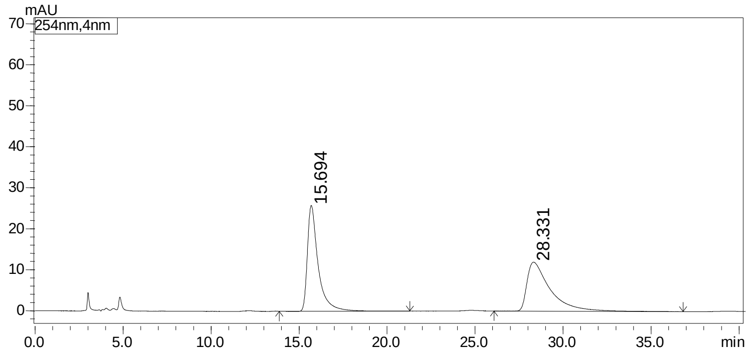

Peak	Ret. time	Area	Height	Area%	Height%
1	15.694	1093145	25810	49.974	68.329
2	28.331	1094285	11963	50.026	31.671
Total		2187431	37773	100.000	100.000

Supplementary Figure 129. HPLC spectrum of *racemic* **3j**

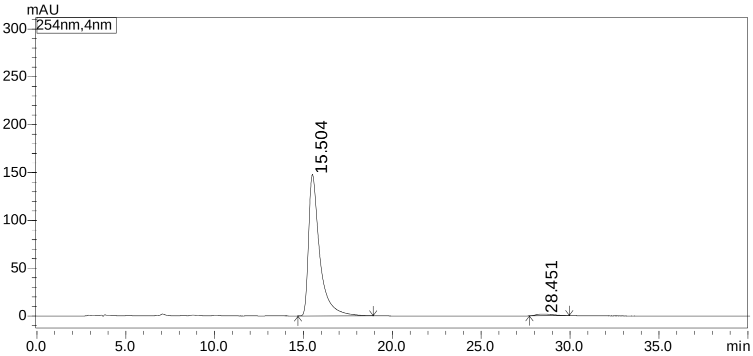

Peak	Ret. time	Area	Height	Area%	Height%
1	15.504	6418191	147689	98.165	98.796
2	28.451	119976	1799	1.835	1.204
Total		6538167	149489	100.000	100.000

Supplementary Figure 130. HPLC spectrum of **3j**

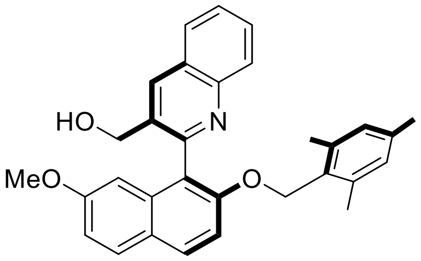

3k

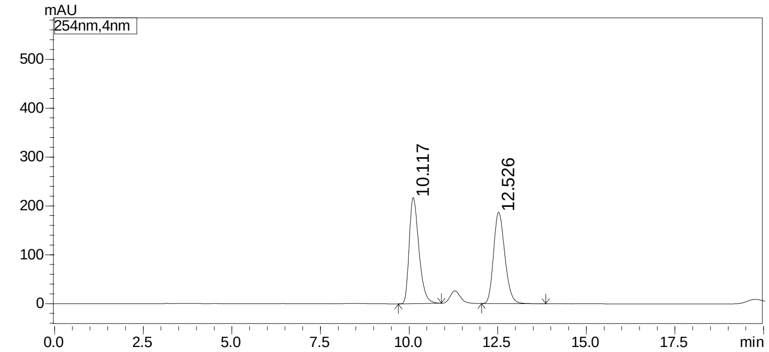

Peak	Ret. time	Area	Height	Area%	Height%
1	10.117	4129947	216745	49.912	53.676
2	12.526	4144527	187060	50.088	46.324
Total		8274474	403804	100.000	100.000

Supplementary Figure 131. HPLC spectrum of *racemic* **3k**

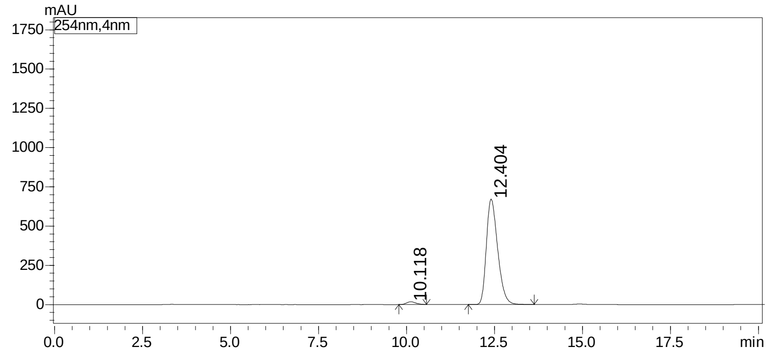

Peak	Ret. time	Area	Height	Area%	Height%
1	10.118	331144	17690	2.159	2.566
2	12.404	15005459	671594	97.841	97.434
Total		15336602	689284	100.000	100.000

Supplementary Figure 132. HPLC spectrum of **3k**

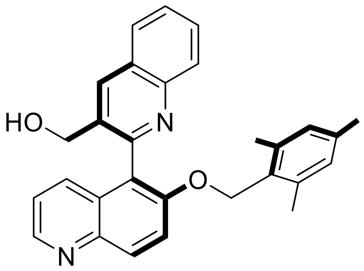

3l

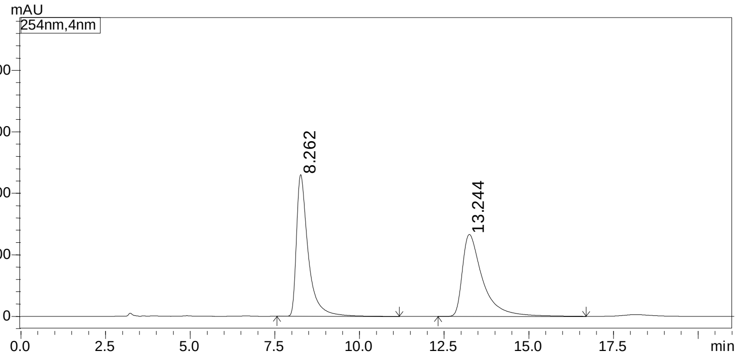

Peak	Ret. time	Area	Height	Area%	Height%
1	8.262	5536249	230104	49.949	63.335
2	13.244	5547648	133207	50.051	36.665
Total		11083897	363310	100.000	100.000

Supplementary Figure 133. HPLC spectrum of *racemic* **3l**

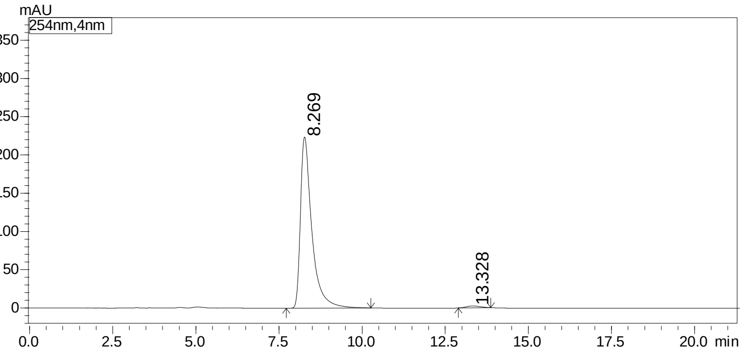

Peak	Ret. time	Area	Height	Area%	Height%
1	8.269	5337935	223772	98.670	98.930
2	13.328	71933	2419	1.330	1.070
Total		5409868	226191	100.000	100.000

Supplementary Figure 134. HPLC spectrum of **3l**

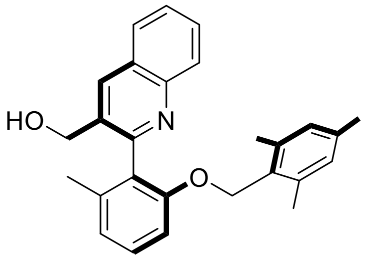

3m

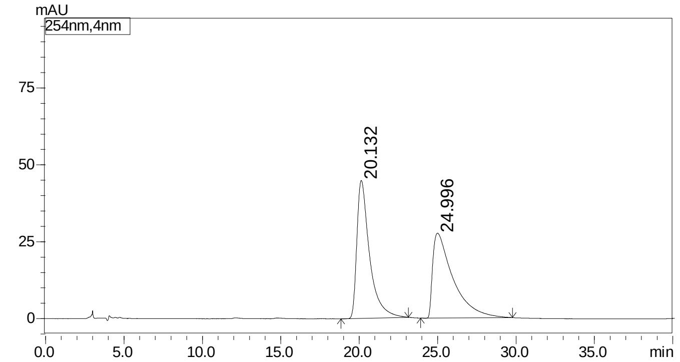

Peak	Ret. time	Area	Height	Area%	Height%
1	20.132	2522682	44859	50.311	61.915
2	24.996	2491469	27593	49.689	38.085
Total		5014151	72451	100.000	100.000

Supplementary Figure 135. HPLC spectrum of *racemic* **3m**

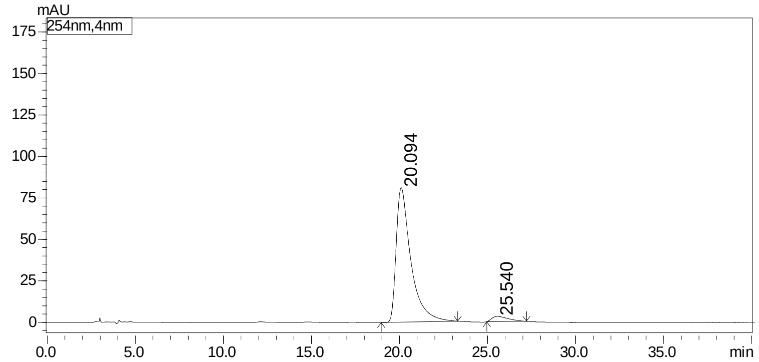

Peak	Ret. time	Area	Height	Area%	Height%
1	20.094	4689477	81023	95.839	96.297
2	25.540	203606	3116	4.161	3.703
Total		4893082	84138	100.000	100.000

Supplementary Figure 136. HPLC spectrum of **3m**

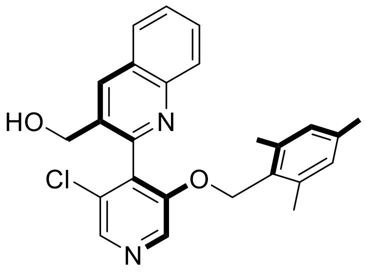

3n

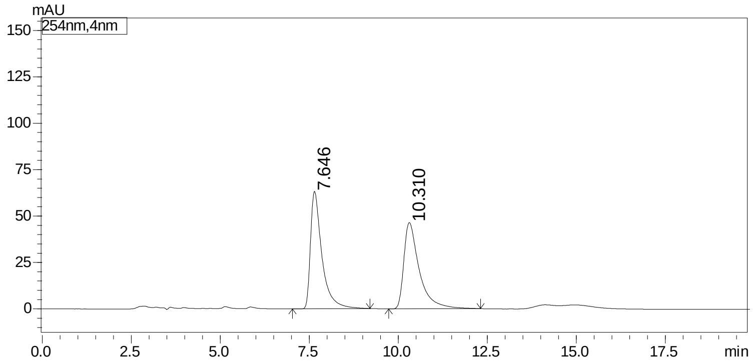

Peak	Ret. time	Area	Height	Area%	Height%
1	7.646	1412860	63240	50.197	57.663
2	10.310	1401795	46431	49.803	42.337
Total		2814655	109671	100.000	100.000

Supplementary Figure 137. HPLC spectrum of *racemic* **3n**

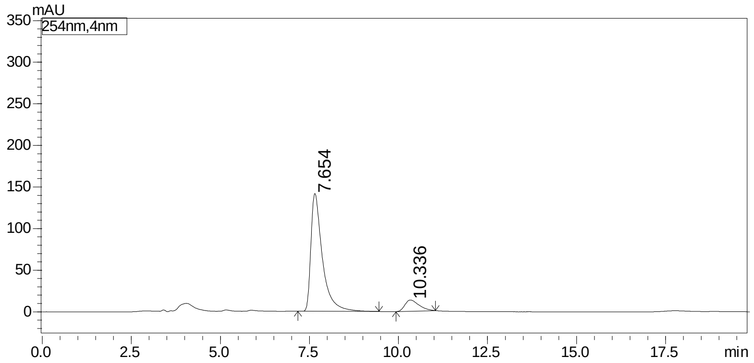

Peak	Ret. time	Area	Height	Area%	Height%
1	7.654	3179259	141445	89.514	91.284
2	10.336	372441	13505	10.486	8.716
Total		3551700	154951	100.000	100.000

Supplementary Figure 138. HPLC spectrum of **3n**

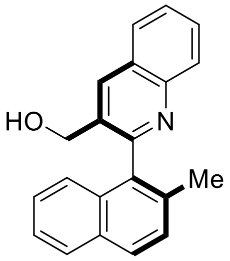

3o

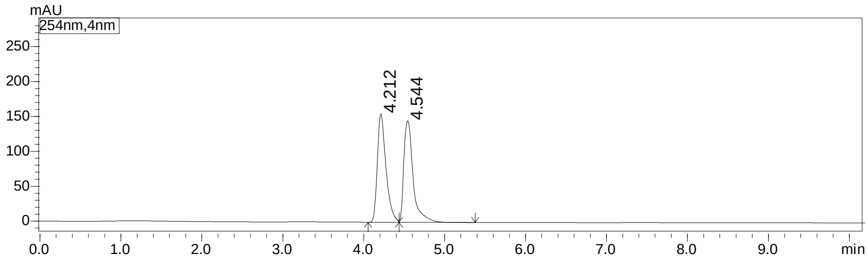

Peak	Ret. time	Area	Height	Area%	Height%
1	4.212	1153564	155494	49.731	51.644
2	4.544	1166032	145596	50.269	48.356
Total		2319595	301090	100.000	100.000

Supplementary Figure 139. HPLC spectrum of *racemic* **3o**

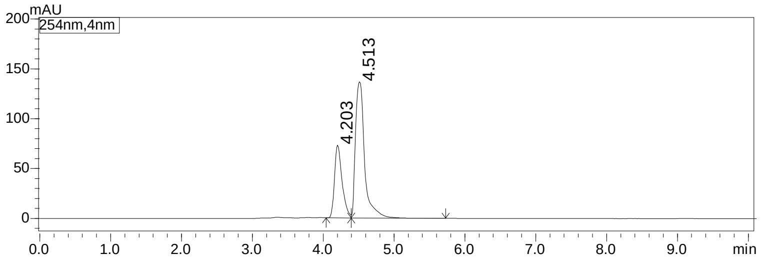

Peak	Ret. time	Area	Height	Area%	Height%
1	4.203	542290	72931	30.865	34.809
2	4.513	1214685	136586	69.135	65.191
Total		1756975	209517	100.000	100.000

Supplementary Figure 140. HPLC spectrum of **3o**

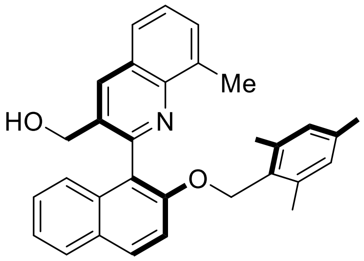

4a

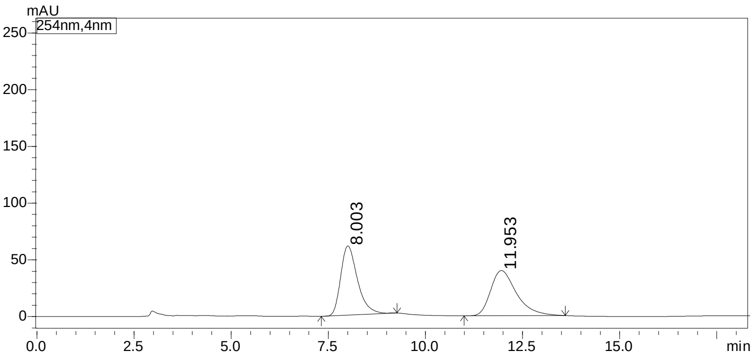

Peak	Ret. time	Area	Height	Area%	Height%
1	8.003	1835097	61057	49.803	60.496
2	11.953	1849626	39871	50.197	39.504
Total		3684723	100927	100.000	100.000

Supplementary Figure 141. HPLC spectrum of *racemic* **4a**

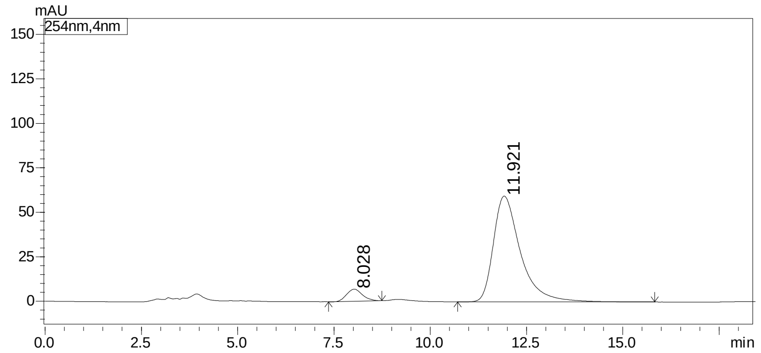

Peak	Ret. time	Area	Height	Area%	Height%
1	8.028	194374	6848	6.246	10.320
2	11.921	2917777	59514	93.754	89.680
Total		3112151	66362	100.000	100.000

Supplementary Figure 142. HPLC spectrum of **4a**

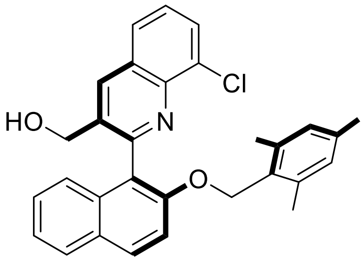

4b

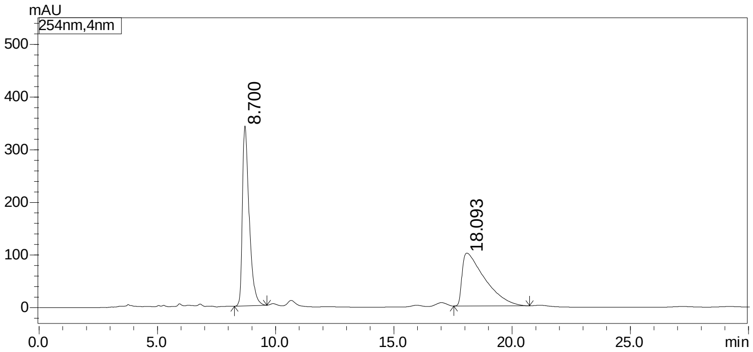

Peak	Ret. time	Area	Height	Area%	Height%
1	8.700	6843316	342001	50.468	77.278
2	18.093	6716439	100559	49.532	22.722
Total		13559754	442560	100.000	100.000

Supplementary Figure 143. HPLC spectrum of *racemic* **4b**

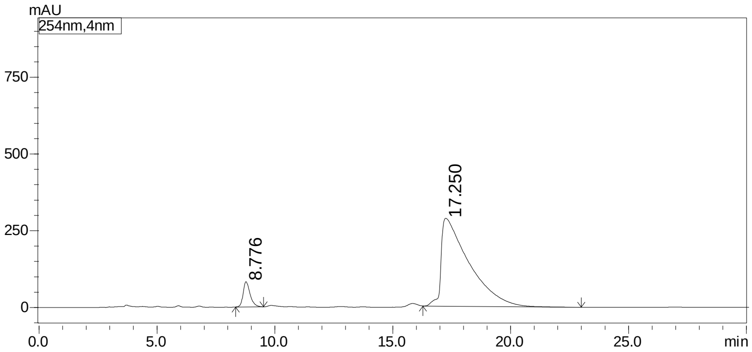

Peak	Ret. time	Area	Height	Area%	Height%
1	8.776	1691801	82127	6.663	22.279
2	17.250	23699736	286496	93.337	77.721
Total		25391537	368623	100.000	100.000

Supplementary Figure 144. HPLC spectrum of **4b**

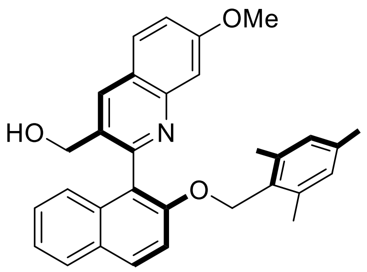

4c

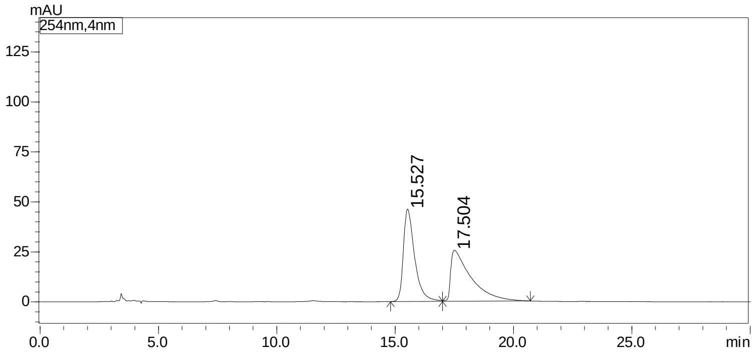

Peak	Ret. time	Area	Height	Area%	Height%
1	15.527	1537388	46239	50.231	64.390
2	17.504	1523253	25572	49.769	35.610
Total		3060640	71811	100.000	100.000

Supplementary Figure 145. HPLC spectrum of *racemic* **4c**

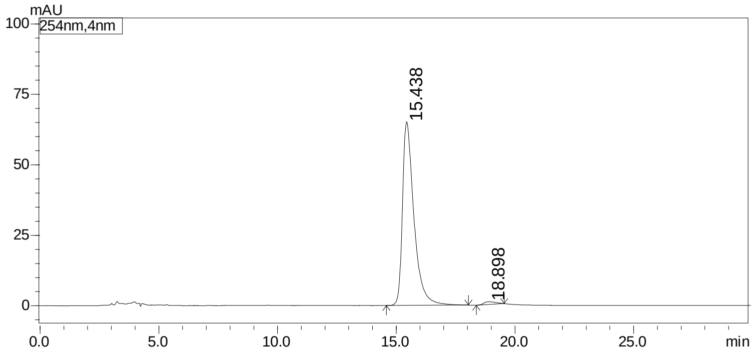

Peak	Ret. time	Area	Height	Area%	Height%
1	15.438	2191077	65115	98.459	98.513
2	18.898	34286	983	1.541	1.487
Total		2225363	66098	100.000	100.000

Supplementary Figure 146. HPLC spectrum of **4c**

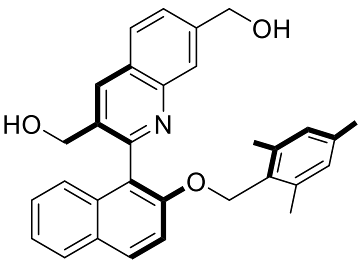

4d

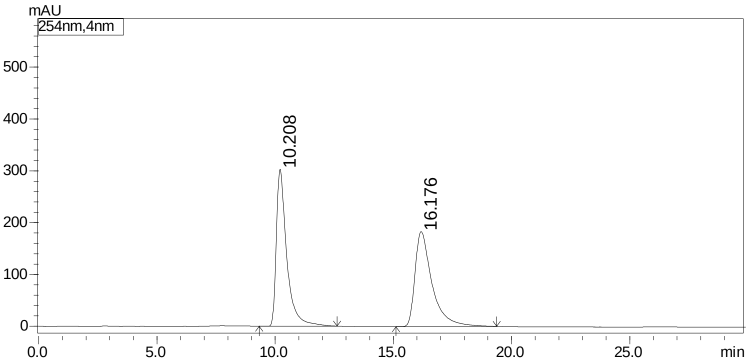

Peak	Ret. time	Area	Height	Area%	Height%
1	10.208	9274157	302587	50.228	62.268
2	16.176	9189840	183355	49.772	37.732
Total		18463997	485942	100.000	100.000

Supplementary Figure 147. HPLC spectrum of *racemic* **4d**

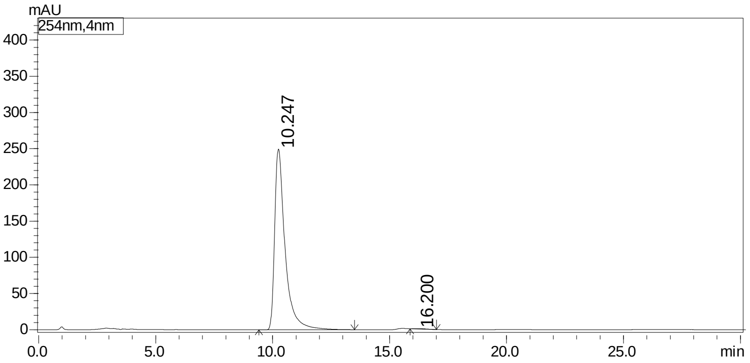

Peak	Ret. time	Area	Height	Area%	Height%
1	10.247	7763038	249672	99.721	99.730
2	16.200	21736	677	0.279	0.270
Total		7784774	250349	100.000	100.000

Supplementary Figure 148. HPLC spectrum of **4d**

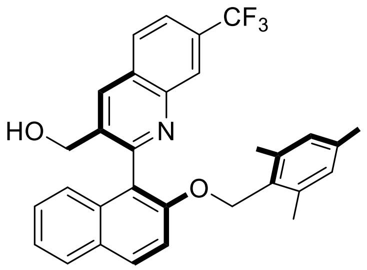

4e

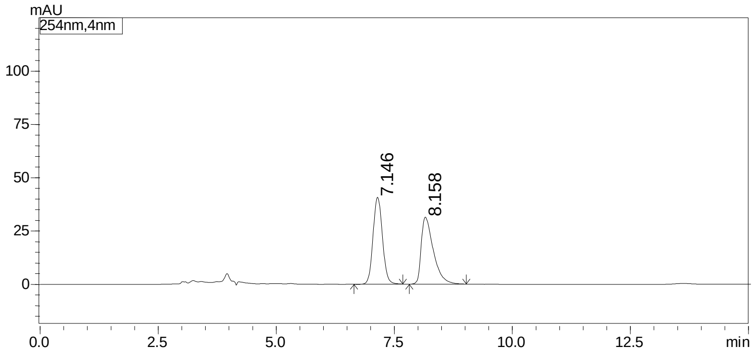

Peak	Ret. time	Area	Height	Area%	Height%
1	7.146	566945	40794	50.145	56.446
2	8.158	563671	31477	49.855	43.554
Total		1130617	72270	100.000	100.000

Supplementary Figure 149. HPLC spectrum of *racemic* **4e**

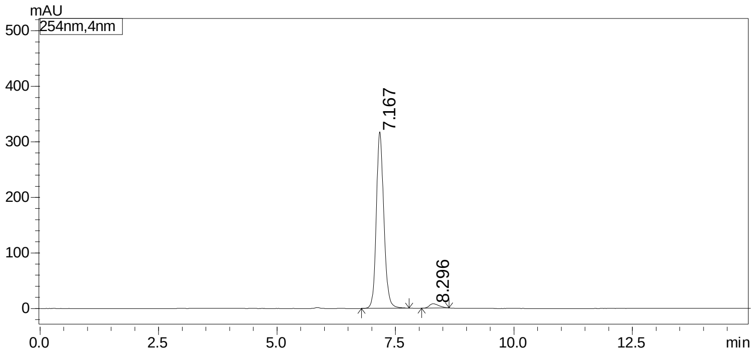

Peak	Ret. time	Area	Height	Area%	Height%
1	7.167	3515420	318134	96.954	97.611
2	8.296	110438	7787	3.046	2.389
Total		3625858	325921	100.000	100.000

Supplementary Figure 150. HPLC spectrum of **4e**

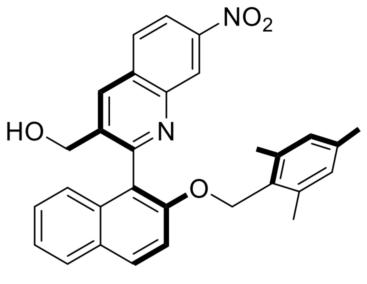

4f

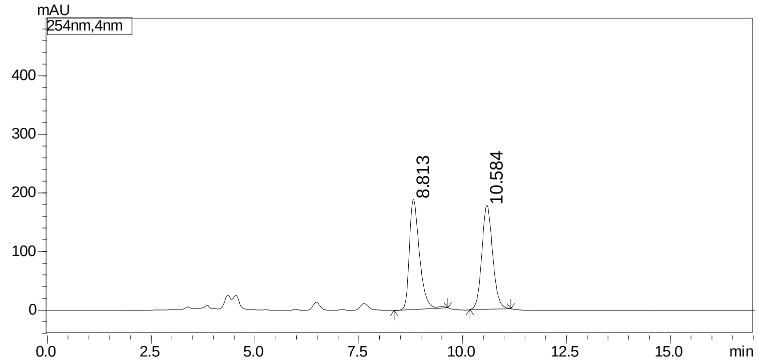

Peak	Ret. time	Area	Height	Area%	Height%
1	8.813	3175824	187942	49.673	51.437
2	10.584	3217668	177439	50.327	48.563
Total		6393492	365380	100.000	100.000

Supplementary Figure 151. HPLC spectrum of *racemic* **4f**

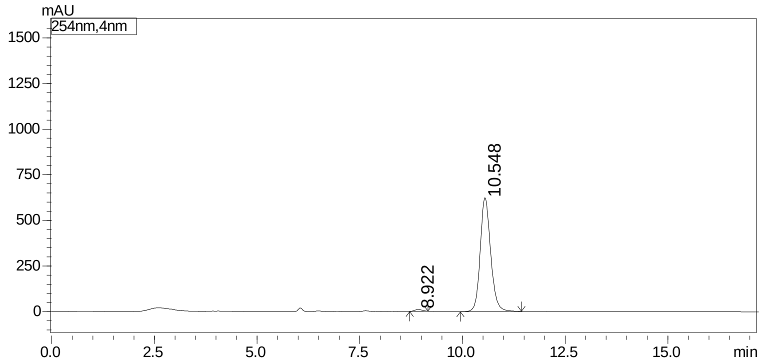

Peak	Ret. time	Area	Height	Area%	Height%
1	8.922	137911	10090	1.230	1.591
2	10.548	11074884	624298	98.770	98.409
Total		11212795	634389	100.000	100.000

Supplementary Figure 152. HPLC spectrum of **4f**

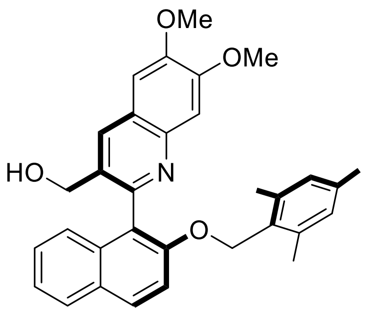

4g

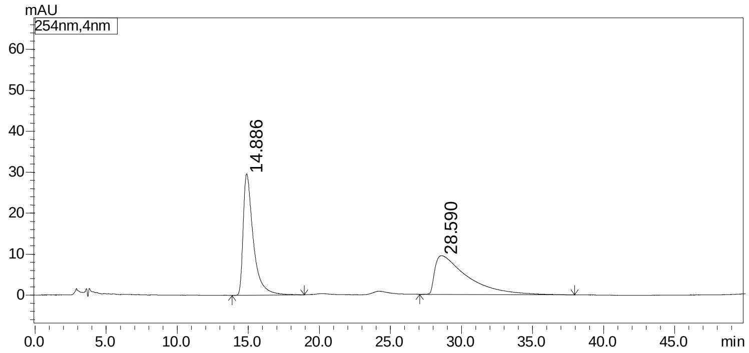

Peak	Ret. time	Area	Height	Area%	Height%
1	14.886	1463326	29618	49.394	75.808
2	28.590	1499239	9452	50.606	24.192
Total		2962564	39069	100.000	100.000

Supplementary Figure 153. HPLC spectrum of *racemic* **4g**

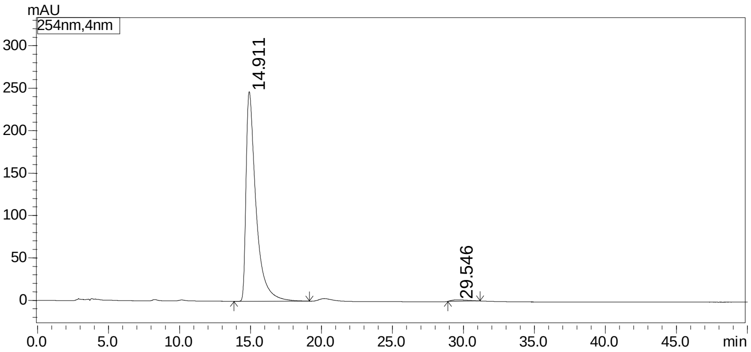

Peak	Ret. time	Area	Height	Area%	Height%
1	14.911	12047443	247001	98.961	99.346
2	29.546	126444	1626	1.039	0.654
Total		12173887	248626	100.000	100.000

Supplementary Figure 154. HPLC spectrum of **4g**

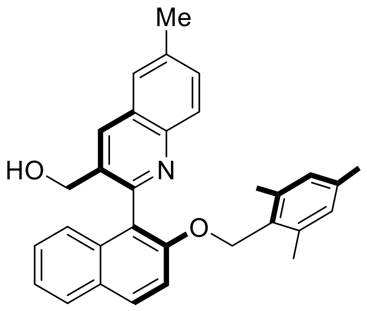

4h

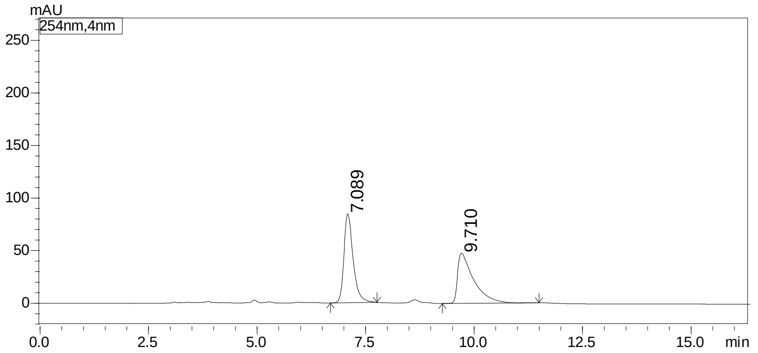

Peak	Ret. time	Area	Height	Area%	Height%
1	7.089	1246544	84428	50.528	63.929
2	9.710	1220490	47637	49.472	36.071
Total		2467035	132065	100.000	100.000

Supplementary Figure 155. HPLC spectrum of *racemic* **4h**

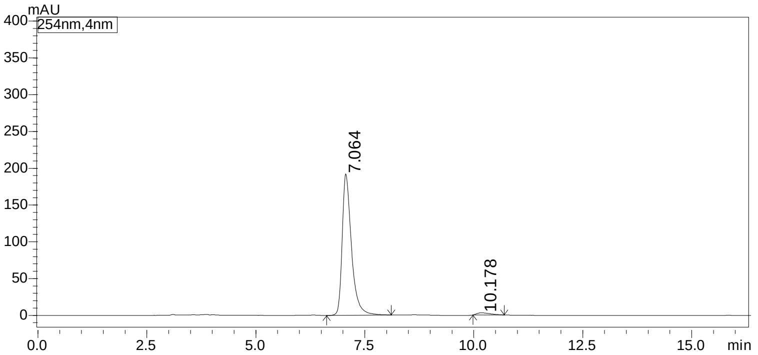

Peak	Ret. time	Area	Height	Area%	Height%
1	7.064	2845593	192076	97.931	98.534
2	10.178	60125	2857	2.069	1.466
Total		2905719	194933	100.000	100.000

Supplementary Figure 156. HPLC spectrum of **4h**

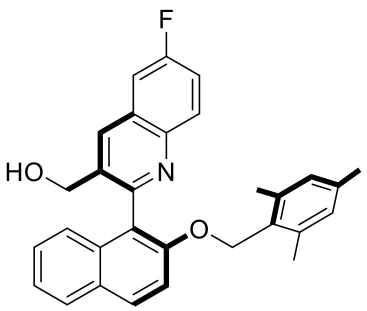

4i

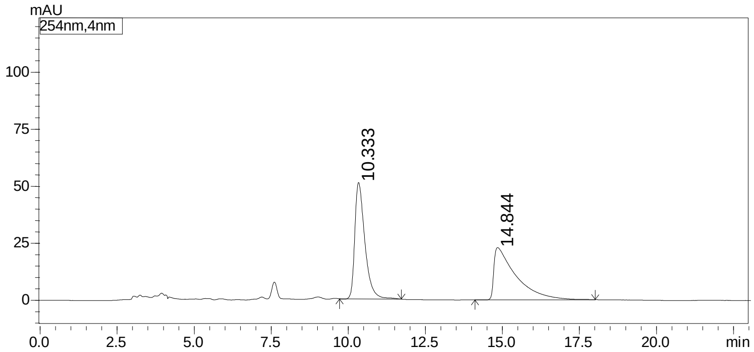

Peak	Ret. time	Area	Height	Area%	Height%
1	10.333	1164331	51079	50.626	69.080
2	14.844	1135549	22863	49.374	30.920
Total		2299881	73943	100.000	100.000

Supplementary Figure 157. HPLC spectrum of *racemic* **4i**

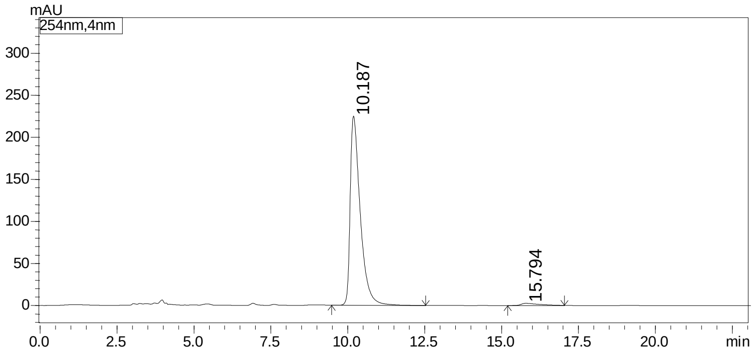

Peak	Ret. time	Area	Height	Area%	Height%
1	10.187	5096868	224996	97.894	98.837
2	15.794	109675	2647	2.106	1.163
Total		5206543	227644	100.000	100.000

Supplementary Figure 158. HPLC spectrum of **4i**

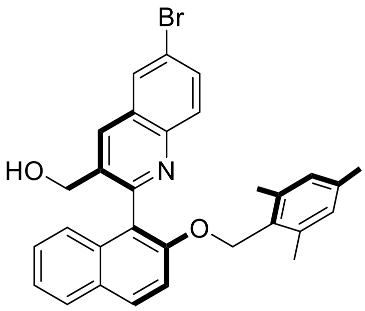

4j

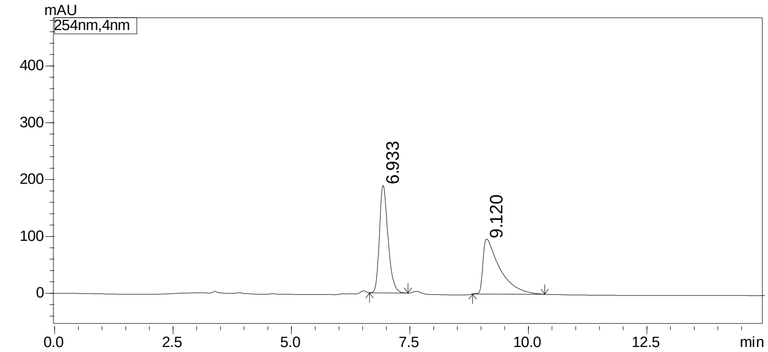

Peak	Ret. time	Area	Height	Area%	Height%
1	6.933	2389386	189245	50.116	66.201
2	9.120	2378336	96620	49.884	33.799
Total		4767722	285865	100.000	100.000

Supplementary Figure 159. HPLC spectrum of *racemic* **4j**

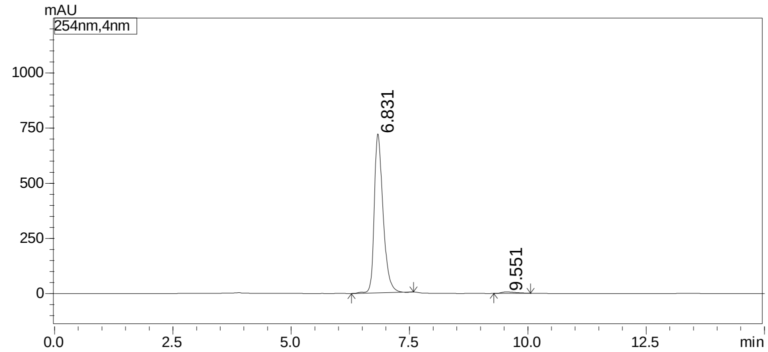

Peak	Ret. time	Area	Height	Area%	Height%
1	6.831	9330149	720962	98.527	99.082
2	9.551	139507	6676	1.473	0.918
Total		9469656	727638	100.000	100.000

Supplementary Figure 160. HPLC spectrum of **4j**

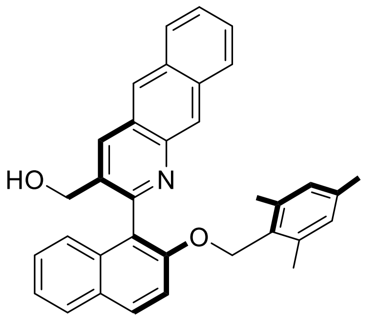

4k

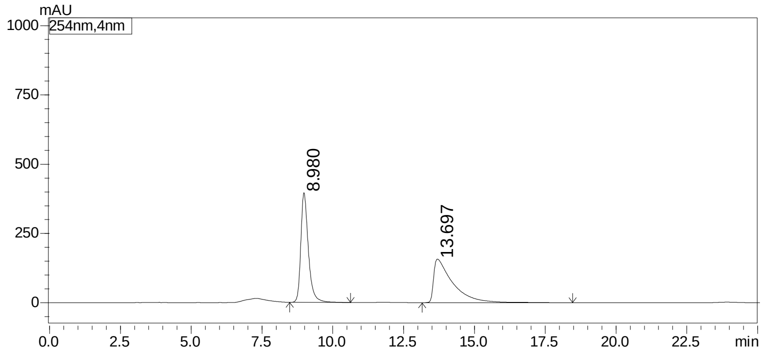

Peak	Ret. time	Area	Height	Area%	Height%
1	8.980	7352181	396204	49.669	71.562
2	13.697	7450191	157450	50.331	28.438
Total		14802371	553655	100.000	100.000

Supplementary Figure 161. HPLC spectrum of *racemic* **4k**

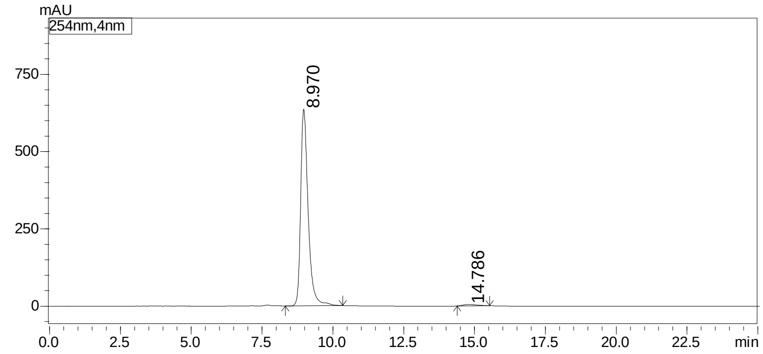

Peak	Ret. time	Area	Height	Area%	Height%
1	8.970	11665992	636348	98.688	99.335
2	14.786	155089	4259	1.312	0.665
Total		11821081	640607	100.000	100.000

Supplementary Figure 162. HPLC spectrum of **4k**

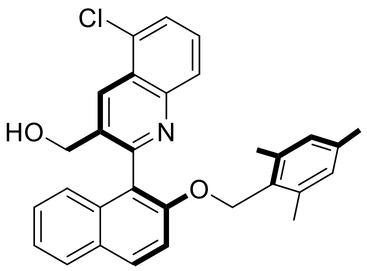

4l

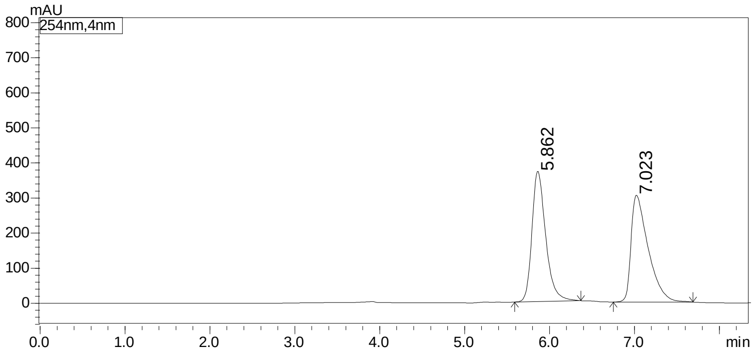

Peak	Ret. time	Area	Height	Area%	Height%
1	5.862	4195625	371029	49.544	54.924
2	7.023	4272931	304505	50.456	45.076
Total		8468557	675534	100.000	100.000

Supplementary Figure 163. HPLC spectrum of *racemic* **4l**

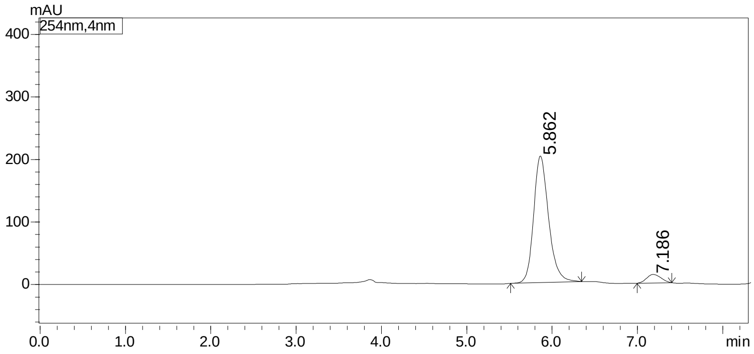

Peak	Ret. time	Area	Height	Area%	Height%
1	5.862	2472982	202539	93.971	93.702
2	7.186	158675	13612	6.029	6.298
Total		2631658	216151	100.000	100.000

Supplementary Figure 164. HPLC spectrum of **4l**

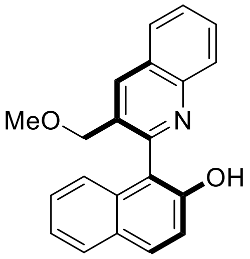

5

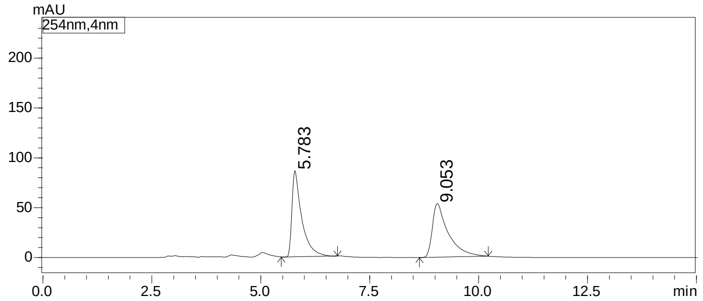

Peak	Ret. time	Area	Height	Area%	Height%
1	5.783	1340206	86352	49.751	61.667
2	9.053	1353607	53678	50.249	38.333
Total		2693813	140031	100.000	100.000

Supplementary Figure 165. HPLC spectrum of *racemic* **5**

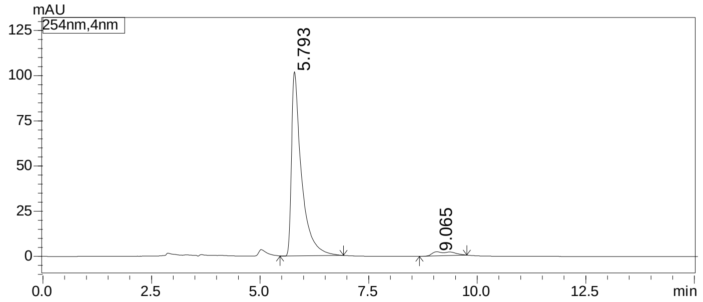

Peak	Ret. time	Area	Height	Area%	Height%
1	5.793	1630649	100965	95.737	97.784
2	9.065	72612	2310	4.263	2.216
Total		1703261	104276	100.000	100.000

Supplementary Figure 166. HPLC spectrum of **5**

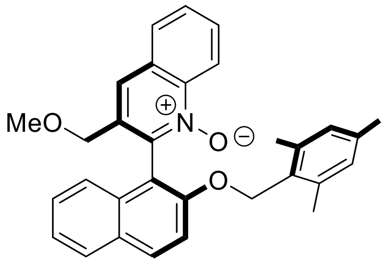

6

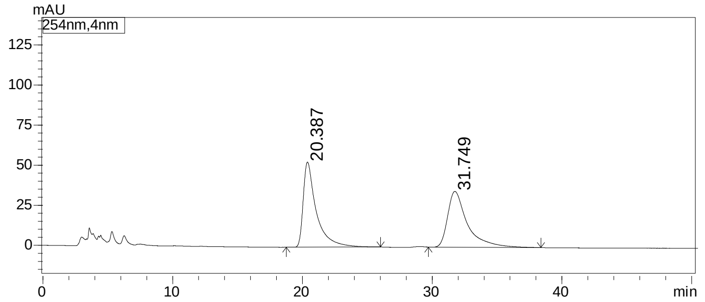

Peak	Ret. time	Area	Height	Area%	Height%
1	20.387	3706846	53092	50.137	60.374
2	31.749	3686571	34847	49.863	39.626
Total		7393417	87938	100.000	100.000

Supplementary Figure 167. HPLC spectrum of *racemic* **6**

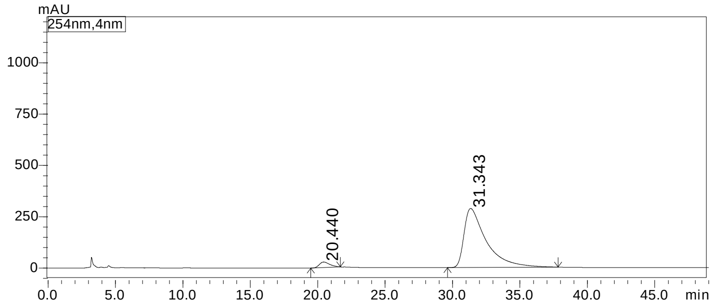

Peak	Ret. time	Area	Height	Area%	Height%
1	20.440	1441009	27031	4.178	8.585
2	31.343	33053137	287816	95.822	91.415
Total		34494156	314847	100.000	100.000

Supplementary Figure 168. HPLC spectrum of **6**

General information

Chemicals were purchased from commercial suppliers and used as received. Solvents were dried on alumina columns using a solvent dispensing system. Thin-layer chromatography (TLC) was conducted on plates (GF254) supplied by Yantai Chemicals (China) and visualized using a combination of UV, anisaldehyde, iodine, and potassium permanganate staining. ^{1}H NMR, ^{13}C NMR, ^{19}F NMR, spectra were recorded on a Bruker ACF400 (400 MHz) spectrometer. Chemical shifts were reported in parts per million (ppm), and the residual solvent peak was used as an internal reference: proton (chloroform δ 7.26, menthol δ 3.31), carbon (chloroform δ 77.16) or tetramethylsilane (TMS δ 0.00) was used as a reference. Multiplicity was indicated as follows: s (singlet), d (doublet), t (triplet), q (quartet), m (multiplet), dd (doublet of doublet), bs (broad singlet). Coupling constants were reported in Hertz (Hz). All high resolution mass spectra were obtained from the Tsinghua University Mass Spectrometry Facility. Flash chromatography separations were performed on Silica gel (300-400 mesh) supplied by Tsingdao Haiyang Chemicals (China). Infrared spectra were recorded on a Perkin Elmer Spectrum two equipped with MIRacle™ single reflection with ZnSe ATR unit. The enantiomeric excesses of products were determined on a Shimadzu LC-20AT Chiral HPLC. Optical rotations were recorded on Optical rotations were recorded on SGW-1 autopolarimeter.

General procedure for the synthesis of 1[1-3]

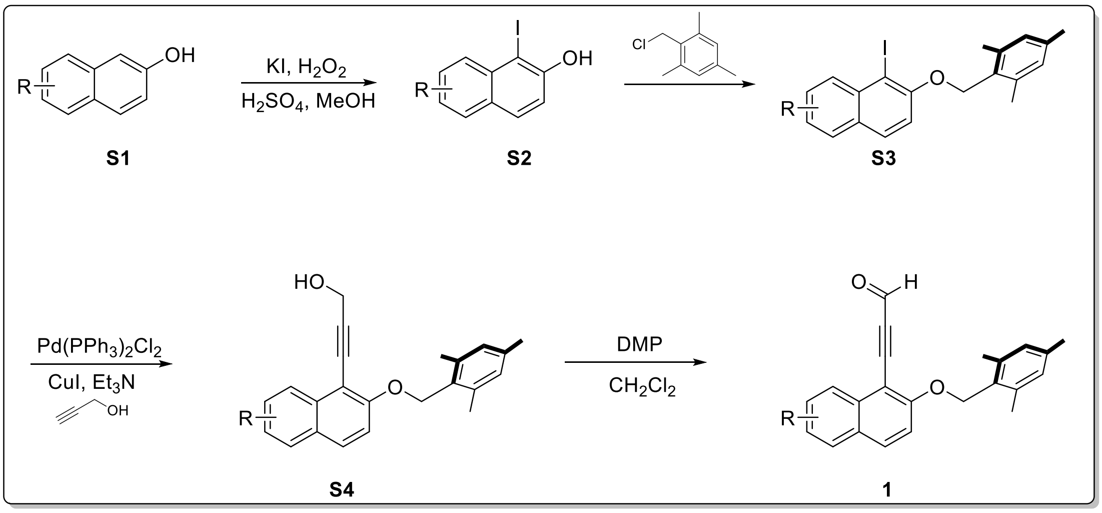

To MeOH (50 mL) being stirred at 0 °C in a round-bottom flask was added sulfuric acid (95.8%, 30.0 mmol, 2.0 equiv), **S1** (10.0 mmol), KI (10.0 mmol, 1.0 equiv), and H_2O_2 (31.6%, 42.0 mmol, 22.0 equiv). The dark mixture was kept stirring at 0 °C for 9 h at when the substrate appeared consumed (TLC). The mixture was quenched by adding excess saturated aqueous Na_2SO_3. The MeOH was evaporated, and the residue was extracted with EtOAc (3 x 30 mL) and washed with brine, dried over sodium sulfate. The solvent was evaporated to yield the product **S2** which was used directly for the next step.

To a solution of **S2** (10.0 mmol) in MeCN (30 mL) was added K_2CO_3 (20.0 mmol, 2.0 equiv), 2-(chloromethyl)-1,3,5-trimethylbenzene (15.0 mmol, 1.5 equiv). The solution was warmed to 70 °C for 4 h at which time TLC indicated complete consumption of the naphthol. The reaction mixture was cooled to RT. Et_3N (10.0 mL) was added, and the reaction was stirred for 1 h. The reaction mixture was filtered through a pad of Celite, the solid was washed with DCM and the solvent was evaporated to yield a yellow solid. The solid was washed with PE for three times to give the product **S3** which was used directly for the next step.

To a solution of **S3** (10.0 mmol) and propargyl alcohol (30.0 mmol, 3.0 equiv) in triethylamine (30 mL) was added $Pd(PPh_3)_2Cl_2$ (0.4 mmol, 0.04 equiv) and CuI (0.8 mmol, 0.08 equiv). The flask was flushed with nitrogen gas. The resulting mixture was stirred at room temperature for 40 h. The reaction mixture was filtered and washed with EtOAc. The combined filtrate was concentrated and the residue was purified by a silica gel column chromatography to give **S4**. To a solution of **S4** (7.0 mmol) in DCM (50 mL) was added $NaHCO_3$ (14.0 mmol, 2.0 equiv), and Dess-Martin oxidant (10.5 mmol, 1.5 equiv) at 0 °C. The reaction was stirred at room temperature for 2 h. at which time TLC indicated complete conumption of the **S4**. The mixture was quenched by adding excess saturated aqueous Na_2SO_3. The mixture was vigorously stirred at room temperature for 1 h. The mixture was extracted with EtOAc (3 x 30 mL) and washed with brine, dried over sodium sulfate. The solvent was concentrated and the residue was

purified by a silica gel column chromatography to give **1**.

Characterization data of 1.

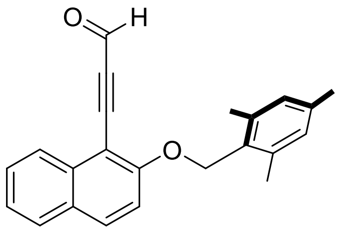

1c

1c: Yellow solid; **^{1}H NMR** (400 MHz, Chloroform-*d*) δ 9.49 (s, 1H), 8.26 (d, *J* = 8.4 Hz, 1H), 8.01 (d, *J* = 9.1 Hz, 1H), 7.85 (d, *J* = 8.1 Hz, 1H), 7.63 (t, *J* = 7.7 Hz, 1H), 7.52 – 7.37 (m, 2H), 6.95 (s, 2H), 5.32 (s, 2H), 2.47 (s, 4H), 2.33 (s, 3H). **^{13}C NMR** (100 MHz, Chloroform-*d*) δ 176.79, 161.90, 138.57, 138.16, 134.93, 133.89, 129.24, 129.15, 128.55, 128.50, 128.40, 125.01, 124.93, 114.68, 103.64, 98.11, 91.38, 67.09, 21.11, 19.76; **HRMS** (ESI): *m/z*: calculated for $C_{23}H_{21}O_2$: $[M + H]^+$ 329.1536, found: 329.1540.

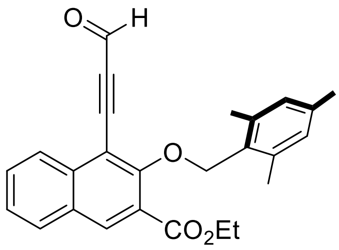

1d

1d: White solid; **^{1}H NMR** (400 MHz, Chloroform-*d*) δ 9.33 (s, 1H), 8.45 (s, 1H), 8.22 (d, *J* = 8.5 Hz, 1H), 7.92 (d, *J* = 8.1 Hz, 1H), 7.70 (t, *J* = 7.7 Hz, 1H), 7.56 (t, *J* = 7.5 Hz, 1H), 6.84 (s, 2H), 5.43 (s, 2H), 4.35 (q, *J* = 7.1 Hz, 2H), 2.28 (s, 6H), 2.27 (s, 3H), 1.36 (t, *J* = 7.1 Hz, 3H). **^{13}C NMR** (100 MHz, Chloroform-*d*) δ 176.23, 165.46, 160.85, 138.50, 138.46, 136.10, 135.83, 130.07, 129.99, 129.36, 129.02, 128.83, 126.60, 125.58, 125.30, 110.72, 97.08, 90.02, 72.86, 61.70, 21.05, 19.75, 14.14; **HRMS** (ESI): *m/z*: calculated for $C_{26}H_{25}O_4$: $[M + H]^+$ 401.1747, found: 401.1749.

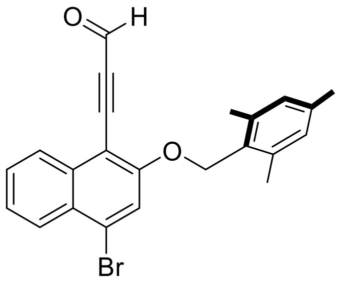

1e

1e: Yellow solid; **^{1}H NMR** (400 MHz, Chloroform-*d*) δ 9.47 (s, 1H), 8.27 (d, *J* = 8.3 Hz, 1H), 8.22 (d, *J* = 8.2 Hz, 1H), 7.79 (s, 1H), 7.66 (t, *J* = 7.4 Hz, 1H), 7.56 (t, *J* = 7.3 Hz, 1H), 6.96 (s, 2H), 5.31 (s, 2H), 2.46 (s, 6H), 2.33 (s, 3H). **^{13}C NMR** (100 MHz, Chloroform-*d*) δ 176.58, 160.81, 138.77, 138.17, 135.24, 129.28, 129.21, 128.97, 128.68, 127.76, 127.52, 126.27, 125.41, 119.10, 103.70, 98.50, 90.26, 67.35, 21.09, 19.72; **HRMS** (ESI): *m/z*: calculated for $C_{23}H_{20}BrO_2$: $[M + H]^+$ 407.0641, found: 407.0638.

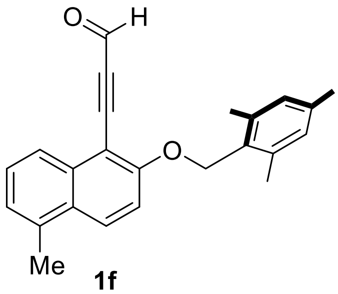

1f

1f: Light yellow solid; **^{1}H NMR** (400 MHz, Chloroform-*d*) δ 9.49 (s, 1H), 8.16 (dd, *J* = 17.0, 8.9 Hz, 2H), 7.51 (t, *J* = 7.7 Hz, 1H), 7.45 (d, *J* = 9.3 Hz, 1H), 7.29 (d, *J* = 7.5 Hz, 1H), 6.95 (s, 2H), 5.32 (s, 2H), 2.71 (s, 3H), 2.47 (s, 6H), 2.33 (s, 3H). **^{13}C NMR** (100 MHz, Chloroform-*d*) δ 176.75, 161.55, 138.51, 138.12, 135.33, 135.03, 130.10, 129.22, 128.37, 127.78, 125.90, 123.38, 114.19, 104.12, 98.09, 91.73, 67.05, 21.08, 19.74, 19.42; **HRMS** (ESI): *m/z*: calculated for $C_{24}H_{23}O_2$: $[M + H]^+$ 343.1693, found: 343.1691.

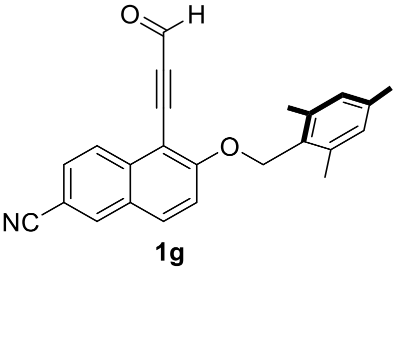

1g

1g: Brown solid; **^{1}H NMR** (400 MHz, Chloroform-*d*) δ 9.47 (s, 1H), 8.31 (d, *J* = 8.7 Hz, 1H), 8.22 (s, 1H), 8.06 (d, *J* = 9.0 Hz, 1H), 7.73 (d, *J* = 8.7 Hz, 1H), 7.57 (d, *J* = 9.1 Hz, 1H), 6.96 (s, 2H), 5.36 (s, 2H), 2.45 (s, 6H), 2.33 (s, 3H). **^{13}C NMR** (100 MHz, Chloroform-*d*) δ 176.47, 163.57, 138.87, 138.09, 136.67, 134.32, 133.97, 133.42, 129.33, 128.90, 128.49, 127.27, 126.24, 118.87, 115.82, 108.40, 103.87, 98.22, 89.07, 67.05, 21.09, 19.73; **HRMS** (ESI): *m/z*: calculated for $C_{24}H_{20}NO_2$: $[M + H]^+$ 354.1489, found: 354.1495.

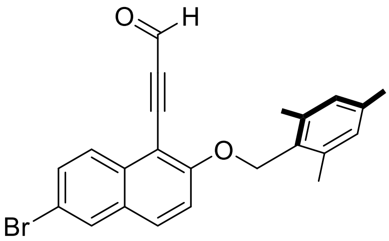

1h

1h: White solid; **^{1}H NMR** (400 MHz, Chloroform-*d*) δ 9.47 (s, 1H), 8.11 (d, *J* = 8.9 Hz, 1H), 7.99 (s, 1H), 7.90 (d, *J* = 9.1 Hz, 1H), 7.67 (d, *J* = 9.0 Hz, 1H), 7.46 (d, *J* = 9.1 Hz, 1H), 6.95 (s, 2H), 5.31 (s, 2H), 2.45 (s, 6H), 2.33 (s, 3H). **^{13}C NMR** (100 MHz, Chloroform-*d*) δ 176.58, 161.88, 138.66, 138.11, 133.46, 132.65, 131.66, 130.28, 129.56, 129.26, 128.93, 126.74, 118.76, 115.66, 103.88, 98.06, 90.26, 67.12, 21.08, 19.71; **HRMS** (ESI): *m/z*: calculated for $C_{23}H_{20}BrO_2$: $[M + H]^+$ 407.0641, found: 407.0643.

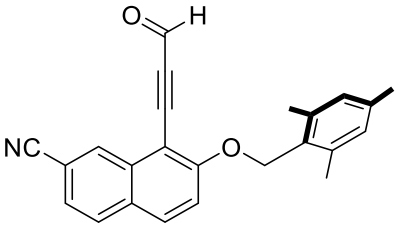

1i

1i: Brown solid; **^{1}H NMR** (400 MHz, Chloroform-*d*) δ 9.51 (s, 1H), 8.59 (s, 1H), 8.05 (d, *J* = 9.2 Hz, 1H), 7.93 (d, *J* = 8.4 Hz, 1H), 7.60 (d, *J* = 8.9 Hz, 2H), 6.95 (s, 2H), 5.35 (s, 2H), 2.45 (s, 6H), 2.33 (s, 3H). **^{13}C NMR** (100 MHz, Chloroform-*d*) δ 176.28, 162.62, 138.84, 138.08, 134.01, 133.40, 130.75, 129.65, 129.60, 129.32, 128.59, 125.65, 118.86, 117.47, 111.93, 98.34, 88.62, 67.23, 21.09, 19.72; **HRMS** (ESI): *m/z*: calculated for $C_{24}H_{20}NO_2$: $[M + H]^+$ 354.1489, found: 354.1485.

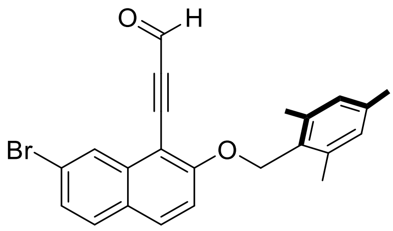

1j

1j: White solid; **^{1}H NMR** (400 MHz, Chloroform-*d*) δ 9.50 (s, 1H), 8.37 (d, *J* = 2.0 Hz, 1H), 7.96 (d, *J* = 9.1 Hz, 1H), 7.70 (d, *J* = 8.7 Hz, 1H), 7.53 (dd, *J* = 8.7, 1.9 Hz, 1H), 7.44 (d, *J* = 9.1 Hz, 1H), 6.94 (s, 2H), 5.32 (s, 2H), 2.45 (s, 6H), 2.33 (s, 3H). **^{13}C NMR** (100 MHz, Chloroform-*d*) δ 176.60, 162.40, 138.67, 138.11, 136.05, 133.62, 129.93, 129.27, 128.89, 128.52, 127.09, 126.91, 123.42, 114.82, 102.86, 98.05, 90.30, 67.07, 21.10, 19.75; **HRMS** (ESI): *m/z*: calculated for $C_{23}H_{20}BrO_2$: $[M + H]^+$ 407.0641, found: 407.0644.

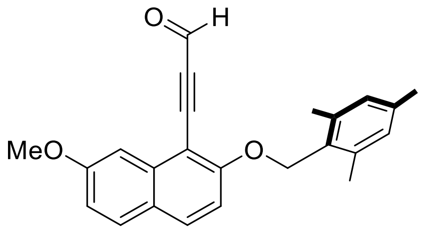

1k

1k: White solid; **^{1}H NMR** (400 MHz, Chloroform-*d*) δ 9.48 (s, 1H), 7.92 (d, *J* = 9.0 Hz, 1H), 7.72 (d, *J* = 8.9 Hz, 1H), 7.51 (d, *J* = 2.4 Hz, 1H), 7.27 (d, *J* = 8.5 Hz, 1H), 7.10 (dd, *J* = 8.9, 2.5 Hz, 1H), 6.95 (s, 2H), 5.30 (s, 2H), 3.99 (s, 3H), 2.46 (s, 6H), 2.33 (s, 3H). **^{13}C NMR** (100 MHz, Chloroform-*d*) δ 176.71, 162.52, 160.06, 138.51, 138.15, 136.98, 133.63, 130.06, 129.22, 123.91, 117.69, 111.61, 103.30, 102.47, 98.58, 91.96, 66.84, 55.49, 21.10, 19.75; **HRMS** (ESI): *m/z*: calculated for $C_{24}H_{23}O_3$: $[M + H]^+$ 359.1642, found: 359.1638.

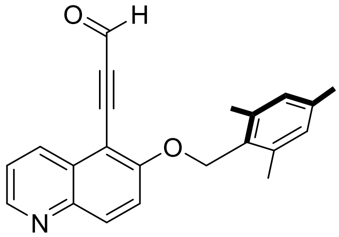

1l

1l: White solid; **^{1}H NMR** (400 MHz, Chloroform-*d*) δ 9.47 (s, 1H), 8.88 (d, *J* = 4.0 Hz, 1H), 8.56 (d, *J* = 8.5 Hz, 1H), 8.30 (d, *J* = 9.4 Hz, 1H), 7.68 (d, *J* = 9.4 Hz, 1H), 7.53

(dd, J = 8.5, 4.2 Hz, 1H), 6.95 (s, 2H), 5.35 (s, 2H), 2.46 (s, 6H), 2.33 (s, 3H). **^{13}C NMR** (100 MHz, Chloroform-*d*) δ 176.57, 161.76, 149.06, 143.20, 138.78, 138.15, 135.04, 133.47, 130.41, 129.30, 128.76, 122.94, 117.97, 103.31, 97.86, 89.32, 67.18, 21.11, 19.74; **HRMS** (ESI): *m/z*: calculated for $C_{22}H_{20}NO_2$: $[M + H]^+$ 330.1489, found: 330.1495.

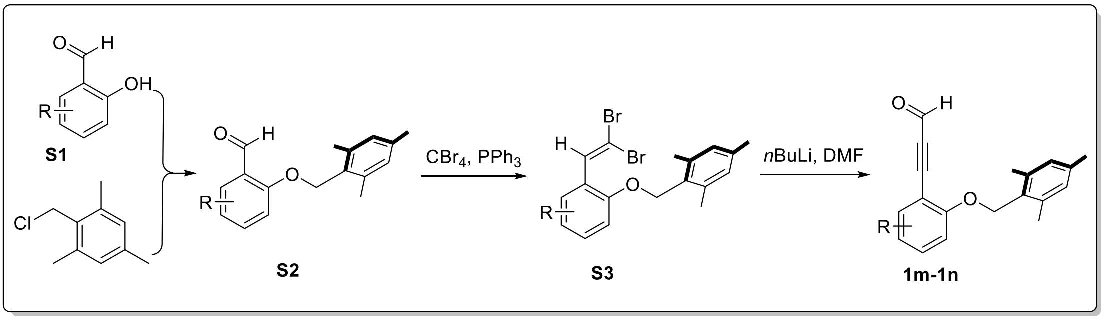

To a solution of **S1** (10.0 mmol) in MeCN (30 mL) was added K_2CO_3 (20.0 mmol, 2.0 equiv), 2-(chloromethyl)-1,3,5-trimethylbenzene (15.0 mmol, 1.5 equiv). The solution was warmed to 70 °C for 4 h at which time TLC indicated complete consumption of the naphthol. The reaction mixture was cooled to RT. Et_3N (10.0 mL) was added, and the reaction was stirred for 1 h. The reaction mixture was filtered through a pad of Celite, the solid was washed with DCM and the solvent was evaporated to yield a yellow solid. The solid was washed with PE for three times to give the product **S2** which was used directly for the next step.

To a solution of **S2** (10.0 mmol) in DCM (50 mL) was added PPh_3 (50.0 mmol, 5.0 equiv), CBr_4 (25.0 mmol, 2.5 equiv) was added slowly at 0 °C. The solution was stirred for 30 mins. Half of the solvent was evaporated, PE: EtOAc (50 mL, 1:1) was added to the mixture. The mixture was filtered through a pad of silica gel, the solid was washed with PE: EtOAc (50 mL, 1:1) for three times and the solvent was evaporated and the residue was purified by a silica gel column chromatography to give **S3**.

To the above product **S3** (8.0 mmol), in THF (30 mL) was added *n*BuLi (17.6 mmol, 2.2 equiv) at -78 °C under N_2, the mixture was warmed to -30 °C and stirred for 1 h. Then DMF (16.0 mmol, 2.0 equiv) was added to the reaction mixture. The reaction was

warmed to room temperature and stirred for another 2 h and then quenched by addition of NH_4Cl. The reaction mixture was extracted with EtOAc (3 x 30 mL) and washed with brine, dried over sodium sulfate. The solvent was concentrated and the residue was purified by a silica gel column chromatography to give **1m-1n**.

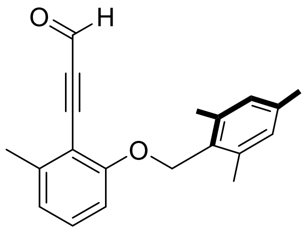

1m

1m: White solid; **^{1}H NMR** (400 MHz, Chloroform-*d*) δ 9.39 (s, 1H), 7.37 (t, *J* = 8.0 Hz, 1H), 6.98 - 6.90 (m, 4H), 5.12 (s, 2H), 2.49 (s, 3H), 2.42 (s, 6H), 2.33 (s, 3H). **^{13}C NMR** (100 MHz, Chloroform-*d*) δ 176.92, 161.82, 144.85, 138.33, 138.09, 132.15, 129.27, 129.14, 122.38, 110.00, 109.64, 96.74, 91.65, 65.99, 21.06, 20.78, 19.67; **HRMS** (ESI): *m/z*: calculated for $C_{20}H_{21}O_2$: $[M + H]^+$ 293.1536, found: 293.1540.

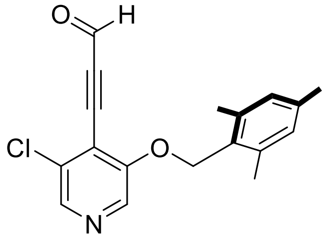

1n

1n: White solid; **^{1}H NMR** (400 MHz, Chloroform-*d*) δ 9.42 (s, 1H), 8.46 (s, 1H), 8.39 (s, 1H), 6.94 (s, 2H), 5.27 (s, 2H), 2.40 (s, 6H), 2.32 (s, 3H). **^{13}C NMR** (100 MHz, Chloroform-*d*) δ 175.87, 156.26, 142.02, 138.98, 138.09, 134.10, 133.63, 129.32, 128.17, 117.48, 97.93, 84.11, 67.35, 21.05, 19.59; **HRMS** (ESI): *m/z*: calculated for $C_{18}H_{17}ClNO_2$: $[M + H]^+$ 314.0942, found: 314.0937.

General procedure for the synthesis of 2[4]

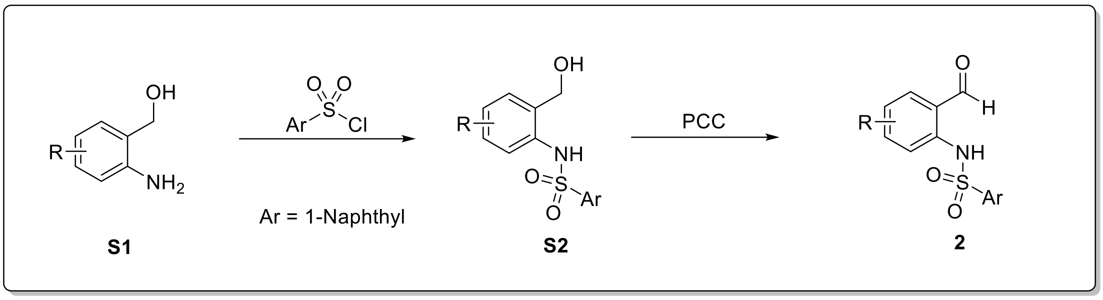

To a solution of S1 (5.0 mmol, 1.0 equiv) in DCM (10 mL) was added pyridine (6.0 mmol, 1.2 equiv) followed by naphthalene-1-sulfonyl chloride (5.0 mmol, 1.0 equiv) at room temperature. The resulting mixture was continued to stir at room temperature for 5 h. After completion of the reaction, as monitored by TLC, the reaction mixture was poured into water and the aqueous suspension was extracted with DCM (2 x 40 mL), washed with water and brine. The organic layer was dried over anhydrous Na_2SO_4 and concentrated under reduced pressure. The crude product was purified by flash column chromatography using petroleum ether-ethyl acetate mixture as eluent (80:20, v/v) to obtain compounds **S2**.

Compound **S2** (5.0 mmol, 1.0 equiv) was added to a stirred solution of PCC (7.5 mmol, 1.5 equiv) in DCM (10 mL) at room temperature and stirring was continued for 1.5 h. After completion of the reaction, as monitored by TLC, the reaction mixture was passed through silica gel (60-120 mesh) and the collected fraction was concentrated under reduced pressure to obtain compound **2.**

Characterization data of 2.

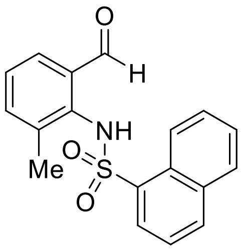

2c

2c: Light yellow solid; **^{1}H NMR** (400 MHz, Chloroform-*d*) δ 9.40 (s, 1H), 8.52 (s, 1H), 8.37 (d, *J* = 8.6 Hz, 1H), 8.13 (d, *J* = 7.4 Hz, 1H), 8.06 (d, *J* = 8.2 Hz, 1H), 7.90 (d, *J* =

8.1 Hz, 1H), 7.54 (t, J = 7.5 Hz, 1H), 7.51 – 7.43 (m, 3H), 7.35 (d, J = 7.4 Hz, 1H), 7.30 (d, J = 7.8 Hz, 1H), 2.31 (s, 3H). **^{13}C NMR** (100 MHz, Chloroform-*d*) δ 192.36, 137.95, 137.28, 136.00, 134.96, 134.69, 133.85, 131.09, 131.06, 129.66, 128.76, 128.68, 127.99, 126.97, 126.79, 124.58, 124.24, 18.77; **HRMS** (ESI): *m/z*: calculated for $C_{18}H_{16}NO_3S$: $[M + H]^+$ 326.0845, found: 326.0844.

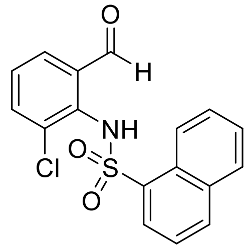

2d

2d: Yellow solid; **^{1}H NMR** (400 MHz, Chloroform-*d*) δ 10.30 (s, 1H), 8.43 (d, J = 8.6 Hz, 1H), 8.10 (d, J = 8.3 Hz, 1H), 8.05 (d, J = 7.4 Hz, 1H), 7.93 (d, J = 8.3 Hz, 1H), 7.90 (t, J = 4.6 Hz, 1H), 7.58 (t, J = 7.5 Hz, 1H), 7.51 (t, J = 7.8 Hz, 1H), 7.46 (t, J = 7.8 Hz, 1H), 7.30 (d, J = 4.4 Hz, 3H). **^{13}C NMR** (100 MHz, Chloroform-*d*) δ 189.26, 135.30, 134.92, 134.59, 134.24, 134.14, 134.11, 131.63, 130.02, 129.03, 128.73, 128.33, 128.12, 127.97, 126.94, 124.16, 123.98; **HRMS** (ESI): *m/z*: calculated for $C_{17}H_{13}ClNO_3S$: $[M + H]^+$ 346.0299, found: 346.0297.

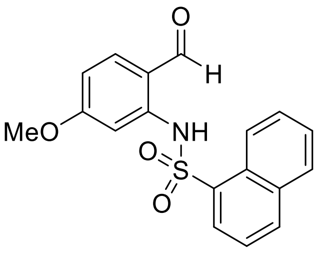

2e

2e: Light yellow solid; **^{1}H NMR** (400 MHz, Chloroform-*d*) δ 11.59 (s, 1H), 9.60 (s, 1H), 8.71 (d, J = 8.7 Hz, 1H), 8.41 (d, J = 7.3 Hz, 1H), 8.06 (d, J = 8.2 Hz, 1H), 7.90 (d, J = 8.2 Hz, 1H), 7.71 (t, J = 7.8 Hz, 1H), 7.56 (dt, J = 16.0, 7.7 Hz, 2H), 7.37 (d, J = 8.6 Hz, 1H), 7.28 (s, 1H), 6.53 (d, J = 8.6 Hz, 1H), 3.80 (s, 3H). **^{13}C NMR** (100 MHz, Chloroform-*d*) δ 193.02, 165.39, 142.20, 138.06, 135.10, 134.24, 133.87, 130.39, 129.06, 128.75, 127.93, 127.12, 124.19, 123.75, 115.50, 108.98, 100.66, 55.71; **HRMS** (ESI): *m/z*: calculated for $C_{18}H_{16}NO_4S$: $[M + H]^+$ 342.0795, found: 342.0799.

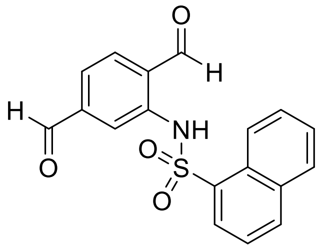

2f

2f: Colorless solid; **^{1}H NMR** (400 MHz, Chloroform-*d*) δ 11.27 (s, 1H), 9.98 (s, 1H), 9.89 (s, 1H), 8.69 (d, *J* = 8.7 Hz, 1H), 8.48 (d, *J* = 7.4 Hz, 1H), 8.11 (s, 1H), 8.07 (d, *J* = 8.4 Hz, 1H), 7.92 (d, *J* = 8.2 Hz, 1H), 7.72 (d, *J* = 8.1 Hz, 2H), 7.58 (t, *J* = 8.2 Hz, 3H). **^{13}C NMR** (100 MHz, Chloroform-*d*) δ 194.72, 190.72, 140.48, 136.58, 135.41, 134.26, 133.45, 130.67, 129.23, 128.91, 127.78, 127.22, 124.22, 124.00, 123.97, 122.27, 118.18; **HRMS** (ESI): *m/z*: calculated for $C_{18}H_{16}NO_4S$: [M + H]$^+$ 340.0638, found: 340.0636.

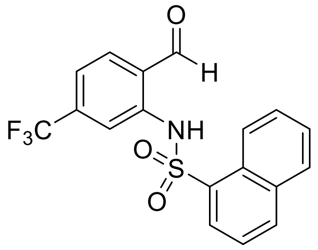

2g

2g: White sold; **^{1}H NMR** (400 MHz, Chloroform-*d*) δ 11.27 (s, 1H), 9.85 (s, 1H), 8.68 (d, *J* = 8.7 Hz, 1H), 8.45 (d, *J* = 7.2 Hz, 1H), 8.08 (d, *J* = 8.2 Hz, 1H), 7.92 (t, *J* = 4.0 Hz, 2H), 7.73 (t, *J* = 7.8 Hz, 1H), 7.66 (d, *J* = 8.0 Hz, 1H), 7.62 – 7.56 (m, 2H), 7.31 (d, *J* = 8.0 Hz, 1H). **^{13}C NMR** (100 MHz, Chloroform-*d*) δ 194.28, 140.17, 136.35, 135.49, 134.26, 133.16, 130.91, 129.22, 128.92, 127.77, 127.21, 123.91, 123.88, 122.98, 118.96 (q, *J* = 3.6 Hz), 114.01 (q, *J* = 4.0 Hz); **HRMS** (ESI): *m/z*: calculated for $C_{18}H_{13}F_3NO_3S$: [M + H]$^+$ 380.0563, found: 380.0568.

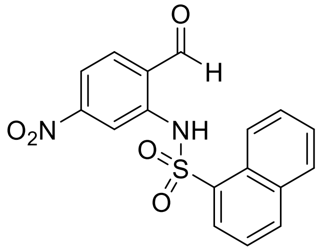

2h

2h: Brown solid; **^{1}H NMR** (400 MHz, Chloroform-*d*) δ 11.30 (s, 1H), 9.92 (s, 1H),

8.65 (d, J = 8.4 Hz, 1H), 8.52 (d, J = 7.0 Hz, 1H), 8.44 (s, 1H), 8.09 (d, J = 7.9 Hz, 1H), 7.92 (d, J = 7.8 Hz, 1H), 7.84 (d, J = 7.9 Hz, 1H), 7.73 (t, J = 8.0 Hz, 2H), 7.60 (t, J = 7.7 Hz, 2H). **^{13}C NMR** (100 MHz, Chloroform-*d*) δ 194.00, 151.36, 140.76, 136.93, 135.73, 134.28, 132.93, 131.10, 129.34, 129.05, 127.69, 127.31, 124.12, 124.06, 123.74, 116.61, 111.88; **HRMS** (ESI): *m/z*: calculated for $C_{17}H_{13}N_2O_5S$: $[M + H]^+$ 357.0540, found: 357.0540.

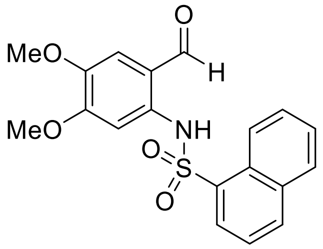

2i

2i: Brown solid; **^{1}H NMR** (400 MHz, Chloroform-*d*) δ 11.40 (s, 1H), 9.57 (s, 1H), 8.70 (d, J = 8.7 Hz, 1H), 8.36 (d, J = 7.2 Hz, 1H), 8.05 (d, J = 8.2 Hz, 1H), 7.90 (d, J = 8.1 Hz, 1H), 7.71 (t, J = 7.6 Hz, 1H), 7.59 (t, J = 7.6 Hz, 1H), 7.51 (t, J = 7.8 Hz, 1H), 7.23 (s, 1H), 6.86 (s, 1H), 3.90 (s, 3H), 3.80 (s, 3H). **^{13}C NMR** (100 MHz, Chloroform-*d*) δ 192.77, 155.19, 144.58, 135.97, 135.13, 134.20, 133.87, 130.27, 129.05, 128.76, 127.89, 127.17, 124.20, 123.66, 116.16, 114.41, 100.68, 56.37, 56.19; **HRMS** (ESI): *m/z*: calculated for $C_{19}H_{18}NO_5S$: $[M + H]^+$ 372.0900, found: 372.0896.

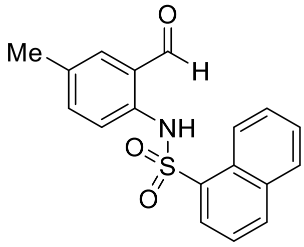

2j

2j: White solid; **^{1}H NMR** (400 MHz, Chloroform-*d*) δ 11.14 (s, 1H), 9.70 (s, 1H), 8.70 (d, J = 8.7 Hz, 1H), 8.38 (dd, J = 7.4, 1.0 Hz, 1H), 8.03 (d, J = 8.2 Hz, 1H), 7.89 (d, J = 8.2 Hz, 1H), 7.70 (t, J = 7.8 Hz, 1H), 7.60 – 7.55 (m, 1H), 7.55 – 7.48 (m, 2H), 7.24 (d, J = 8.5 Hz, 1H), 2.26 (s, 3H). **^{13}C NMR** (100 MHz, Chloroform-*d*) δ 195.06, 137.37, 136.59, 136.21, 134.94, 134.18, 133.87, 132.42, 130.46, 129.05, 128.66, 127.86, 127.06, 124.22, 123.86, 121.40, 116.98, 20.26; **HRMS** (ESI): *m/z*: calculated for $C_{18}H_{16}NO_3S$: $[M + H]^+$ 326.0845, found: 326.0846.

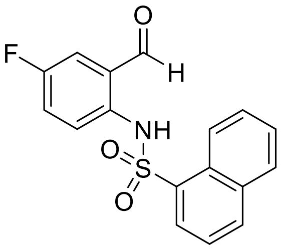

2k

2k: White solid; **^{1}H NMR** (400 MHz, Chloroform-*d*) δ 11.00 (s, 1H), 9.69 (s, 1H), 8.68 (d, *J* = 8.7 Hz, 1H), 8.37 (d, *J* = 7.3 Hz, 1H), 8.06 (d, *J* = 8.2 Hz, 1H), 7.91 (d, *J* = 8.2 Hz, 1H), 7.72 (t, *J* = 7.8 Hz, 1H), 7.67 (dd, *J* = 8.9, 4.2 Hz, 1H), 7.60 (t, *J* = 7.5 Hz, 1H), 7.53 (t, *J* = 7.8 Hz, 1H), 7.21 – 7.16 (m, 2H). **^{13}C NMR** (100 MHz, Chloroform-*d*) δ 193.55 (d, *J* = 2.2 Hz), 157.79 (d, *J* = 245.4 Hz), 136.02 (d, *J* = 2.6 Hz), 135.16, 134.21, 133.63, 130.47, 129.13, 128.77, 127.79, 127.16, 124.10, 123.88, 123.08 (d, *J* = 22.7 Hz), 122.34 (d, *J* = 5.5 Hz), 121.16 (d, *J* = 22.4 Hz), 119.27 (d, *J* = 7.0 Hz); **HRMS** (ESI): *m/z*: calculated for $C_{17}H_{13}FNO_3S$: $[M + H]^+$ 330.0595, found: 330.0600.

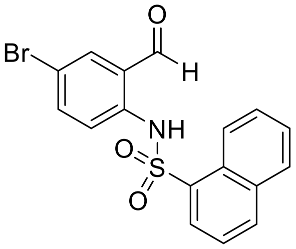

2l

2l: White solid; **^{1}H NMR** (400 MHz, Chloroform-*d*) δ 11.16 (s, 1H), 9.70 (s, 1H), 8.66 (d, *J* = 8.7 Hz, 1H), 8.39 (d, *J* = 7.3 Hz, 1H), 8.07 (d, *J* = 8.1 Hz, 1H), 7.91 (d, *J* = 8.1 Hz, 1H), 7.72 (t, *J* = 7.8 Hz, 1H), 7.61 – 7.51 (m, 5H). **^{13}C NMR** (100 MHz, Chloroform-*d*) δ 193.70, 138.80, 138.47, 138.12, 135.33, 134.23, 133.43, 130.60, 129.21, 128.89, 127.75, 127.22, 123.98, 123.89, 122.63, 118.71, 114.95; **HRMS** (ESI): *m/z*: calculated for $C_{17}H_{13}BrNO_3S$: $[M + H]^+$ 389.9794, found: 389.9797.

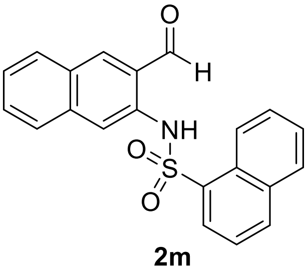

2m

2m: Brown solid; **^{1}H NMR** (400 MHz, Chloroform-*d*) δ 10.97 (s, 1H), 9.88 (s, 1H), 8.76 (d, *J* = 8.7 Hz, 1H), 8.45 (d, *J* = 7.3 Hz, 1H), 8.02 (s, 1H), 7.99 (d, *J* = 8.2 Hz, 1H),

7.95 (s, 1H), 7.86 (d, *J* = 8.1 Hz, 1H), 7.81 – 7.67 (m, 3H), 7.57 (q, *J* = 7.6 Hz, 2H), 7.49 (t, *J* = 7.8 Hz, 1H), 7.41 (t, *J* = 7.5 Hz, 1H). **^{13}C NMR** (100 MHz, Chloroform-*d*) δ 194.85, 140.21, 136.44, 134.90, 134.51, 134.19, 133.69, 130.62, 129.07, 129.04, 128.63, 128.49, 127.91, 127.44, 127.03, 125.91, 124.30, 123.84, 122.37, 114.32; **HRMS** (ESI): *m/z*: calculated for $C_{21}H_{16}NO_3S$: $[M + H]^+$ 362.0845, found: 362.0844.

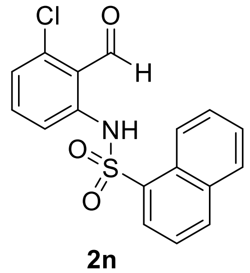

2n

2n: Light yellow solid; **^{1}H NMR** (400 MHz, Chloroform-*d*) δ 11.85 (s, 1H), 10.40 (s, 1H), 8.68 (d, *J* = 8.7 Hz, 1H), 8.40 (d, *J* = 7.4 Hz, 1H), 8.08 (d, *J* = 8.2 Hz, 1H), 7.92 (d, *J* = 8.2 Hz, 1H), 7.73 (t, *J* = 7.8 Hz, 1H), 7.60 (t, *J* = 7.6 Hz, 1H), 7.55 (T, *J* = 8.3 Hz, 2H), 7.32 (t, *J* = 8.3 Hz, 1H), 6.99 (d, *J* = 7.9 Hz, 1H). **^{13}C NMR** (100 MHz, Chloroform-*d*) δ 193.73, 142.15, 140.35, 136.31, 135.27, 134.25, 133.63, 130.55, 129.17, 128.85, 127.82, 127.20, 124.14, 124.06, 123.86, 117.10, 115.72; **HRMS** (ESI): *m/z*: calculated for $C_{17}H_{13}ClNO_3S$: $[M + H]^+$ 346.0299, found: 346.0295.

Supplementary Table 1. Opitimization of the reaction conditions.[a]

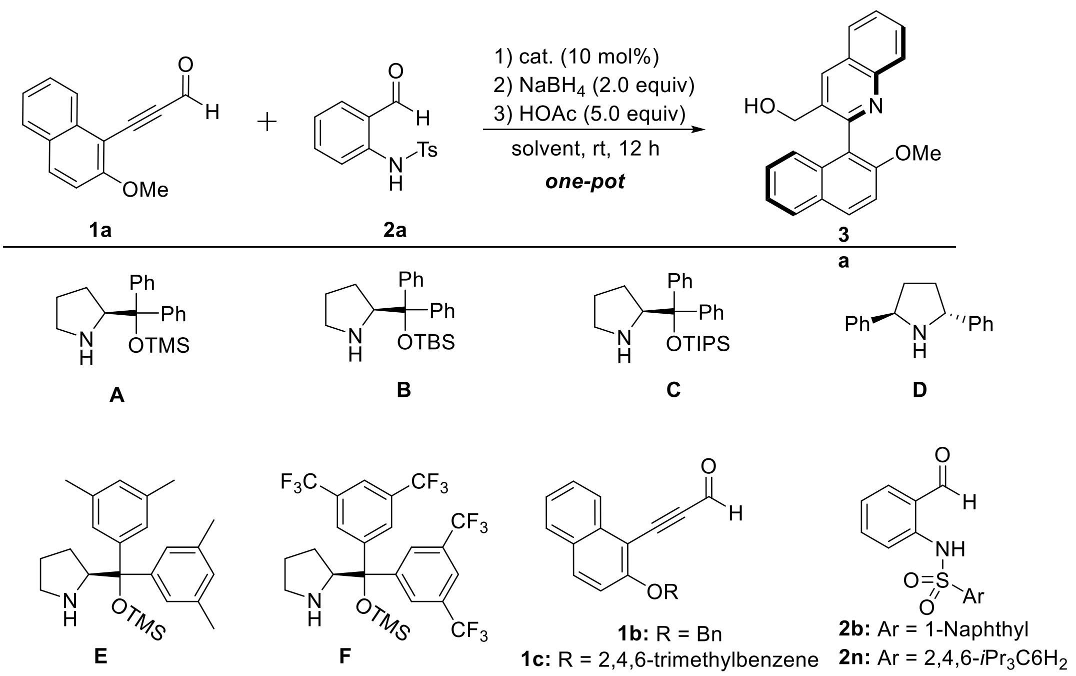

Entry	Cat.	Solvent	**Yield** [%][b]	**Er**[c]
1	**A**	$CHCl_3$	91	48:52
2	**B**	$CHCl_3$	89	49:51
3	**C**	$CHCl_3$	87	49:51
4	**D**	$CHCl_3$	89	75:25
5	**E**	$CHCl_3$	90	48:52
6	**F**	$CHCl_3$	trace	/
7[d]	**D**	$CHCl_3$	91	85:15
8[e]	**D**	$CHCl_3$	90	94:6
**9[e,f]**	**D**	**$CHCl_3$**	**91**	**96:4**
10[e,g]	**D**	$CHCl_3$	87	92:8
11[e,f]	**D**	CH_2Cl_2	90	95:5
12[e,f]	**D**	MTBE	80	95:5
13[e,f]	**D**	Toluene	73	94:6
15[e,f,h]	**D**	$CHCl_3$	90	96:4
15[e,f,i]	**D**	$CHCl_3$	86	96:4
16[e,f,j]	**D**	$CHCl_3$	trace	/
17[e,f,k]	**D**	$CHCl_3$	< 10	/
18[e,f,l]	**D**	$CHCl_3$	78	96:4

[a] Conditions: **1a** (0.11 mmol), **2a** (0.10 mmol), catalyst (10 mol %), solvent (2.0 mL), room temperature, 24 h. [b] Isolated yield after flash column chromatography. [c] Determined by HPLC analysis using a chiral stationary phase. [d] **1b** was used. [e] **1c** was used. [f] **2b** was used. [g] **2n** was used. [h] K_2CO_3 (20 mol%) was added. [i] HOAc was replaced with KHSO4 (5.0 equiv) for 6 h. [j] PhCOOH (20 mol%) was added. [k] 4Å M.S. (50 mg) was added. [l] **D** (5 mol %) was used, 48 h.

Experimental measurement of rotation barrier of 3c

The rotation barriers were obtained by racemization experiments of an enantiomer via chiral HPLC analysis. The racemization constant was obtained from the slope of the first-order kinetic line ($k_{racemization} = 2 \times k_{enantiomerzation}$). Rotation barrier ($\Delta G^{\ddagger}$) was obtained from the eyring equation. ($R = 8.31451\ J\cdot K^{-1}\cdot mol^{-1}$, $h = 6.62608 \times 10^{-34}\ J\cdot s$ and $k_B = 1.38066\ 10^{-23}\ J\cdot K^{-1}$).

Eyring Equation: $k_{enantiomerization} = \frac{k_B * T}{h} * e^{\frac{-\Delta G^{\ddagger}}{RT}}$

Half-life time: $t_{1/2} = \frac{\ln 2}{k_{racemetization}}$

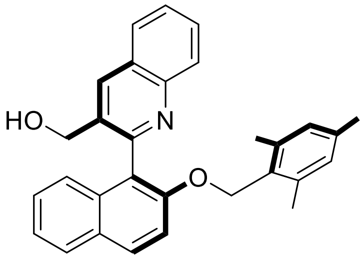

3c

Rotation barrier for 3c: (10 mg) was dissolved in *i*PrOH (20 mL) and stirred at 80 °C for about 56 h. Samples (5 μL) of this solution were diluted by *n*hexane (5 μL) andinjected on Chiralpak IF (*n*hexane/*i*PrOH = 90/10, 1.0 mL/min, λ = 254 nm) to monitor the percentage decrease of the second eluted enantiomer over time.

Solvent: *i*PrOH, Temperature: 80 ℃

Time (min)	% second eluted enantiomer (%t)	ln ((%t-50)/(%0-50))
0	96.1	0
240	92.6	-0.079
480	89.5	-0.155
720	86.5	-0.234
1200	79.1	-0.460
1920	72.5	-0.717
3360	61.5	-1.388

Kinetic line: ((ln(%*t*–50)/(%0–50)) vs. time (minutes)

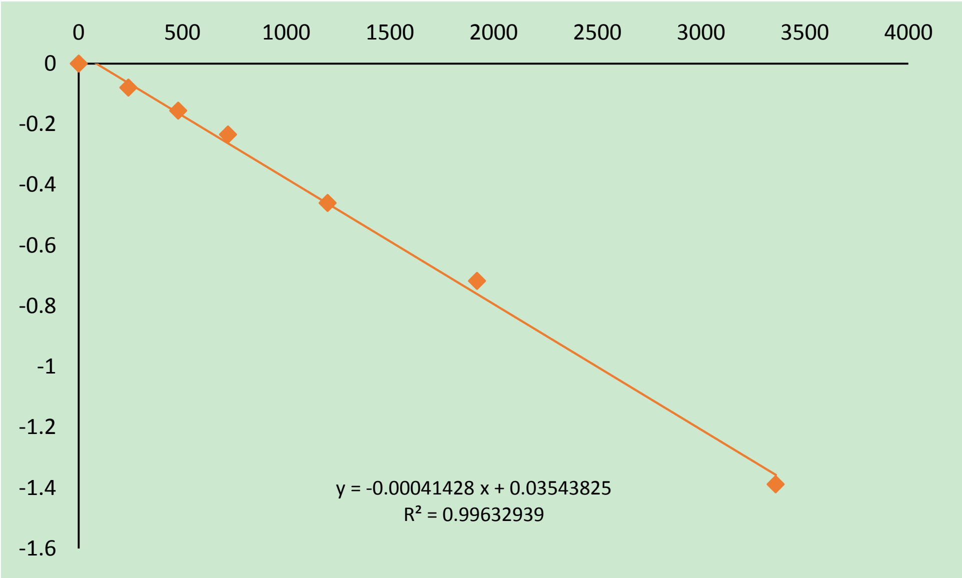

k $_{\text{enantiomerization}}$ = 3.4525E-06

k $_{\text{racemization}}$ = 6.905E-06 s^{-1}

$\Delta G^{\ddagger}$ = 121.83 kJ/mol = 29.10 kcal/mol

80 ℃ Half-life time $t_{1/2}$ = 1.0E5 s = 27.8 h

General procedure for preparation of compounds 3 and 4

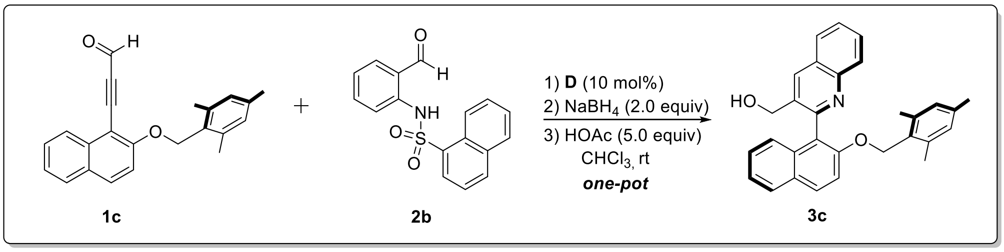

To a flame-dried Schlenk reaction tube equipped with a magnetic stir bar, was added the catalyst **D** (2.2 mg, 0.01 mmol), **1c** (39.4 mg, 0.12 mmol) and **2b** (0.10 mmol). The Schlenk tube was closed with a septum, CH_2Cl_2 (2.0 mL) was added. The mixture was then stirred at room temperature and monitored by TLC until **2b** was full consumed. Then the mixture was cooled to 0 ℃ and MeOH (1.0 mL) was added, subsequently, $NaBH_4$ (11.3 mg, 0.3 mmol) was added slowly to the mixture and stirred for 2.0 h at this temperature. After the completion of the reaction, as monitored by TLC, HOAc (5.0 equiv) was added to the mixture and then the mixture was warmed to room

temperature and stirred for another 6 h. After the reaction was completed, as monitored by TLC, saturated $NaHCO_3$ was added and stirred for another 0.5 h. Then the mixture was extracted with EtOAc. The combined organic layers was washed with water and brine, dried over anhydrous Na_2SO_4, filtered and concentrated. The residue was purified by a silica gel flash chromatography (Hexane/EtOAc) to afford the desired product **3c**.

Note: Racemic samples for the standard of chiral HPLC spectra were prepared using racemic *rac*-**A** as catalyst.

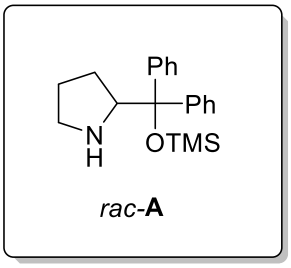

Characterization data of 3 and 4.

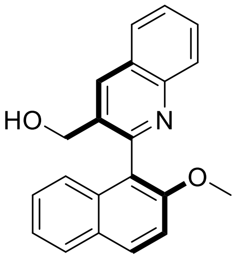

3a

3a: Colorless oil, 28 mg, 89% yield, 24 h; Colorless oil; **^{1}H NMR** (400 MHz, Chloroform-*d*) δ 8.42 (s, 1H), 8.21 (d, *J* = 8.0 Hz, 1H), 7.95 (t, *J* = 7.2 Hz, 2H), 7.85 (d, *J* = 8.1 Hz, 1H), 7.75 (t, *J* = 7.6 Hz, 1H), 7.62 (t, *J* = 7.4 Hz, 1H), 7.35 (t, *J* = 7.5 Hz, 2H), 7.28 (t, *J* = 8.0 Hz, 1H), 7.08 (d, *J* = 8.4 Hz, 1H), 4.49 (d, *J* = 13.7 Hz, 1H), 4.37 (d, *J* = 13.6 Hz, 1H), 3.79 (s, 3H), 2.65 (s, 1H). **^{13}C NMR** (100 MHz, Chloroform-*d*) δ 155.77, 154.01, 147.36, 134.87, 134.70, 133.19, 130.67, 129.42, 129.35, 129.21, 128.06, 127.75, 127.70, 127.14, 126.81, 124.27, 124.00, 122.10, 113.41, 62.32, 56.70; **HRMS** (ESI): *m/z*: calculated for $C_{21}H_{18}NO_2$: $[M + H]^+$ 316.1332, found: 316.1329; HPLC (Chiralpak IC, *i*-propanol/hexane = 20/80, flow rate 1.0 mL/min, λ = 254 nm):

t_R (major) = 12.5 min, t_R (minor) = 6.7 min, *er* = 75:25; $[\alpha]^{25}_D$ = - 129.6 (*c* = 1.0, $CHCl_3$).

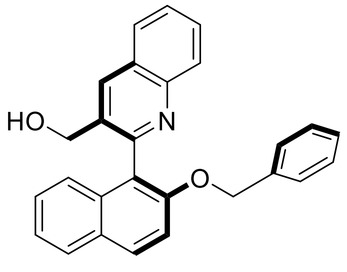

3b

3b: Colorless oil, 36 mg, 91% yield, 24 h; **^{1}H NMR** (400 MHz, Chloroform-*d*) δ 8.42 (s, 1H), 8.22 (d, *J* = 7.7 Hz, 1H), 7.93 (t, *J* = 10.1 Hz, 2H), 7.85 (d, *J* = 8.2 Hz, 1H), 7.76 (t, *J* = 7.8 Hz, 1H), 7.63 (t, *J* = 7.6 Hz, 1H), 7.50 – 7.33 (m, 2H), 7.31 (d, *J* = 8.1 Hz, 1H), 7.22 – 7.13 (m, 4H), 7.09 (d, *J* = 5.4 Hz, 2H), 5.20 – 4.85 (m, 2H), 4.57 – 4.25 (m, 2H), 2.52 (s, 1H). **^{13}C NMR** (100 MHz, Chloroform-*d*) δ 155.81, 153.39, 147.49, 136.67, 134.90, 134.76, 133.22, 130.56, 129.78, 129.40, 129.29, 128.40, 128.08, 127.82, 127.75, 127.72, 127.13, 127.09, 126.77, 124.52, 124.33, 123.77, 115.85, 72.18, 62.39; **HRMS** (ESI): *m/z*: calculated for $C_{27}H_{22}NO_2$: $[M + H]^+$ 392.1645, found: 392.1645; HPLC (Chiralpak IC, *i*-propanol/hexane = 20/80, flow rate 1.0 mL/min, λ = 254 nm): t_R (major) = 7.7 min, t_R (minor) = 6.2 min, *er* = 88:12; $[\alpha]^{25}_D$ = - 117.9 (*c* = 1.0, $CHCl_3$).

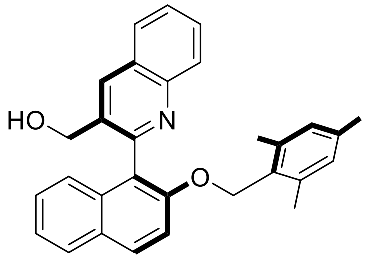

3c

3c: Colorless oil, 39 mg, 91% yield, 24 h; **^{1}H NMR** (400 MHz, Chloroform-*d*) δ 8.33 (s, 1H), 8.18 (d, *J* = 6.6 Hz, 1H), 7.99 (d, *J* = 8.9 Hz, 1H), 7.89 (t, *J* = 8.0 Hz, 2H), 7.76 (t, *J* = 7.4 Hz, 1H), 7.62 (t, *J* = 7.4 Hz, 1H), 7.56 (d, *J* = 8.9 Hz, 1H), 7.40 (t, *J* = 7.3 Hz, 1H), 7.31 (t, *J* = 7.8 Hz, 1H), 7.15 (d, *J* = 8.3 Hz, 1H), 6.68 (s, 2H), 5.18 (d, *J* = 10.2 Hz, 1H), 4.88 (d, *J* = 10.3 Hz, 1H), 4.66 – 4.03 (m, 2H), 2.61 (s, 1H), 2.17 (s, 3H), 1.99 (s, 6H). **^{13}C NMR** (100 MHz, Chloroform-*d*) δ 155.86, 153.74, 147.45, 138.15, 138.04, 135.39, 134.79, 133.30, 130.68, 130.28, 129.43, 129.26, 128.87, 128.09,

127.75, 127.65, 127.07, 126.73, 125.35, 124.76, 124.61, 117.66, 68.58, 62.58, 20.96, 19.10; **HRMS** (ESI): *m/z*: calculated for $C_{30}H_{28}NO_2$: $[M + H]^+$ 434.2115, found: 434.2119; HPLC (Chiralpak IF, *i*-propanol/hexane = 10/90, flow rate 1.0 mL/min, λ = 254 nm): t_R (major) = 8.5 min, t_R (minor) = 11.8 min, *er* = 96:4; $[\alpha]^{25}_D$ = - 159.4 (*c* = 1.0, $CHCl_3$).

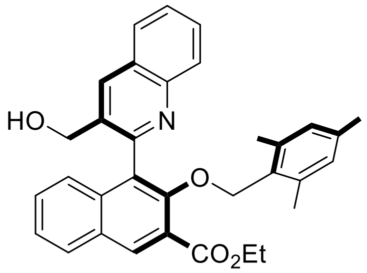

3d

3d: Colorless oil, 47 mg, 94% yield, 24 h; **^{1}H NMR** (400 MHz, Chloroform-*d*) δ 8.48 (s, 1H), 8.38 (s, 1H), 8.07 (s, 1H), 7.95 (t, *J* = 9.0 Hz, 2H), 7.76 (t, *J* = 7.3 Hz, 1H), 7.64 (t, *J* = 7.4 Hz, 1H), 7.48 (t, *J* = 7.5 Hz, 1H), 7.40 (t, *J* = 7.7 Hz, 1H), 7.20 (d, *J* = 8.4 Hz, 1H), 6.48 (s, 2H), 5.08 (d, *J* = 11.6 Hz, 1H), 4.79 (d, *J* = 11.6 Hz, 1H), 4.52 (d, *J* = 13.4 Hz, 1H), 4.42 (d, *J* = 5.6 Hz, 1H), 4.36 (q, *J* = 7.1 Hz, 2H), 2.68 (s, 1H), 2.08 (s, 3H), 1.86 (s, 6H), 1.38 (t, *J* = 7.1 Hz, 3H). **^{13}C NMR** (100 MHz, Chloroform-*d*) δ 166.19, 155.29, 152.43, 147.16, 137.81, 137.66, 135.47, 134.73, 133.58, 130.01, 129.80, 129.37, 129.06, 128.94, 128.63, 127.76, 127.58, 126.93, 126.00, 125.34, 125.21, 72.53, 62.60, 61.44, 20.96, 19.34, 14.22; **HRMS** (ESI): *m/z*: calculated for $C_{33}H_{31}NO_4$: $[M + H]^+$ 506.2326, found: 506.2331; HPLC (Chiralpak IF, *i*-propanol/hexane = 10/90, flow rate 1.0 mL/min, λ = 254 nm): t_R (major) = 11.4 min, t_R (minor) = 14.2 min, *er* = 96:4; $[\alpha]^{25}_D$ = - 138.4 (*c* = 1.0, $CHCl_3$).

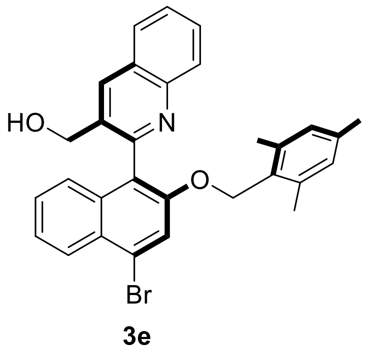

3e

3e: Colorless oil, 47 mg, 92% yield, 24 h; **^{1}H NMR** (400 MHz, Chloroform-*d*) δ 8.35

(s, 1H), 8.28 (d, J = 8.5 Hz, 1H), 8.15 (d, J = 7.6 Hz, 1H), 8.01 – 7.88 (m, 2H), 7.77 (t, J = 7.5 Hz, 1H), 7.63 (t, J = 7.4 Hz, 1H), 7.50 (t, J = 8.1 Hz, 1H), 7.35 (t, J = 7.6 Hz, 1H), 7.14 (d, J = 8.4 Hz, 1H), 6.68 (s, 2H), 5.20 (d, J = 10.2 Hz, 1H), 4.91 (d, J = 10.2 Hz, 1H), 4.72 – 4.19 (m, 2H), 2.22 (s, 1H), 2.17 (s, 3H), 2.01 (s, 6H). **^{13}C NMR** (100 MHz, Chloroform-*d*) δ 154.98, 153.29, 147.55, 138.38, 138.07, 135.39, 134.57, 133.90, 129.57, 129.28, 128.95, 128.93, 128.85, 127.88, 127.76, 127.69, 127.42, 125.96, 125.25, 124.57, 121.64, 68.66, 62.50, 20.98, 19.15; **HRMS** (ESI): *m/z*: calculated for $C_{30}H_{27}BrNO_2$: $[M + H]^+$ 512.1220, found: 512.1227; HPLC (Chiralpak ADH, *i*-propanol/hexane = 10/90, flow rate 1.0 mL/min, λ = 254 nm): t_R (major) = 5.5 min, t_R (minor) = 6.9 min, *er* = 97:3; $[\alpha]^{25}_D$ = - 151.4 (*c* = 1.0, $CHCl_3$).

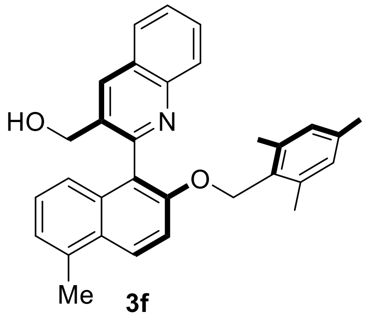

3f

3f: Colorless oil, 41 mg, 92% yield, 24 h; **^{1}H NMR** (400 MHz, Chloroform-*d*) δ 8.34 (s, 1H), 8.18 (d, J = 8.5 Hz, 2H), 7.92 (d, J = 8.0 Hz, 1H), 7.76 (t, J = 7.1 Hz, 1H), 7.62 (t, J = 8.9 Hz, 2H), 7.27 – 7.14 (m, 2H), 6.99 (d, J = 7.5 Hz, 1H), 6.68 (s, 2H), 5.20 (d, J = 10.2 Hz, 1H), 4.90 (d, J = 9.7 Hz, 1H), 4.43 - 4.34 (m, 2 H), 2.75 (s, 3H), 2.38 (s, 1H), 2.17 (s, 3H), 2.00 (s, 6H). **^{13}C NMR** (100 MHz, Chloroform-*d*) δ 154.88, 153.52, 138.12, 138.04, 135.39, 134.51, 133.54, 129.48, 129.36, 128.86, 127.73, 127.63, 126.91, 126.68, 125.48, 123.23, 117.20, 68.50, 62.77, 20.95, 19.68, 19.11; **HRMS** (ESI): *m/z*: calculated for $C_{31}H_{30}NO_2$: $[M + H]^+$ 448.2271, found: 448.2268; HPLC (Chiralpak ADH, *i*-propanol/hexane = 20/80, flow rate 1.0 mL/min, λ = 254 nm): t_R (major) = 6.8 min, t_R (minor) = 11.2 min, *er* = 98:2; $[\alpha]^{25}_D$ = - 182.6 (*c* = 1.0, $CHCl_3$).

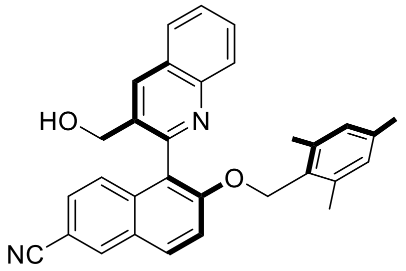

3g

3g: Colorless oil, 39 mg, 85% yield, 36 h; **^{1}H NMR** (400 MHz, Chloroform-*d*) δ 8.24 (s, 1H), 8.12 (d, *J* = 8.1 Hz, 1H), 8.04 (t, *J* = 7.0 Hz, 1H), 7.88 (t, *J* = 6.6 Hz, 1H), 7.75 (t, *J* = 7.5 Hz, 1H), 7.69 (d, *J* = 9.0 Hz, 1H), 7.61 (t, *J* = 7.2 Hz, 1H), 7.38 (d, *J* = 8.1 Hz, 1H), 7.20 (d, *J* = 8.7 Hz, 1H), 6.68 (s, 2H), 5.25 (d, *J* = 10.2 Hz, 1H), 4.94 (d, *J* = 10.1 Hz, 1H), 4.52 – 4.22 (m, 2H), 2.42 (s, 1H), 2.16 (s, 3H), 2.04 (s, 6H). **^{13}C NMR** (100 MHz, Chloroform-*d*) δ 156.27, 154.36, 147.36, 138.46, 137.96, 135.27, 134.93, 134.49, 134.25, 131.26, 129.64, 129.14, 128.99, 128.79, 128.56, 127.74, 127.69, 127.42, 127.04, 126.00, 124.89, 119.28, 118.14, 118.11, 107.66, 67.79 (d, *J* = 2.5 Hz), 62.17, 20.95, 19.18; **HRMS** (ESI): *m/z*: calculated for $C_{31}H_{27}N_2O_2$: $[M + H]^+$ 459.2067, found: 459.2069; HPLC (Chiralpak AD-H, *i*-propanol/hexane = 20/80, flow rate 1.0 mL/min, λ = 254 nm): t_R (major) = 10.2 min, t_R (minor) = 16.2 min, *er* = 99:1; $[\alpha]^{25}_{D}$ = - 165.7 (*c* = 1.0, $CHCl_3$).

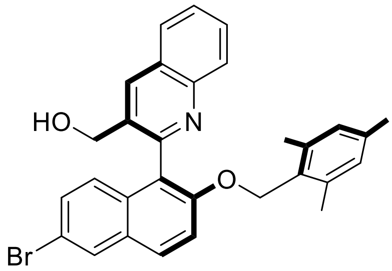

3h

3h: Colorless oil, 47 mg, 91% yield, 24 h; **^{1}H NMR** (400 MHz, Chloroform-*d*) δ 8.34 (s, 1H), 8.16 (d, *J* = 6.6 Hz, 1H), 8.04 (s, 1H), 7.91 (t, *J* = 6.0 Hz, 2H), 7.76 (t, *J* = 7.5 Hz, 1H), 7.63 (t, *J* = 7.5 Hz, 1H), 7.58 (d, *J* = 9.0 Hz, 1H), 7.37 (d, *J* = 8.9 Hz, 1H), 7.02 (d, *J* = 9.0 Hz, 1H), 6.68 (s, 2H), 5.18 (d, *J* = 10.3 Hz, 1H), 4.88 (d, *J* = 10.3 Hz, 1H), 4.36 (q, *J* = 13.6 Hz, 2H), 2.49 (s, 1H), 2.17 (s, 3H), 1.99 (s, 6H). **^{13}C NMR** (100 MHz, Chloroform-*d*) δ 155.15, 154.03, 138.28, 138.00, 134.63, 131.84, 131.21, 130.35, 130.03, 129.75, 129.17, 128.91, 127.76, 127.67, 126.97, 126.58, 118.53, 68.43, 62.52,

20.95, 19.10; **HRMS** (ESI): *m/z*: calculated for $C_{30}H_{26}BrNO_2$: $[M + H]^+$ 512.1220, found: 512.1224; HPLC (Chiralpak AD-H, *i*-propanol/hexane = 10/90, flow rate 1.0 mL/min, λ = 254 nm): t_R (major) = 14.1 min, t_R (minor) = 26.1 min, *er* = 98:2; $[\alpha]^{25}_D$ = - 137.6 (*c* = 1.0, $CHCl_3$).

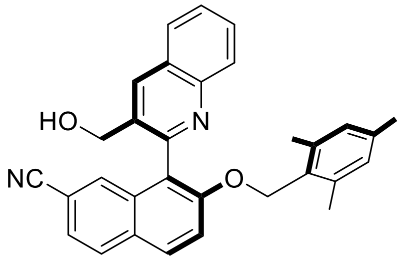

3i

3i: Colorless oil, 39 mg, 86% yield, 36 h; **^{1}H NMR** (400 MHz, Chloroform-*d*) δ 8.34 (d, *J* = 11.0 Hz, 1H), 8.13 (d, *J* = 8.4 Hz, 1H), 8.05 (d, *J* = 9.0 Hz, 1H), 7.95 (d, *J* = 8.5 Hz, 1H), 7.89 (d, *J* = 8.5 Hz, 1H), 7.78 (t, *J* = 7.6 Hz, 1H), 7.72 (d, *J* = 9.0 Hz, 1H), 7.64 (t, *J* = 7.0 Hz, 1H), 7.57 (s, 1H), 7.52 (d, *J* = 8.4 Hz, 1H), 6.68 (s, 2H), 5.23 (d, *J* = 10.2 Hz, 1H), 4.92 (d, *J* = 10.2 Hz, 1H), 4.56 – 4.24 (m, 2H), 2.47 (s, 1H), 2.17 (s, 3H), 2.01 (s, 6H). **^{13}C NMR** (100 MHz, Chloroform-*d*) δ 155.00, 154.11, 147.41, 138.45, 137.94, 135.69, 134.45, 134.43, 132.57, 131.22, 131.14, 130.69, 129.77, 129.34, 129.25, 128.99, 128.84, 127.80, 127.71, 127.16, 125.63, 124.97, 120.14, 120.10, 119.21, 110.51, 68.24, 62.36, 20.97, 19.13; **HRMS** (ESI): *m/z*: calculated for $C_{31}H_{27}N_2O_2$: $[M + H]^+$ 459.2067, found: 459.2073; HPLC (Chiralpak AD-H, *i*-propanol/hexane = 20/80, flow rate 1.0 mL/min, λ = 254 nm): t_R (major) = 8.9 min, t_R (minor) = 11.9 min, *er* = 94:6; $[\alpha]^{25}_D$ = - 201.6 (*c* = 1.0, $CHCl_3$).

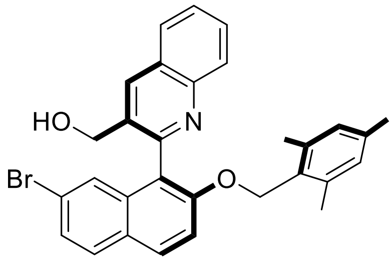

3j

3j: Colorless oil, 48 mg, 93% yield, 24 h; **^{1}H NMR** (400 MHz, Chloroform-*d*) δ 8.30 (s, 1H), 8.16 (d, *J* = 8.3 Hz, 1H), 7.95 (d, *J* = 8.8 Hz, 1H), 7.87 (d, *J* = 8.0 Hz, 1H), 7.76 (t, *J* = 9.6 Hz, 2H), 7.61 (t, *J* = 7.2 Hz, 1H), 7.56 (d, *J* = 8.9 Hz, 1H), 7.47 (d, *J* = 8.7

Hz, 1H), 7.30 (s, 1H), 6.66 (s, 2H), 5.18 (d, *J* = 10.1 Hz, 1H), 4.87 (d, *J* = 9.9 Hz, 1H), 4.51 – 4.20 (m, 2H), 2.55 (s, 1H), 2.15 (s, 3H), 1.99 (s, 6H). **^{13}C NMR** (100 MHz, Chloroform-*d*) δ 154.93, 154.57, 147.44, 138.26, 137.97, 135.51, 134.59, 134.48, 130.61, 129.75, 129.50, 129.29, 129.13, 128.91, 128.54, 128.06, 127.78, 127.69, 126.88, 126.83, 124.46, 121.76, 117.66, 68.30, 62.46, 20.96, 19.11; **HRMS** (ESI): *m/z*: calculated for $C_{30}H_{27}BrNO_2$: $[M + H]^+$ 512.1220, found: 512.1226; HPLC (Chiralpak AD-H, *i*-propanol/hexane = 10/90, flow rate 1.0 mL/min, λ = 254 nm): t_R (major) = 15.5 min, t_R (minor) = 28.4 min, *er* = 98:2; $[\alpha]^{25}_D$ = - 156.2 (*c* = 1.0, $CHCl_3$).

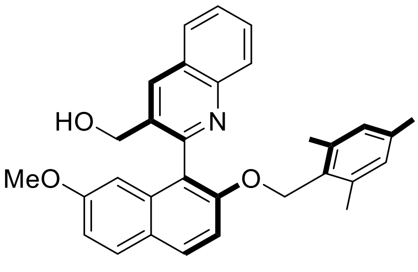

3k

3k: Colorless oil, 44 mg, 95% yield, 24 h; **^{1}H NMR** (400 MHz, Chloroform-*d*) δ 8.35 (s, 1H), 8.19 (d, *J* = 8.3 Hz, 1H), 7.93 – 7.90 (m, 2H), 7.80 – 7.74 (m, 2H), 7.62 (t, *J* = 7.5 Hz, 1H), 7.40 (d, *J* = 8.9 Hz, 1H), 7.07 (dd, *J* = 9.0, 2.5 Hz, 1H), 6.67 (s, 2H), 6.44 (s, 1H), 5.16 (d, *J* = 10.3 Hz, 1H), 4.86 (d, *J* = 10.3 Hz, 1H), 4.45 – 4.35 (m, 2H), 3.56 (s, 3H), 2.55 (s, 1H), 2.17 (s, 3H), 1.98 (s, 6H). **^{13}C NMR** (100 MHz, Chloroform-*d*) δ 158.60, 156.04, 154.49, 147.51, 138.12, 138.03, 135.63, 134.78, 134.58, 130.41, 129.73, 129.44, 129.24, 128.86, 127.72, 127.67, 126.74, 125.86, 124.17, 116.98, 114.94, 103.40, 68.40, 62.65, 55.12, 20.97, 19.10; **HRMS** (ESI): *m/z*: calculated for $C_{31}H_{29}NO_3$: $[M + H]^+$ 464.2220, found: 464.2217; HPLC (Chiralpak IC, *i*-propanol/hexane = 10/90, flow rate 1.0 mL/min, λ = 254 nm): t_R (major) = 12.4 min, t_R (minor) = 11.1 min, *er* = 98:2; $[\alpha]^{25}_D$ = - 145.3 (*c* = 1.0, $CHCl_3$).

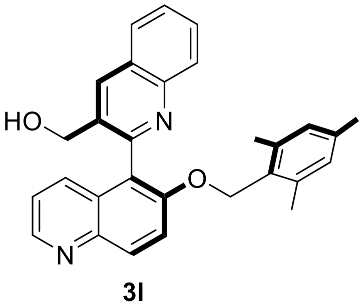

3l

3l: Colorless oil, 36 mg, 84% yield, 24 h; **^{1}H NMR** (400 MHz, Chloroform-*d*) δ 8.78 (d, *J* = 4.2 Hz, 1H), 8.36 (s, 1H), 8.30 (d, *J* = 9.2 Hz, 1H), 8.13 (d, *J* = 8.4 Hz, 1H), 7.92 (d, *J* = 8.1 Hz, 1H), 7.81 – 7.74 (m, 2H), 7.65 – 7.58 (m, 2H), 7.23 (dd, *J* = 8.6, 4.2 Hz, 1H), 6.68 (s, 2H), 5.23 (d, *J* = 10.2 Hz, 1H), 4.92 (d, *J* = 10.3 Hz, 1H), 4.49 (d, *J* = 13.4 Hz, 1H), 4.39 (d, *J* = 13.5 Hz, 1H), 2.95 (s, 1H), 2.17 (s, 3H), 2.02 (s, 6H). **^{13}C NMR** (100 MHz, Chloroform-*d*) δ 154.35, 154.07, 148.36, 147.30, 144.11, 138.36, 137.99, 135.48, 134.80, 133.84, 131.43, 129.61, 129.17, 129.00, 128.95, 128.60, 127.76, 127.68, 127.01, 124.91, 121.75, 120.86, 68.36, 62.29, 20.95, 19.12; **HRMS** (ESI): *m/z*: calculated for $C_{29}H_{27}N_2O_2$: $[M + H]^+$ 435.2067, found: 435.2068; HPLC (Chiralpak AD-H, *i*-propanol/hexane = 10/90, flow rate 1.0 mL/min, λ = 254 nm): t_R (major) = 13.2 min, t_R (minor) = 8.3 min, *er* = 99:1; $[\alpha]^{25}_D$ = - 117.6 (*c* = 1.0, $CHCl_3$).

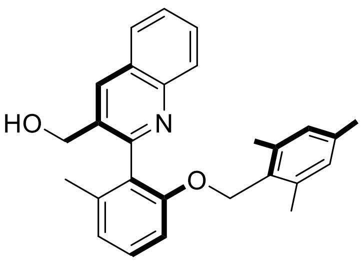

3m

3m: Colorless oil, 37 mg, 92% yield, 24 h; ^{1}H NMR (400 MHz, Chloroform-*d*) δ 8.19 (s, 1H), 8.14 (d, *J* = 8.4 Hz, 1H), 7.80 (d, *J* = 7.9 Hz, 1H), 7.71 (t, *J* = 7.7 Hz, 1H), 7.54 (t, *J* = 7.5 Hz, 1H), 7.37 (t, *J* = 8.0 Hz, 1H), 7.13 (d, *J* = 8.2 Hz, 1H), 7.03 (d, *J* = 7.6 Hz, 1H), 6.66 (s, 2H), 5.08 (d, *J* = 10.1 Hz, 1H), 4.78 (d, *J* = 10.1 Hz, 1H), 4.49 - 4.38 (m, 2H), 2.63 (dd, *J* = 8.0, 4.1 Hz, 1H), 2.14 (s, 3H), 2.01 (s, 6H), 2.00 (s, 3H). ^{13}C NMR (100 MHz, Chloroform-*d*) δ 156.88, 156.37, 147.43, 138.30, 138.01, 137.97, 135.20, 133.70, 129.89, 129.46, 129.33, 129.20, 129.07, 128.83, 127.60, 127.55, 126.43, 124.38, 112.39, 66.94, 62.62, 20.92, 19.72, 19.12; **HRMS** (ESI): *m/z*: calculated for $C_{27}H_{28}NO_2$: $[M + H]^+$ 398.2115, found: 398.2119; HPLC (Chiralpak AD-H, *i*-propanol/hexane = 5/95, flow rate 1.0 mL/min, λ = 254 nm): t_R (major) = 20.1 min, t_R (minor) = 24.5 min, *er* = 96:4; $[\alpha]^{25}_D$ = -118.3 (*c* = 1.0, $CHCl_3$).

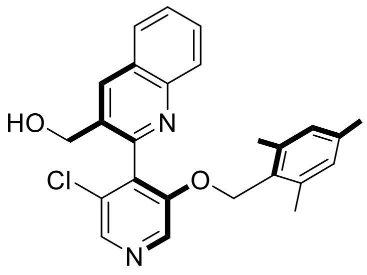

3n

3n: Colorless oil, 34 mg, 81% yield, 48 h; **^{1}H NMR** (400 MHz, Chloroform-*d*) δ 8.55 (s, 1H), 8.46 (s, 1H), 8.28 (s, 1H), 8.11 (d, *J* = 8.4 Hz, 1H), 7.84 (d, *J* = 7.9 Hz, 1H), 7.73 (t, *J* = 8.3 Hz, 1H), 7.58 (t, *J* = 7.5 Hz, 1H), 6.70 (s, 2H), 5.18 (d, *J* = 10.2 Hz, 1H), 4.98 (d, *J* = 10.2 Hz, 1H), 4.52 (q, *J* = 13.5 Hz, 2H), 2.35 (s, 1H), 2.16 (s, 3H), 2.09 (s, 6H). **^{13}C NMR** (100 MHz, Chloroform-*d*) δ 153.40, 151.61, 147.24, 143.23, 138.61, 138.03, 136.38, 135.23, 135.03, 132.75, 131.26, 129.72, 129.20, 129.00, 128.42, 127.86, 127.71, 127.24, 67.78, 61.90, 20.93, 19.25; **HRMS** (ESI): *m/z*: calculated for $C_{29}H_{27}N_2O_2$: $[M + H]^+$ 419.1521, found: 419.1524; HPLC (Chiralpak AD-H, *i*-propanol/hexane = 10/90, flow rate 1.0 mL/min, λ = 254 nm): t_R (major) = 7.7 min, t_R (minor) = 10.3 min, *er* = 90:10; $[\alpha]^{25}_D$ = - 113.7 (*c* = 1.0, $CHCl_3$).

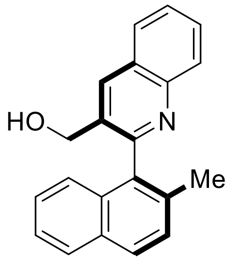

3o

3o: Colorless oil, 26.1 mg, 87% yield, 24 h; **^{1}H NMR** (400 MHz, Chloroform-*d*) δ 8.39 (s, 1H), 8.17 (d, *J* = 8.4 Hz, 1H), 7.92 (d, *J* = 8.1 Hz, 1H), 7.81 (dd, *J* = 12.6, 8.3 Hz, 2H), 7.74 (t, *J* = 7.6 Hz, 1H), 7.62 (t, *J* = 7.5 Hz, 1H), 7.41 – 7.32 (m, 2H), 7.23 (t, *J* = 7.6 Hz, 1H), 7.00 (d, *J* = 8.4 Hz, 1H), 4.34 – 4.10 (m, 2H), 2.85 (s, 1H), 2.09 (s, 3H). **^{13}C NMR** (100 MHz, Chloroform-*d*) δ 157.87, 147.23, 134.52, 134.05, 133.53, 132.05, 131.90, 129.43, 129.05, 128.67, 128.48, 128.08, 127.70, 127.63, 126.80, 126.54, 125.11, 124.62, 61.38, 19.83; **HRMS** (ESI): *m/z*: calculated for $C_{21}H_{18}NO$: $[M + H]^+$ 300.1388, found: 300.1395; HPLC (Chiralpak IC, *i*-propanol/hexane = 30/70, flow rate 1.0 mL/min, λ = 254 nm): t_R (major) = 4.5 min, t_R (minor) = 4.2 min, *er* = 69:31; $[\alpha]^{25}_D$ =

- 82.6 (c = 1.0, $CHCl_3$).

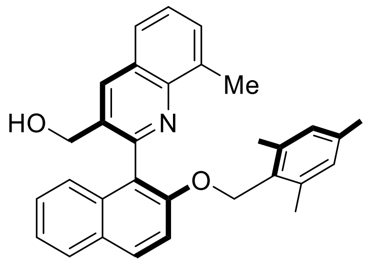

` **4a**

4a: Colorless oil, 38 mg, 86%yield, 48 h; **^{1}H NMR** (400 MHz, Chloroform-*d*) δ 8.31 (s, 1H), 7.99 (d, J = 9.0 Hz, 1H), 7.91 (d, J = 8.2 Hz, 1H), 7.77 (d, J = 8.1 Hz, 1H), 7.60 (d, J = 6.9 Hz, 1H), 7.53 - 7.49 (m, 2H), 7.42 (t, J = 7.0 Hz, 1H), 7.32 (t, J = 7.6 Hz, 1H), 7.22 (d, J = 8.5 Hz, 1H), 6.68 (s, 2H), 5.16 (d, J = 10.5 Hz, 1H), 4.91 (d, J = 10.5 Hz, 1H), 4.57 – 4.20 (m, 2H), 2.76 (s, 3H), 2.38 (dd, J = 8.0, 4.4 Hz, 1H), 2.18 (s, 3H), 2.00 (s, 6H). **^{13}C NMR** (100 MHz, Chloroform-*d*) δ 154.52, 153.67, 146.75, 138.09, 138.05, 137.47, 135.62, 134.20, 133.61, 130.37, 130.26, 129.55, 129.30, 128.84, 127.99, 127.66, 126.78, 126.67, 126.37, 125.62, 125.20, 124.57, 118.56, 69.03, 62.95, 20.93, 19.06, 18.22; **HRMS** (ESI): *m/z*: calculated for $C_{31}H_{30}NO_2$: $[M + H]^+$ 448.2271, found: 448.2270; HPLC (Chiralpak AD-H, *i*-propanol/hexane = 20/80, flow rate 1.0 mL/min, λ = 254 nm): t_R (major) = 11.9 min, t_R (minor) = 8.0 min, *er* = 94:6; $[\alpha]^{25}_{D}$ = - 131.5 (c = 1.0, $CHCl_3$).

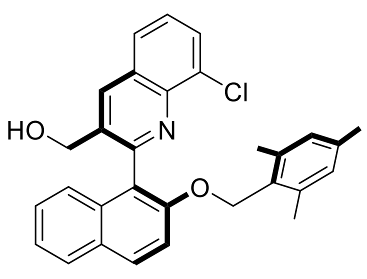

4b

4b: Colorless oil, 40 mg, 85% yield, 48 h; **^{1}H NMR** (400 MHz, Chloroform-*d*) δ 8.39 (s, 1H), 8.00 (d, J = 9.0 Hz, 1H), 7.95 – 7.80 (m, 3H), 7.60 – 7.49 (m, 2H), 7.41 (t, J = 7.8 Hz, 1H), 7.33 (t, J = 7.6 Hz, 1H), 7.18 (d, J = 8.4 Hz, 1H), 6.69 (s, 2H), 5.21 (d, J = 10.4 Hz, 1H), 4.95 (d, J = 10.4 Hz, 1H), 4.65 – 4.26 (m, 2H), 2.23 (s, 1H), 2.18 (s, 3H), 2.04 (s, 6H). **^{13}C NMR** (100 MHz, Chloroform-*d*) δ 156.52, 153.88, 143.71,

138.11, 138.07, 135.78, 135.36, 133.58, 133.28, 130.70, 130.30, 129.57, 129.38, 129.06, 128.84, 128.06, 126.98, 126.73, 126.51, 125.36, 124.79, 124.60, 118.08, 68.86, 62.40, 20.93, 19.13; **HRMS** (ESI): *m/z*: calculated for $C_{30}H_{27}ClNO_2$: $[M + H]^+$ 468.1725, found: 468.1730; HPLC (Chiralpak IC, *i*-propanol/hexane = 30/70, flow rate 1.0 mL/min, λ = 254 nm): t_R (major) = 17.3 min, t_R (minor) = 8.7 min, *er* = 93:7; $[\alpha]^{25}_D$ = - 109.8 (*c* = 1.0, $CHCl_3$).

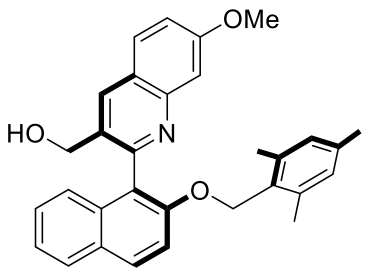

4c

4c: Colorless oil, 43 mg, 93% yield, 24 h; **^{1}H NMR** (400 MHz, Chloroform-*d*) δ 8.26 (s, 1H), 8.00 (d, *J* = 9.0 Hz, 1H), 7.89 (d, *J* = 8.0 Hz, 1H), 7.80 (d, *J* = 9.0 Hz, 1H), 7.57 (d, *J* = 9.0 Hz, 1H), 7.51 (s, 1H), 7.41 (t, *J* = 8.0 Hz, 1H), 7.33 (t, *J* = 7.0 Hz, 1H), 7.30 – 7.25 (m, 2H), 7.19 (d, *J* = 8.4 Hz, 1H), 6.70 (s, 2H), 5.19 (d, *J* = 10.3 Hz, 1H), 4.89 (d, *J* = 10.2 Hz, 1H), 4.57 – 4.18 (m, 2H), 3.95 (s, 3H), 2.50 (s, 1H), 2.18 (s, 3H), 2.01 (s, 6H). **^{13}C NMR** (100 MHz, Chloroform-*d*) δ 160.81, 156.04, 153.62, 149.22, 138.20, 138.05, 135.79, 133.40, 132.50, 130.59, 130.37, 129.39, 128.89, 128.66, 128.08, 127.04, 125.74, 124.92, 124.63, 122.96, 120.06, 117.80, 107.20, 68.71, 62.88, 55.56, 20.98, 19.11; **HRMS** (ESI): *m/z*: calculated for $C_{31}H_{30}NO_3$: $[M + H]^+$ 464.2220, found: 464.2217; HPLC (Chiralpak IF, *i*-propanol/hexane = 10/90, flow rate 1.0 mL/min, λ = 254 nm): t_R (major) = 15.4 min, t_R (minor) = 18.9 min, *er* = 98:2; $[\alpha]^{25}_D$ = - 152.1 (*c* = 1.0, $CHCl_3$).

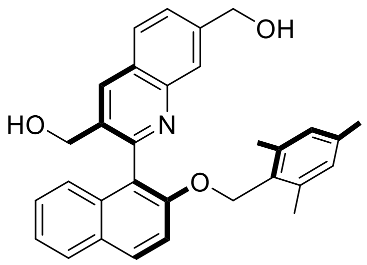

4d

4d: Colorless oil, 42 mg, 91% yield, 24 h; **^{1}H NMR** (400 MHz, Chloroform-*d*) δ 8.28 (s, 1H), 8.12 (s, 1H), 8.00 (d, *J* = 9.0 Hz, 1H), 7.88 (d, *J* = 8.1 Hz, 1H), 7.82 (d, *J* = 8.4 Hz, 1H), 7.55 (t, *J* = 7.9 Hz, 2H), 7.39 (t, *J* = 7.4 Hz, 1H), 7.30 (d, *J* = 8.3 Hz, 1H), 7.11 (d, *J* = 8.4 Hz, 1H), 6.67 (s, 2H), 5.18 (d, *J* = 10.3 Hz, 1H), 4.89 (d, *J* = 10.3 Hz, 1H), 4.81 (s, 2H), 4.38 (q, *J* = 13.4 Hz, 2H), 2.52 (s, 2H), 2.16 (s, 3H), 2.00 (s, 6H). **^{13}C NMR** (100 MHz, Chloroform-*d*) δ 156.14, 153.68, 147.49, 142.49, 138.15, 138.00, 135.35, 134.72, 133.35, 130.68, 130.28, 129.41, 128.87, 128.09, 127.94, 127.08, 127.04, 126.46, 125.91, 125.20, 124.73, 124.58, 117.56, 68.49, 65.16, 62.72, 20.92, 19.09; **HRMS** (ESI): *m/z*: calculated for $C_{31}H_{30}NO_3$: $[M + H]^+$ 464.2218, found: 464.2218; HPLC (Chiralpak IF, *i*-propanol/hexane = 10/90, flow rate 1.0 mL/min, λ = 254 nm): t_R (major) = 10.2 min, t_R (minor) = 16.2 min, *er* = > 99:1; $[\alpha]^{25}_D$ = - 172.3 (*c* = 1.0, $CHCl_3$).

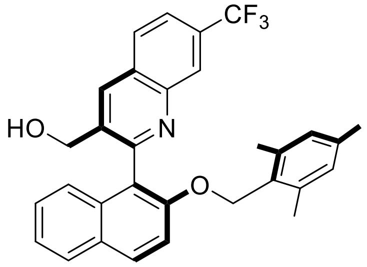

4e

4e: Colorless oil, 47 mg, 93% yield, 24 h; ^{1}H NMR (400 MHz, Chloroform-*d*) δ 8.48 (s, 1H), 8.39 (s, 1H), 8.02 (t, *J* = 10.0 Hz, 2H), 7.91 (d, *J* = 8.1 Hz, 1H), 7.78 (d, *J* = 8.5 Hz, 1H), 7.60 (d, *J* = 9.0 Hz, 1H), 7.41 (t, *J* = 7.4 Hz, 1H), 7.33 (t, *J* = 7.6 Hz, 2H), 7.10 (d, *J* = 8.4 Hz, 1H), 6.67 (s, 2H), 5.23 (d, *J* = 10.4 Hz, 1H), 4.94 (d, *J* = 10.4 Hz, 1H), 4.47 (d, *J* = 13.8 Hz, 1H), 4.38 (d, *J* = 13.8 Hz, 1H), 2.32 (s, 1H), 2.17 (s, 3H), 2.01 (s, 6H). ^{13}C NMR (100 MHz, Chloroform-*d*) δ 157.48, 153.71, 146.18, 138.23, 137.94,

137.00, 134.62, 133.00, 131.00, 130.13, 129.35, 129.12, 128.89, 128.80, 128.21, 127.30, 127.07, 125.37, 124.67, 124.39, 122.40, 122.36, 117.22, 68.24, 62.29, 20.90, 19.13; **HRMS** (ESI): *m/z*: calculated for $C_{31}H_{27}F_3NO_2$: $[M + H]^+$ 502.1988, found: 502.1994; HPLC (Chiralpak IF, *i*-propanol/hexane = 10/90, flow rate 1.0 mL/min, λ = 254 nm): t_R (major) = 7.2 min, t_R (minor) = 8.3 min, *er* = 97:3; $[\alpha]^{25}_D$ = - 116.2 (*c* = 1.0, $CHCl_3$).

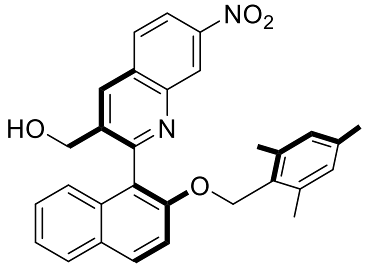

4f

4f: Colorless oil, 46 mg, 96% yield, 36 h; **^{1}H NMR** (400 MHz, Chloroform-*d*) δ 9.08 (s, 1H), 8.48 (s, 1H), 8.39 (d, *J* = 7.6 Hz, 1H), 8.05 (d, *J* = 8.9 Hz, 2H), 7.90 (d, *J* = 7.4 Hz, 1H), 7.60 (d, *J* = 8.9 Hz, 1H), 7.46 – 7.31 (m, 2H), 7.08 (s, 1H), 6.67 (s, 2H), 5.24 (d, *J* = 10.5 Hz, 1H), 4.95 (d, *J* = 10.5 Hz, 1H), 4.65 – 4.33 (m, 2H), 2.16 (s, 3H), 2.02 (s, 6H). **^{13}C NMR** (100 MHz, Chloroform-*d*) δ 158.44, 153.79, 148.04, 138.50, 138.30, 137.93, 134.42, 132.73, 131.34, 130.85, 129.97, 129.30, 129.24, 128.90, 128.32, 127.52, 125.34, 124.75, 124.16, 120.36, 116.92, 68.04, 62.02, 20.94, 19.23; **HRMS** (ESI): *m/z*: calculated for $C_{30}H_{27}N_2O_4$: $[M + H]^+$ 479.1965, found: 479.1969; HPLC (Chiralpak IF, *i*-propanol/hexane = 20/80, flow rate 1.0 mL/min, λ = 254 nm): t_R (major) = 10.5 min, t_R (minor) = 8.9 min, *er* = 99:1; $[\alpha]^{25}_D$ = - 147.3 (*c* = 1.0, $CHCl_3$).

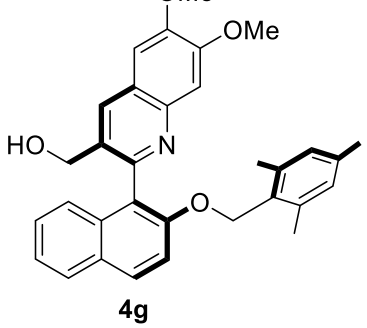

4g

4g: Colorless oil, 44 mg, 90% yield, 36 h; **^{1}H NMR** (400 MHz, Chloroform-*d*) δ 8.20 (s, 1H), 7.99 (d, *J* = 9.0 Hz, 1H), 7.88 (d, *J* = 8.1 Hz, 1H), 7.56 (d, *J* = 9.1 Hz, 1H), 7.52

(s, 1H), 7.39 (t, J = 7.5 Hz, 1H), 7.32 (t, J = 7.7 Hz, 1H), 7.19 (s, 1H), 7.15 (s, 1H), 6.70 (s, 2H), 5.18 (d, J = 10.2 Hz, 1H), 4.89 (s, 1H), 4.35 (s, 2H), 4.08 (s, 3H), 4.03 (s, 3H), 2.55 (s, 1H), 2.18 (s, 3H), 2.01 (s, 6H). **^{13}C NMR** (100 MHz, Chloroform-*d*) δ 153.71, 152.51, 150.04, 144.57, 138.18, 138.07, 134.52, 133.54, 132.88, 130.51, 130.40, 129.41, 128.88, 128.07, 127.00, 124.98, 124.61, 123.38, 117.89, 107.94, 104.87, 68.76, 62.97, 56.17, 20.99, 19.12; **HRMS** (ESI): *m/z*: calculated for $C_{32}H_{32}NO_4$: $[M + H]^+$ 494.2326, found: 494.2328; HPLC (Chiralpak ADH, *i*-propanol/hexane = 10/90, flow rate 1.0 mL/min, λ = 254 nm): t_R (major) = 14.9 min, t_R (minor) = 29.5 min, *er* = 99:1; $[\alpha]^{25}_D$ = - 162.3 (*c* = 1.0, $CHCl_3$).

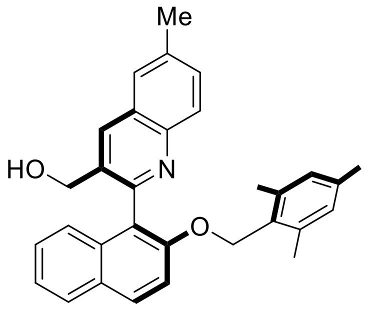

4h

4h: Colorless oil, 42 mg, 93% yield, 24 h; **^{1}H NMR** (400 MHz, Chloroform-*d*) δ 8.20 (s, 1H), 8.07 (s, 1H), 7.97 (d, J = 9.0 Hz, 1H), 7.87 (d, J = 8.2 Hz, 1H), 7.63 – 7.48 (m, 3H), 7.39 (t, J = 7.5 Hz, 1H), 7.30 (t, J = 8.0 Hz, 1H), 7.13 (d, J = 8.5 Hz, 1H), 6.67 (s, 2H), 5.17 (d, J = 10.2 Hz, 1H), 4.88 (d, J = 10.3 Hz, 1H), 4.56 – 4.05 (m, 2H), 2.75 (s, 1H), 2.60 (s, 3H), 2.16 (s, 3H), 2.00 (s, 6H). **^{13}C NMR** (100 MHz, Chloroform-*d*) δ 154.76, 153.76, 146.04, 138.13, 138.06, 136.59, 134.77, 133.37, 131.75, 130.64, 130.25, 129.45, 128.88, 128.08, 127.79, 127.03, 126.46, 124.78, 124.57, 117.58, 68.51, 62.54, 21.72, 20.97, 19.14; **HRMS** (ESI): *m/z*: calculated for $C_{31}H_{30}NO_2$: $[M + H]^+$ 448.2271, found: 448.2277; HPLC (Chiralpak IF, *i*-propanol/hexane = 20/80, flow rate 1.0 mL/min, λ = 254 nm): t_R (major) = 7.1 min, t_R (minor) = 10.2 min, *er* = 98:2; $[\alpha]^{25}_D$ = - 182.4 (*c* = 1.0, $CHCl_3$).

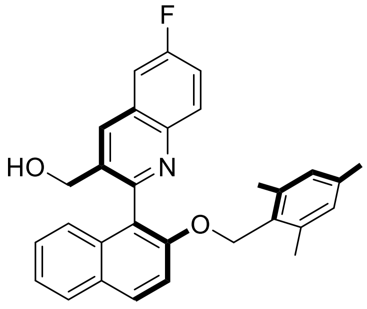

4i

4i: Colorless oil, 42 mg, 93% yield, 24 h; **^{1}H NMR** (400 MHz, Chloroform-*d*) δ 8.24 (s, 1H), 8.15 (t, *J* = 7.3 Hz, 1H), 7.99 (d, *J* = 9.0 Hz, 1H), 7.89 (d, *J* = 8.1 Hz, 1H), 7.56 (d, *J* = 9.0 Hz, 1H), 7.53 – 7.44 (m, 2H), 7.40 (t, *J* = 7.4 Hz, 1H), 7.32 (t, *J* = 7.6 Hz, 1H), 7.12 (d, *J* = 8.4 Hz, 1H), 6.68 (s, 2H), 5.19 (d, *J* = 10.3 Hz, 1H), 4.89 (d, *J* = 10.3 Hz, 1H), 4.40 (d, *J* = 13.7 Hz, 1H), 4.31 (dd, *J* = 13.9, 4.9 Hz, 1H), 2.56 (s, 1H), 2.17 (s, 3H), 2.00 (s, 6H). **^{13}C NMR** (100 MHz, Chloroform-*d*) δ 161.86, 159.40, 155.11, 153.78, 144.45, 131.71, 130.78, 130.20, 129.42, 128.88, 128.37 (d, *J* = 10.2 Hz), 128.14, 127.14, 124.84, 124.60, 124.57, 119.55 (d, *J* = 25.9 Hz), 117.45, 110.53 (d, *J* = 21.6 Hz), 68.39, 62.29, 20.94, 19.10; **HRMS** (ESI): *m/z*: calculated for $C_{30}H_{27}FNO_2$: [M + H]$^+$ 452.2020, found: 452.2025; HPLC (Chiralpak IF, *i*-propanol/hexane = 10/90, flow rate 1.0 mL/min, λ = 254 nm): t_R (major) = 10.2 min, t_R (minor) = 15.8 min, *ee* = 98:2; $[\alpha]^{25}_D$ = - 129.7 (*c* = 1.0, $CHCl_3$).

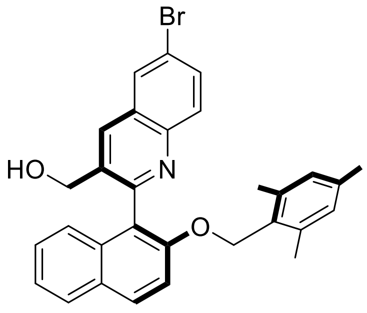

4j

4j: Colorless oil, 49 mg, 95% yield, 24 h; **^{1}H NMR** (400 MHz, Chloroform-*d*) δ 8.09 (s, 1H), 7.99 (dd, *J* = 9.0, 5.0 Hz, 2H), 7.91 (d, *J* = 2.0 Hz, 1H), 7.88 (d, *J* = 8.1 Hz, 1H), 7.77 (dd, *J* = 8.9, 2.2 Hz, 1H), 7.55 (d, *J* = 9.0 Hz, 1H), 7.40 (t, *J* = 7.0 Hz, 1H), 7.31 (t, *J* = 7.6 Hz, 1H), 7.08 (d, *J* = 8.4 Hz, 1H), 6.67 (s, 2H), 5.19 (d, *J* = 10.3 Hz, 1H), 4.88 (d, *J* = 10.2 Hz, 1H), 4.38 (d, *J* = 13.8 Hz, 1H), 4.27 (dd, *J* = 13.8, 5.5 Hz, 1H), 2.69 (s, 1H), 2.15 (s, 3H), 2.01 (s, 6H). **^{13}C NMR** (100 MHz, Chloroform-*d*) δ

156.25, 153.70, 145.83, 138.20, 137.96, 135.83, 133.81, 133.06, 132.76, 130.89, 130.84, 130.10, 129.56, 129.38, 128.91, 128.78, 128.16, 127.19, 124.59, 124.47, 120.56, 117.15, 68.18, 62.18, 20.94, 19.15; **HRMS** (ESI): *m/z*: calculated for $C_{30}H_{27}BrNO_2$: $[M + H]^+$ 512.1220, found: 512.1214; HPLC (Chiralpak IF, *i*-propanol/hexane = 20/80, flow rate 1.0 mL/min, λ = 254 nm): t_R (major) = 6.8 min, t_R (minor) = 9.1 min, *ee* = 99:1; $[\alpha]^{25}_D$ = - 109.2 (*c* = 1.0, $CHCl_3$).

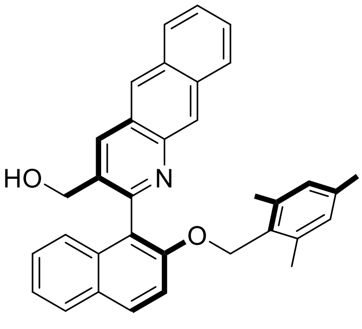

4k

4k: Colorless oil, 45 mg, 94% yield, 24 h; **^{1}H NMR** (400 MHz, Chloroform-*d*) δ 8.76 (s, 1H), 8.41 (d, *J* = 11.3 Hz, 2H), 8.07 (t, *J* = 8.8 Hz, 2H), 8.02 (d, *J* = 9.0 Hz, 1H), 7.90 (d, *J* = 8.1 Hz, 1H), 7.61 – 7.50 (m, 3H), 7.41 (t, *J* = 7.4 Hz, 1H), 7.32 (t, *J* = 7.5 Hz, 1H), 7.21 (d, *J* = 8.1 Hz, 1H), 6.3 (s, 2H), 5.22 (d, *J* = 10.3 Hz, 1H), 4.95 (d, *J* = 9.6 Hz, 1H), 4.63 – 4.21 (m, 2H), 2.61 (s, 1H), 2.11 (s, 3H), 2.01 (s, 6H). **^{13}C NMR** (100 MHz, Chloroform-*d*) δ 157.37, 153.78, 143.88, 138.16, 138.02, 135.08, 134.32, 133.91, 133.24, 132.02, 130.79, 130.21, 129.42, 128.87, 128.66, 128.16, 127.20, 126.47, 126.15, 126.03, 124.64, 117.45, 68.43, 62.70, 20.92, 19.19; **HRMS** (ESI): *m/z*: calculated for $C_{34}H_{30}NO_2$: $[M + H]^+$ 484.2271, found: 484.2273; HPLC (Chiralpak IF, *i*-propanol/hexane = 20/80, flow rate 1.0 mL/min, λ = 254 nm): t_R (major) = 9.0 min, t_R (minor) = 14.8 min, *er* = 99:1; $[\alpha]^{25}_D$ = - 197.5 (*c* = 1.0, $CHCl_3$).

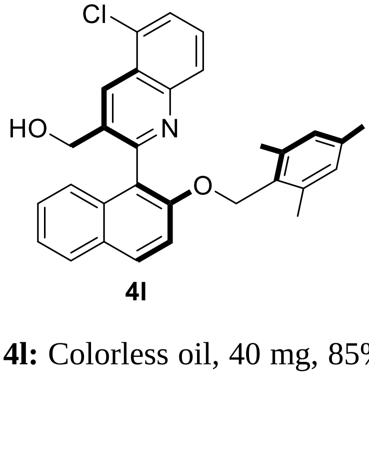

4l

4l: Colorless oil, 40 mg, 85% yield, 48 h; **^{1}H NMR** (400 MHz, Chloroform-*d*) δ 8.77

(s, 1H), 8.10 (d, J = 8.0 Hz, 1H), 8.02 (d, J = 8.9 Hz, 1H), 7.90 (d, J = 8.1 Hz, 1H), 7.71 – 7.64 (m, 2H), 7.58 (d, J = 8.9 Hz, 1H), 7.40 (t, J = 7.3 Hz, 1H), 7.32 (t, J = 7.5 Hz, 1H), 7.10 (d, J = 8.3 Hz, 1H), 6.69 (s, 2H), 5.21 (d, J = 10.3 Hz, 1H), 4.91 (d, J = 10.2 Hz, 1H), 4.48 – 4.39 (m, 2H), 2.38 (s, 1H), 2.19 (s, 3H), 2.02 (s, 6H). **^{13}C NMR** (100 MHz, Chloroform-*d*) δ 156.79, 153.77, 148.02, 138.21, 137.98, 135.87, 133.12, 132.14, 131.30, 130.91, 130.16, 129.34, 129.07, 128.91, 128.47, 128.16, 127.21, 126.75, 125.91, 124.63, 124.51, 117.31, 68.33, 62.59, 20.97, 19.15; **HRMS** (ESI): *m/z*: calculated for $C_{30}H_{27}ClNO_2$: $[M + H]^+$ 468.1725, found: 468.1720; HPLC (Chiralpak IF, *i*-propanol/hexane = 20/80, flow rate 1.0 mL/min, λ = 254 nm): t_R (major) = 5.9 min, t_R (minor) = 7.2 min, *er* = 94:6; $[\alpha]^{25}_D$ = - 147.8 (*c* = 1.0, $CHCl_3$).

Gram-scale synthesis and synthetic transformations

Cram-scale synthesis

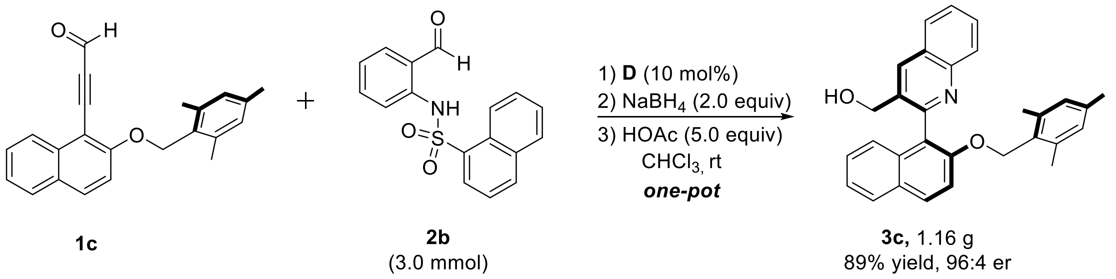

To a flame-dried Schlenk reaction tube equipped with a magnetic stir bar, was added the catalyst **D** (66.0 mg, 0.3 mmol), **1c** (1.18 g, 3.6 mmol) and **2b** (0.93 g, 3.0 mmol). The Schlenk tube was closed with a septum, CH_2Cl_2 (50.0 mL) was added. The mixture was then stirred at room temperature and monitored by TLC until **2b** was full consumed. Then the mixture was cooled to 0 ℃ and MeOH (20.0 mL) was added, subsequently, $NaBH_4$ (340.0 mg, 9.0 mmol) was added slowly to the mixture and stirred for 2.0 h at this temperature. After the completion of the reaction, as monitored by TLC, HOAc (1.5 mmol) was added to the mixture and then the mixture was warmed to room temperature and stirred for another 6 h. After the reaction was completed, as monitored by TLC, saturated $NaHCO_3$ was added and stirred for another 0.5 h. Then the mixture

was extracted with EtOAc. The combined organic layers was washed with water and brine, dried over anhydrous Na_2SO_4, filtered and concentrated. The residue was purified by a silica gel flash chromatography (Hexane/EtOAc) to afford the desired product **3c** in 89% yield with 96:4 er.

General procedure for the synthesis of 1-aryl isoquinoline analogue 5.

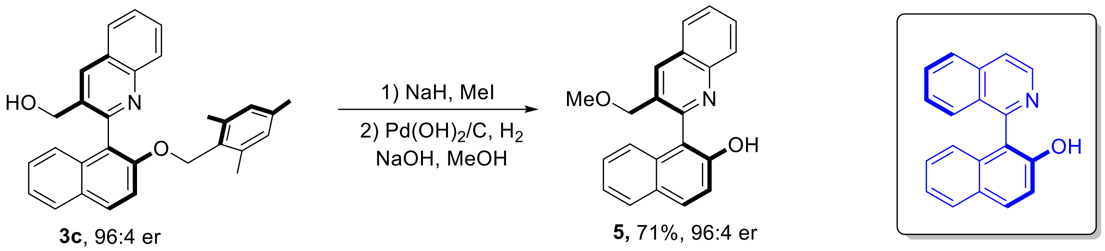

(*S*)-**3c** (43.3 mg, 0.1 mmol) was dissolved in THF (2 mL). Sodium hydride (5.0 mg, 0.2 mmol) was added portionwise at 0 ℃. The reaction mixture was stirred at 0 ℃ for 10 min. Then iodomethane (12.5 μL, 0.2 mmol) was added drop wise. The resultant mixture was stirred from 0 ℃ to room temperature for 2.0 hours and quenched by anhydrous sodium sulfate. After filtered and concentrated, the afforded residue was used directly for next step.

A solution of the residue was prepared in a mixture of MeOH/H_2O (2:1, 2.0 ml) and NaOH (4.0 mg, 0.1 mmol) in an oven-dried two-neck flask equipped with a nitrogen supply. Under nitrogen, $Pd(OH)_2/C$ (3 mg) was added. The nitrogen atmosphere was then removed under vacuum, and the apparatus was placed under 1 atm of H_2 (hydrogen balloon). The reaction mixture was stirred at room temperature for 18 hours until the reaction was completed (monitored by TLC). Then the suspended $Pd(OH)_2/C$ was removed by filtration through celite, and the filtrate was diluted with saturated NH_4Cl aqueous solution. The mixture was extracted with ethyl acetate, washed by brine and dried by anhydrous Na_2SO_4. After filtered and concentrated, the afforded residue was purified by silica gel column and given the desired product QUINOL analogue **5** in 71% yield with 96:4 er.

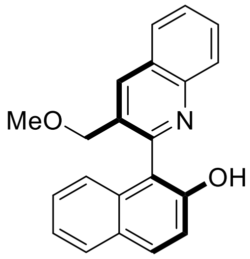

5

5, Colorless oil, 22 mg, 71%, 12 h; **^{1}H NMR** (400 MHz, Chloroform-*d*) δ 8.37 (s, 1H), 7.83 (dd, *J* = 8.2, 4.6 Hz, 2H), 7.74 (d, *J* = 8.4 Hz, 1H), 7.64 (d, *J* = 8.9 Hz, 1H), 7.59 (t, *J* = 7.6 Hz, 1H), 7.49 (t, *J* = 7.6 Hz, 1H), 7.42 – 7.26 (m, 2H), 7.09 (d, *J* = 8.3 Hz, 1H), 6.79 (d, *J* = 8.9 Hz, 1H), 4.39 (d, *J* = 13.6 Hz, 1H), 4.26 (t, *J* = 10.7 Hz, 1H), 3.28 (s, 3H). **^{13}C NMR** (100 MHz, Chloroform-*d*) δ 155.12, 152.54, 145.60, 135.79, 132.88, 132.67, 130.73, 129.55, 128.90, 128.07, 127.56, 127.49, 127.36, 126.99, 126.67, 123.67, 123.39, 120.15, 70.94, 58.70; **HRMS** (ESI): *m/z*: calculated for $C_{21}H_{18}NO_2$: $[M + H]^+$ 316.1332, found: 316.1338; HPLC (Chiralpak ADH, *i*-propanol/hexane = 05/95, flow rate 1.0 mL/min, λ = 254 nm): t_R (major) = 5.8 min, t_R (minor) = 9.1 min, *er* = 96:4; $[\alpha]^{25}_D$ = - 184.7 (*c* = 1.0, $CHCl_3$).

General procedure for the synthesis of QUINOX analogue 6.

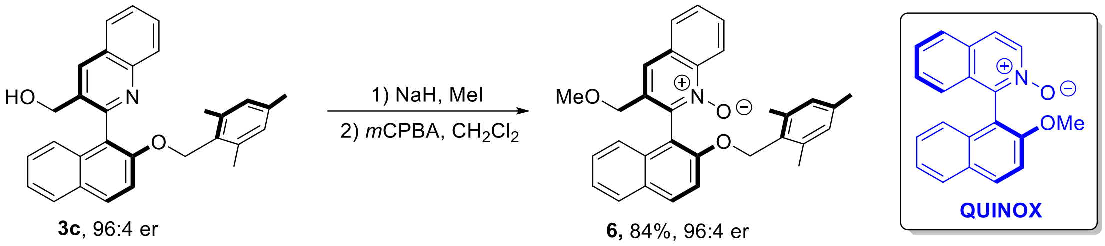

3c, 96:4 er

6, 84%, 96:4 er

(*S*)-**3c** (43.3 mg, 0.1 mmol) was dissolved in THF (2 mL). Sodium hydride (5.0 mg, 0.2 mmol) was added portionwise at 0 ℃. The reaction mixture was stirred at 0 ℃ for 10 min. Then iodomethane (12.5 μL, 0.2 mmol) was added drop wise. The resultant mixture was stirred from 0 ℃ to room temperature for 2.0 hours and quenched by anhydrous sodium sulfate. After filtered and concentrated, the afforded residue was used directly for next step.

A solution of the residue was prepared in CH_2Cl_2 (2.0 mL), the mixture was cooled to 0 ℃, then *m*CPBA (25.8 mg, 0.15 mmol) was added slowly under nitrogen. The reaction mixture was stirred at room temperature for 3.0 hours until the reaction was completed (monitored by TLC). Then reaction mixture was diluted with saturated $NaHCO_3$ aqueous solution. The mixture was extracted with CH_2Cl_2, washed by brine and dried by anhydrous Na_2SO_4. After filtered and concentrated, the afforded residue was purified by silica gel column and given the desired product QUINOX analogue **6** in 84% yield with 96:4 er.

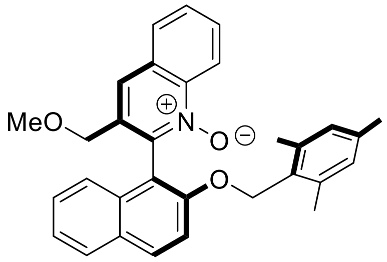

6

6, Colorless oil, 39 mg, 84%, 4 h; **^{1}H NMR** (400 MHz, Chloroform-*d*) δ 8.79 (d, *J* = 8.5 Hz, 1H), 8.06 (d, *J* = 9.0 Hz, 1H), 7.99 – 7.87 (m, 3H), 7.84 – 7.64 (m, 2H), 7.62 (d, *J* = 9.1 Hz, 1H), 7.45 – 7.31 (m, 2H), 7.16 (d, *J* = 8.2 Hz, 1H), 6.72 (s, 2H), 5.23 (d, *J* = 10.6 Hz, 1H), 5.06 (d, *J* = 10.6 Hz, 1H), 4.10 (d, *J* = 14.1 Hz, 1H), 3.95 (d, *J* = 14.1 Hz, 1H), 3.21 (s, 3H), 2.20 (s, 3H), 2.11 (s, 6H). **^{13}C NMR** (100 MHz, Chloroform-*d*) δ 154.67, 141.91, 140.94, 138.08, 138.01, 134.19, 131.96, 131.60, 129.92, 129.85, 129.71, 129.61, 128.85, 128.60, 128.42, 127.95, 127.64, 124.59, 123.43, 122.45, 120.34, 117.46, 117.03, 70.29, 67.89, 58.59, 20.97, 19.25; **HRMS** (ESI): *m/z*: calculated for $C_{31}H_{30}NO_3$: $[M + H]^+$ 464.2220, found: 464.2223; HPLC (Chiralpak ADH, *i*-propanol/hexane = 20/80, flow rate 1.0 mL/min, λ = 254 nm): t_R (major) = 31.7 min, t_R (minor) = 20.4 min, *er* = 96:4; $[\alpha]^{25}_D$ = - 192.8 (*c* = 1.0, $CHCl_3$).

Proposed mechanism

A postulated reaction pathway is proposed in Supplementary Figure 169. Initially, chiral secondary amine catalyst **D** added to alkynaldehyde **3c** and subsequently dehydrated to

produce alkynylamine cationic intermediate **I**. Then **2b** reacted with **I** via aza-Michael addition to give axially chiral allenamine intermediate **II**, which underwent an intramolecular aldol reaction to give the chiral styrene intermediate **III**. Then chiral compound **IV** was generated with the release of the catalyst **D** from intermediate **III**. Finally, chiral compound **IV** was reduced by $NaBH_4$, then dehydrated in the present of acid in one pot to deliever the desired product **3c**.

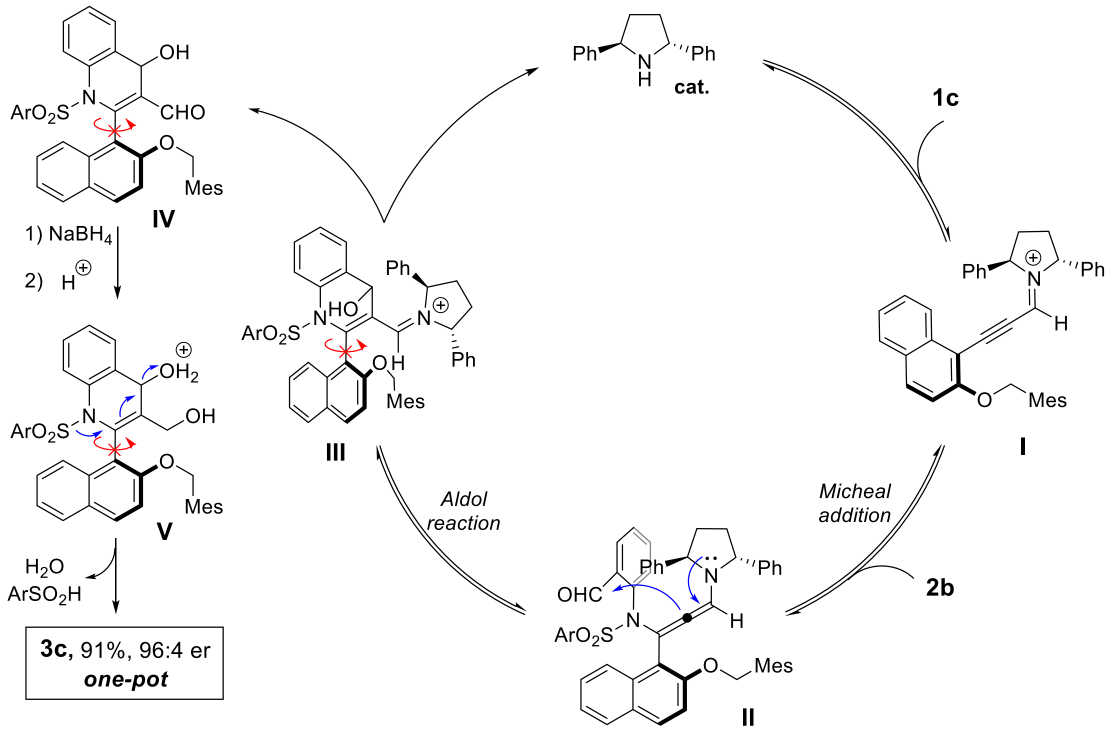

Supplementary Figure 169. Postulated mechanism

Crystal structure of 3k

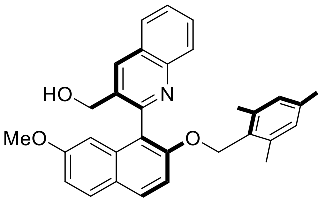

3k
(CCDC:2070558)

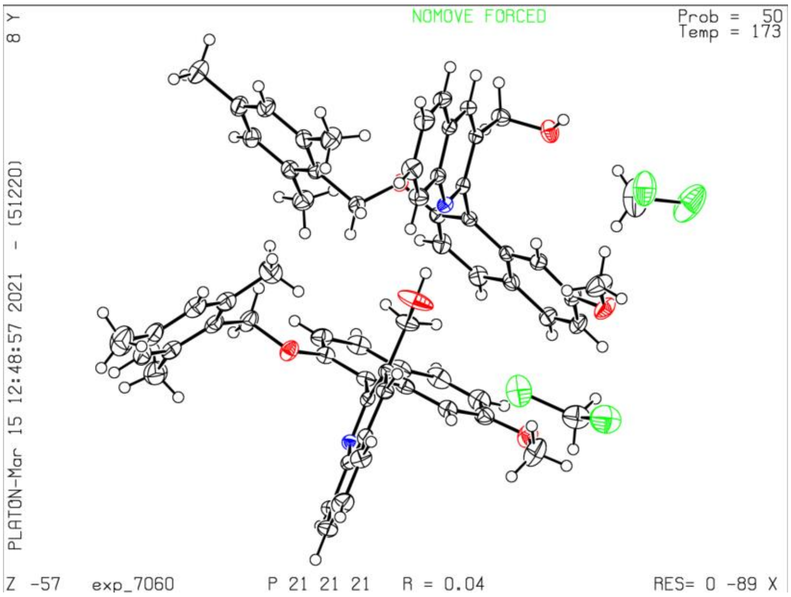

Table 2. Crystal data and structure refinement for **3k** (CCDC 2070558)

Identification code	exp_7060-**3k**
Empirical formula	$C_{32}H_{31}Cl_2NO_3$
Formula weight	548.48
Temperature/K	173.00(10)
Crystal system	orthorhombic
Space group	$P2_12_12_1$
a/Å	12.05140(10)
b/Å	17.64090(10)
c/Å	26.0504(2)
α/°	90

β/°	90
γ/°	90
Volume/Å^3	5538.25(7)
Z	8
ρ_{calc}g/cm^3	1.316
μ/mm^{-1}	2.378
F(000)	2304.0
Crystal size/mm^3	0.4 × 0.1 × 0.1
Radiation	Cu Kα (λ = 1.54184)
2Θ range for data collection/°	6.786 to 130.278
Index ranges	-14 ≤ h ≤ 14, -20 ≤ k ≤ 20, -30 ≤ l ≤ 30
Reflections collected	80650
Independent reflections	9416 [R_{int} = 0.0616, R_{sigma} = 0.0277]
Data/restraints/parameters	9416/0/703
Goodness-of-fit on F^2	1.040
Final R indexes [I>=2σ (I)]	R_1 = 0.0358, wR_2 = 0.0896
Final R indexes [all data]	R_1 = 0.0388, wR_2 = 0.0921
Largest diff. peak/hole / e Å^{-3}	0.32/-0.55
Flack parameter	-0.003(4)

Supplementary References

1. Zhao, C.-G. Guo, D.-H., Munkerup, K., Huang, K.-W., Li, F.-Y. Wang, J. *Nat. Commun.* **9**, 611 (2018).

2. Peng, L., Li, K., Xie, C.-D. Li, S., Qin, W.-L., Yan, H.-L. *Angew. Chem. Int. Ed.* **58**, 17199-17204 (2019).

3. Munday, E. S., Grove, M. A., Feoktistova, T., Brueckner, A. C., Walden, D. M., Young, C. M., Slawin, A. M. Z., Campbell, A. D., Cheong, P. H.-Y., Smith, A. D. *Angew. Chem. Int. Ed.* **59**, 7897-79059 (2020).

4. Yang, T.-T., Zhang, S.-D., Yang, S.-D., Chen, S.-S., Li, X.-W. *Org. Biomol. Chem.* **12**, 4290-4294 (2014).
